# Photoinduced Single Electron Reduction of the 4‐O‐5 Linkage in Lignin Models for C‐P Coupling Catalyzed by Bifunctional N‐Heterocyclic Carbenes

**DOI:** 10.1002/advs.202406095

**Published:** 2024-08-05

**Authors:** Qiang Liu, Ying‐Zheng Ren, Bei‐Bei Zhang, Wen‐Xin Tang, Zhi‐Xiang Wang, Lin He, Xiang‐Yu Chen

**Affiliations:** ^1^ School of Chemical Sciences University of the Chinese Academy of Sciences Beijing National Laboratory for Molecular Sciences Beijing 100049 China; ^2^ State Key Laboratory Incubation Base for Green Processing of Chemical Engineering School of Chemistry and Chemical Engineering Shihezi University Xinjiang 832000 China; ^3^ Binzhou Institute of Technology Weiqiao‐UCAS Science and Technology Park Binzhou Shandong 256606 China

**Keywords:** bifunctional N‐heterocyclic carbenes, C─O bond activation, lignin, one electron reduction, trivalent phosphines

## Abstract

Catalytic activation of C_aryl_‐O bonds is considered as a powerful strategy for the production of aromatics from lignin. However, due to the high reduction potentials of diaryl ether 4‐O‐5 linkage models, their single electron reduction remains a daunting challenge. This study presents the blue light‐induced bifunctional N‐heterocyclic carbene (NHC)‐catalyzed one‐electron reduction of diaryl ether 4‐O‐5 linkage models for the synthesis of trivalent phosphines. The H‐bond between the newly devised bifunctional NHC and diaryl ethers is responsible for the success of the single electron transfer. Furthermore, this approach demonstrates selective one‐electron reduction of unsymmetric diaryl ethers, oligomeric phenylene oxide, and lignin model.

## Graphical abstract

Upon irradiation with blue light, a bifunctional N‐heterocyclic carbene (NHC)‐catalyzed selective single electron reduction of the diaryl ether 4‐O‐5 linkage models is reported. Mechanistic studies suggest that the H‐bond between NHC and diaryl ethers facilitate the single electron transfer from the NHC radical anion to the diaryl ethers.

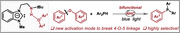

## 1 Introduction

**Figure 1:**
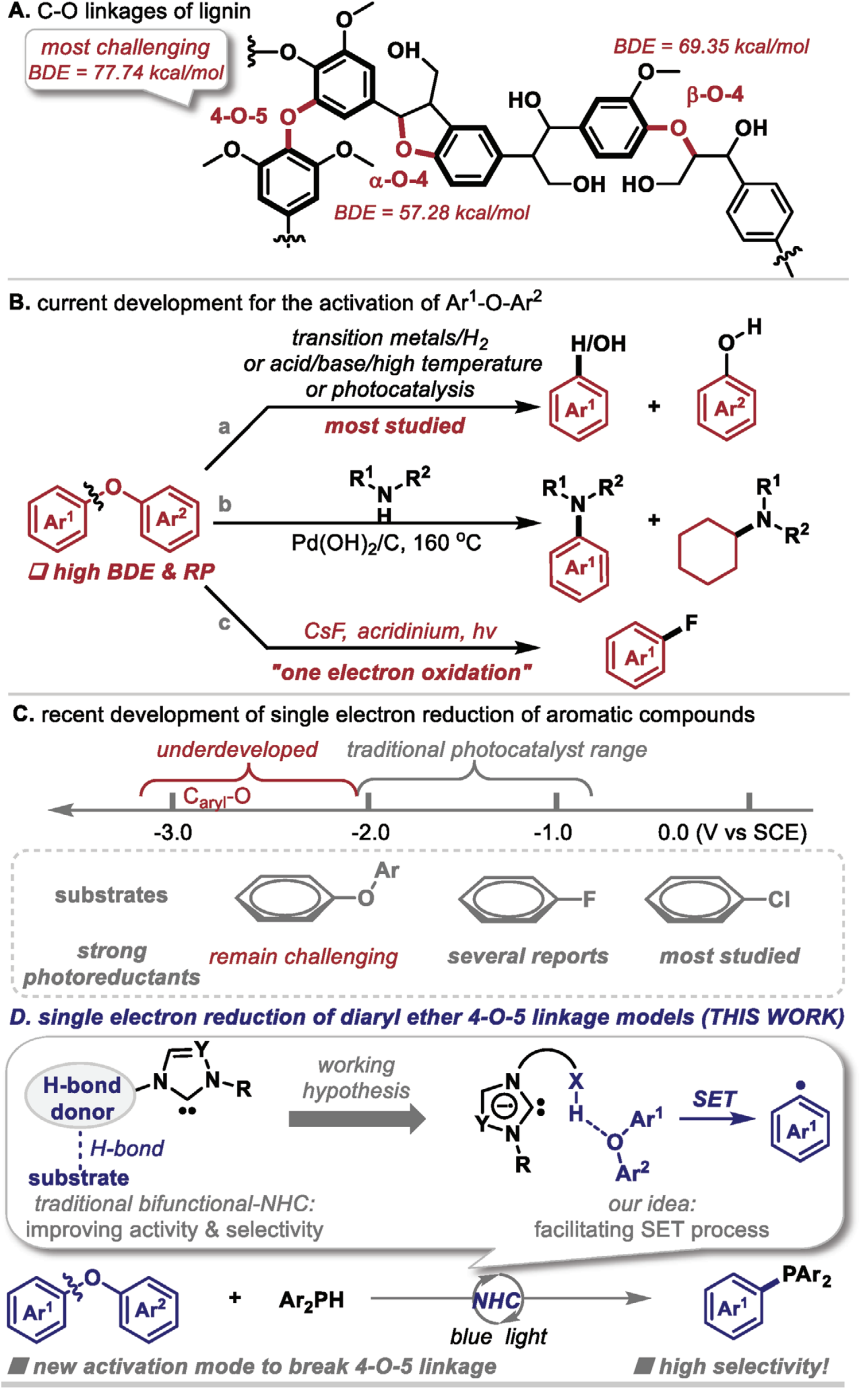
Typical catalytic modes for the activation of diaryl ethers and outline of this work: photoinduced bifunctional NHC‐catalyzed single electron reduction of diaryl ethers.

Lignin, which makes up ≈30% of Earth's organic carbon and has traditionally been regarded as waste, is now gaining attention for its potential as a source of sustainable chemicals and alternative energy sources.^[^ ^1^ ^]^ Lignin consists of aromatic monomers connected by C─C and C─O bonds, including α‐O‐4, β‐O‐4, and 4‐O‐5 linkages (**Figure** **1**A). Efficient and selective cleavage of these C─O bonds could offer a sustainable pathway for the production of valuable feedstock chemicals from lignin biomass. Among the linkages, the 4‐O‐5 bond possesses the highest bond dissociation energy (BDE = 77.74 kcal mol^−1^), compared to α‐O‐4 (BDE < 57.28 kcal mol^−1^) and β‐O‐4 (BDE < 69.35 kcal mol^−1^).^[^ ^2^ ^]^ As such, while the transformation of C─O aliphatic ether bonds (α‐O‐4 and β‐O‐4 linkages) has been well studied, activation of the diphenyl ether bond (4‐O‐5 linkage) remains one of the most challenging tasks in both industrial and academic research.^[^ ^3^ ^]^ Existing studies predominantly focus on hydrogenolysis^[^ ^4^ ^]^ and hydrolysis^[^ ^5^ ^]^ of diaryl ether C─O bonds to produce phenol and benzene at elevated temperatures (typically between 120–400 °C) (Figure 1Ba). Recently, Li and co‐workers demonstrated a visible‐light photoredox‐catalyzed C─O bond cleavage of diaryl ethers with photoredox and vanadate dual catalysis, or using acidolysis followed by one‐pot hydrolysis to produce phenols with an acridinium photocatalyst and copper Lewis acid.^[^ ^6^ ^]^ Jiang and co‐workers also reported an interesting uranyl‐photocatalyzed hydrolysis of diaryl ethers.^[^ ^7^ ^]^ Despite these advances, direct production of other value‐added chemicals from diaryl ether 4‐O‐5 linkage models remains underdeveloped. To our knowledge, only two prior examples exist in the literatures. In 2018, Li and co‐workers accomplished the cross‐coupling of diaryl ethers with amines to form C─N bonds, though the approach lacked selectivity, producing both anilines and cyclohexyl amines (Figure 1Bb).^[^ ^8^ ^]^ Nicewicz and co‐workers developed a photoinduced one‐electron oxidation strategy to cleave diaryl ether bonds and enable the deoxyfluorination of diaryl ethers using an acridinium photocatalyst (Figure 1Bc).^[^ ^9^ ^]^

On the other hand, photoinduced one‐electron reduction of organic molecules is an essential and foundational process that drives a variety of useful synthetic transformations.^[^ ^10^ ^]^ This has resulted in the development of several novel strategies, including consecutive photoinduced electron transfer, electron‐primed photoredox catalysis, excited state anion catalysis, and others,^[^ ^11^ ^]^ for the reductive activation of a variety of inert substrates.^[^ ^12^ ^]^ Despite these progresses, the photoreductive activation of diaryl ethers has remained underdeveloped due to the robust nature of the C_aryl_‐O bond (Figure 1C). Only recently has the photoreduction of diphenyl ether been realized by König and co‐workers. They presented one example of radical borylation of diphenyl ether via thiolate/B_2_pin_2_ and boryl‐anion‐activated substrate system, but showing low efficiency.^[^ ^13^ ^]^

## 2 Results and Discussion

### 2.1 Optimization of Reaction Conditions

In a recent discovery, we observed that upon exposure to blue light irradiation, the combination of N‐heterocyclic carbenes (NHCs) and *t*BuOK could yield highly reducing NHC radical anions.^[^ ^14^ ^]^ These radical anions, in turn, enable the single electron reduction of several C_aryl_‐X bonds. Thus, the model reaction of diphenyl ether **1** and diphenylphosphine **2** was investigated by using NHC **A** as the catalyst in the presence of blue light irradiation. Unfortunately, this system gave a very low yield (**Table** **1**, top). On the other hand, bifunctional NHCs possessing both a reactive nucleophilic site and a hydrogen bond donor can mimic enzyme‐like strategies for various reactions.^[^ ^15^ ^]^ In such instances, the nucleophilic activation of one substrate by NHC combined with H‐bonding to another substrate can facilitate the reaction. Beyond enhancing selectivity in established reactions, bifunctional NHCs can enable new transformations through their ability to interact via H‐bonding. Inspired by this, we envisioned that this advantage could facilitate the single‐electron reduction of diaryl ether 4‐O‐5 linkage models, as the H‐bond can bring diaryl ethers close to the reactive site of NHCs (Figure 1D). To validate our hypothesis, we prepared several bifunctional NHCs bearing an amino group, which was supposed to form H‐bond with ethers (Table 1, bottom). The structure of NHC **B** was affirmed by the X‐ray crystallographic analysis. We were delighted to find that the reaction catalyzed by the NHC **B** in the presence of tBuOK as the base afforded the desired triphenylphosphine **3** in 93% yield (entry 1). The screening of the catalysts indicated that other bifunctional NHCs **C** and **D** were also suitable catalysts (entries 2 and 3). However, when protecting the amino group, the reaction efficiency decreased dramatically (entry 4). These results indicated the important role of bifunctional NHCs for the single electron reduction of 4‐O‐5 linkage model compounds. Control experiments confirmed the necessity of the NHC catalyst, tBuOK, and visible light (entries 5–7).

**Table 1:** Reactivity comparison of normal and bifunctional NHCs.

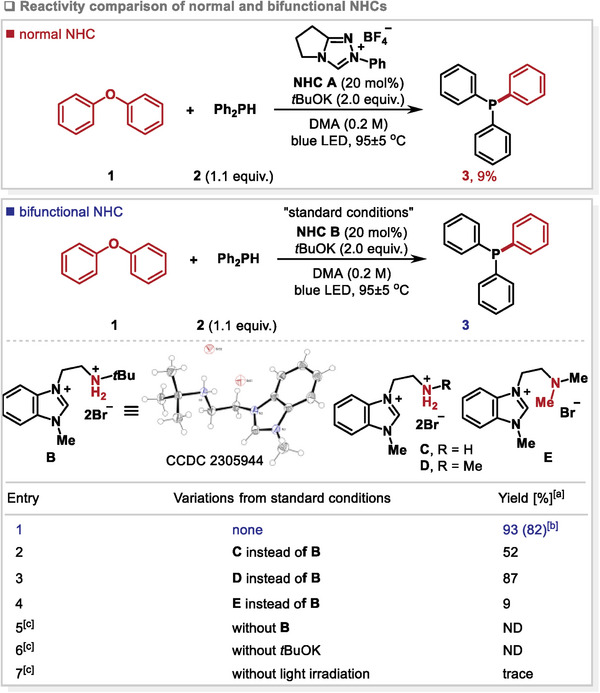

^a)^ Determined by GC‐MS using *n*‐hexadecane as the internal standard;

^b)^ yield of isolated product;

^c)^ reaction was carried out at 95 °C; DMA: *N*, *N*‐dimethylacetamide; ND: not detected.

### 2.2 Substrate Scope

**Figure 2:**
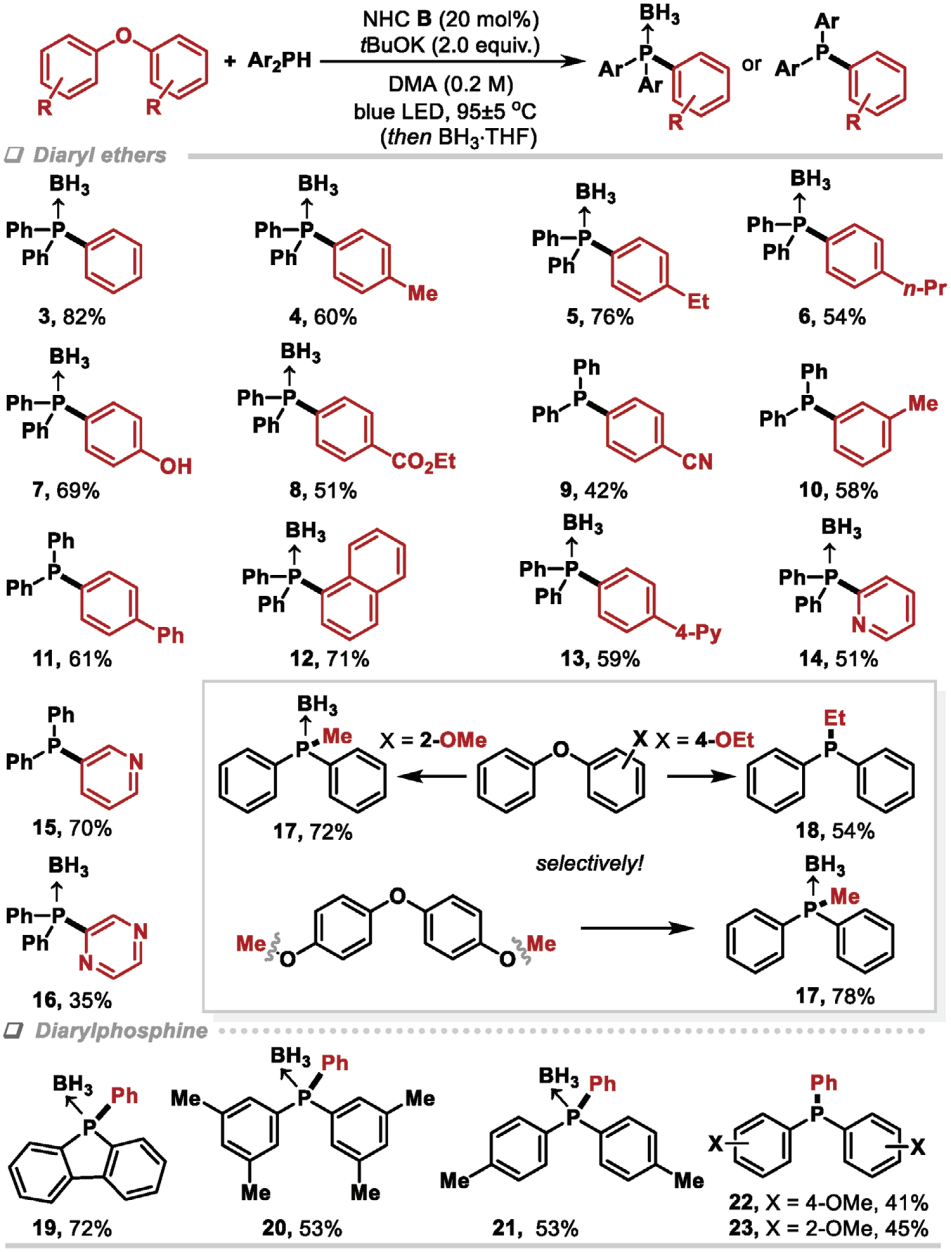
Substrate Scope. Yield of isolated products **3–23** after chromatography.

Having established the optimal conditions, we set about investigating the scope of the reaction with various diaryl ethers. As shown in **Figure** **2**, both electron‐donating (4‐Me, 4‐Et, 4‐*n*Pr and 4‐OH) and electron‐withdrawing (4‐CO_2_Et and 4‐CN) groups on the phenyl ring were tolerable and afforded the desired products **4**–**9** in moderate to good yields. The reaction of meta‐substituted substrate also proceeded smoothly (**10**). Further exploration of the reaction scope indicated phenyl, naphthyl, and pyridyl‐substituted diphenyl ethers were all effective reaction partners (**11‐**‐**13**). Pleasingly, oxybispyridines and oxybispyrazine efficiently reacted with diphenylphosphine **2**, providing the corresponding products **14**–**16** in 35–70% yields. Notably, substrates bearing both alkyl ether group and aryl ether group, such as 2‐methoxydiphenylether, 4‐ethoxydiphenylether, and 4,4′‐oxybis(methoxybenzene), selectively gave the alkyl phosphine products **17** and **18,** respectively. The preference of cleaving the alkyl C─O bond may be due to the more stable alkyl radicals than aryl radicals (see Supporting Information for details). The scope of the diarylphosphines was also briefly explored. The reaction of cyclic diarylphosphine gave the desired product **19** in 72% yield. In addition, meta, meta‐disubstituted, para‐substituted, and ortho‐substituted substrates were all readily coupled under the standard reaction conditions (**20**–**23**). Monoaromatic substituted phosphorus and diphenylphosphorus with electron‐withdrawing group, such as methylphenylphosphine, bis(4‐fluorophenyl)phosphine, and bis(4‐(trifluoromethyl)phenyl)phosphine, were also examined, but these substrates showed incompatibility in the reaction system.

**Figure 3:**
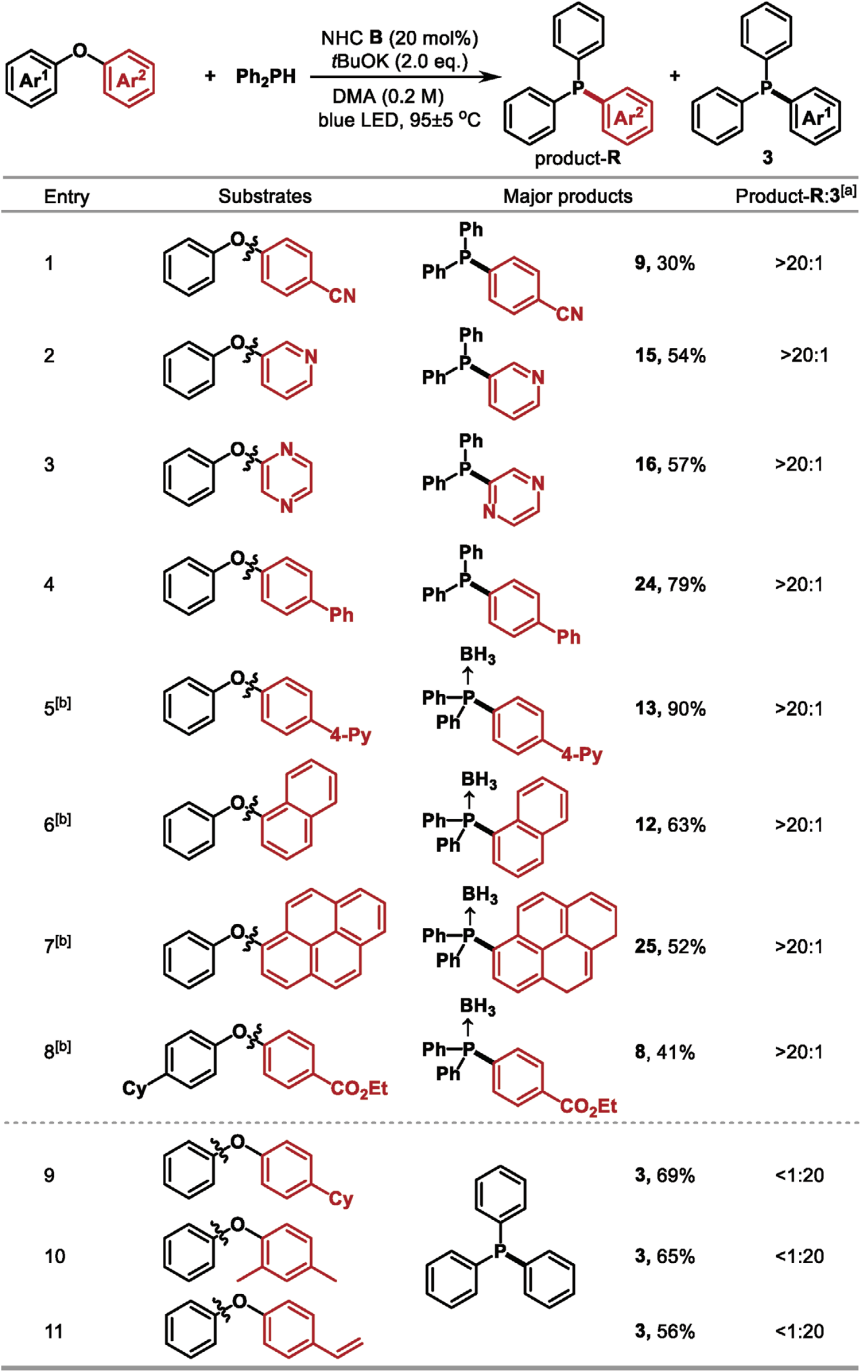
Substrate scope of unsymmetric diaryl ethers. Yield of isolated products after chromatography; a) the ratios were determined by GCMS analysis; b) BH_3_•THF was added.

To further study the generality of the developed strategy, we employed unsymmetric diaryl ethers as coupling partners. As presented in **Figure** **3**, the results revealed that the current strategy showed good selectivity and reaction efficiency. It resulted in the selective cleavage of electron‐deficient aryl groups, rather than electron‐donating aryl groups. Specifically, 4‐phenoxybenzonitrile, pyridinyl phenyl ethers, pyrazinyl phenyl ether, biphenyl phenyl ethers, naphthyl phenyl ethers, and pyrenyl phenyl ethers were all successfully converted into the corresponding triaryl phosphines (entries 1–7). In addition, diaryl ether substrate, bearing an electron‐donating (Cy) and an electron‐withdrawing group (CO_2_Et) on the individual phenyl ring, was also tolerant to selectively deliver the electron‐deficient aryl group substituted triaryl phosphine (entry 8). While the diaryl ethers with electron‐donating groups, triphenylphosphine **3** was obtained as the major product (entries 9–11).

**Figure 4:**
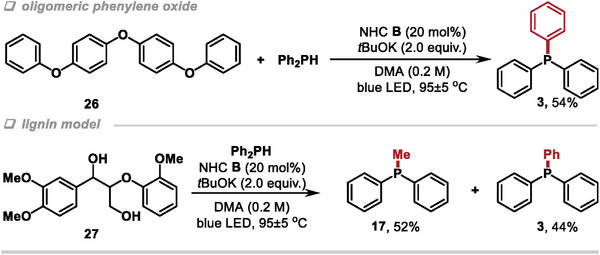
Phosphorylation of oligomeric phenylene oxide and lignin model.

Having identified this effective method for the cleavage of simple diaryl ethers, we tested the ability of this system to achieve the phosphorylation of oligomeric aryl ether. Photoreduction of the oligomeric phenylene oxide containing six aromatic C─O bonds (**26**) resulted in the corresponding triphenylphosphine **3** in 54% yield under the standard conditions (**Figure** **4**, top). To demonstrate the potential application of this methodology, a molecule closer related to lignin was also examined in our reaction system. The cleavage of C─O bonds of β‐O‐4 model **27**, bearing three electron‐donating substituents and two hydroxyl groups for approaching the exact structure in lignin, was realized and provided the corresponding triphenylphosphine **3** and diphenylmethyl phosphine **17** in 44 and 52% yields, respectively (Figure 4, bottom) (see Supporting Information for details of the possible reaction pathway).

### 2.3 Mechanistic Studies

**Figure 5:**
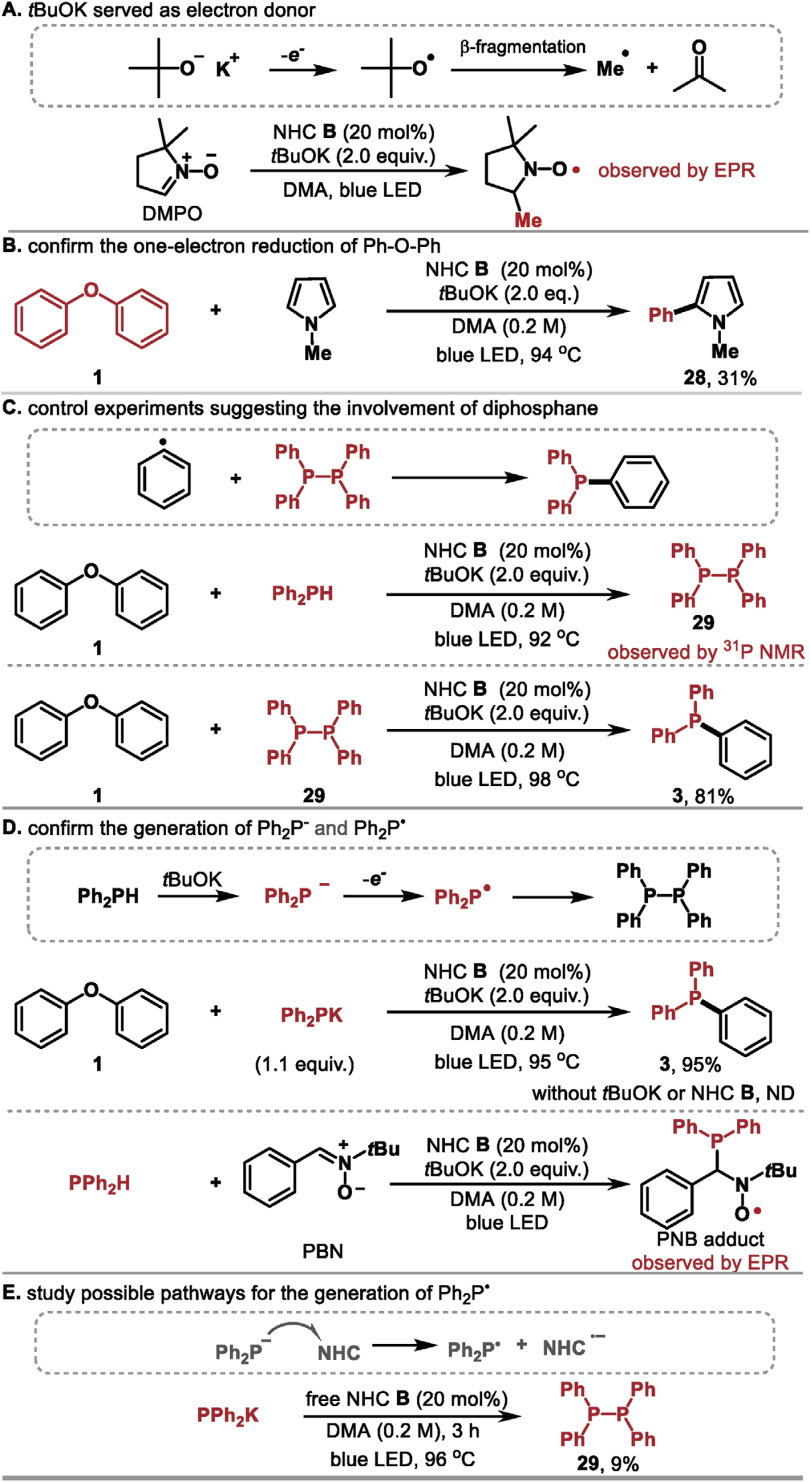
Mechanistic studies.

To understand the reaction mechanism, a series of mechanistic experiments were performed (**Figure** **5**). The EPR experimental results in Figure 5A indicated that NHC **B** and tBuOK could form a photoactive charge transfer complex with tBuOK as an electron donor, in agreement with known reports. ^[^ ^14^, ^16^ ^]^ The isolation of the radical trapping adduct (**28**) suggested that diphenyl ether underwent one‐electron reduction to afford the phenyl radical (Figure 5B). Additionally, we observed 1,1,2,2‐tetraphenyldiphosphane **29** during the reaction (see Supporting Information for details) (Figure 5C, top). The reaction of **29** with diphosphane **1** gave the desired product **3** (Figure 5C, bottom), indicating that the C_aryl_‐P bond could be formed via the reaction of the diphosphane with the phenyl radical. According to previous studies,^[^ ^17^ ^]^ the diphosphane could be produced through the homocoupling of the Ph_2_P• radical. We hypothesized that Ph_2_PH was initially converted to the Ph_2_P^−^ anion in the presence of tBuOK, followed by one‐electron oxidation to form the Ph_2_P• radical. The ^31^P NMR experiments of the reaction mixture showed that Ph_2_PH was completely converted to Ph_2_P^−^ in 10 min (see Supporting Information for details). Supportively, replacing Ph_2_PH with Ph_2_PK as the coupling partner resulted in the desired product with a yield comparable to the reaction with Ph_2_PH as the substrate (Figure 5D, top), and a spin‐trapped Ph_2_P• radical adduct was obtained when Ph_2_PH was subjected to the standard reaction conditions (Figure 5D, bottom). Finally, the diphosphane **29** was detected from the irradiation of the mixture of Ph_2_PK and free NHC **B**, indicating that the Ph_2_P^−^ anion could act as an electron donor to reduce NHC, generating Ph_2_P• and NHC^·−^ (Figure 5E).

**Figure 6:**
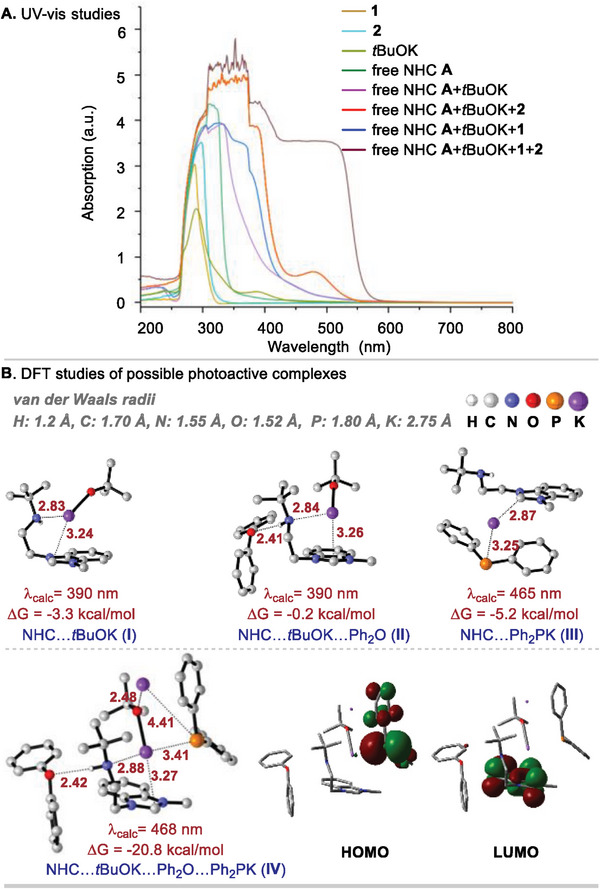
Studies of possible photoactive complexes.

Based on the findings presented in Figure 5, both tBuOK and Ph_2_PK exhibit the ability to act as electron donors, facilitating the reduction of NHC. Consequently, we aimed to explore potential photoactive complexes involving NHC. UV–vis experiments confirmed the formation of a photoactive complex between NHC and tBuOK, the addition of Ph_2_PH and diphenyl ether further enhanced the absorption (**Figure** **6**A). Given these results, we envisioned that the complexes such as NHC···tBuOK (**I**), Ph_2_O···NHC···tBuOK (**II**), NHC···Ph_2_PK (**III**), and Ph_2_O···NHC···tBuOK···Ph_2_PK (**IV**) could play a role in the reaction. To gain further insight into these complexes, we conducted density functional theory (DFT) and time‐dependent DFT (TDDFT) calculations (see Supporting Information for the details). As depicted in Figure 6B, the calculations revealed that the complex NHC···Ph_2_PK (**III**, 465 nm) and Ph_2_O···NHC···tBuOK···Ph_2_PK (**IV**, 468 nm) exhibited absorption wavelengths longer than those of NHC···tBuOK (**I**, 390 nm) and Ph_2_O···NHC···tBuOK (**II**, 390 nm). The computed wavelengths are in reasonable agreement with the red‐shift feature of UV–vis spectra. Notably, the absorption of **III** and **IV** primarily arises from the electron transfer from Ph_2_P^−^ (see HOMO) to the NHC (see LUMO), which is different from the cases of **I**, **II**, where the electron transfer takes place from tBuOK to NHC. According to that the mixture of NHC **B**, tBuOK, **1**, and **2** has the strongest absorption in the visible light region and Ph_2_O···NHC···tBuOK···Ph_2_PK (**IV**) has the largest complexation free energy, we propose the complex could play a more important role than the others (**I**)–(**III**).

**Figure 7:**
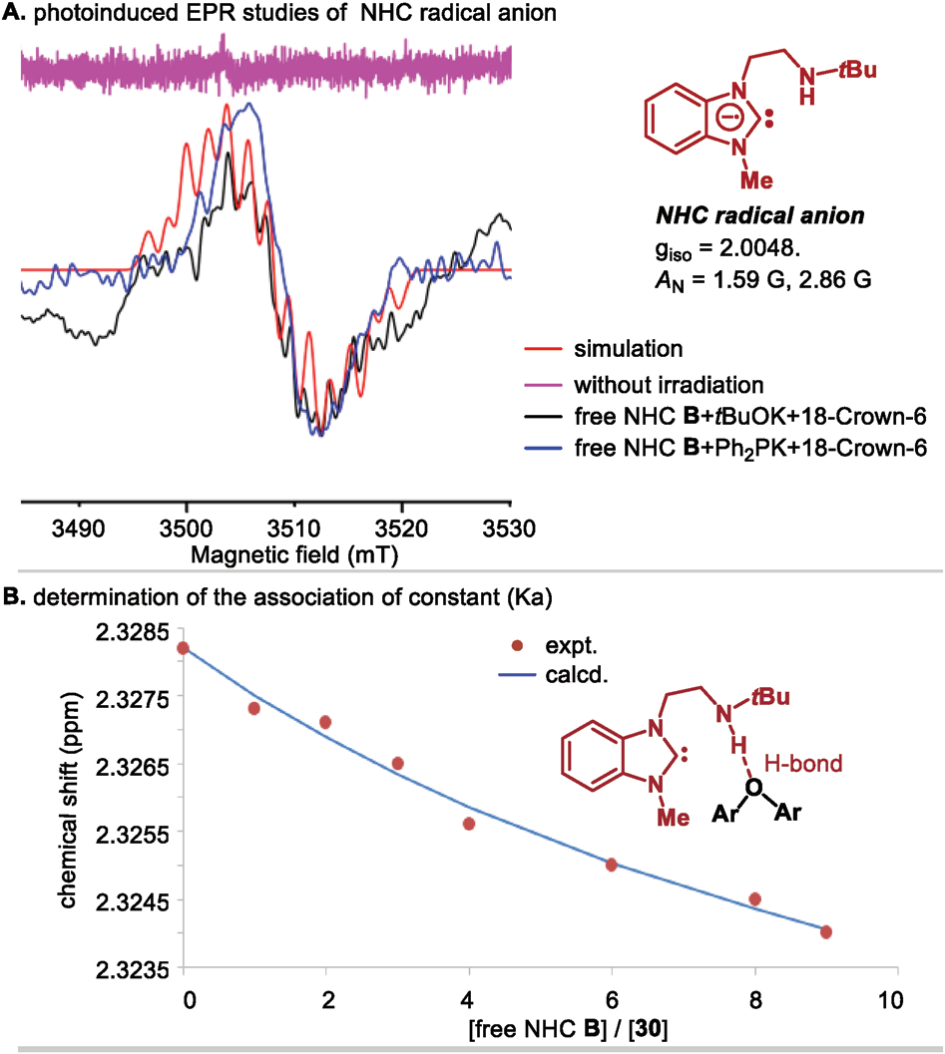
A) photoinduced EPR studies; simulated with g_iso_ = 2.0048, *A*_N_ = 1.59 G, 2.86 G; B) the curve fitting of the ^1^H NMR titration data by Bindfit program.

To further confirm the generation of the NHC radical anion in our catalytic system, we conducted EPR studies. We monitored a solution containing tBuOK and free NHC **B** using EPR spectroscopy. In the absence of light irradiation, no signal was detected. However, when exposed to blue light irradiation, the NHC radical anion became evident. The EPR spectrum exhibited a resonance with g = 2.0048 and displayed a hyperfine splitting pattern indicative of couplings with two nitrogen atoms (1.59 and 2.86 G). This was also true for the mixture of free NHC **B** and Ph_2_PK (**Figure** **7**A). Furthermore, the weak H‐bond interaction between bifunctional NHC **B** and m‐tolyl ether (**30**) was corroborated by a ^1^H NMR titration. The association constant (Ka) between them was calculated to be 7.94 ± 0.83 m ^−1^ (Figure 7B). These findings suggest that the amino group of NHC enables the binding of diaryl ether, which in turn facilitates the SET process.

**Figure 8:**
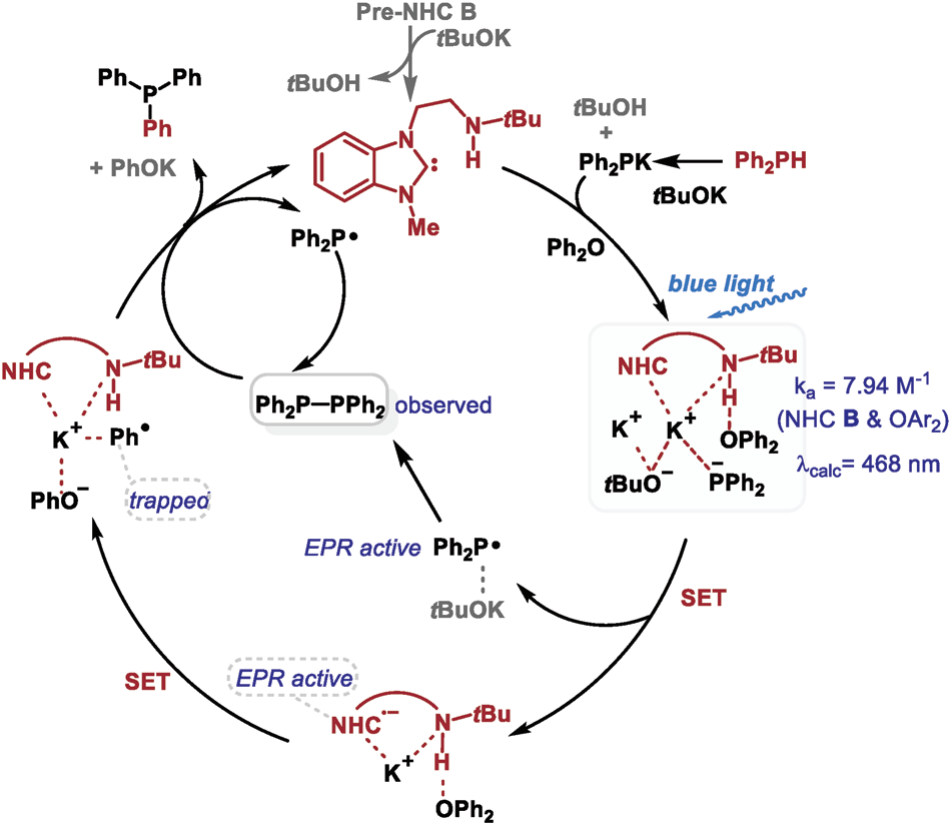
Proposed mechanism.

Based on the above results and our previous report,^[^ ^14^ ^]^ we propose a possible major pathway through the complex **IV** (**Figure** **8**). The in situ generated free NHC first forms the photoactive complex **IV** with **1**, Ph_2_PK, and tBuOK. The complex then undergoes a SET process upon blue light irradiation, where the Ph_2_P^−^ donates an electron to NHC. The SET process results in the NHC radical anion and Ph_2_P• radical. The Ph_2_P• is then involved in the formation of 1,1,2,2‐tetraphenyldiphosphane, while the NHC radical anion reduces diphenyl ether **2** to a phenyl radical. In the subsequent step, the phenyl radical reacts with 1,1,2,2‐tetraphenyldiphosphane, yielding the desired product PPh_3_ and releasing NHC to participate in the next cycle and Ph_2_P• for the formation of 1,1,2,2‐tetraphenyldiphosphane. We also considered an alternative pathway involving consecutive photoinduced electron transfer, however, our studies do not support this mechanism (see Supporting Information for the details).

## 3 Conclusion

In conclusion, we have developed a new bifunctional amino‐NHC that can cleave the 4‐O‐5 linkage in lignin model compounds. Under visible light irradiation, this catalyst enables the selective one‐electron reduction of diaryl ethers, facilitating the synthesis of trivalent phosphines. Mechanistic studies indicated that the reaction involves a reducing NHC radical anion intermediate and the H‐bond between the NH group of NHC and the diaryl ether plays a pivotal role to enable the single electron transfer from the NHC radical anion to the diaryl ether for its reduction. Moreover, this method also carried out the selective one‐electron reduction of unsymmetric diaryl ethers, oligomeric phenylene oxide, and lignin model. The method is characterized by simple reaction setup, high selectivity, and making use of easily accessible bifunctional NHC. We anticipate that the presented strategy could find applications in the degradation of lignin and inspire new applications in the photoreduction of compounds with high reduction potentials.

## Conflict of Interest

The authors declare no conflict of interest.

## Supporting information

Supporting Information

## Data Availability

The data that support the findings of this study are available in the supplementary material of this article.
