# Supplementary material for: Photoinduced Single Electron Reduction of the 4‐O‐5 Linkage in Lignin Models for C‐P Coupling Catalyzed by Bifunctional N‐Heterocyclic Carbenes
Source: Adv Sci (Weinh). 2024 Aug 5;11(38):2406095. doi: 10.1002/advs.202406095 (PMC11481192; doi:10.1002/advs.202406095)
Supplement: Supplementary file 1 — Supporting Information [file ADVS-11-2406095-s001.pdf]

ADVANCED SCIENCE
Open Access

Supporting Information

for *Adv. Sci.*, DOI 10.1002/advs.202406095

Photoinduced Single Electron Reduction of the 4-O-5 Linkage in Lignin Models for C-P Coupling Catalyzed by Bifunctional N-Heterocyclic Carbenes

Qiang Liu, Ying-Zheng Ren, Bei-Bei Zhang, Wen-Xin Tang, Zhi-Xiang Wang, Lin He* and Xiang-Yu Chen**

Supporting Information

Photoinduced Single Electron Reduction of the 4-O-5 Linkage in Lignin Models for C-P Coupling Catalyzed by Bifunctional N-Heterocyclic Carbenes

Qiang Liu[a+], Ying-Zheng Ren[a,c+], Bei-Bei Zhang[a+], Wen-Xin Tang[a], Zhi-Xiang Wang*[a,b], Lin He*[c], and Xiang-Yu Chen*[a,b]

[a] Dr. Q. Liu, Y.-Z. Ren, Dr. B.-B. Zhang, Prof. Dr. Z.-X. Wang, Prof. Dr. X.-Y. Chen
School of Chemical Sciences, University of the Chinese Academy of Sciences, Beijing National Laboratory for Molecular Sciences, Beijing 100049 China
E-mail: zxwang@ucas.ac.cn, chenxiangyu20@ucas.ac.cn
[b] Binzhou Institute of Technology, Weiqiao-UCAS Science and Technology Park, Binzhou, Shandong Province, 256606 China
[c] Y.-Z. Ren, Prof. Dr. L. He
State Key Laboratory Incubation Base for Green Processing of Chemical Engineering, School of Chemistry and Chemical Engineering, Shihezi University, Xinjiang 832000, China
E-mail: helin@shzu.edu.cn
[+] These authors contributed equally to this work.

Table of Contents

1. General Information S3
2. Experimental procedures S5
   2.1. Investigation of reaction conditions S5
   2.2. General procedure for the synthesis of trivalent phosphines S6
   2.3. General procedure for the synthesis of NHC salts S6
   2.4. Procedures for the phosphorylation of oligomeric phenylene oxide and lignin model S7
3. Mechanism studies S8
   3.1. UV/vis studies S8
   3.2. Confirm the one-electron reduction of diphenyl ether S9
   3.3. ^{31}P NMR experiments suggested the involvement of diphosphane and Ph_2P^- S9
   3.4. Confirm the generation of Ph_2P^- S11
   3.5. Study possible pathways for the generation of $Ph_2P^•$ S11
   3.6. EPR spectroscopy experiments S12
   3.7. Quantum yield determination S14
   3.8. Light on-off experiment S15
   3.9. Determination of the binding ratio by Job's plot S15
   3.10. ^{1}H NMR titrations of *m*-tolyl ether with free NHC **B** S16
   3.11. The dependence of the reaction yield on the excitation power S18
4. Transformations of boron-coordinated phosphorus compounds S18
5. Compound characterization data S21
6. Computational details S30
7. NMR spectra S40
8. Single crystal X-ray diffraction of NHC-**B** S107
9. References S109

1. General Information

- Chemicals were purchased from Heowns, Innochem and Bidepharm, and they were used without further purification unless otherwise noted. Diaryl ethers were prepared according to the literature methods.[1] Solvents were purified using a solvent-purification system (VSPS-8, Vigor).
- Chromatographic purification of the products was performed on 200-300 mesh silica gel.
- IR spectra were taken on a Vertex 70 spectrophotometer and reported as wave numbers (cm^{-1}).
- UV-vis absorption spectra were acquired on UV-1900i spectrophotometer (Shimadzu, Japan).
- The GC-MS TQ8040 was used in the detection of the reaction mixture.
- The SGW X-4 was used to measure the melting point of solids.
- The reaction temperature was monitored by using the IR Thermometer (TA601B).
- HRMS (ESI) were obtained with the Thermo Scientific LTQ Orbitrap XL mass spectrometer.
- ^{1}H-, ^{19}F-, ^{31}P-, ^{11}B- and ^{13}C- NMR spectra were recorded at ambient temperature on a Shimadzu Avance 400 Spectrometer, Shimadzu Avance 500 Spectrometer, and Shimadzu Avance 600 Spectrometer. The chemical shifts are reported in ppm downfield of tetramethylsilane (TMS) and referenced to residual solvent peaks resonance as the internal standard. The order of citation in parentheses is a) multiplicity (s = singlet, d = doublet, t = triplet, q = quartet, dd = doublet of doublet, m = multiplet), b) coupling constants, c) number of protons. Coupling constants (*J*) are reported in Hertz (Hz).
- Photochemical experiments were performed magnetically stirred in 10 mL glass tubes, sealed with a rubber septum. The tubes were irradiated with blue LED lamps (Kelo-AO100S or PLS-LED100C). The distance from the light source to the irradiation vessel was 0.2 cm and a fan was used to keep the reaction temperature at 95±5 °C. (The purchase link of Kelo-AO100S blue LED is https://item.taobao.com/item.htm?spm=a1z10.5-c-s.w4002-21207510047.14.dbef5298YBVk03&id=522759747619. The purchase link of PLS-LED100C blue LED (460 nm) is https://www.perfectlight.cn/Product/detail/id/32.html.).

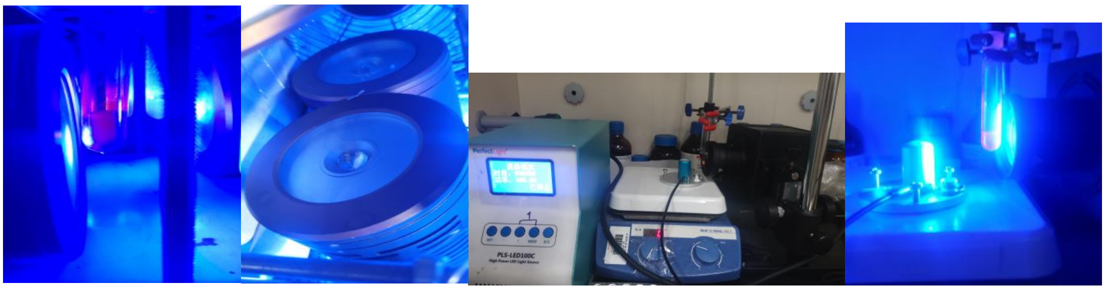

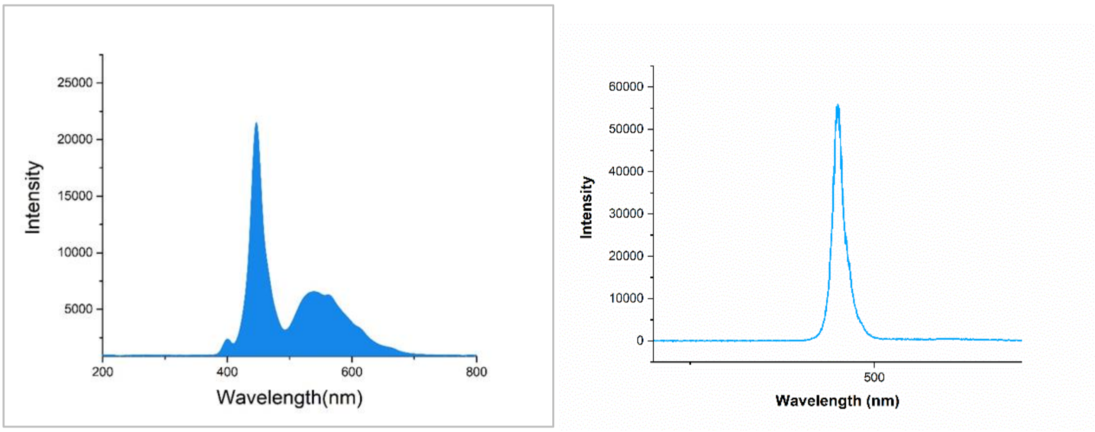

Figure S1. The spectra of blue LEDs employed in the reaction (left: Kelo-AO100S, right: PLS-LED100C).

2. Experimental procedures

2.1. Investigation of reaction conditions

Table S1. Optimization of the reaction conditions

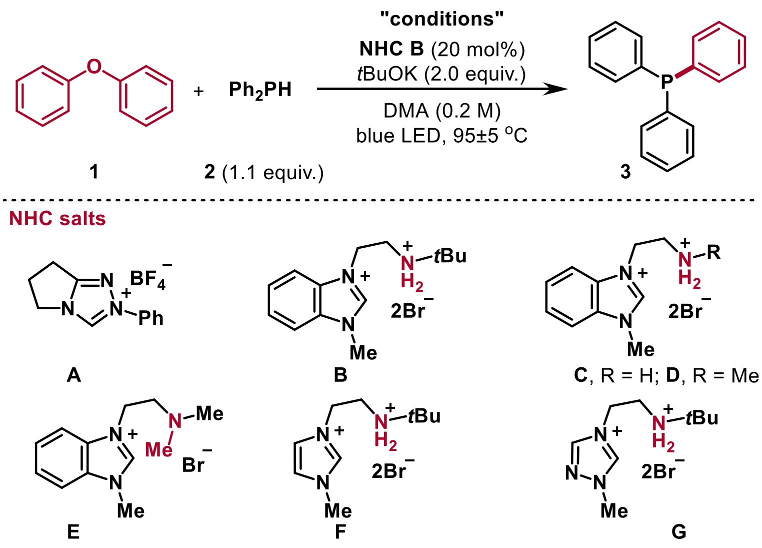

Entry	Variations from standard conditions	Yield (%)[a]
1	none	93 (82)[b]
2	KOH instead of *t*BuOK	14
3	TMEDA instead of *t*BuOK	ND
4	DBU instead of *t*BuOK	2
5	*t*BuONa instead of *t*BuOK	8
6	*t*BuOLi instead of *t*BuOK	trace
7	**A** instead of **B**	9
8	**C** instead of **B**	52
9	**D** instead of **B**	87
10	**E** instead of **B**	9
11	**F** instead of **B**	22
12	**G** instead of **B**	12
13	**free B** instead of **B**	87
14	DMSO instead of DMA	trace
15	DMF instead of DMA	51
16	DCM instead of DMF	trace
17	*t*BuOK (1.0 equiv.)	6
18	55 °C instead of 95 °C	30
19	Without NHC **B**	ND
20	without *t*BuOK	ND

21	without visible-light irradiation	trace

[a] Determined by GC-MS using *n*-hexadecane as the internal standard. [b] Yield of the isolated product after chromatography.

2.2. General procedure for the synthesis of trivalent phosphines

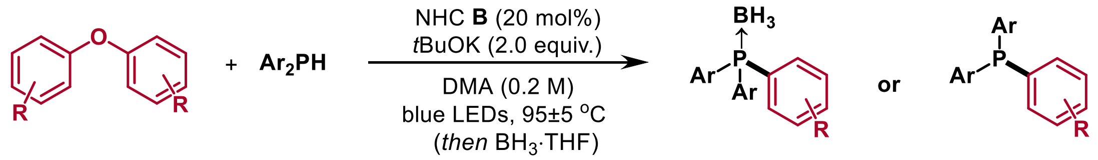

General procedure I: In a nitrogen-filled glovebox, to a dry tube equipped with a stirring bar, the diaryl ether (0.2 mmol, 1.0 equiv.), Ar_2PH (0.22 mmol, 1.1 equiv.), *t*BuOK (44 mg, 0.4 mmol, 2.0 equiv), NHC **B** (15.6 mg, 0.04 mmol, 0.2 equiv.), and DMA (1.0 mL) were added. The resulting mixture was allowed to stir at 95 ± 5 °C under blue LED (100 W) irradiation for 12 hours. Then borane–tetrahydrofuran complex (2 mL, 1 M solution in THF) was added to the reaction mixture under nitrogen at 0 °C. The mixture was stirred for 2 hours at rt. The solvent was removed under vacuum and the residue was subjected to silica gel chromatography using petroleum ether and ethyl acetate as eluent to afford the desired product.

Scale-up reaction:

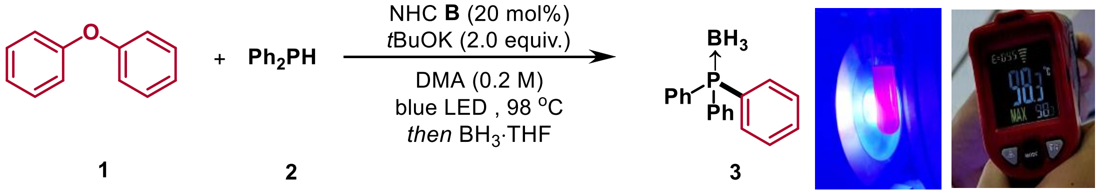

In a nitrogen-filled glovebox, to a dry tube equipped with a stirring bar, the diphenyl ether **1** (1.0 mmol, 1.0 equiv.), Ph_2PH (1.1 mmol, 1.1 equiv.), *t*BuOK (220 mg, 2.2 mmol, 2.0 equiv.), NHC (78 mg, 0.2 mmol, 0.2 equiv.), and DMA (5.0 mL) were added, the mixture was stirred under a 100 W blue LED lamp with an interval of 0.2 cm from the lamp, and a fan was used to keep the reaction temperature at 98 °C. After 24 hours, the reaction mixture was transferred to a 25 mL reaction vessel and borane–tetrahydrofuran complex (10 mL, 1 M solution in THF) was added to the reaction mixture under nitrogen at 0 °C. The mixture was stirred for 4 hours at rt. Then the solvent was removed under vacuum and the residue was subjected to silica gel chromatography using petroleum ether as eluent to afford the desired product **3** with 84% yield.

2.3. General procedure for the synthesis of NHC salts

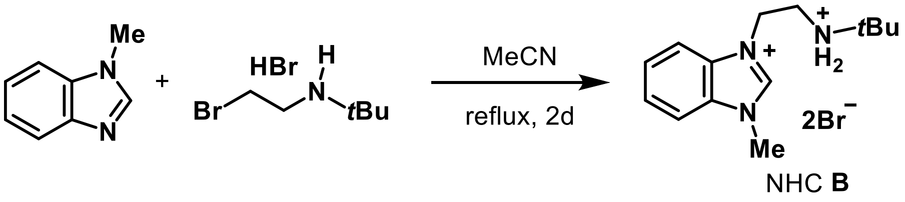

General procedure II: *t*BuNH$_2$CH$_2$-CH$_2$Br•Br (10 mmol, 1.0 equiv.) and 1-methylbenziMidazol (12 mmol, 1.2 equiv.) were dissolved in acetonitrile (80 mL) in a 200 mL flask. The resulting mixture was then stirred at 90 °C for 2 days. After cooling to room temperature, the resulting mixture was filtered and washed with EA. The collected solid was dried under vacuum to give the pure desired product NHC **B**. NHC salts **C**, **D, E, F,** and **G** were prepared according to the similar procedure.

Preparation of free NHC-**B**

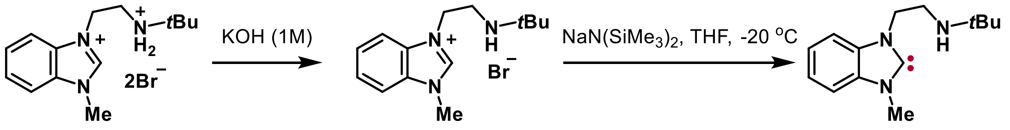

According to known report,[2] to a solution of 1.0 M potassium hydroxide (1.0 equiv.) was added compound NHC **B** (1.56 g, 1.0 equiv. 4.0 mmol). The mixture was stirred at room temperature for 8 h. The resulting solution was extracted with CH_2Cl_2, and the organic layer was dried over $MgSO_4$. The solvent was removed in vacuo to afford a white residue, which was dissolved in 50 mL of acetone/CH_2Cl_2 (50:50 by volume) and cooled to -10 °C to afford NHC **B1** as a white solid. In a nitrogen-filled glovebox, to the suspension of NHC **B1** (2.0 mmol) in 8 mL of THF was added a THF solution of NaN(TMS)$_2$ (2 mol/L) (2.0 mmol, 1 mL) at -20 °C. After stirring for 10 min, the insoluble salt was removed from the reaction mixture by filtering through Celite to afford a light yellowish solution. The volatiles were removed under vacuum in the glovebox, and the generated free NHC **B** was directly used to carry out experiments.

2.4. Procedures for the phosphorylation of oligomeric phenylene oxide and lignin model

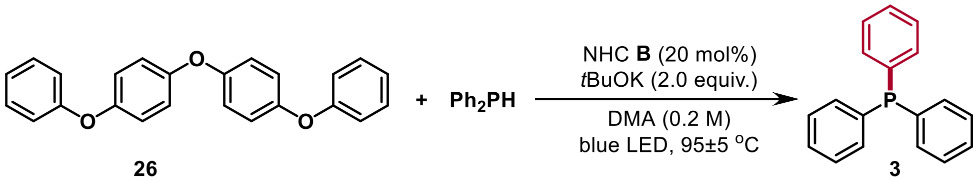

26 **3**

In a nitrogen-filled glovebox, to a dry tube equipped with a stirring bar, the oligomeric phenylene oxide (0.2 mmol, 1.0 equiv.), Ph$_2$PH (0.22 mmol, 1.1 equiv.), *t*BuOK (44 mg, 0.4 mmol, 2.0 equiv.), NHC **B** (15.6 mg, 0.04 mmol, 0.2 equiv.), and DMA (1.0 mL) were added. The resulting mixture was allowed to stir at 95 ± 5 °C under blue LED (100 W) irradiation for 12 hours. The solvent was removed under vacuum and the residue was subjected to silica gel chromatography using petroleum ether as eluent to afford the desired triphenylphosphine **3** with 54%.

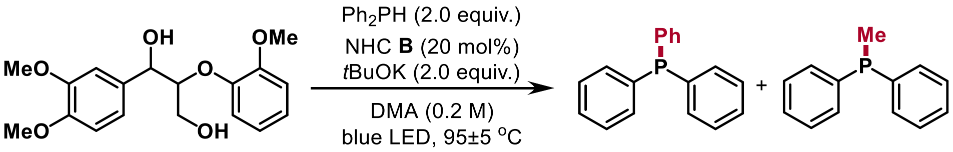

27 **3**, 44% **17**, 52%

In a nitrogen-filled glovebox, to a dry tube equipped with a stirring bar, the oligomeric phenylene oxide (0.2 mmol, 1.0 equiv.), Ph_2PH (0.40 mmol, 2.0 equiv.), *t*BuOK (44 mg, 0.4 mmol, 2.0 equiv.), NHC **B** (15.6 mg, 0.04 mmol, 0.2 equiv.), and DMA (1.0 mL) were added. The resulting mixture was allowed to stir at 95 ± 5 ^{o}C under blue LED (100 W) irradiation for 12 hours. The solvent was removed under vacuum and the residue was subjected to silica gel chromatography using petroleum ether as eluent to afford the desired triphenylphosphine **3** with 44% yield and diphenylmethylphosphine **17** with 52% yield.

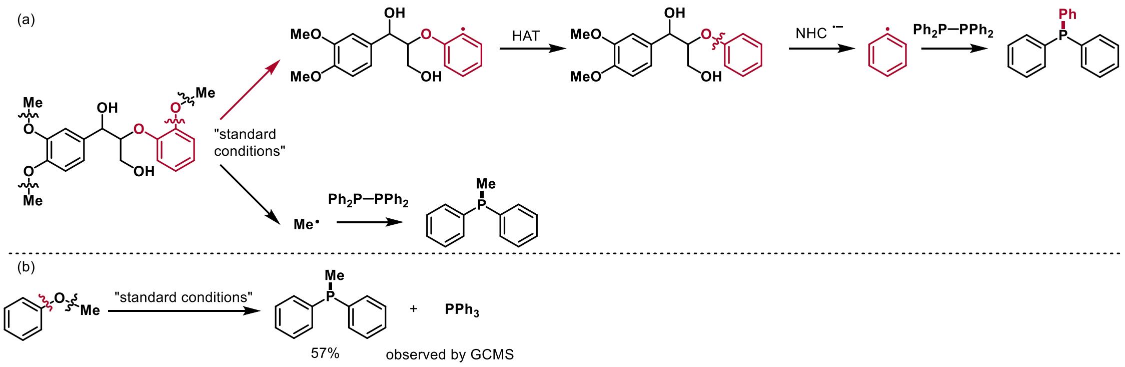

Figure S2. Possible reaction pathway of β-O-4 model compound **27**.

The reaction of the lignin model compound **27** resulted in the formation of triphenylphosphine (**17**) and diphenylmethylphosphine (**3**) with yields of 52% and 44%, respectively. Our studies indicate that both the Me-O and Ar-O bonds can undergo cleavage under the reaction conditions. Based on this observation, we propose the following pathways for the formation of products **17** and **3.** The lack of selectivity in the reaction can be attributed to the unique structural features of compound **27**, which may facilitate the cleavage of both bond types (Figure S2a).

Further experimentation with anisole supported our hypothesis, demonstrating that both the Me-O and Ar-O bonds can indeed be cleaved. This was evidenced by the detection of both diphenylmethylphosphine and triphenylphosphine (Figure S2b).

3. Mechanism studies

3.1. UV/vis studies

UV/vis absorption spectra were recorded using DMA as the solvent in 1 cm path quartz cuvettes using a UV-1900i UV/vis spectrometer.

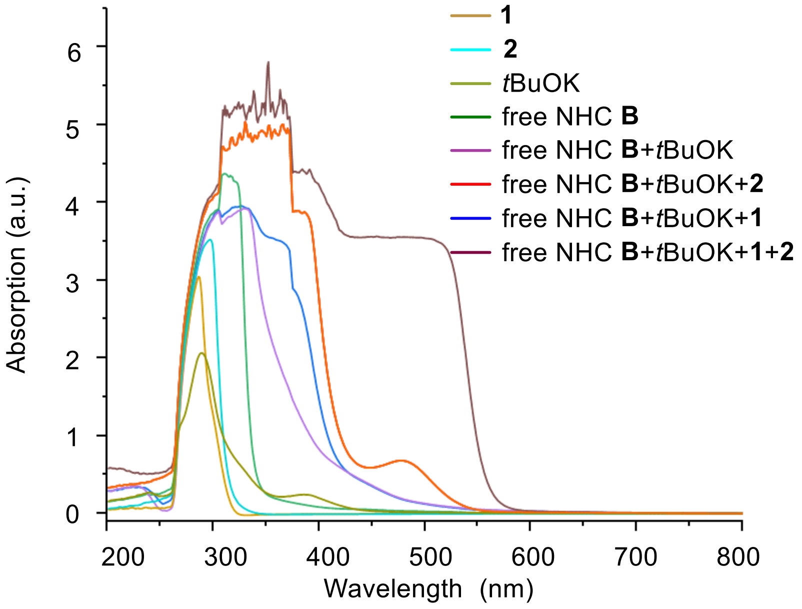

Figure S3. Absolute UV-vis absorption spectra in DMA. UV/vis spectra of free NHC **B** ($4*10^{-2}$ M), Ph_2O ($2*10^{-1}$ M), Ph_2PH ($2.2*10^{-1}$ M), *t*BuOK ($4*10^{-1}$ M), and their mixtures in DMA.

3.2. Confirm the one-electron reduction of diphenyl ether

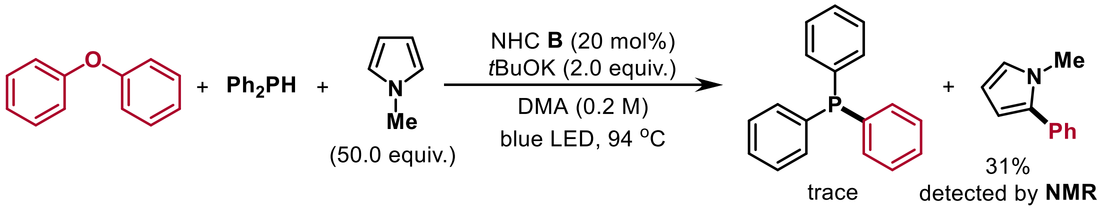

In a nitrogen-filled glovebox, to a dry tube equipped with a stirring bar, the diphenyl ether **1** (0.2 mmol, 1.0 equiv.), Ph_2PH (0.22 mmol, 1.1 equiv.), 1-methyl-1H-pyrrole (50 mmol, 50.0 equiv.), *t*BuOK (44 mg, 0.4 mmol, 2.0 equiv.), NHC **B** (15.6 mg, 0.04 mmol, 0.2 equiv.), and DMA (1.0 mL) were added. The resulting mixture was allowed to stir at 94 °C under blue LED irradiation for 12 hours. A trace amount of the desired product was observed and the trapped radical species **28** was detected with 31% yield by ^{1}H NMR spectra analysis using 1,1,2,2-tetrachloroethane as an internal standard.

3.3. ^{31}P NMR experiments suggested the involvement of diphosphane and Ph_2P^-

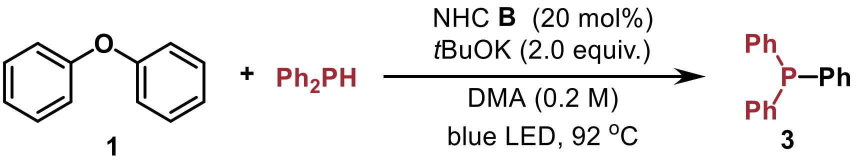

In a nitrogen-filled glovebox, to a dry tube equipped with a stirring bar, the diphenyl ether **1** (0.2 mmol, 1.0 equiv.), Ph_2PH (0.22 mmol, 1.1 equiv.), *t*BuOK (44 mg, 0.4 mmol, 2.0 equiv.), NHC **B** (15.6 mg, 0.04 mmol, 0.2 equiv.), and DMA (1.0 mL) were added. The resulting mixture was allowed to stir at 92 °C under blue LED irradiation. When the reaction was 10 min, 30 min, 1.5 h, 2.5 h, 3.5 h, 4.5 h, 5.5 h, 7.5 h, the ^{31}P NMR of the reaction mixture were measured. The ^{31}P NMR spectra indicated that Ph_2PH was completely converted to Ph_2P^- in 10 min and the amount of

Ph_2P^- decreased with the increase of reaction time. In addition, the Ph_2PPPh_2, generated from $Ph_2P\cdot$, could be observed during the reaction time.

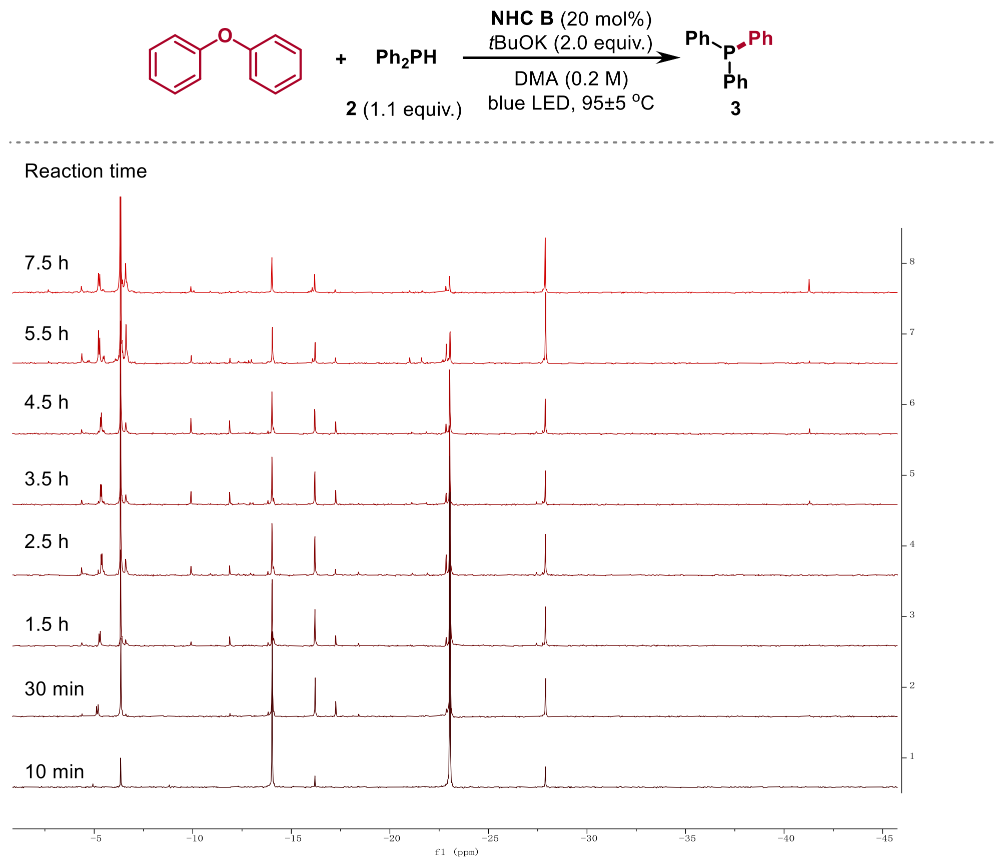

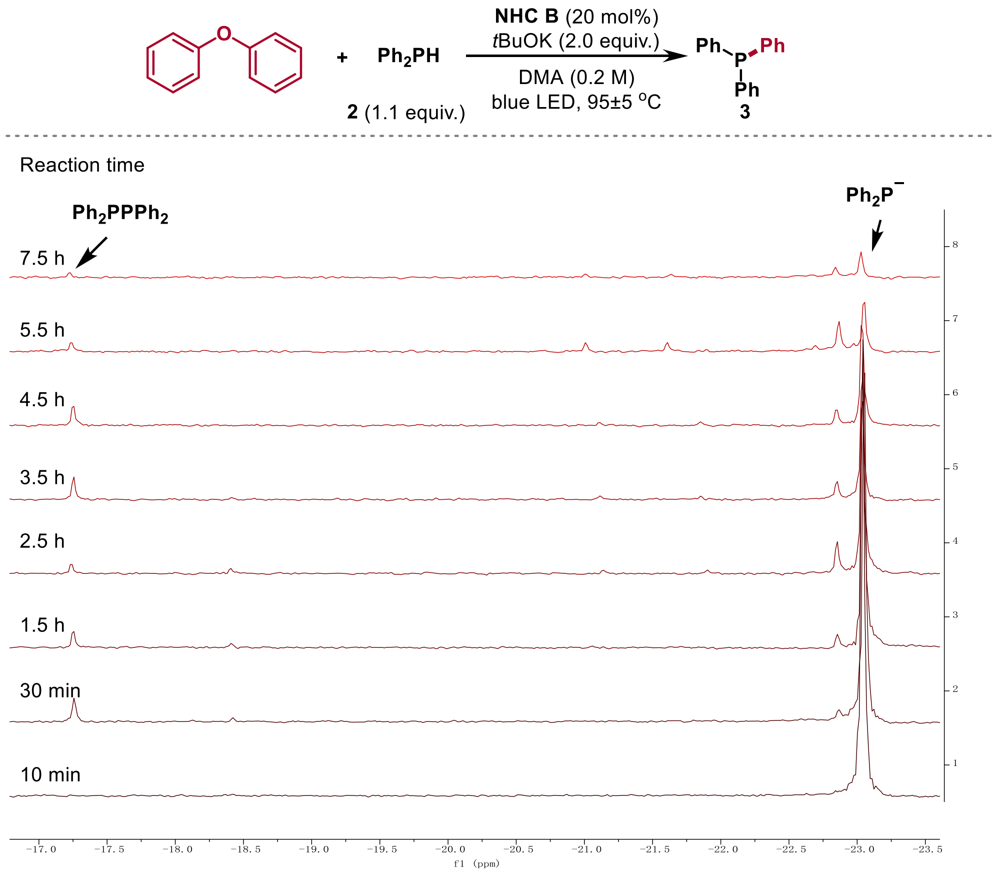

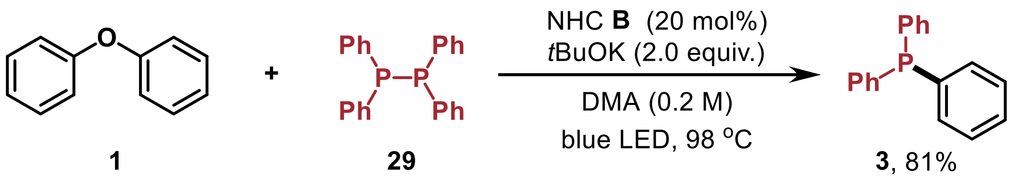

In a nitrogen-filled glovebox, to a dry tube equipped with a stirring bar, the diphenyl ether **1** (0.2 mmol, 1.0 equiv.), diphosphane **29** (0.6 mmol, 3.0 equiv.), *t*BuOK (44 mg, 0.4 mmol, 2.0 equiv.), NHC **B** (15.6 mg, 0.04 mmol, 0.2 equiv.), and DMA (1.0 mL) were added. The resulting mixture was allowed to stir at 98 °C under blue LED irradiation for 12 hours. The targeted product **3** was obtained with 81% yield.

3.4. Confirm the generation of Ph_2P^-

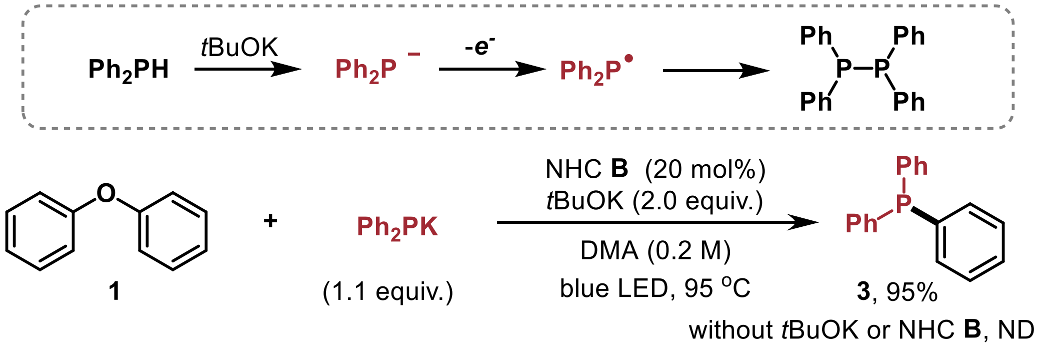

In a nitrogen-filled glovebox, to a dry tube equipped with a stirring bar, the diphenyl ether **1** (0.2 mmol, 1.0 equiv.), Ph_2PK (2.2 mmol, 1.1 equiv.), *t*BuOK (44 mg, 0.4 mmol, 2.0 equiv.), NHC **B** (15.6 mg, 0.04 mmol, 0.2 equiv.), and DMA (1.0 mL) were added. The resulting mixture was allowed to stir at 94 °C under blue LED irradiation for 12 hours. The desired product **3** was obtained with 95% yield. When *t*BuOK or NHC **B** was absent, the desired product **3** could not be detected.

3.5. Study possible pathways for the generation of Ph_2P•

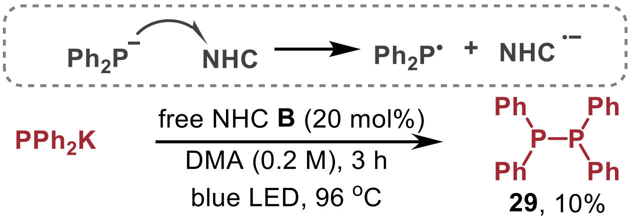

In a nitrogen-filled glovebox, to a dry tube equipped with a stirring bar, Ph_2PK (0.2 mmol, 1.0 equiv.), free NHC **B** (0.04 mmol, 0.2 equiv.), and DMA (1.0 mL) were added. The resulting mixture was allowed to stir at 96 °C under blue LED irradiation for 3.5 hours. The desired diphosphane **29** was observed with 9% yield by ^{31}P NMR spectra analysis using Ph_3PO as an internal standard.

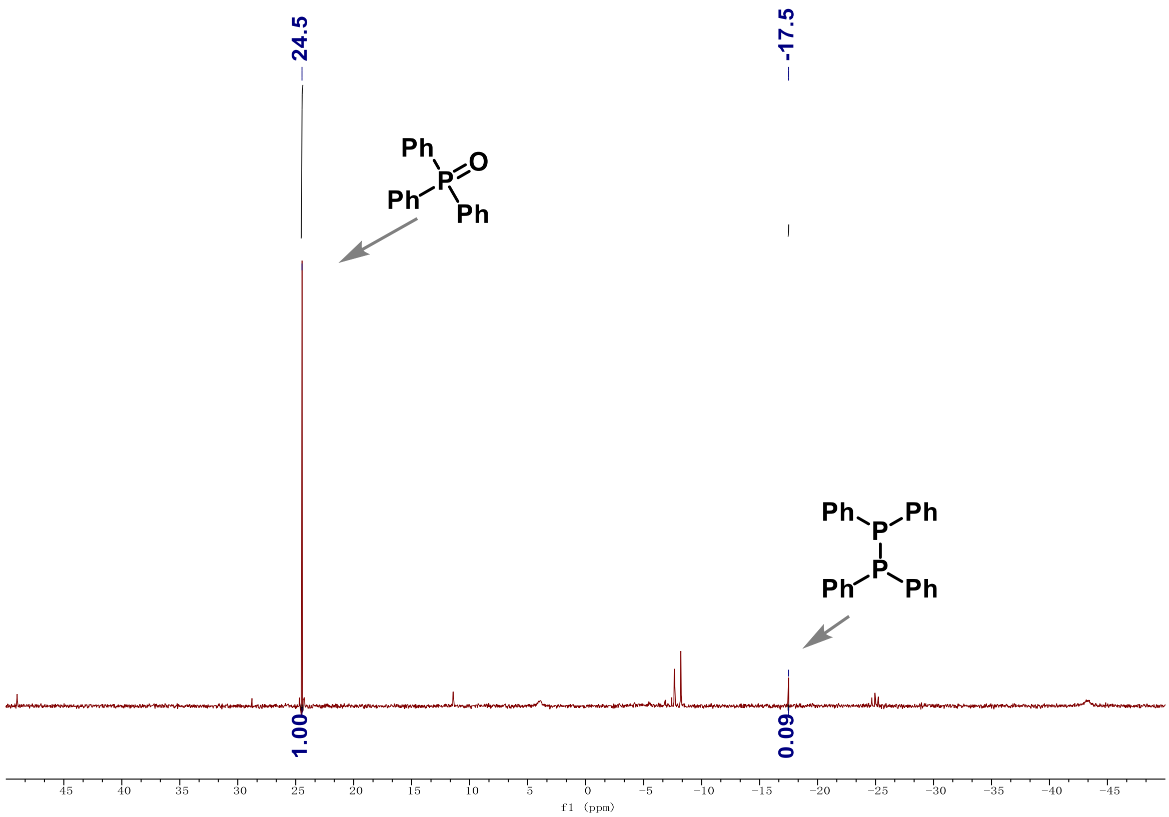

3.6. EPR spectroscopy experiments

Continuous-wave (CW) electron paramagnetic resonance (EPR) measurements were performed on DMA solutions in capillary tubes at ambient temperature with a Bruker EMXplus spectrometer at microwave frequencies of about 9.85 GHz. A Bruker super-high Q resonator (ER 4119HS) with slits in the cavity wall for optical excitation was used. As a light source, a 100 W blue LED (λ = 465 nm, PLS-LED100C) was placed outside the magnet 40 cm from the cavity wall. CW EPR spectra were measured with a microwave power of 2 mW and a modulation amplitude of 0.1 mT to avoid line broadening by saturation or overmodulation.

3.6.1. Characterization of the NHC radical anion

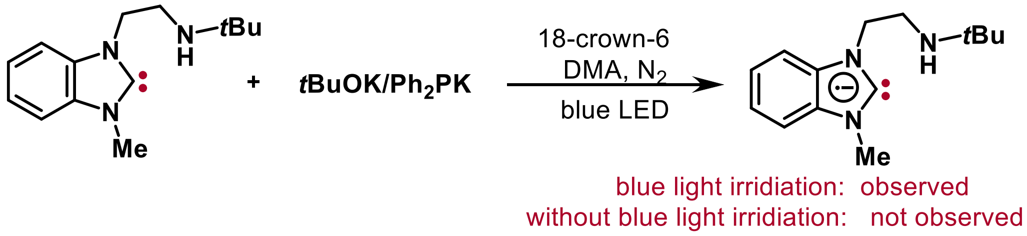

The NHC radical anion was generated upon mixing free NHC and *t*BuOK or Ph_2PK in dry DMA under blue light irradiation (λ = 465 nm). The recorded EPR spectrum (Figure S4) shows a resonance with g_{iso} = 2.0048, displaying a hyperfine splitting pattern indicative of couplings with two nitrogen atoms (1.59 and 2.86 G) and four hydrogen atoms (3.79, 1.66, 4.09, 3.48 G)

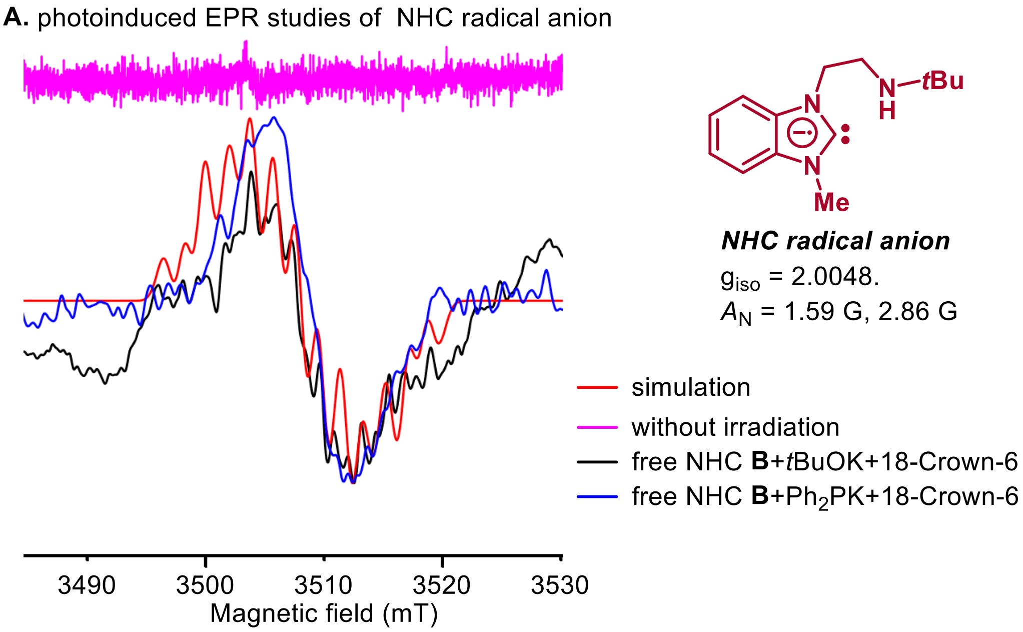

Figure S4. EPR spectrum of the NHC radical anion. The hyperfine couplings are A_N = 1.59, 2.86 G and A_H =3.79, 1.66, 4.09, 3.48 G for NHC radical anion.

3.6.2. Characterization of the methyl radical

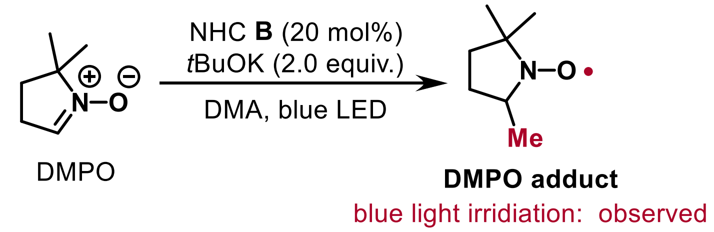

The methyl radical was generated upon mixing *t*BuOK, NHC **B**, and DMPO in dry DMA under blue light irradiation (λ = 465 nm). The recorded EPR spectrum (Figure S5) shows a resonance with g_{iso} = 2.0062, displaying a hyperfine splitting pattern indicative of couplings with one nitrogen atom (14.49 G) and one hydrogen atom (20.93 G)

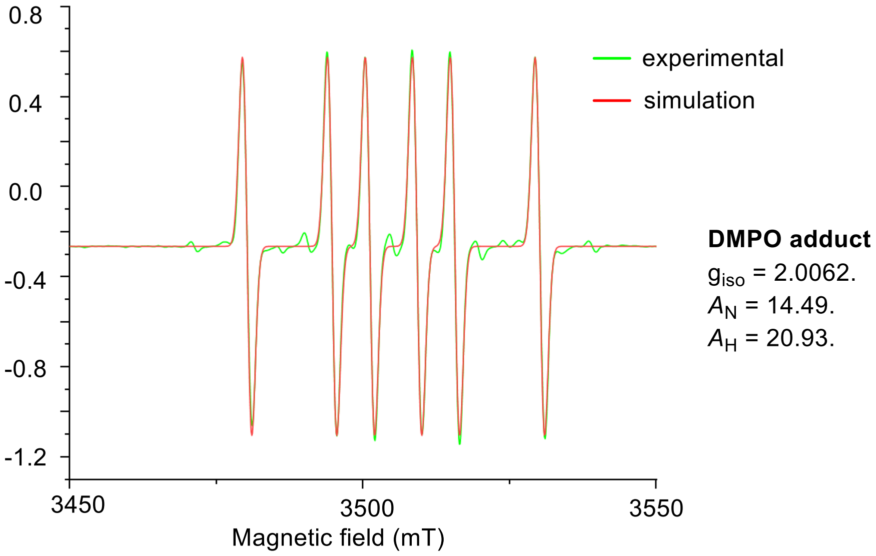

Figure S5. EPR spectrum of the methyl radical. The hyperfine couplings are A_N = 14.49 G and A_H =20.93 G for methyl radical.

3.6.3. Characterization of the diphenylphosphine radical

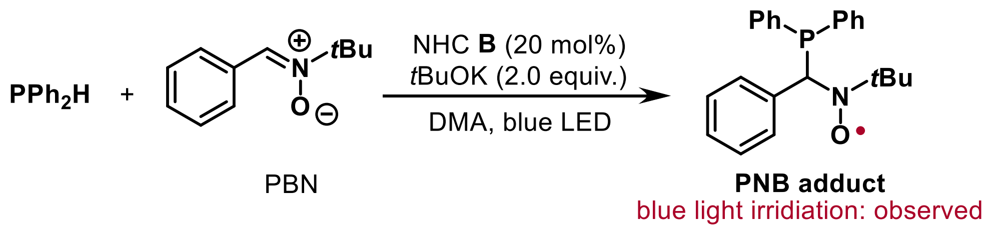

The diphenylphosphine radical was generated upon mixing the diphenyl ether **1** (0.2 mmol, 1.0 equiv.), Ph_2PH (0.22 mmol, 1.1 equiv.), PBN (0.6 mmol, 3.0 equiv.), *t*BuOK (0.44 mmol, 2.0 equiv.), NHC **B** (16 mg, 0.04 mmol, 0.2 eq), and *t*BuOK in dry DMA under blue light irradiation (λ = 465 nm). The recorded EPR spectrum (Figure S6) shows a resonance with g_{iso} = 2.0062, displaying a hyperfine splitting pattern indicative of couplings with one nitrogen atom (7.00 G)， one hydrogen atom (28.00 G), and one phosphine atom (21.10 G).

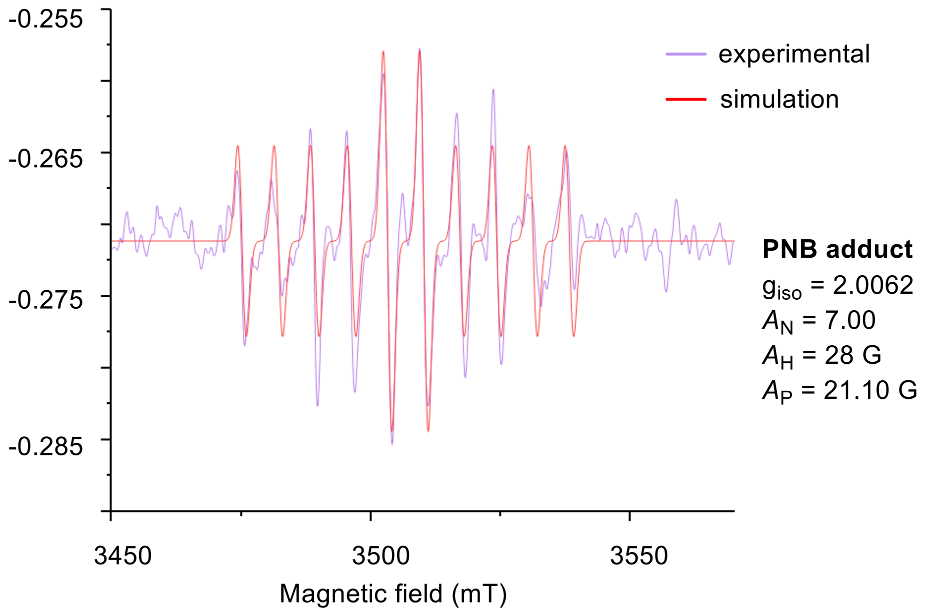

Figure S6. EPR spectrum of the diphenylphosphine radical. The hyperfine couplings are A_N = 7.00 G, A_H =28 and A_P =21.10 G for diphenylphosphine radical.

3.7. Quantum yield determination

According to the procedure of Xu[3]: To an oven-dried 10 mL glass tubes sealed with rubber septum, diphenyl ether **1** (34.0 mg, 0.2 mmol), diphenylphosphane **2** (40.9 mg, 0.22 mmol), NHC **B** (15.6 mg, 0.04 mmol), *t*BuOK (44.8 mg, 0.4 mmol) were combined in DMA (1 mL) under N_2 atmosphere. The reaction mixture was stirred and irradiated (λ = 460 nm, PLS-LED100C) for 1.0 h. After irradiation, the solution was measured the unit area photon flux (MQ-500 photosynthetic active radiation meter). And the yield of product formed was isolated. The quantum yield is calculated using the following equation：

$$\phi = \frac{mol\ product}{flux \cdot S \cdot t}$$

Where, Φ is quantum yield, S (m^2) is the irradiation area and t (s) is the photoreaction time. Experiment: the unit photon flux was 423 μmol·s^{-1}·m^{-2} (average of three experiments), the irradiation area was 1.0×1.4×10^{-4} m^2, and the product yield was 16% after 1.0 h (3600 s).

Quantum yield calculation:

$$\phi = \frac{mol\ product}{flux \cdot S \cdot t} = \frac{0.16 \times 0.2 \times 10^3}{524 \times 1.0 \times 1.4 \times 10^{-4} \times 3600} = 0.12$$

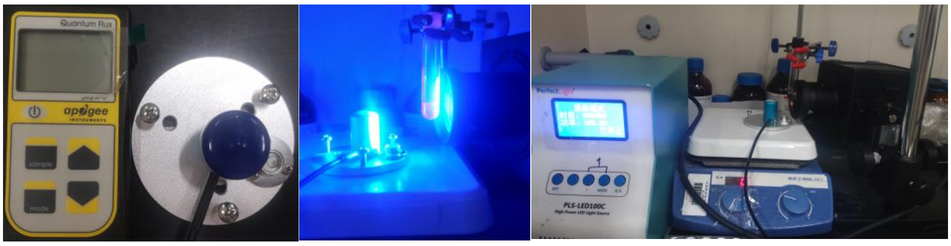

3.8. Light on-off experiment

In a nitrogen-filled glovebox, to a dry tube equipped with a stirring bar, the substrate **1** (0.2 mmol, 1.0 equiv.), Ph_2PH (0.22 mmol, 1.1 equiv.), *t*BuOK (44 mg, 0.4 mmol, 2.0 equiv), NHC **B** (15.6 mg, 0.04 mmol, 0.2 equiv.), and DMA (1.0 mL) were added. The resulting mixture was allowed to stir at 95 ± 5 °C under blue LED (100 W) irradiation for 1 h, then the reaction mixture was stirred in dark for another 1 h, and repeating this operation to the sixth hour. The yields of the reaction at 1 h, 2 h, 3 h, 4 h, 5 h, and 6 h were determined by GC-MS using n-hexadecane as the internal standard. The results showed that there ais no increase in product generation was observed without blue light irradiation, making a radical chain reaction less likely to be operative

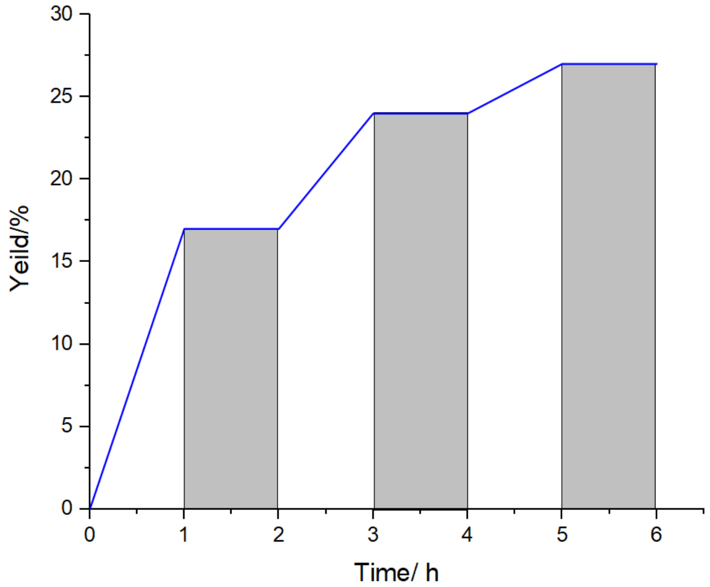

3.9. Determination of the binding ratio by Job's plot

Job's plot was performed according to the literature method.[4] 3,3'-oxybis(methylbenzene) **30**

and free NHC were dissolved in DMSO-d_6 (0.5 mL) to provide a mixed solution of 0.1 mol/L. Then, the solution was added to the NMR tube based on the specific ratio in Table S2. The $^1H(CH_3)$ chemical shift of free **30** solution (0.1 mol/L) is 2.2345 ppm. The Job's plot had a maximum when X (**30**) equals to 0.5, suggesting that the binding stoichiometry between free NHC **B** and **30** is 1:1.

Table S2. ^{1}H NMR experimental data for Job's plot.

**30** (M^{-1})	Free NHC **B** (M^{-1})	X(**30**)	δ (OCH_3, ppm)	Δδ (ppm)	Δδ* X (**30**)*10^3
0.10	0	1	2.2345	0	0
0.07	0.03	0.7	2.2335	0.0010	0.070
0.05	0.05	0.5	2.2325	0.0020	0.100
0.03	0.07	0.3	2.2324	0.0021	0.063
0.02	0.08	0.2	2.2315	0.0030	0.060
0.01	0.09	0.1	2.2335	0.0009	0.009

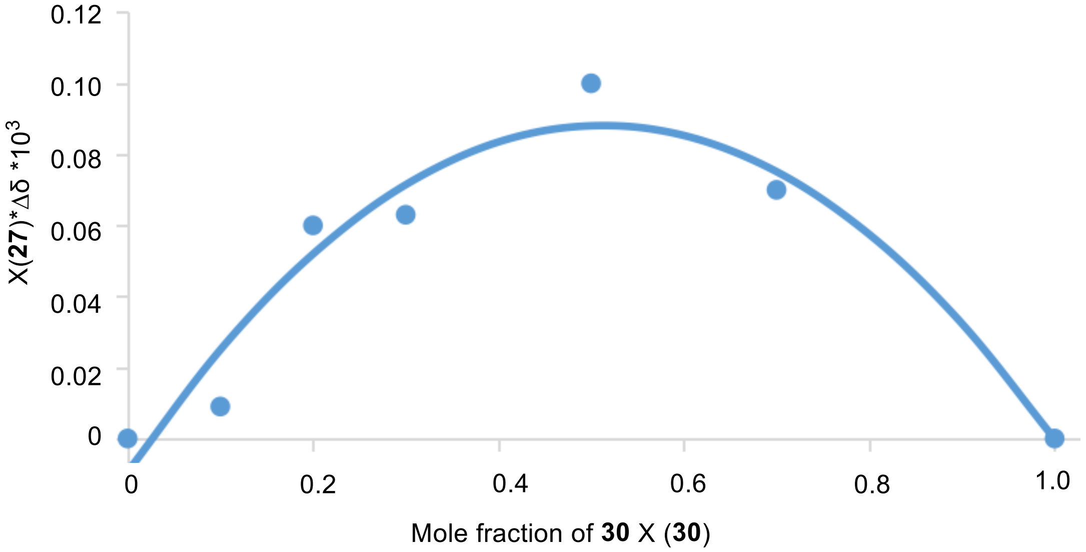

Figure S7. Job's plot for the determination of binding stoichiometry between **30** and free NHC **B.**

3.10. ^{1}H NMR titrations of *m*-tolyl ether with free NHC B

Compound **30** ($1.0*10^{-2}$ mmol) and free NHC **B** were thoroughly mixed in $CDCl_3$ (1.0 mL), then the resulted mixture was injected into an NMR tube. The ^{1}H NMR (500 MHz, $CDCl_3$, 25°C) spectra of the $1.0*10^{-2}$ M $CDCl_3$ solution of **30** with increasing concentration of free NHC **B** (corresponding concentration from bottom to top is 0.0, $1.0*10^{-2}$, $2.0*10^{-2}$, $3.0*10^{-2}$, $4.0*10^{-2}$, $6.0*10^{-2}$, $8.0*10^{-2}$, $9.0*10^{-2}$ M) were recorded and shown in Figure S8 and S9. The titration curve was obtained with the Bindfit program based on the ^{1}H titration experiment (Figure S9), and the association constant Ka between free NHC **B** and **30** was calculated to be 7.94 ± 0.01 M^{-1}.

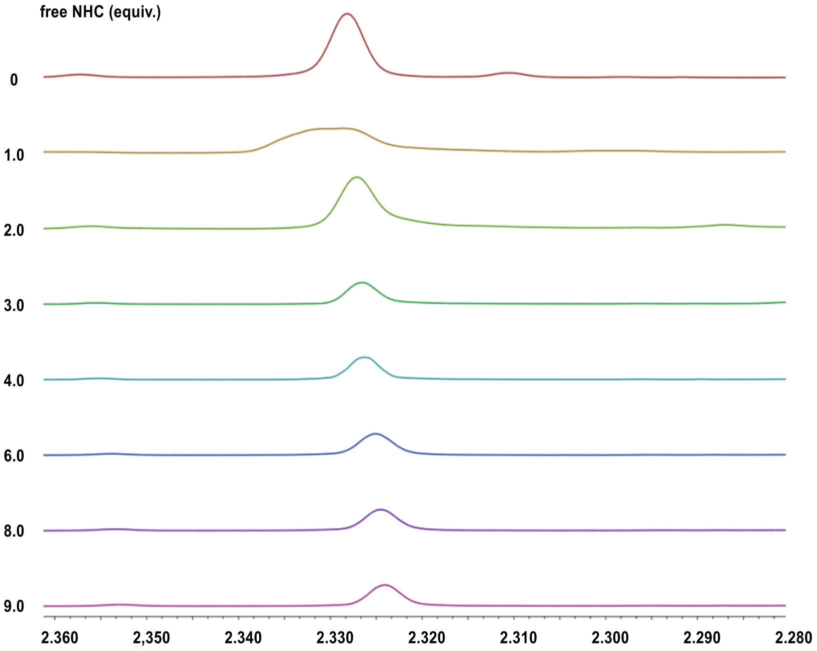

Figure S8. Partial ^{1}H NMR spectra recorded during the titration of **30** (1.0×10^{-2} M) with variable concentrations (0-9 equiv.) of free NHC **B** in $CDCl_3$.

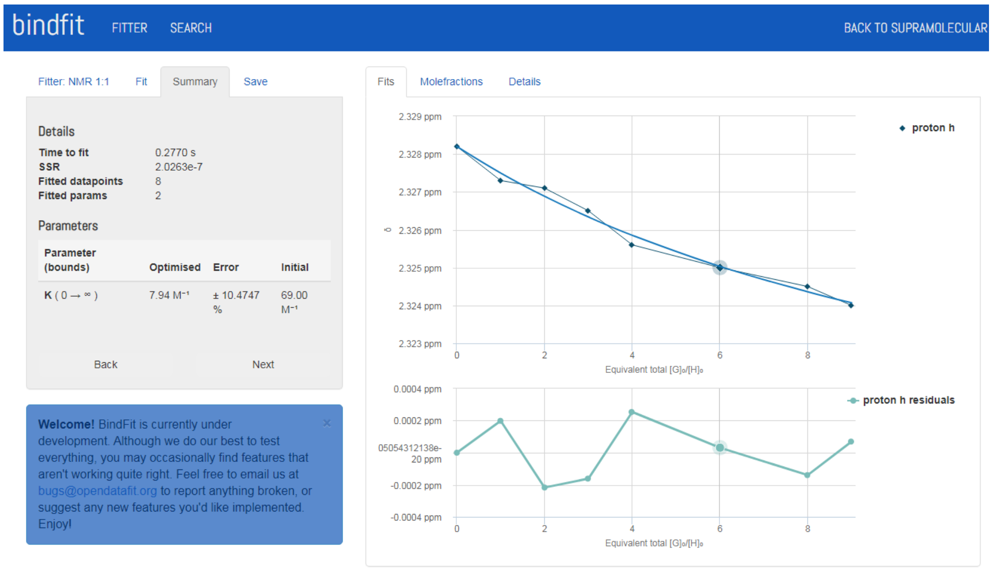

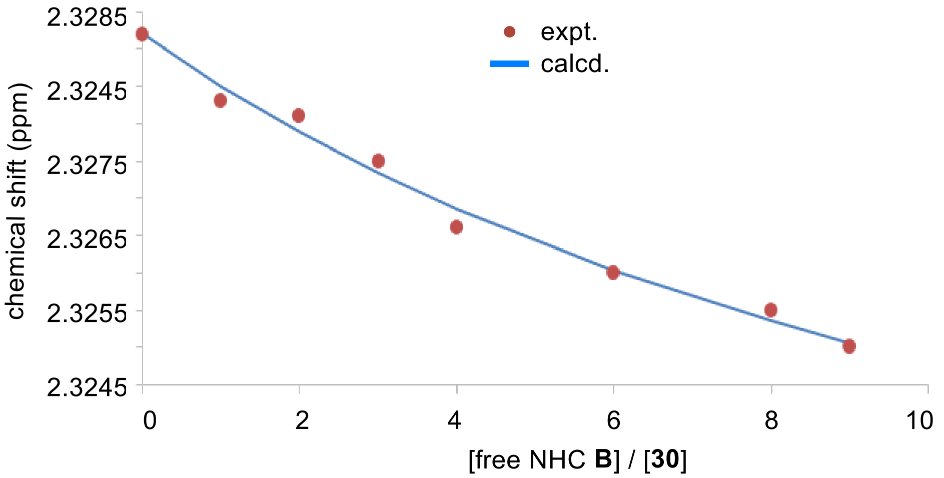

Figure S9. The curve fitting of the ^{1}H NMR titration data by Bindfit program, available online (http://supramolecular.org/); fitting output from Bindfit.

3.11. The dependence of the reaction yield on the excitation power

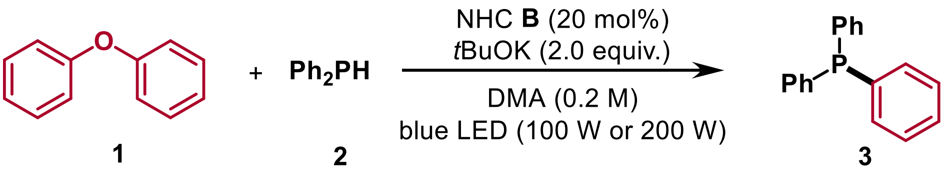

The irradiation power of blue LED was controlled by using one Kelo-AO100S blue LED lamp or two Kelo-AO100S blue LED lamps. The reaction was conducted in a 10 mL glass tube sealed with rubber septum. The diphenyl ether **1** (0.2 mmol, 1.0 equiv.), Ph_2PH (0.22 mmol, 1.1 equiv.), *t*BuOK (44 mg, 0.4 mmol, 2.0 equiv.), and NHC **B** (15.6 mg, 0.04 mmol, 0.2 equiv.) were combined in DMA (2.0 mL) under N_2 atmosphere. Reactions were stirred under irradiation with one Kelo AO100S blue LED lamp or two Kelo-AO100S blue LED lamps, respectively. The distance of the glass tubes from the light source is 0.2 cm. After 0.5 h, 1 h, 1.5 h, and 2.0 h, the corresponding yields were determined by GC-MS using n-hexadecane as the internal standard. As shown in Figure S10, doubling the irradiation power resulted in a doubled yield, suggesting a monophotonic mechanism in our reaction.

Time (h)	0.5	1.0	1.5	2.0
Yield (100W)	3%	6%	13%	18%
Yield (200W)	7%	14%	25%	34%

Figure S10. Reaction yield dependency on the irradiation intensity

4. Transformations of boron-coordinated phosphorus compounds

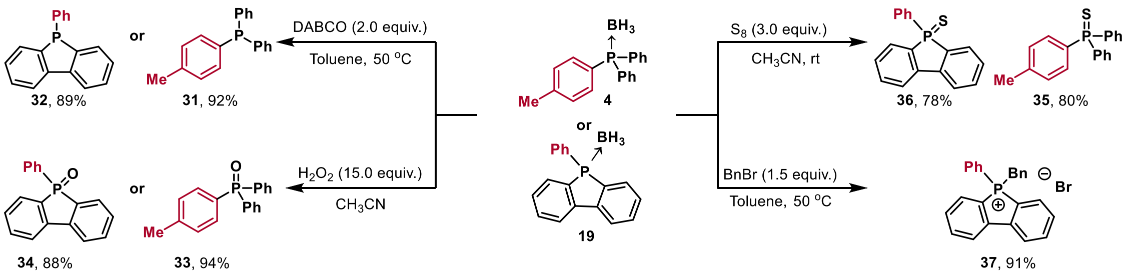

The boron-coordinated phosphorus could be easily transformed to other phosphorus compounds. For instance, the boron-coordinated products **4** and **19** could be readily converted to the corresponding trivalent phosphines, phosphine oxide compounds, phosphine sulfur compound, and phosphonium compounds.

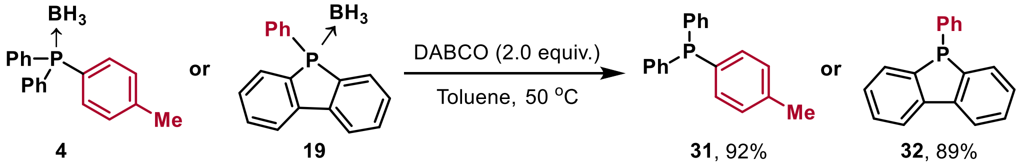

To a dry tube equipped with a stirring bar, the boron-coordinated phosphorus **4** or **19** (0.2 mmol, 1.0 equiv.), DABCO (0.4 mmol, 2.0 equiv.), and toluene (1.0 mL) were added. The resulting mixture was allowed to stir at 50 °C for 12 hours. The solvent was removed under vacuum and the residue was subjected to silica gel chromatography using petroleum ether as eluent to afford the desired product **31** or **32**.

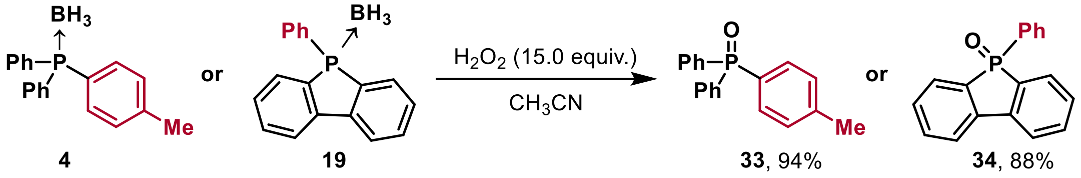

To a dry tube equipped with a stirring bar, the boron-coordinated phosphorus **4** or **19** (0.2 mmol, 1.0 equiv.), H_2O_2 (30 mmol, 15.0 equiv.), and CH_3CN (1.0 mL) were added. The resulting mixture was allowed to stir at room temperature for 4 hours. The solvent was removed under vacuum and the residue was subjected to silica gel chromatography using petroleum ether and ethyl acetate (2:1) as eluent to afford the desired product **33** or **34**.

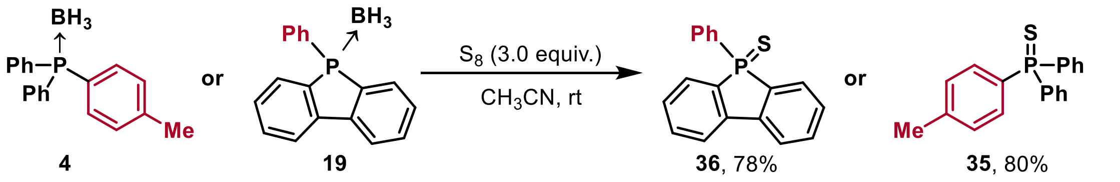

To a dry tube equipped with a stirring bar, the boron-coordinated phosphorus **4** or **19** (0.2 mmol, 1.0 equiv.), S_8 (0.6 mmol, 3.0 equiv.), and CH_3CN (1.0 mL) were added. The resulting mixture was allowed to stir at room temperature for overnight. Then the solid was filtered off and washed with DCM. The filtrate was concentrated under vacuum and the residue was subjected to silica gel

chromatography using petroleum ether and ethyl acetate (5:1) as eluent to afford the desired product **35** or **36**.

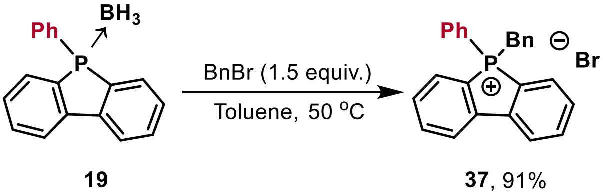

To a dry tube equipped with a stirring bar, the boron-coordinated phosphorus **19** (0.2 mmol, 1.0 equiv.), BnBr (0.3 mmol, 1.5 equiv.), and toluene (1.0 mL) were added. The resulting mixture was allowed to stir at 50 °C for overnight. The reaction mixture was filtered, and the solid was washed with ether to give the desired product **37** as a white solid.

5. Compound characterization data

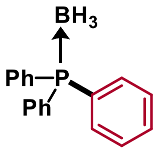

Triphenylphosphane borane adduct (3):

Following the general procedure I, the title product was obtained from **oxydibenzene** after purification by column chromatography (PE/EA = 100:1) as a white solid (45.3 mg, 0.164 mmol, 82%). **^{1}H NMR** (500 MHz, $CDCl_3$) δ 7.61 – 7.54 (m, 6H), 7.53 – 7.48 (m, 3H), 7.46 – 7.41 m, 6H), 1.61 – 0.96 (m, 3H). **^{13}C NMR** (126 MHz, $CDCl_3$) δ 133.3 (d, *J* = 10.1 Hz), 131.4 (d, *J* = 1.26 Hz), 129.27 (d, *J* = 58.0 Hz), 128.9 (d, *J* = 10.1 Hz). **^{11}B NMR** (160 MHz, $CDCl_3$) δ -38.90 (d, *J* = 56.5 Hz). **^{31}P NMR** (202 MHz, $CDCl_3$) δ 21.24 (d, *J* = 75.9 Hz). These data are in agreement with those reported previously in the literature.[5]

Following the general procedure I, the title product **3** could also be obtained from **oligomeric phenylene oxide (26)** with 54% yield (29.8 mg, 0.108 mmol).

Following the general procedure I, the title product **3** could also be obtained from **1-cyclohexyl-4-phenoxybenzene** with 69% yield (38.1 mg, 0.138 mmol).

Following the general procedure I, the title product **3** could also be obtained from **2,4-dimethyl-1-phenoxybenzene** with 65% yield (35.9 mg, 0.130 mmol).

Following the general procedure I, the title product **3** could also be obtained from **1-phenoxy-4-vinylbenzene** with 56% yield (30.9 mg, 0.112 mmol).

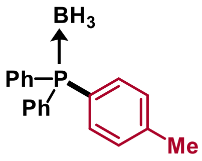

Diphenyl(p-tolyl)phosphane borane adduct (4):

Following the general procedure I, the title product was obtained after purification by column chromatography (PE/EA = 100:1) as a white solid (34.8 mg, 0.120 mmol, 60%). **^{1}H NMR** (500 MHz, $CDCl_3$) δ 7.60 – 7.53 (m, 4H), 7.50 – 7.44 (m, 4H), 7.43 – 7.37 (m, 4H), 7.25 – 7.21 (m, 2H), 2.37 (s, 3H), 1.63 – 0.87 (m, 3H).**^{13}C NMR** (126 MHz, $CDCl_3$) δ 141.9 (d, *J* = 1.3 Hz), 133.3 (d, *J* = 10.1 Hz), 133.2 (d, *J* = 7.6 Hz), 131.3 (d, *J* = 2.5 Hz), 129.6 (d, *J* = 58.0 Hz), 129.7 (d, *J* = 10.1 Hz), 128.9 (d, *J* = 10.1 Hz), 125.7 (d, *J* = 60.5 Hz), 125.4, 21.6. **^{11}B NMR** (160 MHz, $CDCl_3$) δ -38.79 (d, *J* = 68.3 Hz). **^{31}P NMR** (202 MHz, $CDCl_3$) δ 20.51 (d, *J* = 71.5 Hz). These data are in agreement with those reported previously in the literature.[5]

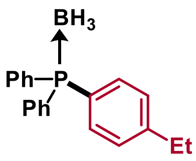

(4-Ethylphenyl)diphenylphosphane borane adduct (5):

Following the general procedure I, the title product was obtained after purification by column chromatography (PE/EA = 100:1) as a white solid (46.2 mg, 0.152 mmol, 76%). **^{1}H NMR** (500 MHz, $CDCl_3$) δ 7.57 – 7.54 (m, 4H), 7.52 – 7.46 (m, 4H), 7.45 – 7.40 (m, 4H), 7.30 – 7.23 (m, 2H), 2.68 (q, *J* = 7.6 Hz, 2H), 1.63 – 0.83 (m, 6H).**^{13}C NMR** (126 MHz, $CDCl_3$) δ 148.0, 133.4 (d, *J* = 8.8 Hz), 133.3 (d, *J* = 6.3 Hz), 131.2 (d, *J* = 2.5 Hz), 129.6 (d, *J* = 58.0 Hz), 128.8 (d, *J* = 10.1 Hz), 128.5 (d, *J* = 10.1 Hz), 125.8 (d, *J* = 59.2 Hz), 28.9, 15.3. **^{11}B NMR** (160 MHz, $CDCl_3$) δ -38.86 (d, *J* = 57.5 Hz). **^{31}P NMR** (202 MHz, $CDCl_3$) δ 20.43 (d, *J* = 79.3 Hz). **MP**: 93 – 94 °C. **IR** (ATR): 2950, 2442, 2412, 1693, 1558, 1456, 1435, 1265, 1107, 1060, 732 cm^{-1}.

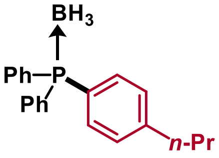

Diphenyl(4-propylphenyl)phosphane borane adduct (6):

Following the general procedure I, the title product was obtained after purification by column chromatography (PE/EA = 100:1) as a white solid (34.3 mg, 0.108 mmol, 54%). **^{1}H NMR** (500 MHz, $CDCl_3$) δ 7.61 – 7.54 (m, 4H), 7.51 – 7.46 (m, 4H), 7.44 – 7.39 (m, 4H), 7.26 – 7.22 (m, 2H), 2.61 (t, *J* = 7.7 Hz, 2H), 1.64 (q, *J* = 7.5 Hz, 2H), 1.59 – 0.98 (m, 3H), 0.94 (t, *J* = 7.3 Hz, 3H).**^{13}C NMR** (126 MHz, $CDCl_3$) δ 146.5 (d, *J* = 2.5 Hz), 133.3 (d, *J* = 10.1 Hz), 133.3 (d, *J* = 10.1 Hz), 131.2 (d, *J* = 2.5 Hz), 129.6 (d, *J* = 58.0Hz), 129.1 (d, *J* = 11.3 Hz), 128.8 (d, *J* = 10.1 Hz), 125.8 (d, *J* = 60.5 Hz), 38.0, 24.3, 13.9. **^{11}B NMR** (160 MHz, $CDCl_3$) δ -38.83 (d, *J* = 57.5 Hz). **^{31}P NMR** (202 MHz, $CDCl_3$) δ 20.43 (d, *J* = 76.2 Hz). **MP**: 97 – 98 °C. **IR** (ATR): 2950, 2447, 2412, 1693, 1558, 1456, 1435, 1265, 1107, 1060, 785 cm^{-1}. **HRMS** (ESI): *m/z* $[M+Na]^+$ calcd for $C_{21}H_{24}BPNa^+$: 341.1601; found: 341.1600.

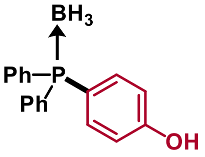

4-(Diphenylphosphaneyl)phenol borane adduct (7):

Following the general procedure I, the title product was obtained after purification by column chromatography (PE/EA = 100:1) as a white solid (40.3 mg, 0.138 mmol, 69%). **^{1}H NMR** (500 MHz, DMSO-*d_6*) δ 10.22 (s, 1H), 7.54 – 7.42 (m, 10H), 7.37 – 7.30 (m, 2H), 6.92 – 6.87 (m, 2H), 1.33 – 0.94 (m, 3H). **^{13}C NMR** (126 MHz, DMSO-*d_6*) δ 161.1, 135.3 (d, *J* = 11.0 Hz), 133.0 (d, *J* = 9.6 Hz), 131.9, 130.1 (d, *J* = 57.9 Hz), 129.6(d, *J* = 10.2 Hz), 116.78 (d, *J* = 11.1 Hz), 116.88 (d, *J* = 63.0 Hz). **^{11}B NMR** (160 MHz, DMSO-*d_6*) δ -37.95. **^{31}P NMR** (202 MHz, DMSO-*d_6*) δ 18.21. These data are in agreement with those reported previously in the literature. [5]

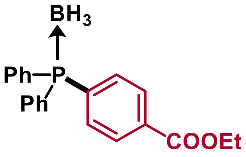

Ethyl 4-(diphenylphosphaneyl)benzoate borane adduct (8):

Following the general procedure I, the title product was obtained after purification by column chromatography (PE/EA = 100:1) as a white solid (35.5 mg, 0.102 mmol, 51%). **^{1}H NMR** (500 MHz, $CDCl_3$) δ 8.09 (d, *J* = 7.8 Hz, 2H), 7.68 – 7.61 (m, 2H), 7.61 – 7.55 (m, 4H), 7.54 – 7.50 (m, 2H), 7.49 – 7.40 (m, 4H), 4.39 (q, *J* = 7.1 Hz, 2H), 1.63 – 0.74 (m, 6H). **^{13}C NMR** (126 MHz, $CDCl_3$) δ 165.9, 135.0 (d, *J* = 55.2 Hz), 133.3 (d, *J* = 9.6 Hz), 133.2, 133.1, 131.7, (d, *J* = 2.5 Hz), 129.7 (d, *J* = 10.3 Hz), 129.1 (d, *J* = 10.2 Hz), 128.5 (d, *J* = 58.0 Hz), 61.5, 14.4. **^{11}B NMR** (160 MHz, $CDCl_3$) δ -38.93 (d, *J* = 63.9 Hz). **^{31}P NMR** (202 MHz, $CDCl_3$) δ 21.86 (d, *J* = 84.7 Hz). These data are in agreement with those reported previously in the literature.[6]

Following the general procedure I, the title product **8** could also be obtained from **ethyl 4-(4-cyclohexylphenoxy)benzoate--ethane** with 41% yield (28.5 mg, 0.082 mmol).

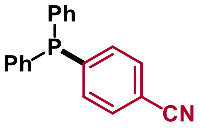

4-(Diphenylphosphaneyl)benzonitrile (9):

Following the general procedure I, the title product was obtained from **3,3'-oxydibenzonitrile** after purification by column chromatography (PE/EA = 100:1) as a white solid (24.2 mg, 0.084 mmol, 42%). **^{1}H NMR** (500 MHz, $CDCl_3$) δ 7.57 (d, *J* = 7.9 Hz, 2H), 7.42 – 7.34 (m, 6H), 7.34 – 7.27 (m, 6H). **^{13}C NMR** (126 MHz, $CDCl_3$) δ 145.09 (d, *J* = 16.7 Hz), 135.34 (d, *J* = 10.4 Hz), 134.02 (d, *J* = 20.3 Hz), 133.46 (d, *J* = 18.5 Hz), 131.69 (d, *J* = 6.0 Hz), 129.48, 128.86 (d, *J* = 7.5 Hz), 118.72, 111.86. **^{31}P NMR** (202 MHz, $CDCl_3$) δ -3.6. These data are in agreement with those reported previously in the literature.[6]

Following the general procedure I, the title product **9** could also be obtained from

3-phenoxybenzonitrile with 30% yield (17.3 mg, 0.060 mmol).

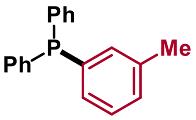

Diphenyl(*m*-tolyl)phosphane (10):
Following the general procedure I, the title product was obtained after purification by column chromatography (PE/EA = 100:1) as a white solid (32.0 mg, 0.116 mmol, 58%). **^{1}H NMR** (500 MHz, $CDCl_3$) δ 7.34 – 7.27 (m, 10H), 7.25 – 7.20 (m, 1H), 7.18 – 7.13 (m, 2H), 7.09 – 7.04 (m, 1H), 2.30 (s, 3H). **^{13}C NMR** (126 MHz, $CDCl_3$) δ 138.2 (d, *J* = 7.6 Hz), 137. (d, *J* = 11.3 Hz), 137.0 (d, *J* = 10.1 Hz), 134.6 (d, *J* = 21.4 Hz), 133.8 (d, *J* = 18.9Hz), 130.9 (d, *J* = 17.6 Hz), 129.7, 128.7, 128.6, 128.5 (d, *J* = 6.3 Hz), 21.5. **^{31}P NMR** (202 MHz, $CDCl_3$) δ -4.70. These data are in agreement with those reported previously in the literature. [6]

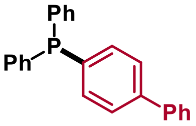

[1,1'-Biphenyl]-4-yldiphenylphosphane (11):
Following the general procedure I, the title product was obtained after purification by column chromatography (PE/EA = 100:1) as a white solid (41.2 mg, 0.122 mmol, 61%). **^{1}H NMR** (500 MHz, $CDCl_3$) δ 7.61 – 7.53 (m, 4H), 7.45 – 7,40 (m, 2H), 7.40 – 7.32 (m, 13H). **^{13}C NMR** (126 MHz, $CDCl_3$) δ 141.6, 140.7, 137.3 (d, *J* = 10.8 Hz), 136.2 (d, *J* = 10.8 Hz), 134.3 (d, *J* = 19.5 Hz), 133.9 (d, *J* = 19.5 Hz), 128.9 (d, *J* = 7.2 Hz), 128.7 (d, *J* = 7.1 Hz), 127.7, 127.3 (d, *J* = 7.0 Hz), 127.2. **^{31}P NMR** (202 MHz, $CDCl_3$) δ -5.47. These data are in agreement with those reported previously in the literature. [6]

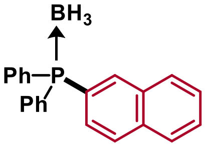

Naphthalen-2-yldiphenylphosphane borane adduct (12):
Following the general procedure I, the title product was obtained from **2,2'-oxydinaphthalene** after purification by column chromatography (PE/EA = 100:1) as a white solid (46.3 mg, 0.142 mmol, 71%). **^{1}H NMR** (500 MHz, $CDCl_3$) δ 8.13 (d, *J* = 12.8 Hz, 1H), 7.90 – 7.80 (m, 3H), 7.65 – 7.59 (m, 4H), 7.59 – 7.55 (m, 2H), 7.54 – 7.48 (m, 3H), 7.46 – 7.41 (m, 4H), 1.62 – 1.18 (m, 3H). **^{13}C NMR** (126 MHz, $CDCl_3$) δ 135.0 (d, *J* = 11.3 Hz), 134.4 (d, *J* = 2.5 Hz), 133.4 (d, *J* = 8.8 Hz), 132.8 (d, *J* = 11.7 Hz), 131.4 (d, *J* = 2.4 Hz), 129.3 (d, *J* = 58.0 Hz), 129.0 (d, *J* = 10.3 Hz), 128.8, 128.7 (d, *J* = 9.8 Hz), 128.24, 128.16, 127.9, 127.0, 126.4 (d, *J* = 58.1 Hz). **^{11}B NMR** (160 MHz, $CDCl_3$) δ -38.91. **^{31}P NMR** (202 MHz, $CDCl_3$) δ 21.52 (d, *J* = 73.9 Hz).**MP**: 101 – 102 °C. **IR** (ATR): 3090, 2984, 2360, 2276, 1430, 1265, 889, 765, 704 cm^{-1}. **HRMS** (ESI): *m/z* $[M+H]^+$ calcd for $C_{22}H_{21}BP^+$: 327.1468; found: 327. 1464.

Following the general procedure I, the title product **12** could also be obtained from **3-phenoxybenzonitrile** with 63% yield (41.1 mg, 0.126 mmol).

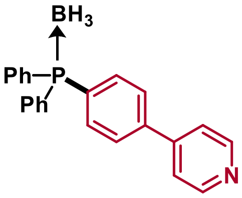

4-(4-(Diphenylphosphaneyl)phenyl)pyridine borane adduct (13):
Following the general procedure I, the title product was obtained from **4,4'-(oxybis(4,1-phenylene))dipyridine** after purification by column chromatography (PE/EA = 100:1) as a white solid (41.7 mg, 0.118 mmol, 59%). **^{1}H NMR** (500 MHz, $CDCl_3$) δ 8.66 (d, *J* = 6.0 Hz, 2H), 7.77 – 7.67 (m, 6H), 7.65 – 7.58 (m, 4H), 7.58 – 7.53 (m, 2H), 7.51 – 7.45 (m, 4H), 1.44 – 1.09 (m, 4H). **^{13}C NMR** (126 MHz, $CDCl_3$) δ 150.3, 148.1, 138.5, 134.4 (d, *J* = 10.1 Hz), 133.4 (d, *J* = 8.8 Hz), 132.8 (d, *J* = 56.7 Hz), 131.8 (d, *J* = 2.5 Hz), 129.1 (d, *J* = 10.1 Hz), 128.4 (d, *J* = 58.0 Hz),

127.6 (d, *J* = 10.1 Hz), 123.2. ^{11}**B NMR** (160 MHz, $CDCl_3$) δ -34.76 – -42.00 (m). ^{31}**P NMR** (202 MHz, $CDCl_3$) δ 21.69.

Following the general procedure I, the title product **13** could also be obtained from **4-(4-phenoxyphenyl)pyridine** with 90% yield (63.6 mg, 0.180 mmol).

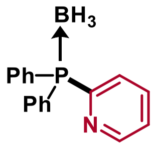

2-(Diphenylphosphaneyl)pyridine borane adduct(14):

Following the general procedure I, the title product was obtained from **2,2'-oxydipyridine** after purification by column chromatography (PE/EA = 20:1) as a white solid (26.8 mg, 0.102 mmol, 51%). ^{1}**H NMR** (500 MHz, $CDCl_3$) δ 8.79 (d, *J* = 4.7 Hz, 1H), 8.07 (t, *J* = 7.0 Hz, 1H), 7.79 – 7.73 (m, 5H), 7.52 – 7.46 (m, 2H), 7.45 – 7.40 (m, 4H), 7.36 (t, *J* = 6.5 Hz, 1H), 1.46 – 0.93 (m, 3H). ^{13}**C NMR** (126 MHz, $CDCl_3$) δ 154.5 (d, *J* = 76.5 Hz), 150.8 (d, *J* = 12.8 Hz), 136.3 (d, *J* = 10.0 Hz), 133.5 (d, *J* = 9.6 Hz), 131.4 (d, *J* = 2.5 Hz), 130.1 (d, *J* = 26.5 Hz), 128.79 (d, *J* =59.2 Hz), 128.69 (d, *J* = 26.5 Hz), 128.6, 125.0 (d, *J* = 3.0 Hz). ^{11}**B NMR** (160 MHz, $CDCl_3$) δ -39.5 (d, *J* = 55.3 Hz). ^{31}**P NMR** (202 MHz, $CDCl_3$) δ 19.05 (d, *J* = 75.0 Hz). These data are in agreement with those reported previously in the literature. [7]

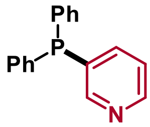

3-(Diphenylphosphaneyl)pyridine (15):

Following the general procedure I, the title product was obtained from **3,3'-oxydipyridine** after purification by column chromatography (PE/EA = 20:1) as a white solid (36.8 mg, 0.140 mmol, 70%). ^{1}**H NMR** (500 MHz, $CDCl_3$) δ 8.59 – 8.50 (m, 2H), 7.55 (t, *J* = 7.4 Hz, 1H), 7.42 – 7.28 (m, 10H), 7.25 – 7.20 (m, 1H). ^{13}**C NMR** (126 MHz, $CDCl_3$) δ 154.3 (d, *J* = 23.7 Hz), 149.7, 141.1(d, *J* = 15.8 Hz), 135.9(d, *J* = 10.3 Hz), 133.8 (d, *J* = 19.9 Hz), 129.3, 128.9 (d, *J* = 7.0 Hz), 123.6 (d, *J* = 4.1 Hz). ^{31}**P NMR** (202 MHz, $CDCl_3$) δ -11.15. These data are in agreement with those reported previously in the literature. [6]

Following the general procedure I, the title product **15** could also be obtained from **3-phenoxypyridine** with 54% yield (28.4 mg, 0.108 mmol).

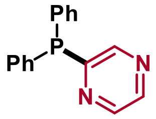

2-(Diphenylphosphaneyl)pyrazine (16):

Following the general procedure I, the title product was obtained from **2,2'-oxydipyrazine** after purification by column chromatography (PE/EA = 10:1) as a white solid (18.5 mg, 0.070 mmol, 35%). ^{1}**H NMR** (500 MHz, $CDCl_3$) δ 8.66 (s, 1H), 8.44 (s, 1H), 8.35 (s, 1H), 7.46 – 7.41 (m, 4H), 7.40 – 7.34 (m, 6H). ^{13}**C NMR** (126 MHz, $CDCl_3$) δ 160.5, 148.4 (d, *J* = 19.1 Hz), 145.3 (d, *J* = 8.6 Hz), 143.0 (d, *J* = 6.6 Hz), 134.8 (d, *J* = 9.5 Hz), 134.3 (d, *J* = 20.2 Hz), 129.7, 128.9 (d, *J* = 7.4 Hz). ^{31}**P NMR** (202 MHz, $CDCl_3$) δ -8.0. These data are in agreement with those reported previously in the literature.[8]

Following the general procedure I, the title product **16** could also be obtained from **2-phenoxypyrazine** with 57% yield (30.1 mg, 0.114 mmol).

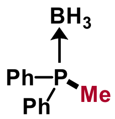

Methyldiphenylphosphane borane adduct (17):

Following the general procedure I, the title product was obtained after purification by column chromatography (PE/EA = 100:1) as a colorless oil (33.4 mg, 0.156 mmol, 78%). ^{1}**H NMR** (500 MHz, $CDCl_3$) δ 7.69 – 7.61 (m, 4H), 7.49 – 7.23 (m, 6H), 1.85 (d, *J* = 10.1 Hz, 3H), 1.39 – 0.66 (m, 3H). ^{13}**C NMR** (126 MHz, $CDCl_3$) δ 131.9 (d, *J* = 9.5 Hz), 131.3 (d, *J* = 2.4 Hz), 130.6 (d, *J* = 56.5 Hz), 129.0 (d, *J* = 10.1 Hz), 12.0 (d, *J* = 40.3 Hz). ^{11}**B NMR** (160 MHz,

CDCl3) δ -38.91 (d, J = 58.8 Hz). **^{31}P NMR** (202 MHz, CDCl3) δ 11.46 – 9.79 (m). These data are in agreement with those reported previously in the literature. [9]

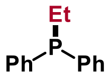

Ethyldiphenylphosphane (18):

Following the general procedure I, the title product was obtained after purification by column chromatography (PE) as a colorless oil (23.1 mg, 0.108 mmol, 54%). **^{1}H NMR** (400 MHz, CDCl3) δ 7.42 – 7.37 (m, 4H), 7.32 – 7.21 (m, 6H), 2.01 (q, J = 7.6 Hz, 2H), 1.10 -1.02 (m, 3H). **^{13}C NMR** (101 MHz, CDCl3) δ 138.9 (d, J = 13.0 Hz), 132.8 (d, J = 17.9 Hz), 130.1 – 124.2 (m), 20.7 (d, J = 10.2 Hz), 10.2 (d, J = 16.6 Hz). **^{31}P NMR** (162 MHz, CDCl3) δ –18.86 (m). These data are in agreement with those reported previously in the literature. [9]

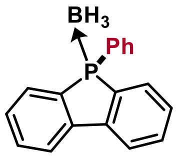

5-Phenyl-5H-benzo[b]phosphindole borane adduct (19):

Following the general procedure I, the title product was obtained after purification by column chromatography (PE/EA = 100:1) as a white solid (39.5 mg, 0.144 mmol, 72%) **^{1}H NMR** (500 MHz, CDCl3) δ 7.91 (d, J = 7.2 Hz, 1H), 7.70 (t, J = 7.9 Hz, 2H), 7.61 – 7.52 (m, 4H), 7.45 – 7.36 (m, 3H), 7.36 – 7.28 (m, 2H), 1.43 – 0.84 (m, 3H). **^{13}C NMR** (126 MHz, CDCl3) δ 143.52 (d, J = 10.1 Hz), 133.67 (d, J = 61.4 Hz), 132.31 (d, J = 10.3 Hz), 132.11 (d, J = 2.0 Hz), 131.83 (d, J = 3.0 Hz), 130.62 (d, J = 12.6 Hz), 129.23 (d, J = 10.4 Hz), 129.05 (d, J = 10.4 Hz), 128.06 (d, J = 50.9 Hz), 121.84 (d, J = 6.3 Hz). **^{11}B NMR** (160 MHz, CDCl3) δ -40.54 (d, J = 46.6 Hz). **^{31}P NMR** (202 MHz, CDCl3) δ 25.57 (d, J = 58.5 Hz). These data are in agreement with those reported previously in the literature. [10]

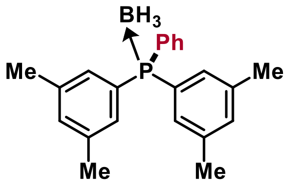

Bis(3,5-dimethylphenyl)(phenyl)phosphane borane adduct (20):

Following the general procedure I, the title product was obtained after purification by column chromatography (PE/EA = 100:1) as a white solid (35.2 mg, 0.106mmol, 53%). **^{1}H NMR** (500 MHz, CDCl3) δ 7.60 – 7.54 (m, 2H), 7.51 – 7.46 (m, 1H), 7.45 – 7.39 (m, 2H), 7.16 (d, J = 11.3 Hz, 4H), 7.11 (s, 2H), 2.30 (s, 12H), 1.37 – 0.92 (m, 3H). **^{13}C NMR** (126 MHz, CDCl3) δ 138.44 (d, J = 10.8 Hz), 133.29 (d, J = 9.5 Hz), 133.13 (d, J = 2.5 Hz), 131.09 (d, J = 2.5 Hz), 130.85 (d, J = 9.6 Hz), 129.2, 128.73 (d, J = 10.1 Hz), 119.0, 21.4. **^{11}B NMR** (160 MHz, CDCl3) δ -38.74. **^{31}P NMR** (202 MHz, CDCl3) δ 20.37 (d, J = 79.4 Hz). **MP**: 107 – 108 °C. **IR** (ATR): 2983, 2941, 1736, 1446, 1373, 1244, 1047, 736 cm^{-1}. **HRMS** (ESI): m/z [M+H]$^+$ calcd for $C_{22}H_{27}BP^+$: 333.1938; found: 333. 1936.

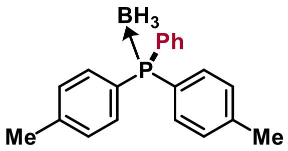

Phenyl di-p-tolylphosphane borane adduct (21):

Following the general procedure I, the title product was obtained after purification by column chromatography (PE/EA = 100:1) as a white solid (32.2 mg, 0.106 mmol, 53%). **^{1}H NMR** (500 MHz, CDCl3) δ 7.58 – 7.53 (m, 2H), 7.50 – 7.43 (m, 5H), 7.43 – 7.39 (m, 2H), 7.25 – 7.21 (m, 4H), 2.38 (s, 6H), 1.40 – 0.94 (m, 4H). **^{13}C NMR** (126 MHz, CDCl3) δ 141.7 (d, J = 2.4 Hz), 133.3 (d, J = 10.3 Hz), 133.1, 132.17 (d, J = 9.2 Hz), 131.1(d, J = 2.4 Hz), 129.6 (d, J = 10.5 Hz), 128.8(d, J = 10.2 Hz), 126.0 (d, J = 60.1 Hz), 21.6. **^{11}B NMR** (160 MHz, CDCl3) δ -38.84 (d, J = 58.2 Hz). **^{31}P NMR** (202 MHz, CDCl3) δ 19.73 (d, J = 79.6 Hz). These data are in agreement with those reported previously in the literature. [11]

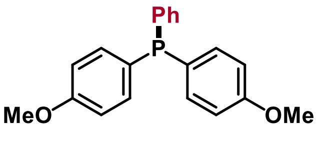

Bis(4-methoxyphenyl)(phenyl)phosphane (22): Following the general procedure I, the title product was obtained after purification by column chromatography (PE/EA = 100:1) as a white solid (26.4 mg, 0.082 mmol, 41%). **^{1}H NMR** (500 MHz, $CDCl_3$) δ 7.57 – 7.45 (m, 7H), 7.44 – 7.38 (m, 2H), 6.97 – 6.93 (m, 4H), 3.83 (s, 6H). **^{13}C NMR** (126 MHz, $CDCl_3$) δ 162.03, 134.94 (d, *J* = 10.9 Hz), 132.99 (d, *J* = 9.7 Hz), 131.02, 130.50 (d, *J* = 58.5 Hz), 128.75 (d, *J* = 10.2 Hz), 120.27 (d, *J* = 63.2 Hz), 114.50 (d, *J* = 11.1 Hz), 55.43. **^{31}P NMR** (202 MHz, $CDCl_3$) δ 18.37 (d, *J* = 75.5 Hz). These data are in agreement with those reported previously in the literature.[12]

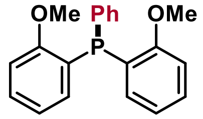

Bis(2-methoxyphenyl)(phenyl)phosphane (23): Following the general procedure I, the title product was obtained after purification by column chromatography (PE/EA = 100:1) as a white solid (29.0 mg, 0.090 mmol, 45%). **^{1}H NMR** (400 MHz, $CDCl_3$): δ = 7.36 – 7.27 (m, 7H), 6.91 – 6.82 (m, 4H), 6.69 – 6.63 (m, 2H), 3.74 (s, 6H). **^{13}C NMR** (151 MHz, $CDCl_3$) δ 161.4 (d, *J* = 15.8 Hz), 134.1 (d, *J* = 20.9 Hz), 133.8, 130.2, 128.6, 128.3 (d, *J* = 7.3 Hz), 125.2, 125.1, 121.0, 110.3, 55.8. **^{31}P NMR** (162 MHz, Chloroform-*d*) δ 27.6. These data are in agreement with those reported previously in the literature.[13]

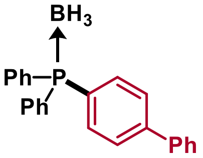

[1,1'-Biphenyl]-4-yldiphenylphosphane borane adduct(24): Following the general procedure I, the title product was obtained after purification by column chromatography (PE/EA = 100:1) as a white solid (53.4 mg, 0.158 mmol, 79%). **^{1}H NMR** (500 MHz, $CDCl_3$) δ 7.61 – 7.53 (m, 4H), 7.43 (t, *J* = 7.6 Hz, 2H), 7.40 – 7.32 (m, 13H). **^{13}C NMR** (126 MHz, $CDCl_3$) δ 141.6, 140.7, 137.3 (d, *J* = 10.8 Hz), 136.2 (d, *J* = 10.8 Hz), 134.3 (d, *J* = 19.5 Hz), 133.9 (d, *J* = 19.5 Hz), 128.9 (d, *J* = 7.2 Hz), 128.7 (d, *J* = 7.1 Hz), 127.7, 127.3 (d, *J* = 7.0 Hz), 127.2. **^{11}B NMR** (160 MHz, $CDCl_3$) δ -38.41 (d, *J* = 58.8 Hz). **^{31}P NMR** (202 MHz, $CDCl_3$) δ -5.47. **MP**: 95 – 96 °C. **IR** (ATR): 3045, 2966, 2296, 1477, 1255, 925, 784, 704, cm^{-1}. **HRMS** (ESI): *m/z* $[M+H]^+$ calcd for $C_{24}H_{23}BP^+$: 353.1625; found: 353.1617.

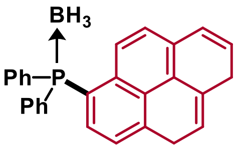

(4,6-Dihydropyren-1-yl)diphenylphosphane borane adduct (25): Following the general procedure I, the title product was obtained after purification by column chromatography (PE/EA = 100:1) as a white solid (41.8 mg, 0.104 mmol, 52%). **^{1}H NMR** (500 MHz, $CDCl_3$) δ 8.41 (d, *J* = 9.3 Hz, 1H), 8.21 (d, *J* = 7.6 Hz, 1H), 8.19 - 8.12 (m, 2H), 8.10 - 8.07 (m, 1H), 8.05 – 8.01 (m, 2H), 7.98 (d, *J* = 9.3 Hz, 1H), 7.78 – 7.74 (m, 1H), 7.72 – 7.67 (m, 4H), 7.55 – 7.50 (m, 2H), 7.47 – 7.42 (m, 4H). **^{13}C NMR** (126 MHz, $CDCl_3$) δ 133.93, 133.56 (d, *J* = 9.5 Hz), 133.42, 132.03 (d, *J* = 8.5 Hz), 131.42 (d, *J* = 2.4 Hz), 131.15, 130.42, 129.92, 129.66, 129.46, 129.07 (d, *J* = 10.2 Hz), 128.27, 127.23, 126.55 (d, *J* = 11.9 Hz), 126.53, 126.27, 125.28 (d, *J* = 8.7 Hz), 124.38 (d, *J* = 10.5 Hz), 124.26, 122.09 (d, *J* = 55.2 Hz). **^{11}B NMR** (160 MHz, $CDCl_3$) δ -36.50. **^{31}P NMR** (202 MHz, $CDCl_3$) δ 21.46. **MP**: 126- 127 °C. **IR** (ATR): 3042, 2975, 2296, 1435, 1265, 925, 756, 704, cm^{-1}. **HRMS** (ESI): *m/z* $[M+H]^+$ calcd for $C_{28}H_{25}BP^+$: 403.1781; found: 403.1779.

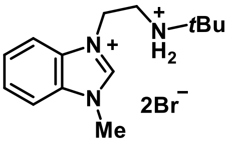

3-(2-(tert-butylamino)ethyl)-1-methyl-1H-benzo[d]imidazol-3-ium

bromide, hydrobromide (NHC B):

Following the general procedure II, the title product was obtained as a white solid. **^{1}H NMR** (500 MHz, DMSO-d_6) δ 9.88 (s, 1H), 9.05 (s, 2H), 8.25 – 8.18 (m, 1H), 8.11 – 8.03 (m, 1H), 7.79 – 7.71 (m, 2H), 4.95 (t, *J* = 6.5 Hz, 2H), 4.13 (s, 3H), 3.59 – 3.50 (m, 2H), 1.35 (s, 9H).**^{13}C NMR** (126 MHz, DMSO-d_6) δ 144.0, 132.4, 131.5, 127.2, 127.1, 114.2, 114.1, 58.0, 43.5, 40.0, 34.0, 25.7. **MP** = 272 – 273°C. **IR** (ATR): 2750, 2542, 2412, 1577, 1444, 1338, 1228, 1195, 1143, 1022, 900, cm^{-1}. **HRMS** (ESI): *m/z* [M]$^+$ calcd for $C_{14}H_{22}N_3$: 232.1808; found: 232.1804.

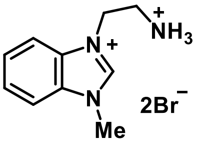

3-(2-(tert-butylamino)ethyl)-1-methyl-1H-benzo[d]imidazol-3-ium bromide, hydrobromide (NHC C):

Following the general procedure II, the title product was obtained as a white solid. **^{1}H NMR** (400 MHz, DMSO-d_6) δ 9.86 (s, 1H), 8.27 – 8.04 (m, 5H), 7.77 – 7.68 (m, 2H), 4.84 (d, *J* = 7.3 Hz, 2H), 4.11 (s, 3H), 3.45 (s, 2H). **^{13}C NMR** (101 MHz, DMSO-d_6) δ 144.1, 132.5, 131.5, 127.1, 114.2, 114.1, 44.6, 38.4, 34.0. **MP** = 265 – 266°C. **IR** (ATR): 3745, 2972, 2096, 1991, 1541, 1395, 1066, 75 cm^{-1}. **HRMS** (ESI): *m/z* [M]$^+$ calcd for $C_{10}H_{14}N_3$: 176.1182; found: 76.1178.

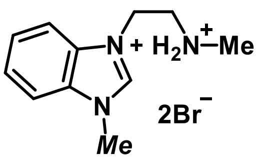

1-methyl-3-(2-(methylammonio)ethyl)-1H-benzo[d]imidazol-3-iu m, hydrobromide (NHC D):

Following the general procedure II, the title product was obtained as a white solid. **^{1}H NMR** (500 MHz, DMSO-d_6) δ 9.90 (s, 1H), 8.91 (s, 2H), 8.21 – 8.15 (m, 1H), 8.09 – 8.04 (m, 1H), 7.75 – .7.69 (m, 2H), 4.92 (t, *J* = 5.8 Hz, 2H), 4.11 (s, 3H), 3.57 (t, *J* = 5.8 Hz, 2H), 2.63 (s, 3H). **^{13}C NMR** (101 MHz, DMSO-d_6) δ 144.1, 132.5, 131.4, 127.1, 114.2.1, 114.19, 47.0, 43.5, 33.9, 32.9. **MP** = 225 – 226°C. **HRMS** (ESI): *m/z* [M]$^+$ calcd for $C_{11}H_{16}N_3^+$: 190.1338; found: 190.1337.

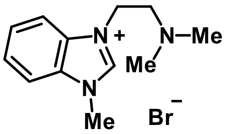

3-(2-(dimethylamino)ethyl)-1-methyl-1H-benzo[d]imidazol-3-ium bromide, hydrobromide (NHC E):

Following the general procedure II, the title product was obtained as a white solid. **^{1}H NMR** (500 MHz, DMSO-d_6) δ 9.95 (s, 1H), 8.18 – 8.14 (m, 1H), 8.04 – 8.00 (m, 1H), 7.71 – 7.66 (m, 2H), 4.97 (t, *J* = 6.2 Hz, 2H), 4.06 (s, 3H), 3.73 (t, *J* = 6.2 Hz, 2H), 2.88 (s, 6H). **^{13}C NMR** (126 MHz, DMSO-d_6) δ 144.2, 132.5, 131.3, 127.2, 127.1, 114.3, 114.2, 54.8, 43.1, 42.0, 34.0. **MP** = 77 – 78°C. **IR** (ATR): 2994, 2890, 2380, 1740, 1620, 1582, 1558, 1460,1180 cm^{-1}. **HRMS** (ESI): *m/z* [M]$^+$ calcd for $C_{12}H_{16}N_3^+$: 202.1338; found: 202.1336.

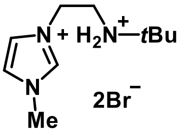

3-(2-(tert-butylamino)ethyl)-1-methyl-1H-imidazol-3-ium bromide, hydrobromide (NHC F):

Following the general procedure II, the title product was obtained as a white solid. **^{1}H NMR** (500 MHz, DMSO-d_6) δ 9.25 (s, 1H), 8.96 (s, 2H), 7.88 – 7.85 (m, 1H), 7.74 – 7.71 (m, 1H), 4.57 (t, *J* = 6.2 Hz, 2H), 3.82 (s, 3H), 3.45 (s, 2H), 1.29 (s, 9H). **^{13}C NMR** (126 MHz, DMSO-d_6) δ 137.9, 124.2, 123.1, 58.0, 45.8, 40.7, 36.4, 25.6. **IR** (ATR): 2760, 2416, 1565, 1452, 1379, 1918, 904, 862, cm^{-1}. **MP** = 268 – 269°C. **HRMS** (ESI): *m/z* [M]$^+$ calcd for $C_{10}H_{20}N_3^+$: 183.1685; found: 183.1682.

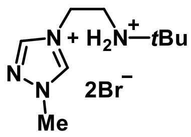

4-(2-(tert-butylamino)ethyl)-1-methyl-1H-1,2,4-triazol-4-ium bromide, hydrobromide (NHC G):
Following the general procedure II, the title product was obtained as a white solid. **^{1}H NMR** (500 MHz, DMSO-d_6) δ 10.20 (s, 1H), 9.25 (s, 1H), 8.92 (s, 2H), 4.64 (t, *J* = 6.2 Hz, 2H), 4.06 (s, 3H), 3.55 – 3.47 (m, 2H), 1.29 (s, 9H). **^{13}C NMR** (126 MHz, DMSO-d_6) δ 145.2, 144.0, 58.1, 44.5, 40.3, 39.3, 25.6. **MP** = 283 – 284°C. **IR** (ATR): 2780, 1586, 1456, 1379, 1185, 877, cm^{-1}. **HRMS** (ESI): *m/z* [M]$^+$ calcd for $C_9H_{19}N_4^+$: 183.1604; found: 183.1602.

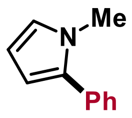

1- methyl-2-phenyl-1H-pyrrole (28): The title product was obtained after purification by column chromatography (petroleum ether : EtOAc = 50:1) as a white solid. **Yield:** (9.7 mg, 0.062 mmol, 31%). **^{1}H NMR** (500 MHz, $CDCl_3$) δ 7.45 – 7.38 (m, 4H), 7.32 – 7.28 (m, 1H), 6.73 – 6.71 (m, 1H), 6.25 – 6.19 (m, 2H), 3.67 (d, *J* = 1.2 Hz, 3H). **^{13}C NMR** (126 MHz, Chloroform-d) δ 134.8, 133.5, 128.8, 128.5, 126.9, 123.8, 108.8, 107.9, 35.2. These data are in agreement with those reported previously in the literature. [14]

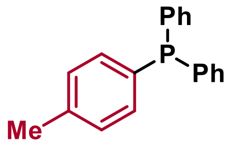

Diphenyl(p-tolyl)phosphane. Yield: 53.7 mg, 0.184 mmol, 92%. **^{1}H NMR** (400 MHz, Chloroform-*d*) δ 7.36 – 7.30 (m, 10H), 7.28 – 7.21 (m, 2H), 7.19 – 7.15 (m, 2H), 2.37 (s, 3H). **^{13}C NMR** (151 MHz, Chloroform-*d*) δ 139.0, 137.6 (d, *J* = 10.4 Hz), 134.1 (d, *J* = 19.9 Hz), 133.7 (d, *J* = 19.3 Hz), 133.6 (d, *J* = 9.1 Hz), 129.5 (d, *J* = 7.3 Hz), 128.7, 128.6 (d, *J* = 6.8 Hz), 21.4. **^{31}P NMR** (162 MHz, Chloroform-*d*) δ -5.63. These data are in agreement with those reported previously in the literature.[15]

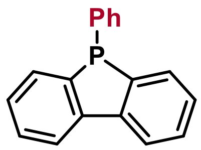

5-phenyl-5H-benzo[b]phosphindole. Yield: 46.3 mg, 0.178 mmol, 89%. **^{1}H NMR** (400 MHz, Chloroform-*d*) δ 7.95 (dd, *J* = 7.7, 1.6 Hz, 2H), 7.74 – 7.66 (m, 2H), 7.49 – 7.44 (m, 2H), 7.35 – 7.20 (m, 7H). **^{13}C NMR** (151 MHz, Chloroform-*d*) δ 143.8 (d, *J* = 2.9 Hz), 142.7 (d, *J* = 2.6 Hz), 136.3 (d, *J* = 18.9 Hz), 132.8 (d, *J* = 20.1 Hz), 130.6 (d, *J* = 21.9 Hz), 129.4, 128.8, 128.8 (d, *J* = 7.8 Hz), 127.7 (d, *J* = 7.6 Hz), 121.5. **^{31}P NMR** (162 MHz, Chloroform-*d*) δ -9.51. These data are in agreement with those reported previously in the literature.[16]

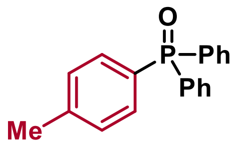

diphenyl(p-tolyl)phosphine oxide. Yield: 54.9 mg, 0.188 mmol, 94%. **^{1}H NMR** (400 MHz, Chloroform-*d*) δ 7.66 – 7.61 (m, 4H), 7.57 – 7.47 (m, 4H), 7.44 – 7.40 (m, 4H), 7.24 (d, *J* = 5.6 Hz, 2H), 2.37 (s, 3H). **^{13}C NMR** (101 MHz, Chloroform-*d*) δ 142.6 (d, *J* = 2.9 Hz), 133.4, 132.4, 132.2 (d, *J* = 10.1 Hz), 132.1 (d, *J* = 10.0 Hz), 131.9 (d, *J* = 2.8 Hz), 129.7, 129.4 (d, *J* = 12.6 Hz), 128.7, 128.5 (d, *J* = 12.0 Hz), 21.7. **^{31}P NMR** (162 MHz, Chloroform-*d*) δ 29.87. These data are in agreement with those reported previously in the literature.[17]

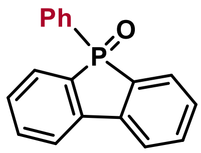

5-phenylbenzo[b]phosphindole 5-oxide. Yield: 48.6 mg, 0.176 mmol, 88%. **^{1}H NMR** (600 MHz, Chloroform-*d*) δ 7.83 (dd, *J* = 7.8, 2.9 Hz, 2H), 7.76 – 7.70 (m, 2H), 7.67 – 7.64 (m, 2H), 7.62 – 7.57 (m, 2H), 7.51 – 7.48 (m, 1H), 7.41 – 7.37 (m, 4H). **^{13}C NMR** (151 MHz, Chloroform-*d*) δ 141.9 (d, *J* = 21.9 Hz), 133.6

(d, J = 2.2 Hz), 133.3, 132.5, 132.4 (d, J = 2.8 Hz), 131.2 (d, J = 10.9 Hz), 130.5, 130.1 (d, J = 9.7 Hz), 129.6 (d, J = 11.2 Hz), 128.9 (d, J = 12.6 Hz), 121.3 (d, J = 10.1 Hz). **^{31}P NMR** (243 MHz, Chloroform-*d*) δ 34.65. These data are in agreement with those reported previously in the literature.[18]

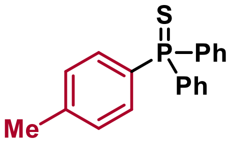

diphenyl(p-tolyl)phosphine sulfide. Yield: 49.3 mg, 0.160 mmol, 80%. **^{1}H NMR** (400 MHz, Chloroform-*d*) δ 7.76 – 7.66 (m, 4H), 7.63 – 7.57 (m, 2H), 7.53 – 7.47 (m, 2H), 7.45 – 7.40 (m, 4H), 7.28 – 7.20 (m, 2H), 2.39 (s, 3H). **^{13}C NMR** (101 MHz, Chloroform-*d*) δ 142.2 (d, J = 3.0 Hz), 133.7, 132.9, 132.5, 132.3 (d, J = 10.3 Hz), 131.6 (d, J = 3.0 Hz), 130.0, 129.4 (d, J = 13.0 Hz), 129.1, 128.6 (d, J = 12.5 Hz), 21.6. **^{31}P NMR** (162 MHz, Chloroform-*d*) δ 43.64. These data are in agreement with those reported previously in the literature.[19]

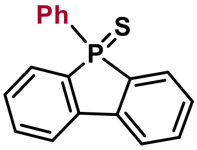

5-phenylbenzo[b]phosphindole 5-sulfide. Yield: 45.6 mg, 0.156 mmol, 78%. **^{1}H NMR** (400 MHz, Chloroform-*d*) δ 7.86 (dd, J = 7.8, 3.1 Hz, 2H), 7.76 – 7.64 (m, 4H), 7.61 – 7.53 (m, 2H), 7.46 – 7.33 (m, 5H). **^{13}C NMR** (101 MHz, Chloroform-*d*) δ 141.50 (d, J = 18.7 Hz), 136.5, 135.6, 132.9 (d, J = 2.4 Hz), 132.0 (d, J = 3.2 Hz), 131.6, 131.0 (d, J = 11.9 Hz), 130.8, 129.9 (d, J = 11.9 Hz), 129.6 (d, J = 10.9 Hz), 128.7 (d, J = 13.0 Hz), 121.5 (d, J = 9.4 Hz). **^{31}P NMR** (162 MHz, Chloroform-*d*) δ 41.36. These data are in agreement with those reported previously in the literature.[20]

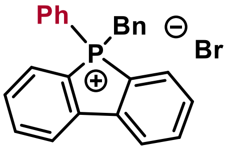

5-benzyl-5-phenyl-5H-benzo[b]phosphindol-5-ium bromide. Yield: 78.3 mg, 0.182 mmol, 91%. **^{1}H NMR** (600 MHz, DMSO-d_6) δ 8.53 – 8.45 (m, 2H), 8.11 (dd, J = 7.9, 3.1 Hz, 2H), 8.09 – 8.02 (m, 2H), 7.86 – 7.79 (m, 3H), 7.73 – 7.67 (m, 4H), 7.06 – 6.98 (m, 3H), 6.96 – 6.91 (m, 2H), 5.23 (d, J = 17.3 Hz, 1H). **^{13}C NMR** (101 MHz, DMSO-d_6) δ 144.7 (d, J = 17.2 Hz), 136.7, 135.8 (d, J = 3.4 Hz), 133.5 (d, J = 10.2 Hz), 133.1 (d, J = 11.0 Hz), 131.2 (d, J = 11.8 Hz), 130.9 (d, J = 12.9 Hz), 130.5 (d, J = 6.0 Hz), 128.9 (d, J = 3.8 Hz), 128.5 (d, J = 4.4 Hz), 127.8 (d, J = 9.0 Hz), 124.0 (d, J = 9.1 Hz), 120.6, 119.7, 119.1, 118.2, 27.7 (d, J = 41.3 Hz). **^{31}P NMR** (243 MHz, DMSO-d_6) δ 30.02. These data are in agreement with those reported previously in the literature.[21]

6. Computational details

All the DFT calculations were performed by using Gaussian 16 program. [22] Geometry optimizations were carried out at the B3LYP[23]-D3BJ[24]/6-31G(d,p) level in N, N-dimethylacetamide (DMA) solvent with the implicit solvent model SMD.[25] The followed frequency analyses were calculated at the same theoretical level to verify that the nature of stationary points (minima without imaginary frequency and transition states having unique one imaginary frequency) and to obtain the thermal Gibbs free energy corrections at 298.15 K. The free energies were further refined by single-point calculations at B3LYP-D3BJ/6-311++G(d,p) level with SMD solvent model. The standard state change from 1 atm to 1 M were corrected by adding 1.89 kcal/mol to the above free energy. With the optimized structures of ground state, the vertical excitation energies were calculated at TD[26]-B3LYP-D3BJ/6-311++G(d,p) level with SMD solvent model. The selected calculated geometries were visualized by the CYLview[27] program.

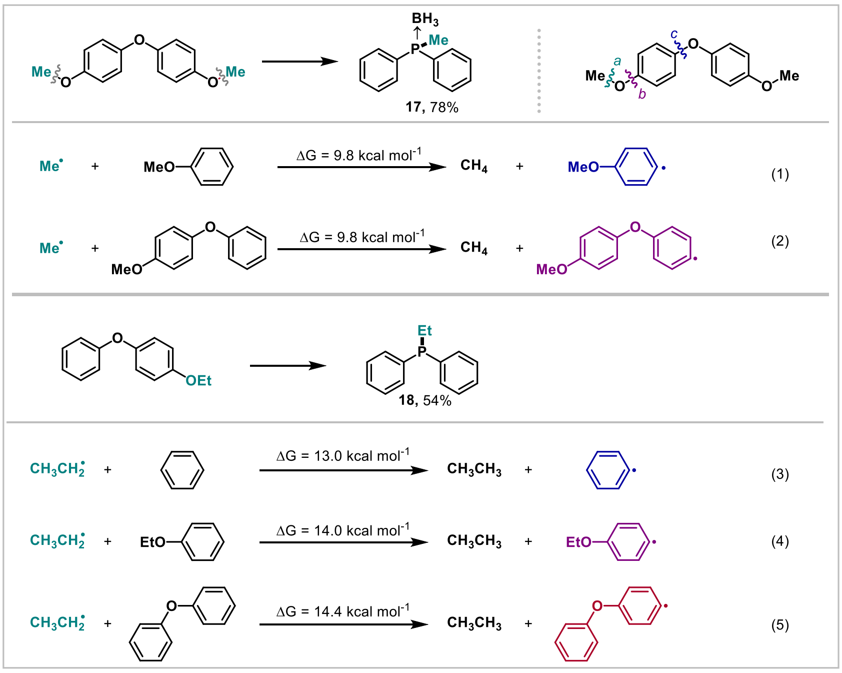

Figure S10. DFT calculations on the stability of alkyl radicals and aryl radicals in the reaction system.

Due to the complex catalytic system, we are not able to pinpoint the exact reasons for the preferred productions of **17** and **18**. Nevertheless, we assumed that the stability of the *in-situ* generated radicals could be one of the causes. As shown above, the DFT calculations show that the alkyl radicals are more stable than aryl radicals.

Cartesian Coordinates in Å, SCF Energies and Free Energies (in a.u.) at 298.15 K for the Optimized Structures. [BS1=6-31G(d,p), BS2=6-311++G(d,p)]

NHC -710.4883012 a.u.

B3LYP-D3BJ/BS1 SCF energy in DMA: B3LYP-D3BJ/BS2 SCF energy in DMA:

-710.6518919 a.u.
B3LYP-D3BJ/BS2 free energy in DMA:
-710.3611090 a.u.

N	1.918138	-0.012895	-0.244946
N	-0.819845	1.037233	-0.830228
N	-2.566308	1.297048	0.403082
C	3.413899	0.781662	1.618866
H	4.330440	0.550151	2.172292
H	2.574943	0.745193	2.323519
H	3.502113	1.807624	1.248251
C	3.200718	-0.221018	0.469524
C	1.658866	1.324320	-0.774292
H	2.482366	1.616738	-1.433235
H	1.574031	2.101120	0.002640
C	0.380113	1.340804	-1.609670
H	0.239816	2.333282	-2.041691
H	0.453170	0.614993	-2.424128
C	-1.317596	-0.249713	-0.608399
C	-2.459121	-0.075933	0.192723
C	-3.231613	-1.158912	0.612507
H	-4.112037	-1.019870	1.230590
C	-2.820697	-2.428434	0.203286
H	-3.394235	-3.297602	0.510043
C	-1.573204	2.005571	-0.217597
C	-0.902928	-1.520004	-1.012086
H	-0.004868	-1.657109	-1.602155
C	-1.675457	-2.604661	-0.593843
H	-1.381798	-3.607812	-0.887133
C	3.135257	-1.643910	1.039743
H	2.977564	-2.372765	0.238024
H	2.306917	-1.738739	1.752069
H	4.061354	-1.898439	1.564251
C	4.360671	-0.126678	-0.530849
H	5.303264	-0.397899	-0.044755
H	4.477006	0.886229	-0.928577
H	4.196936	-0.808199	-1.372099
C	-3.632250	1.901848	1.183633
H	-3.613781	1.528152	2.211991
H	-4.607872	1.673836	0.743262
H	-3.481901	2.980875	1.189048
H	1.172733	-0.239113	0.411169

***t*BuOK**

B3LYP-D3BJ/BS1 SCF energy in DMA:
-833.0356451 a.u.
B3LYP-D3BJ/BS2 SCF energy in DMA:
-833.1494766 a.u.
B3LYP- D3BJ/BS2 free energy in DMA:
-833.0582197 a.u.

O	-0.277998	-0.030193	-0.145810
C	1.078944	-0.001280	-0.007325
C	1.554959	1.406271	0.441859
C	1.545053	-1.031490	1.056281
C	1.776931	-0.343796	-1.350790
H	1.081176	1.665760	1.397048
H	2.643809	1.488409	0.567987
H	1.240008	2.153638	-0.297095
H	1.223480	-2.037240	0.758216
H	1.071161	-0.804239	2.019445
H	2.633514	-1.052449	1.208333
H	1.467503	0.375301	-2.119450
H	2.874650	-0.333815	-1.295834
H	1.461078	-1.340104	-1.684485
K	-2.589880	-0.002734	-0.011991

Ph_2O

B3LYP-D3BJ/BS1 SCF energy in DMA:
-538.5867094 a.u.
B3LYP-D3BJ/BS2 SCF energy in DMA:
-538.7123629 a.u.
B3LYP-D3BJ/BS2 free energy in DMA:
-538.5618000 a.u.

O	0.000005	-1.216555	-0.000026
C	1.193378	-0.521433	0.041534
C	1.361463	0.620345	0.830146
C	2.260028	-1.054117	-0.682939
C	2.613286	1.233550	0.878174
H	0.526519	1.019495	1.395464
C	3.509012	-0.435529	-0.618110
H	2.100225	-1.942721	-1.284645
C	3.690337	0.711416	0.157485
H	2.746390	2.121012	1.489726
H	4.339165	-0.850385	-1.181904
H	4.661689	1.193703	0.201707
C	-1.193377	-0.521445	-0.041553

C	-2.259995	-1.054103	0.682975
C	-1.361494	0.620309	-0.830203
C	-3.508986	-0.435514	0.618172
H	-2.100179	-1.942686	1.284709
C	-2.613315	1.233509	-0.878206
H	-0.526567	1.019433	-1.395564
C	-3.690342	0.711398	-0.157453
H	-4.339112	-0.850358	1.182016
H	-2.746452	2.120948	-1.489784
H	-4.661691	1.193691	-0.201660

Ph_2PK

B3LYP-D3BJ/BS1 SCF energy in DMA:
-1404.668917 a.u.
B3LYP-D3BJ/BS2 SCF energy in DMA:
-1404.837447 a.u.
B3LYP-D3BJ/BS2 free energy in DMA:
-1404.6944179 a.u.

P	-0.072347	0.416906	1.630943
C	1.320162	-0.289104	0.662139
C	2.623593	-0.153434	1.209971
C	1.246888	-0.841917	-0.642191
C	3.762404	-0.519337	0.500863
H	2.734026	0.261719	2.209906
C	2.394690	-1.209829	-1.351459
H	0.278750	-0.991982	-1.111141
C	3.663660	-1.049586	-0.793037
H	4.739327	-0.393288	0.961175
H	2.287225	-1.624921	-2.350779
H	4.553884	-1.336261	-1.344107
C	-1.571838	-0.088719	0.671201
C	-2.578149	0.866564	0.407802
C	-1.823679	-1.412544	0.247687
C	-3.764355	0.522857	-0.245769
H	-2.427613	1.896602	0.726063
C	-2.996063	-1.753490	-0.425346
H	-1.089948	-2.186273	0.454890
C	-3.976954	-0.788253	-0.676042
H	-4.517184	1.285371	-0.428258
H	-3.152920	-2.781642	-0.741503
H	-4.893104	-1.056455	-1.193736
K	0.672137	2.156542	-1.002188

NHC...*t*BuOK (I)

B3LYP-D3BJ/BS1 SCF energy in DMA:
-1543.566008 a.u.
B3LYP-D3BJ/BS2 SCF energy in DMA:
-1543.827131 a.u.
B3LYP-D3BJ/BS2 free energy in DMA:
-1543.4246014 a.u.

N	1.366447	1.806043	0.364676
H	1.195598	1.637611	1.355755
N	2.482599	-0.918808	0.218694
N	1.751502	-2.492277	-1.059234
C	0.923107	3.443249	-1.440045
H	0.552563	4.450213	-1.654150
H	0.303089	2.747982	-2.017841
H	1.948466	3.380997	-1.815935
C	0.845368	3.171089	0.069023
C	2.790133	1.565958	0.106151
H	3.452014	2.338465	0.524212
H	2.961302	1.547590	-0.975282
C	3.245654	0.234098	0.700598
H	4.295874	0.071740	0.452619
H	3.159359	0.256814	1.789837
C	1.418811	-1.501120	0.911769
C	0.947297	-2.530138	0.080716
C	-0.153598	-3.313993	0.430431
H	-0.516397	-4.101980	-0.220000
C	-0.766319	-3.030574	1.650251
H	-1.626562	-3.614216	1.961349
C	2.712044	-1.519978	-0.989442
C	0.800278	-1.213931	2.130308
H	1.149658	-0.410816	2.769178
C	-0.296383	-1.998546	2.484637
H	-0.803627	-1.805209	3.424310
C	-0.621187	3.177851	0.525607
H	-0.680348	3.010381	1.608393
H	-1.215094	2.398303	0.030254
H	-1.077548	4.151115	0.316075
C	1.619384	4.266796	0.825130
H	1.138834	5.240309	0.683801
H	2.652488	4.357213	0.476091
H	1.638035	4.051503	1.899630
C	1.600804	-3.421843	-2.170214
H	0.586402	-3.366709	-2.576611

H	1.792347	-4.445954	-1.837557
H	2.317190	-3.150133	-2.944064
O	-2.372685	0.463614	-0.811435
C	-3.577086	0.202547	-0.218669
C	-4.684537	1.134413	-0.777872
C	-4.006938	-1.267611	-0.472180
C	-3.493908	0.416391	1.317047
H	-4.769776	0.995144	-1.862812
H	-5.674459	0.961121	-0.332636
H	-4.410075	2.181446	-0.599373
H	-3.248829	-1.946963	-0.063401
H	-4.070709	-1.450609	-1.552220
H	-4.976402	-1.529800	-0.024934
H	-3.221098	1.456019	1.535123
H	-4.433452	0.195657	1.843480
H	-2.710357	-0.227464	1.734514
K	-0.092021	0.066246	-1.320251

NHC...*t*BuOK...Ph$_2$O (II)

B3LYP-D3BJ/BS1 SCF energy in DMA:
-2082.173637 a.u.

B3LYP-D3BJ/BS2 SCF energy in DMA:
-2082.554726 a.u.

B3LYP-D3BJ/BS2 free energy in DMA:
-2081.9814121 a.u.

N	-0.219995	-0.882459	1.738235
H	-0.826336	-0.268701	1.194576
N	0.230985	-2.686501	-0.592659
N	2.147232	-3.252966	-1.400683
C	0.848668	-1.102195	3.968331
H	0.942501	-0.636906	4.953973
H	1.858903	-1.150707	3.545815
H	0.494483	-2.125892	4.118288
C	-0.111832	-0.277602	3.099246
C	-0.770810	-2.241090	1.663351
H	-1.738514	-2.352557	2.173163
H	-0.073761	-2.936739	2.140830
C	-0.989158	-2.686831	0.217872
H	-1.394055	-3.700206	0.217566
H	-1.715176	-2.040141	-0.276837
C	0.586962	-1.661244	-1.471125
C	1.833799	-2.032306	-1.999873
C	2.533051	-1.207409	-2.883574
H	3.499358	-1.495938	-3.281997
C	1.934641	0.006704	-3.220308
H	2.445317	0.680182	-3.900907
C	1.170053	-3.680746	-0.542919
C	-0.007980	-0.443902	-1.805379
H	-0.959067	-0.146836	-1.386315
C	0.685710	0.381128	-2.688884
H	0.255641	1.339139	-2.962092
C	0.448473	1.140040	2.905124
H	-0.251046	1.747162	2.319700
H	1.416889	1.146262	2.387664
H	0.573093	1.629169	3.877403
C	-1.476746	-0.181456	3.806065
H	-1.368065	0.309477	4.778822
H	-1.918764	-1.166591	3.982831
H	-2.174008	0.409771	3.205225
C	3.369841	-3.989526	-1.688186
H	4.246432	-3.382220	-1.442409
H	3.411484	-4.257346	-2.747684
H	3.374866	-4.894746	-1.082900
O	3.393485	1.141727	1.021928
C	3.750669	2.338682	0.465653
C	4.409038	3.268313	1.519076
C	4.760799	2.126685	-0.694188
C	2.507976	3.068167	-0.114270
H	5.296993	2.778899	1.938387
H	4.715160	4.244184	1.116460
H	3.707780	3.445327	2.343918
H	4.304403	1.494277	-1.465984
H	5.649374	1.604675	-0.317496
H	5.091181	3.060594	-1.170963
H	1.778117	3.249975	0.682927
H	2.744516	4.033579	-0.584231
H	2.030095	2.431039	-0.867365
K	2.478785	-1.043558	0.865585
O	-2.748233	1.050634	0.583970
C	-3.489502	-0.003154	0.078917
C	-3.636438	-0.242173	-1.289940
C	-4.029032	-0.882143	1.020369
C	-4.318179	-1.386436	-1.709210
H	-3.219854	0.445561	-2.016169
C	-4.701547	-2.022865	0.585228
H	-3.906686	-0.670856	2.076160
C	-4.847840	-2.282836	-0.779876

H	-4.426486	-1.575745	-2.772898
H	-5.114190	-2.708516	1.319038
H	-5.372366	-3.171911	-1.114685
C	-2.536671	2.177199	-0.201358
C	-1.220844	2.580601	-0.417555
C	-3.612333	2.916614	-0.695119
C	-0.978543	3.747672	-1.145167
H	-0.404934	1.986087	-0.024539
C	-3.356324	4.075407	-1.427853
H	-4.628398	2.584605	-0.509712
C	-2.042309	4.494275	-1.654730
H	0.045345	4.065582	-1.313756
H	-4.188102	4.655091	-1.816326
H	-1.850158	5.398667	-2.223337

NHC...Ph_2PK (III)

B3LYP-D3BJ/BS1 SCF energy in DMA:
-2115.19518 a.u.
B3LYP-D3BJ/BS2 SCF energy in DMA:
-2115.516897 a.u.
B3LYP-D3BJ/BS2 free energy in DMA:
-2115.0637533 a.u.

N	1.961995	2.473663	-0.174112
N	-0.890479	1.611768	0.464934
N	-2.172782	1.345963	-1.251879
C	3.546685	1.244247	-1.702705
H	4.565993	1.241068	-2.102158
H	2.870773	1.301247	-2.566407
H	3.412753	0.296122	-1.171109
C	3.318196	2.450714	-0.768340
C	1.601850	1.383717	0.726488
H	2.327239	1.340020	1.541420
H	1.610219	0.379883	0.273997
C	0.252919	1.622789	1.384608
H	0.084022	0.846554	2.138733
H	0.263963	2.592496	1.891782
C	-2.185686	1.867224	0.913345
C	-3.019568	1.688278	-0.201727
C	-4.404644	1.816291	-0.107877
H	-5.048000	1.663058	-0.967169
C	-4.925817	2.134767	1.146710
H	-5.999730	2.239756	1.264316
C	-0.865350	1.278605	-0.859936
C	-2.704288	2.187382	2.167823
H	-2.062409	2.319196	3.031584
C	-4.090617	2.317860	2.264164
H	-4.534793	2.561968	3.223986
C	3.444194	3.743232	-1.584859
H	3.335970	4.619145	-0.937534
H	2.664611	3.790098	-2.354763
H	4.416245	3.796907	-2.085139
C	4.373847	2.440688	0.345316
H	5.367901	2.606171	-0.081282
H	4.399861	1.485959	0.878578
H	4.173483	3.236440	1.070307
C	-2.653087	0.990690	-2.577561
H	-3.383276	1.727850	-2.920327
H	-3.122965	0.002203	-2.562657
H	-1.812162	0.989874	-3.271964
H	1.281155	2.496646	-0.932192
P	0.800738	-3.418305	-0.580067
C	-0.940279	-2.893588	-0.297044
C	-1.920651	-3.544180	-1.091573
C	-1.410004	-1.862968	0.552378
C	-3.266035	-3.191560	-1.042147
H	-1.609548	-4.342080	-1.763816
C	-2.764394	-1.527054	0.617351
H	-0.712203	-1.304765	1.164260
C	-3.706720	-2.179574	-0.179318
H	-3.978913	-3.717662	-1.672579
H	-3.080498	-0.738821	1.293532
H	-4.757187	-1.909843	-0.128392
C	1.822043	-2.530190	0.671079
C	3.135371	-2.153634	0.297796
C	1.449690	-2.264348	2.009248
C	4.010568	-1.537292	1.192015
H	3.472588	-2.349518	-0.718833
C	2.314417	-1.622614	2.896537
H	0.475849	-2.579659	2.368024
C	3.601786	-1.250325	2.497693
H	5.009298	-1.264583	0.861306
H	1.984562	-1.428551	3.914142
H	4.274197	-0.754825	3.191313
K	0.827487	-0.607499	-2.213650

NHC...*t*BuOK...Ph_2O...Ph_2PK (IV)

B3LYP-D3BJ/BS1 SCF energy in DMA:

-3486.912164 a.u.
B3LYP-D3BJ/BS2 SCF energy in DMA:
-3487.448269 a.u.
B3LYP-D3BJ/BS2 free energy in DMA:
-3486.7086874 a.u.

N	1.275177	0.594501	-1.508065
H	2.160413	0.675225	-1.008623
N	1.262283	-2.410685	-1.261276
N	-0.341949	-3.617996	-0.475955
C	-0.508440	1.957650	-2.563878
H	-0.864894	2.981228	-2.716665
H	-1.270761	1.410171	-1.998773
H	-0.446081	1.486680	-3.548982
C	0.849605	1.984532	-1.848737
C	1.480955	-0.321514	-2.637964
H	2.146565	0.085296	-3.412460
H	0.516963	-0.524271	-3.114696
C	2.096297	-1.649062	-2.194791
H	2.266048	-2.268803	-3.076791
H	3.062463	-1.481594	-1.716800
C	1.447565	-2.429467	0.122602
C	0.401923	-3.215889	0.632767
C	0.225306	-3.406429	2.005176
H	-0.592676	-4.003861	2.392156
C	1.142750	-2.783211	2.851162
H	1.040688	-2.901688	3.925086
C	0.177344	-3.149159	-1.650702
C	2.364626	-1.807259	0.970926
H	3.165575	-1.194288	0.582170
C	2.195412	-1.999167	2.341159
H	2.885442	-1.521065	3.028727
C	0.718049	2.737612	-0.515996
H	1.687372	2.802443	-0.011170
H	0.023818	2.239713	0.171799
H	0.378949	3.764937	-0.695852
C	1.874732	2.718551	-2.731907
H	1.539732	3.742155	-2.930889
H	2.011613	2.224444	-3.698178
H	2.843555	2.771778	-2.227009
C	-1.520360	-4.466732	-0.370873
H	-2.222458	-4.043302	0.350100
H	-1.236998	-5.472193	-0.044936
H	-1.996793	-4.519259	-1.348026
O	-1.653376	1.258210	1.575222
C	-1.365749	1.361697	2.925683
C	-1.955186	2.669354	3.513613
C	-1.965526	0.167657	3.704017
C	0.163848	1.375226	3.158160
H	-3.042801	2.691704	3.362883
H	-1.766400	2.783531	4.588794
H	-1.514888	3.542136	3.011430
H	-1.556797	-0.773161	3.315816
H	-3.052746	0.143399	3.570767
H	-1.753973	0.209846	4.780820
H	0.616909	2.221347	2.630473
H	0.430892	1.453069	4.220442
H	0.610194	0.454294	2.765835
K	-0.946077	-0.524645	-0.052657
O	4.415961	1.387907	-0.475152
C	5.001709	0.362079	-1.195451
C	5.531374	-0.784720	-0.598539
C	4.977999	0.498178	-2.585672
C	6.022110	-1.808360	-1.412472
H	5.554351	-0.883607	0.480027
C	5.467925	-0.534242	-3.383112
H	4.568051	1.400333	-3.024293
C	5.989436	-1.693731	-2.802573
H	6.427313	-2.702242	-0.947948
H	5.442060	-0.428527	-4.463394
H	6.369253	-2.495761	-3.427168
C	4.678073	1.495467	0.886289
C	3.610704	1.374074	1.772794
C	5.966929	1.781404	1.337299
C	3.840549	1.540003	3.140381
H	2.620625	1.149092	1.395855
C	6.185237	1.937472	2.706020
H	6.780069	1.874482	0.625016
C	5.125658	1.817237	3.609701
H	3.011217	1.446932	3.834153
H	7.185674	2.158526	3.065141
H	5.301883	1.941850	4.673468
P	-3.792876	-0.611702	-1.929257
C	-4.486289	1.025989	-1.532950
C	-4.182292	2.097199	-2.419577
C	-5.349283	1.342450	-0.451320
C	-4.701972	3.378181	-2.242575
H	-3.528176	1.908760	-3.267759

C	-5.865322	2.627885	-0.271925
H	-5.626440	0.567128	0.254570
C	-5.547509	3.665601	-1.159048
H	-4.441661	4.161223	-2.950481
H	-6.525368	2.819978	0.570655
H	-5.953098	4.662533	-1.018848
C	-4.253235	-1.672891	-0.485702
C	-3.968488	-1.343394	0.861013
C	-4.794711	-2.952267	-0.728858
C	-4.218304	-2.244647	1.897599
H	-3.524708	-0.379014	1.105355

$Me^{\bullet}$

B3LYP-D3BJ/BS1 SCF energy in DMA:
-39.841548 a.u.
B3LYP-D3BJ/BS2 SCF energy in DMA:
-39.854075 a.u.
B3LYP-D3BJ/BS2 free energy in DMA:
-39.841298 a.u.

C	-0.000006	-0.000002	0.000257
H	0.518385	-0.951668	-0.000514
H	0.565062	0.924730	-0.000514
H	-1.083411	0.026952	-0.000514

MeOPh

B3LYP-D3BJ/BS1 SCF energy in DMA:
-346.817390 a.u.
B3LYP-D3BJ/BS2 SCF energy in DMA:
-346.902711 a.u.
B3LYP-D3BJ/BS2 free energy in DMA:
-346.797374 a.u.

C	2.280992	0.338728	-0.000020
C	1.326832	1.354481	-0.000139
C	1.856565	-0.995017	0.000060
C	-0.040499	1.060021	-0.000176
H	1.639920	2.394726	-0.000210
C	0.500974	-1.305460	0.000037
H	2.586757	-1.799304	0.000153
C	-0.455563	-0.277838	-0.000077
H	-0.762294	1.867531	-0.000290
H	0.158996	-2.335600	0.000109
O	-1.757329	-0.680286	-0.000131

C	-5.035162	-3.858250	0.306048
H	-5.031419	-3.239196	-1.750548
C	-4.752018	-3.508959	1.628310
H	-3.983575	-1.959552	2.919514
H	-5.452441	-4.835800	0.078593
H	-4.942954	-4.209253	2.436271
K	-2.700938	3.071550	0.242296

C	-2.765584	0.328061	0.000313
H	-2.703773	0.960013	-0.893559
H	-3.719794	-0.201120	0.000617
H	-2.703112	0.959878	0.894231
H	3.339630	0.578313	0.000009

CH_4

B3LYP-D3BJ/BS1 SCF energy in DMA:
-40.524615 a.u.
B3LYP-D3BJ/BS2 SCF energy in DMA:
-40.534684 a.u.
B3LYP-D3BJ/BS2 free energy in DMA:
-40.504201 a.u.

C	0.000000	0.000000	0.000000
H	0.631141	0.631141	0.631141
H	-0.631141	0.631141	-0.631141
H	0.631141	-0.631141	-0.631141
H	-0.631141	-0.631141	0.631141

$MeOPh^{\bullet}$

B3LYP-D3BJ/BS1 SCF energy in DMA:
-346.125098 a.u.
B3LYP-D3BJ/BS2 SCF energy in DMA:
-346.210506 a.u.
B3LYP-D3BJ/BS2 free energy in DMA:
-346.118805 a.u.

C	2.272588	0.384375	0.000170
C	1.361463	1.414202	0.000002
C	1.949587	-0.958448	0.000150
C	-0.003727	1.076282	-0.000210
H	1.659733	2.458711	-0.000168

C	0.590994	-1.286903	0.000002
H	2.702531	-1.741346	0.000463
C	-0.383150	-0.273546	-0.000233
H	-0.745157	1.866371	-0.000535
H	0.268303	-2.323722	0.000161
O	-1.675911	-0.704734	-0.000509
C	-2.706575	0.280813	0.000457
H	-2.659152	0.913843	-0.893577
H	-3.648282	-0.270155	0.001057
H	-2.657768	0.913516	0.894643

4-MeO-PhOPh

B3LYP-D3BJ/BS1 SCF energy in DMA:
-653.118926 a.u.
B3LYP-D3BJ/BS2 SCF energy in DMA:
-653.276339 a.u.
B3LYP-D3BJ/BS2 free energy in DMA:
-653.096497 a.u.

O	-1.003569	-1.321587	0.119847
C	0.272034	-0.772517	0.154912
C	0.681324	0.009757	1.239187
C	1.159750	-1.074607	-0.869717
C	1.983246	0.489647	1.285861
H	-0.019044	0.238137	2.035869
C	2.475022	-0.601986	-0.822885
H	0.825629	-1.684025	-1.702973
C	2.890029	0.186903	0.256517
H	2.321158	1.097909	2.118439
H	3.154522	-0.850802	-1.628312
C	-2.086545	-0.480583	-0.008740
C	-3.332889	-1.033369	0.302539
C	-1.987412	0.839403	-0.458007
C	-4.482833	-0.259080	0.164991
H	-3.382066	-2.060784	0.648327
C	-3.149197	1.603592	-0.586066
H	-1.021041	1.262714	-0.705071
C	-4.398668	1.064331	-0.277232
H	-5.448270	-0.692472	0.409129
H	-3.069968	2.628985	-0.935319
O	4.142239	0.702413	0.399820
C	5.099488	0.412016	-0.616896
H	4.783578	0.804299	-1.590740
H	6.021922	0.908227	-0.311616
H	5.280140	-0.666077	-0.702568
H	-5.296018	1.666244	-0.379294

4-MeO-PhOPh•

B3LYP-D3BJ/BS1 SCF energy in DMA:
-652.426579 a.u.
B3LYP-D3BJ/BS2 SCF energy in DMA:
-652.584036 a.u.
B3LYP-D3BJ/BS2 free energy in DMA:
-652.417932 a.u.

O	-1.066431	-1.294938	0.102078
C	0.210647	-0.749953	0.142587
C	0.588594	0.116066	1.173770
C	1.131783	-1.141556	-0.820370
C	1.892471	0.589427	1.227998
H	-0.136782	0.414705	1.923404
C	2.449153	-0.675666	-0.763967
H	0.821580	-1.814592	-1.612881
C	2.832925	0.196746	0.261160
H	2.205901	1.261830	2.019821
H	3.154712	-0.994421	-1.520851
C	-2.145196	-0.447265	-0.026836
C	-3.376395	-0.953788	0.404691
C	-2.045151	0.827131	-0.596528
C	-4.534420	-0.177675	0.267927
H	-3.416873	-1.946731	0.841550
C	-3.199238	1.614067	-0.730386
H	-1.085940	1.203807	-0.933523
C	-4.388291	1.073703	-0.292811
H	-5.494093	-0.561749	0.601029
H	-3.133873	2.605124	-1.169906
O	4.084246	0.713894	0.407249
C	5.070324	0.344564	-0.554770
H	4.784896	0.661336	-1.564911
H	5.985176	0.861377	-0.261056
H	5.249544	-0.737136	-0.552089

$CH_3CH_2^•$

B3LYP-D3BJ/BS1 SCF energy in DMA:
-79.170042 a.u.
B3LYP-D3BJ/BS2 SCF energy in DMA:
-79.190190 a.u.
B3LYP-D3BJ/BS2 free energy in DMA:

-79.152061 a.u.

C	0.793685	0.000000	-0.024090
C	-0.692607	0.000000	-0.001476
H	1.351248	0.928956	0.052672
H	1.351246	-0.928958	0.052671
H	-1.090328	-0.000029	1.029368
H	-1.109315	0.888567	-0.490634
H	-1.109316	-0.888539	-0.490682

PhH

B3LYP-D3BJ/BS1 SCF energy in DMA:
-232.283890 a.u.
B3LYP-D3BJ/BS2 SCF energy in DMA:
-232.337516 a.u.
B3LYP-D3BJ/BS2 free energy in DMA:
-232.261308 a.u.

C	1.397053	-0.008030	0.000061
C	0.691555	-1.213878	0.000039
C	0.705461	1.205851	0.000024
C	-0.705491	-1.205909	-0.000024
H	1.229561	-2.157522	0.000069
C	-0.691580	1.213939	-0.000038
H	1.254285	2.143245	0.000042
C	-1.396981	0.008028	-0.000062
H	-1.254358	-2.143214	-0.000041
H	-1.229641	2.157488	-0.000067
H	-2.483263	0.014276	-0.000109
H	2.483312	-0.014272	0.000108

Ph$^{\bullet}$

B3LYP-D3BJ/BS1 SCF energy in DMA:
-231.593307 a.u.
B3LYP-D3BJ/BS2 SCF energy in DMA:
-231.647041 a.u.
B3LYP-D3BJ/BS2 free energy in DMA:
-231.584530 a.u.

C	0.000066	-1.398452	-0.000139
C	1.227511	-0.772414	0.000099
C	-1.227455	-0.772489	0.000140
C	1.214524	0.632708	0.000107
H	2.164733	-1.321525	-0.000426
C	-1.214592	0.632599	0.000041
H	-2.164613	-1.321725	-0.000199
C	-0.000039	1.324262	-0.000031
H	2.154516	1.178009	-0.000106
H	-2.154570	1.177917	-0.000249
H	-0.000157	2.410041	-0.000323

CH_3CH_3

B3LYP-D3BJ/BS1 SCF energy in DMA:
-79.844762 a.u.
B3LYP-D3BJ/BS2 SCF energy in DMA:
-79.862762 a.u.
B3LYP-D3BJ/BS2 free energy in DMA:
-79.808202 a.u.

C	0.763714	0.000000	0.000004
C	-0.763713	0.000000	-0.000010
H	1.162729	0.884108	0.509802
H	1.162729	-0.883585	0.510708
H	-1.162671	0.000442	1.020534
H	-1.162763	0.883591	-0.510608
H	-1.162763	-0.884032	-0.509843
H	1.162734	-0.000523	-1.020561

EtOPh

B3LYP-D3BJ/BS1 SCF energy in DMA:
-386.1448478 a.u.
B3LYP-D3BJ/BS2 SCF energy in DMA:
-386.2379173 a.u.
B3LYP-D3BJ/BS2 free energy in DMA:
-386.1063714 a.u.

C	-2.820092	0.244272	0.000054
C	-2.323713	-1.064379	0.000115
C	-1.922119	1.310073	-0.000038
C	-0.953247	-1.300435	0.000086
H	-3.009003	-1.907273	0.000185
C	-0.540847	1.090407	-0.000070
H	-2.290961	2.331921	-0.000089
C	-0.052635	-0.223100	-0.000005
H	-0.555890	-2.310468	0.000132
H	0.135348	1.936139	-0.000146
O	1.267716	-0.555093	-0.000037
C	2.236160	0.503692	-0.000070

C	3.609770	-0.133360	-0.000011
H	2.095587	1.133942	0.887594
H	2.095631	1.133860	-0.887800
H	4.377712	0.646312	-0.000255
H	3.750240	-0.756322	0.888675
H	3.750093	-0.756719	-0.888443
H	-3.890142	0.426331	0.000079

EtOPh•

B3LYP-D3BJ/BS1 SCF energy in DMA:
-385.4525788 a.u.
B3LYP-D3BJ/BS2 SCF energy in DMA:
-385.5457444 a.u.
B3LYP-D3BJ/BS2 free energy in DMA:
-385.4278415 a.u.

C	-2.817172	0.264303	0.000159
C	-2.403915	-1.053516	0.000032
C	-1.976930	1.352836	0.000024
C	-1.026248	-1.289270	-0.000032
H	-3.102125	-1.885645	0.000132
C	-0.592188	1.107910	-0.000137
H	-2.344756	2.374989	-0.000003
C	-0.121419	-0.213344	-0.000103
H	-0.634543	-2.301950	-0.000162
H	0.093021	1.946814	-0.000177
O	1.196168	-0.557215	-0.000086
C	2.174573	0.492755	0.000059
C	3.541740	-0.157568	0.000090
H	2.039568	1.123906	0.887994
H	2.039669	1.123951	-0.887839
H	4.316945	0.614868	-0.000848
H	3.676517	-0.781224	0.889141
H	3.675719	-0.782627	-0.888096

Ph-O-Ph•

B3LYP-D3BJ/BS1 SCF energy in DMA:
-537.894521 a.u.
B3LYP-D3BJ/BS2 SCF energy in DMA:
-538.020282 a.u.
B3LYP-D3BJ/BS2 free energy in DMA:
-537.882700 a.u.

O	0.059142	-1.200977	0.090923
C	-1.133080	-0.510323	-0.002934
C	-1.265131	0.667963	-0.742927
C	-2.237616	-1.085968	0.627616
C	-2.519027	1.272821	-0.837424
H	-0.403554	1.102886	-1.236820
C	-3.485956	-0.474294	0.517125
H	-2.105514	-2.002232	1.193548
C	-3.632606	0.708462	-0.211456
H	-2.622893	2.188603	-1.411758
H	-4.344151	-0.922938	1.008412
C	1.252303	-0.501323	0.083097
C	2.265845	-0.983408	-0.746692
C	1.457708	0.592638	0.930900
C	3.524272	-0.362885	-0.734103
H	2.068145	-1.835107	-1.389820
C	2.707086	1.227352	0.942459
H	0.654656	0.944059	1.570639
C	3.679124	0.715099	0.109155
H	4.323354	-0.727665	-1.372569
H	2.883762	2.078389	1.593448
H	-4.604465	1.185014	-0.291363

7. NMR spectra

^{1}H NMR of compound **3** (500 MHz in $CDCl_3$)

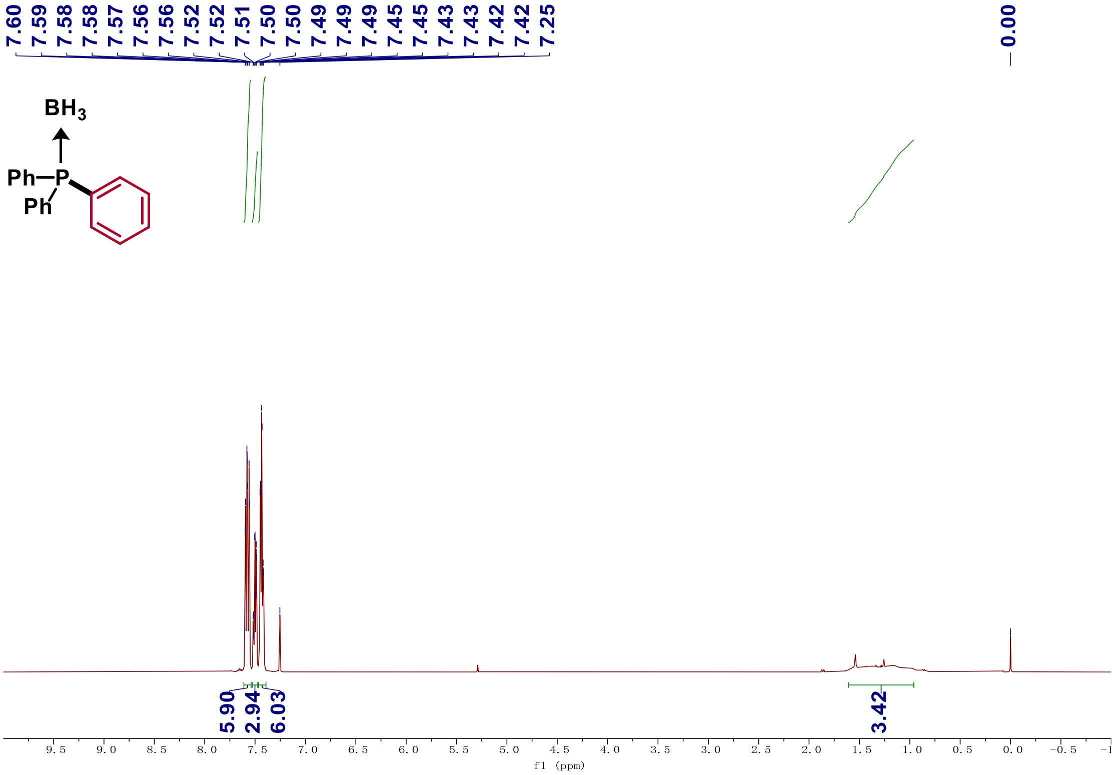

^{13}C NMR of compound **3** (126 MHz in $CDCl_3$)

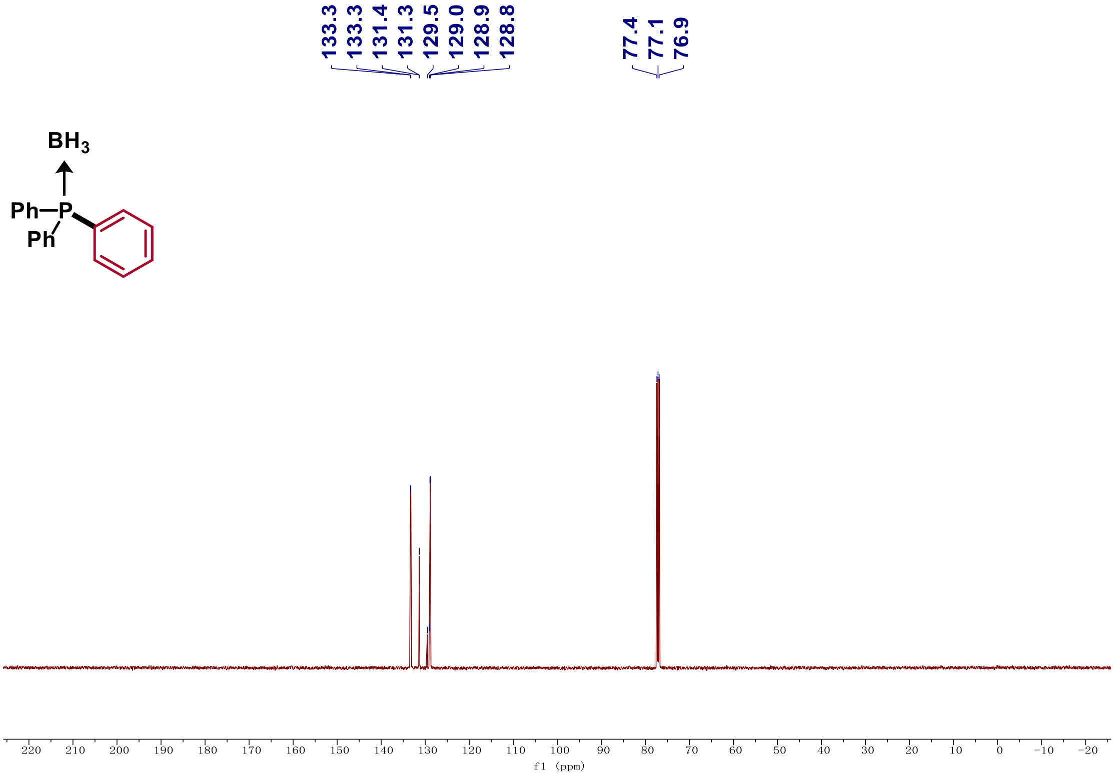

^{11}B NMR of compound **3** (160 MHz in $CDCl_3$)

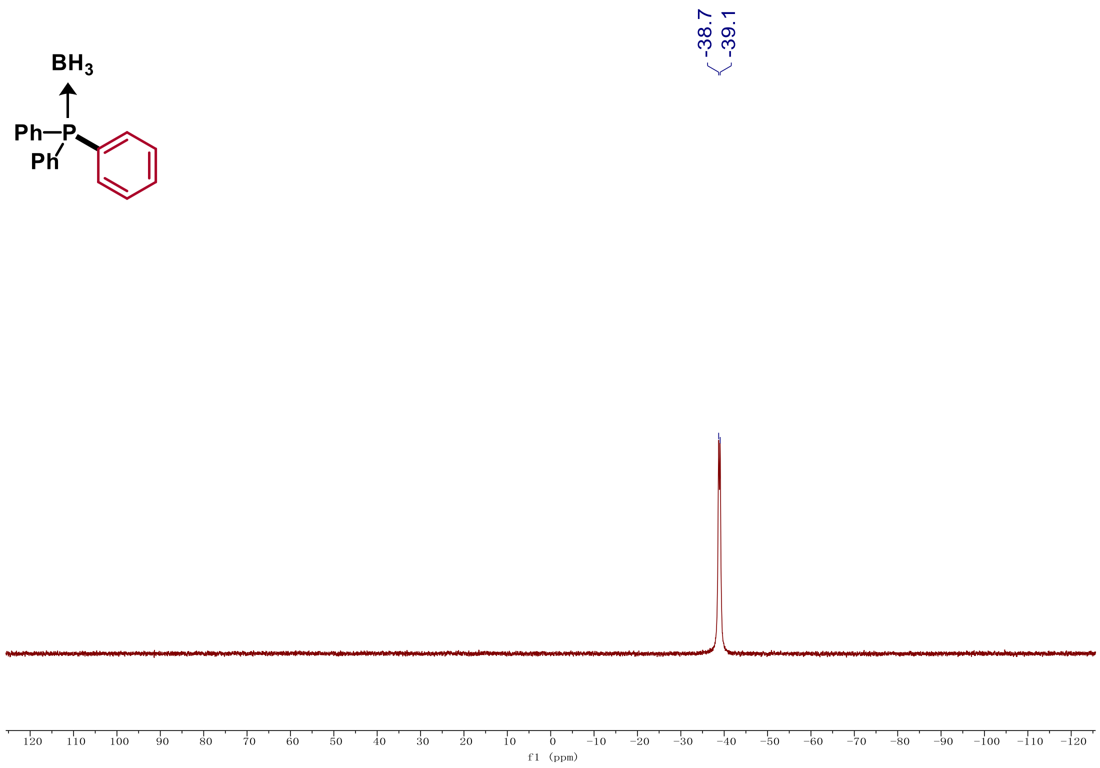

^{31}P NMR of compound **3** (202 MHz in $CDCl_3$)

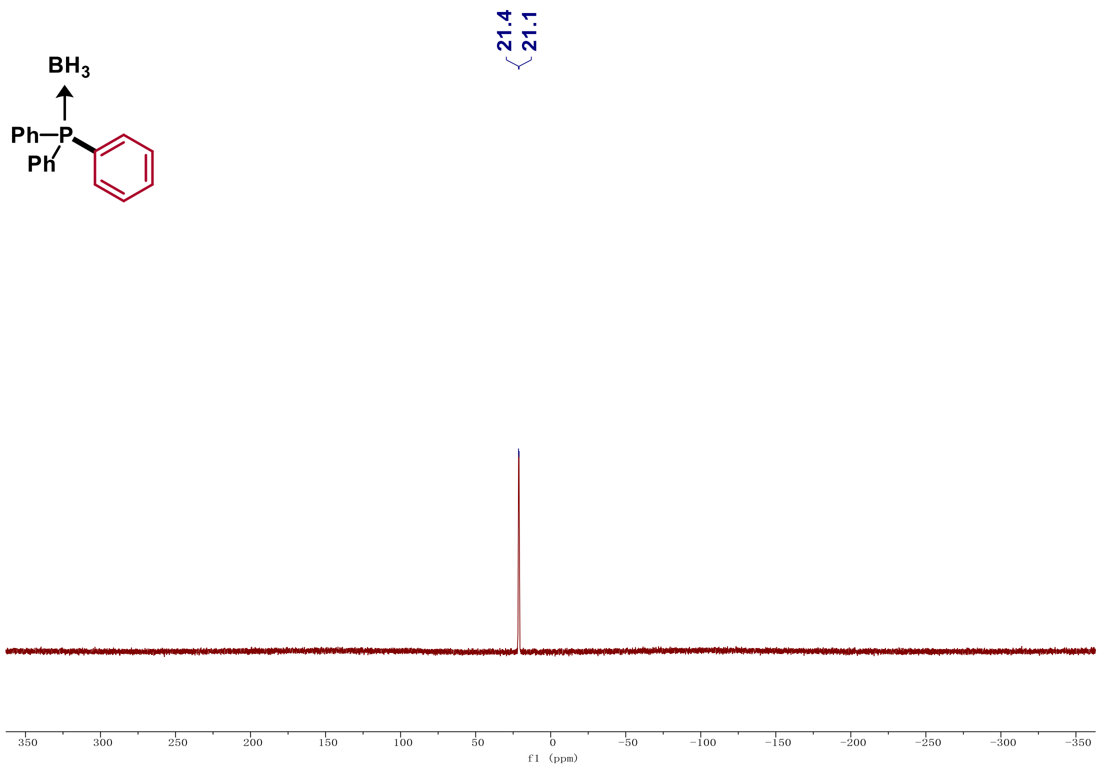

^{1}H NMR of compound **4** (500 MHz in $CDCl_3$)

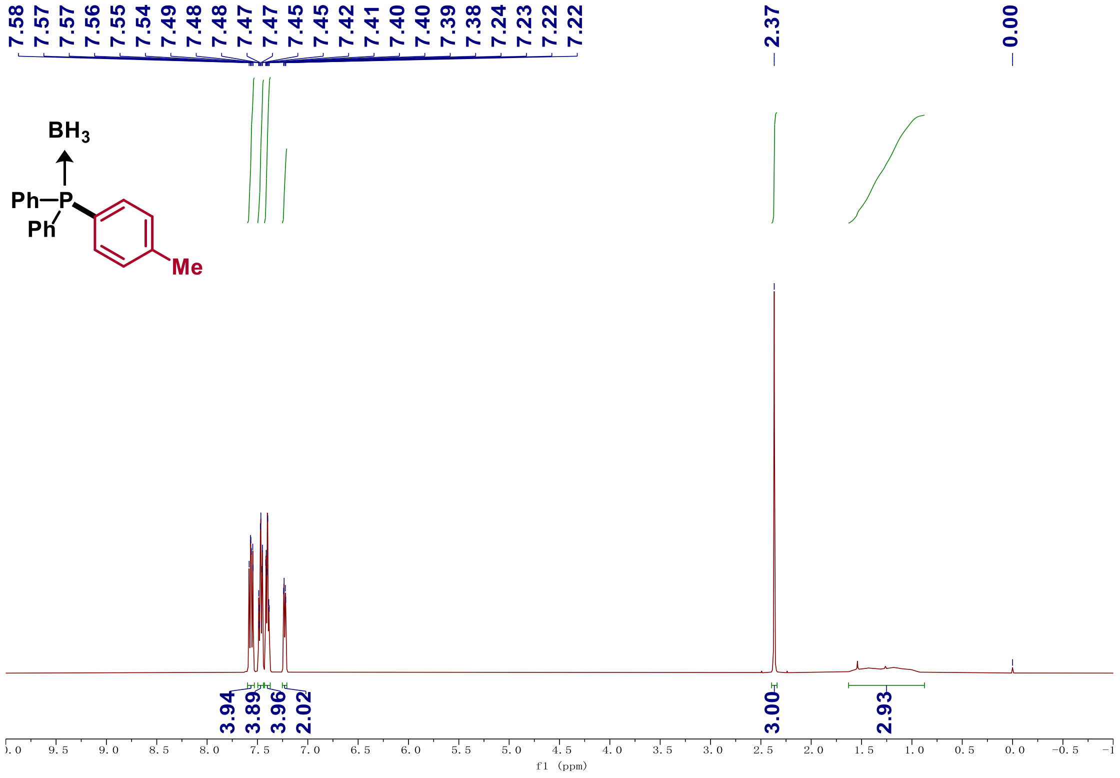

^{13}C NMR of compound **4** (126 MHz in $CDCl_3$)

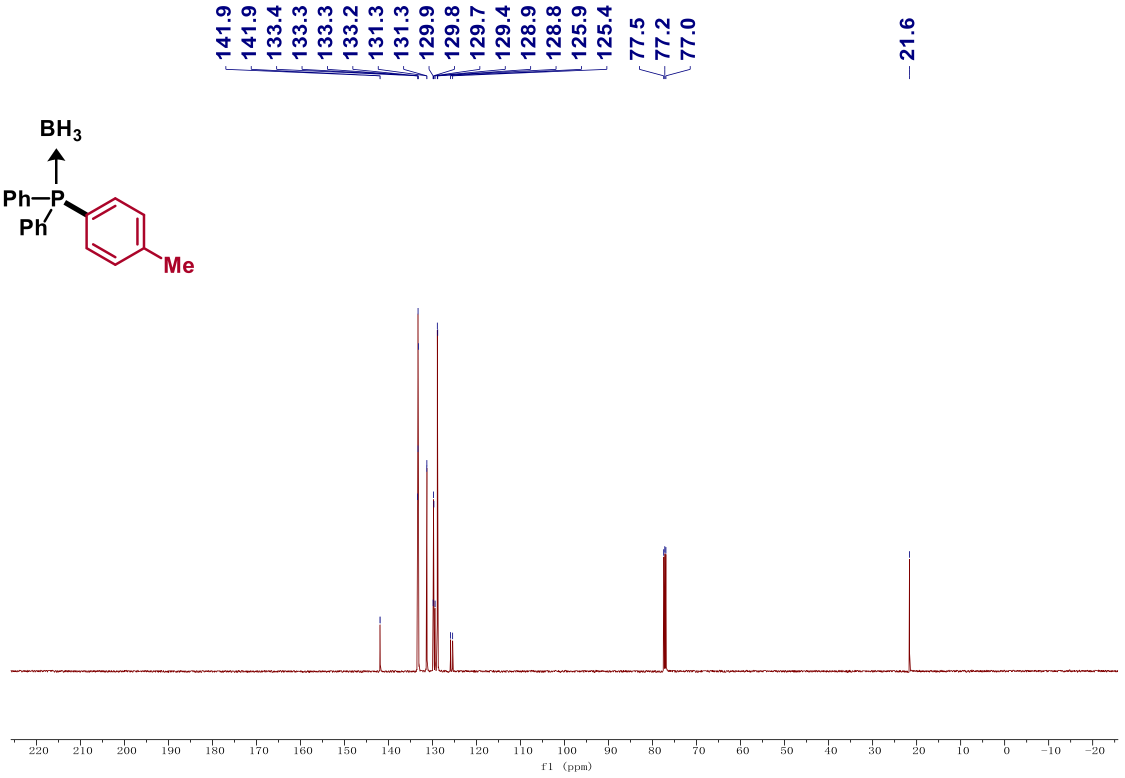

^{11}**B NMR** of compound **4** (160 MHz in $CDCl_3$)

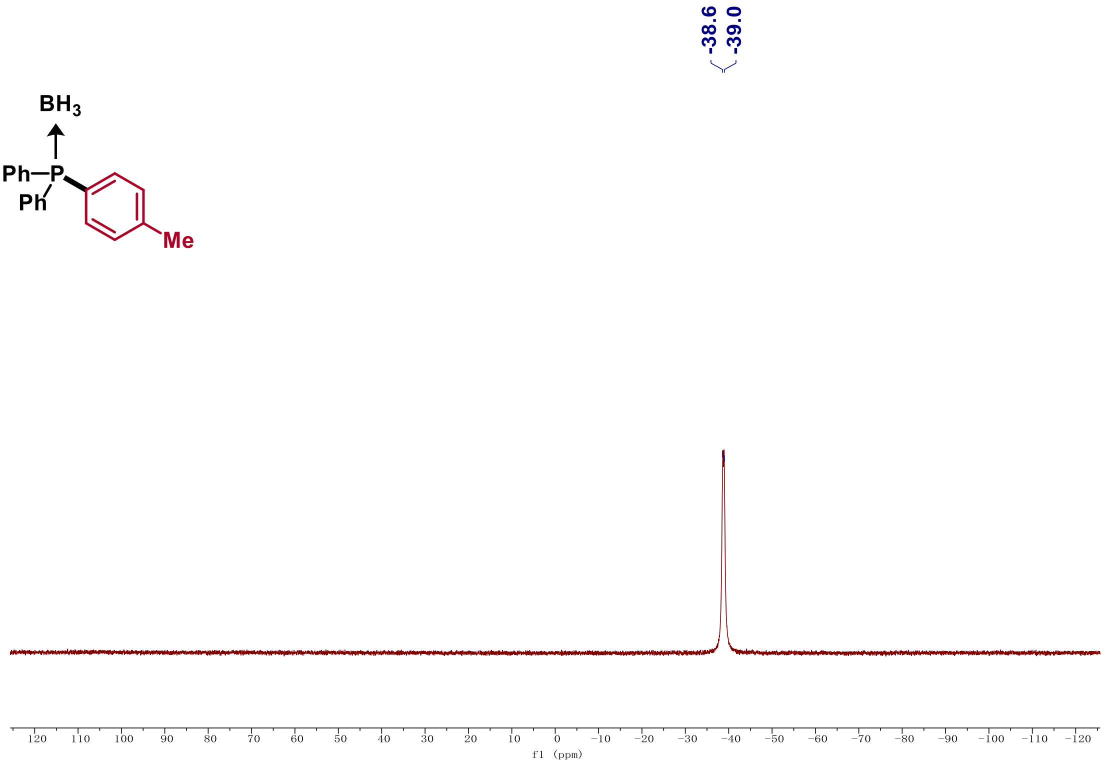

^{31}**P NMR** of compound **4** (202 MHz in $CDCl_3$)

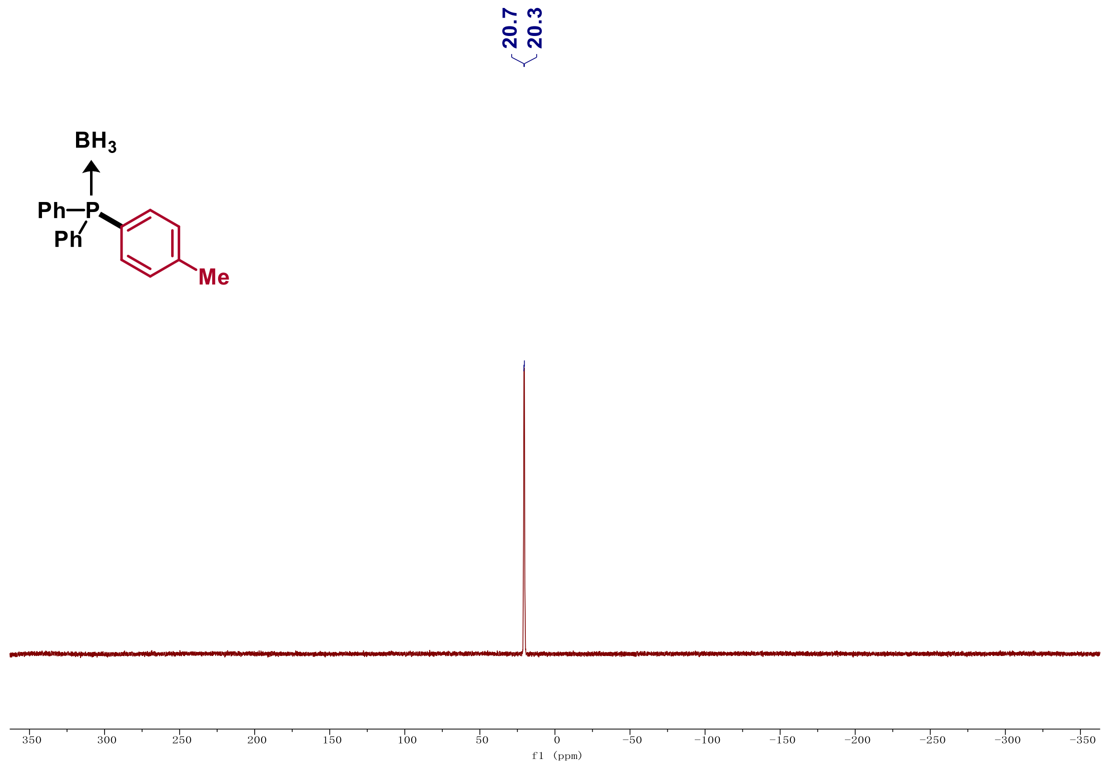

^{1}H NMR of compound **5** (500 MHz in $CDCl_3$)

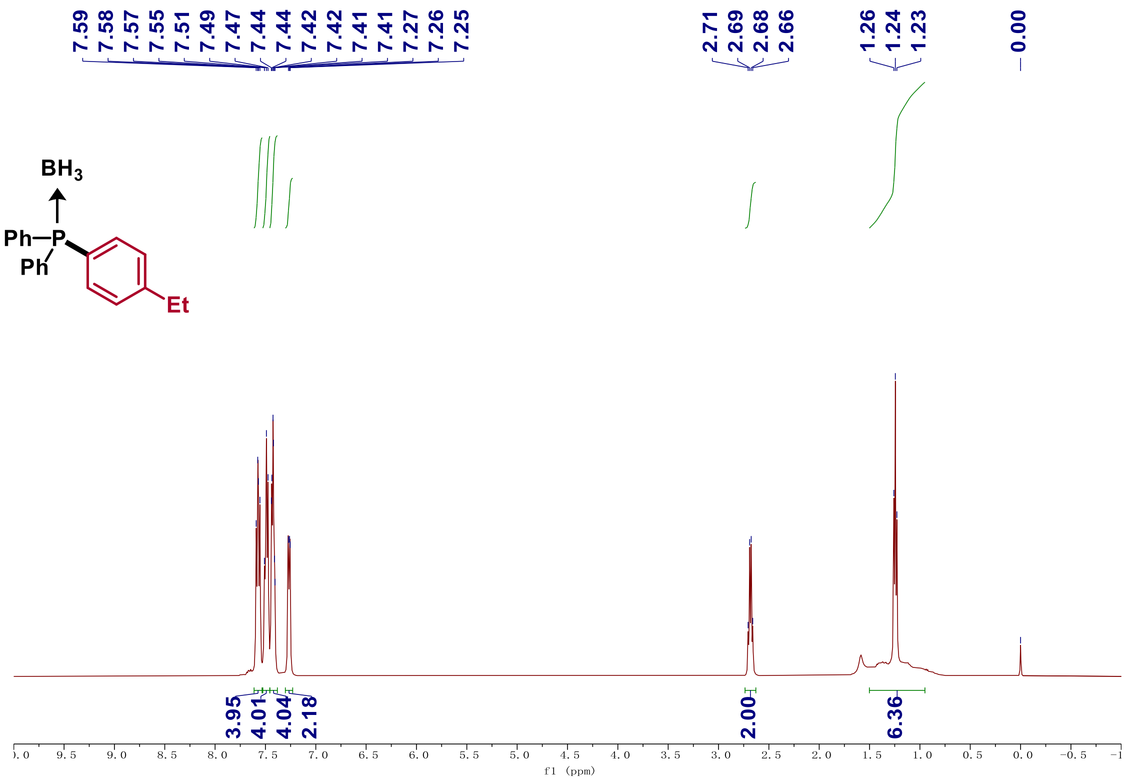

^{13}C NMR of compound **5** (126 MHz in $CDCl_3$)

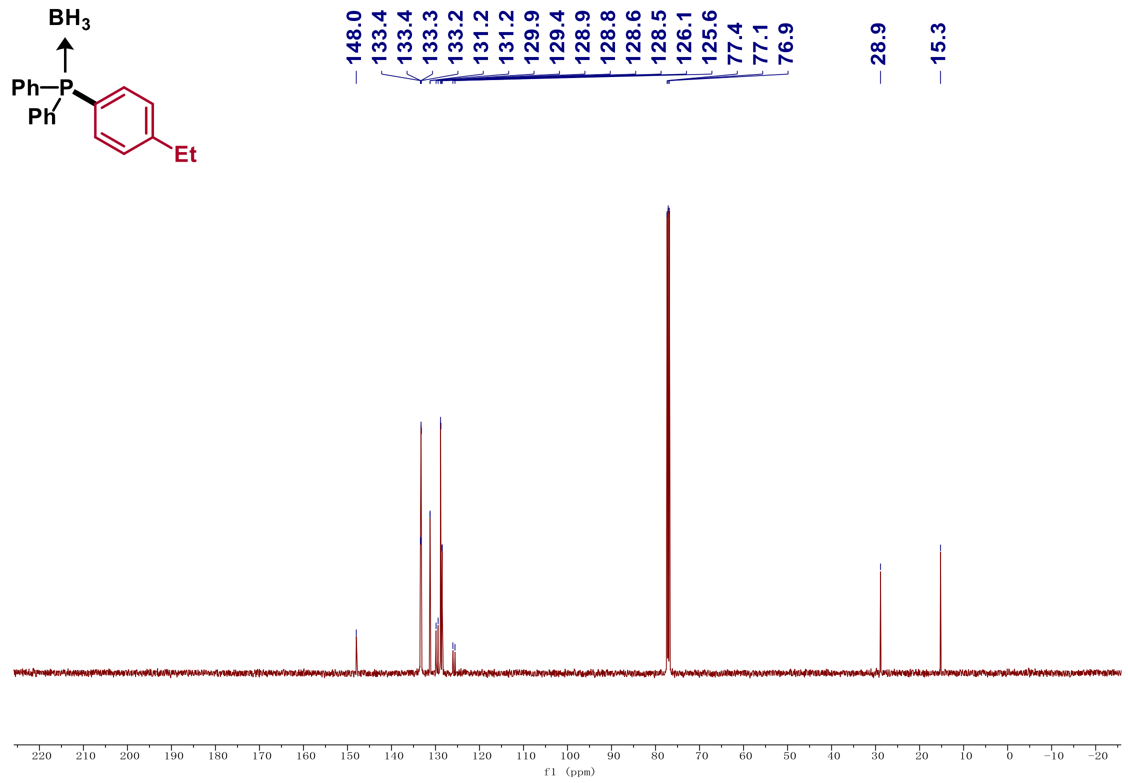

^{11}B NMR of compound **5** (160 MHz in $CDCl_3$)

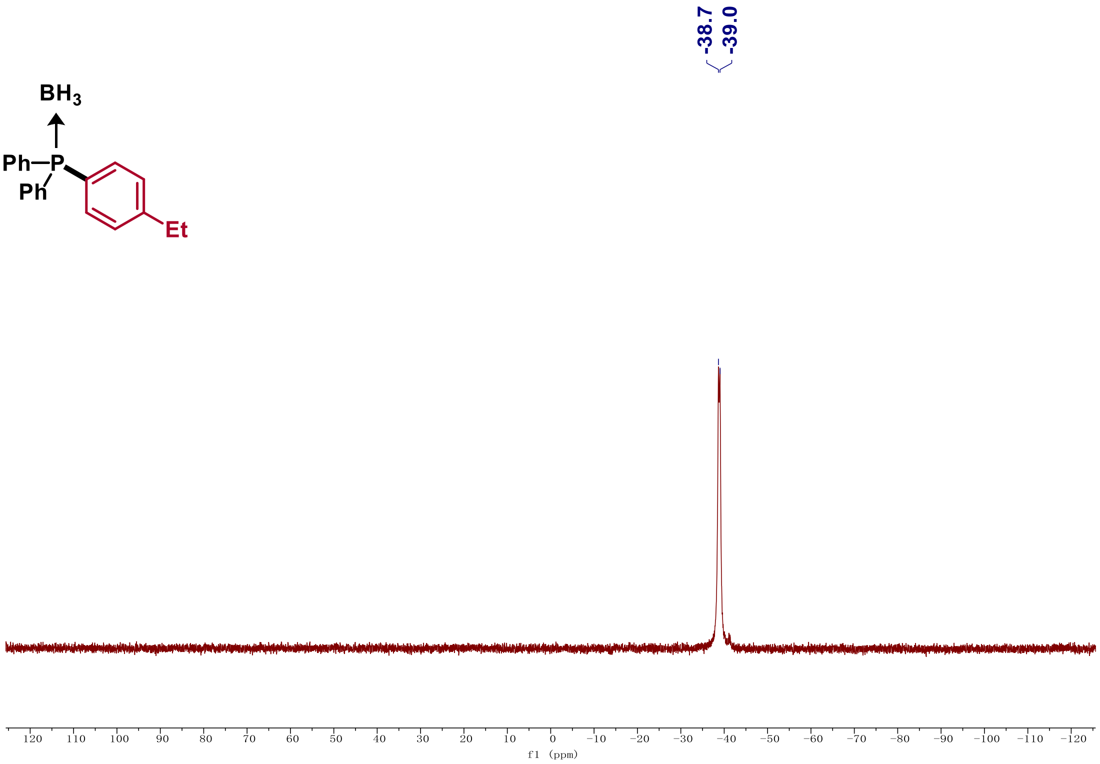

^{31}P NMR of compound **5** (202 MHz in $CDCl_3$)

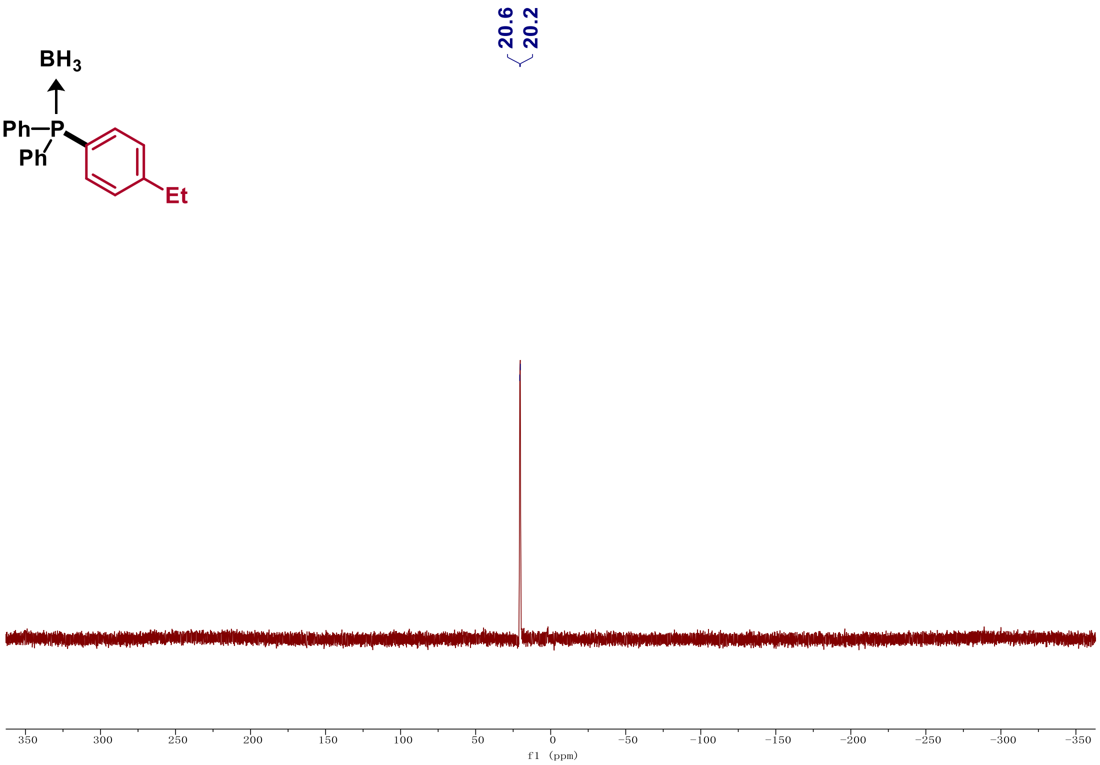

^{1}H NMR of compound **6** (500 MHz in DMSO)

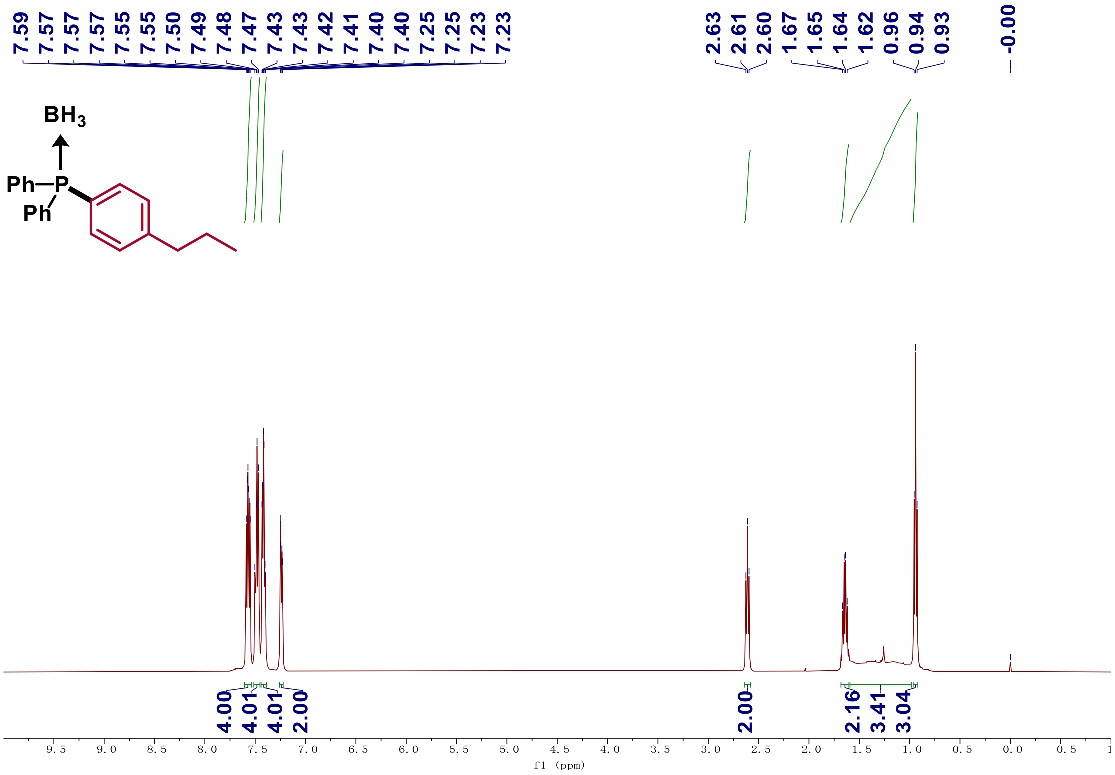

^{13}C NMR of compound **6** (126 MHz in $CDCl_3$)

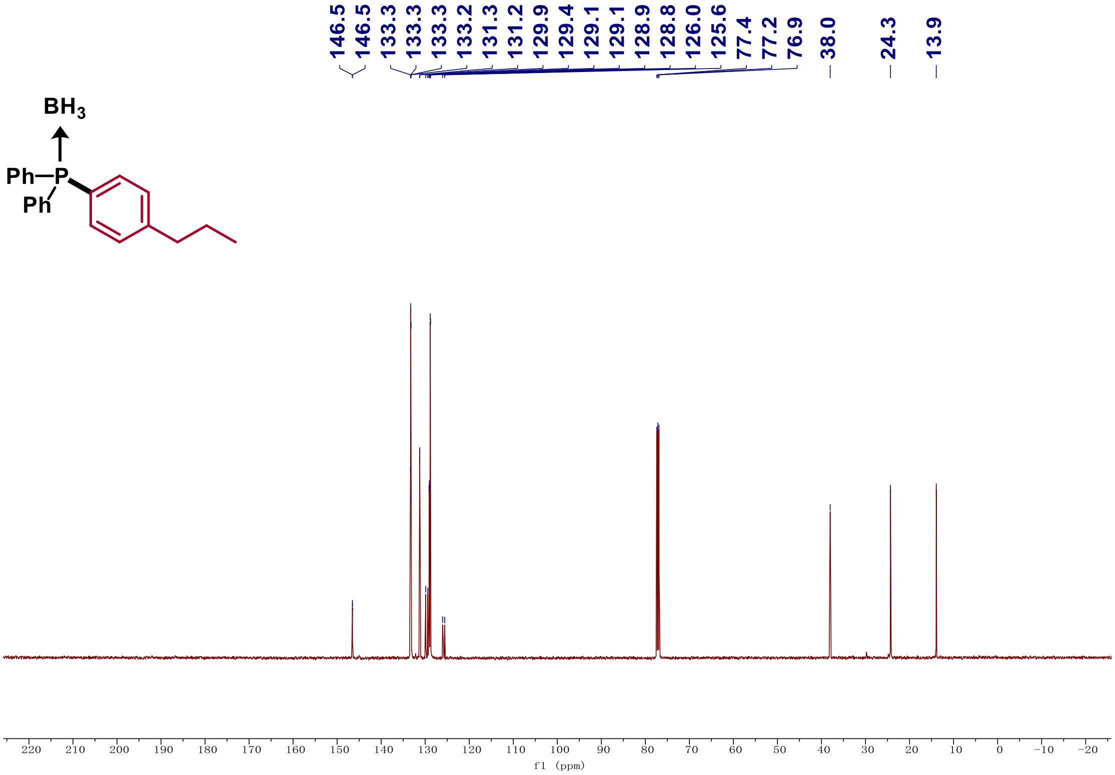

^{11}**B NMR** of compound **6** (160 MHz in $CDCl_3$)

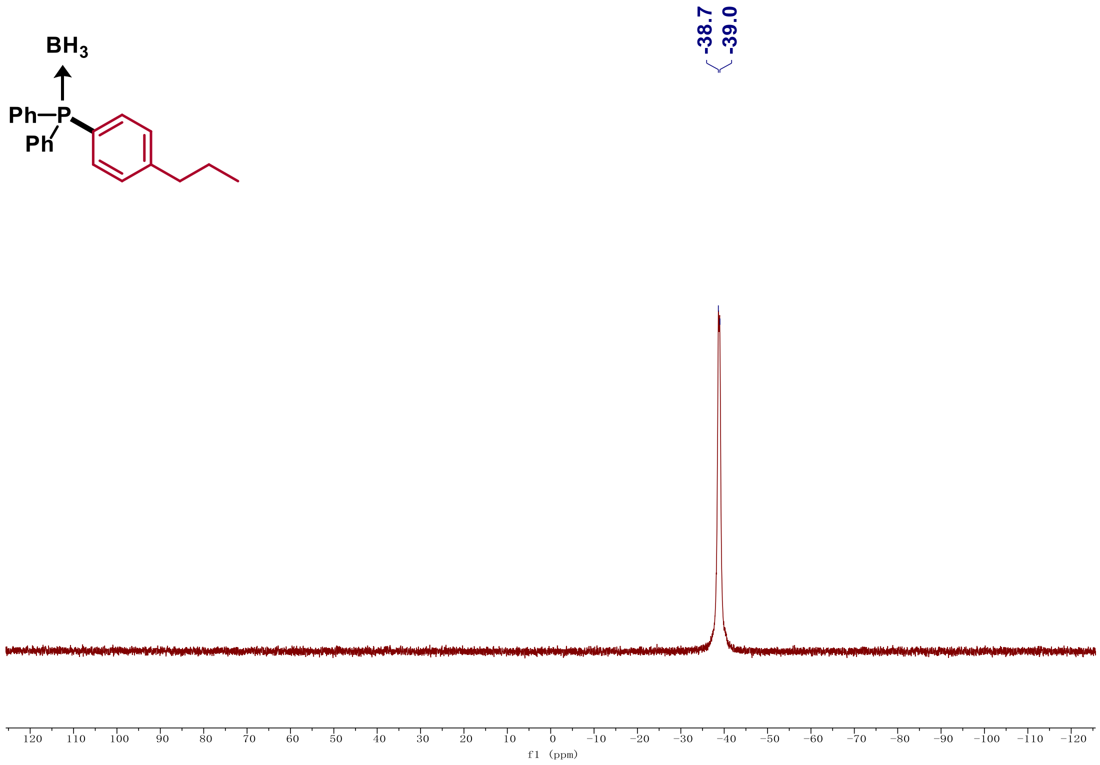

^{31}**P NMR** of compound **6** (202 MHz in $CDCl_3$)

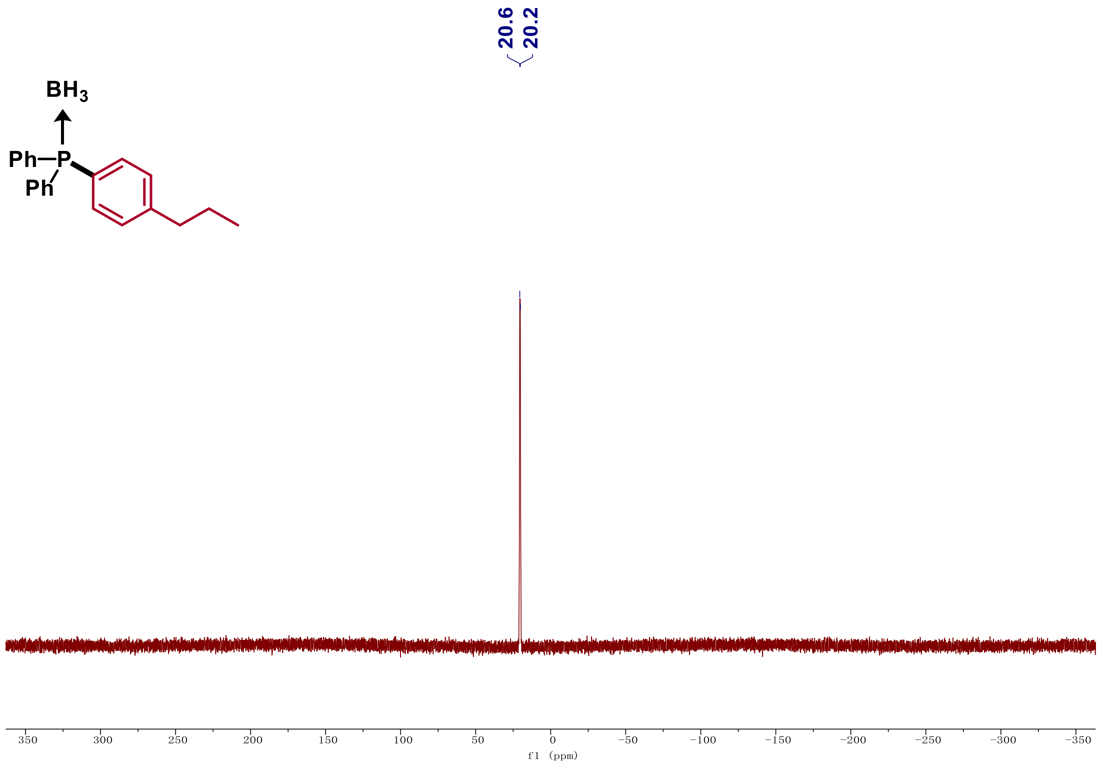

^{1}H NMR of compound 7 (500 MHz in DMSO-*d*$_6$)

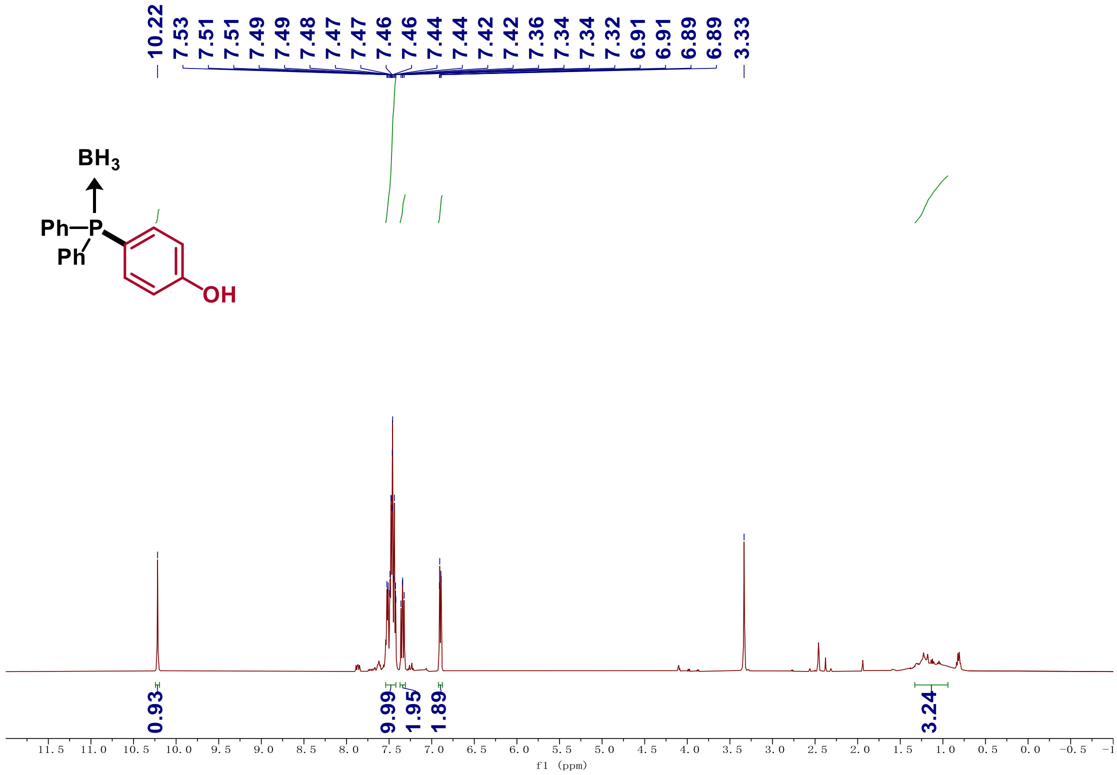

^{13}C NMR of compound **7** (126 MHz in $CDCl_3$)

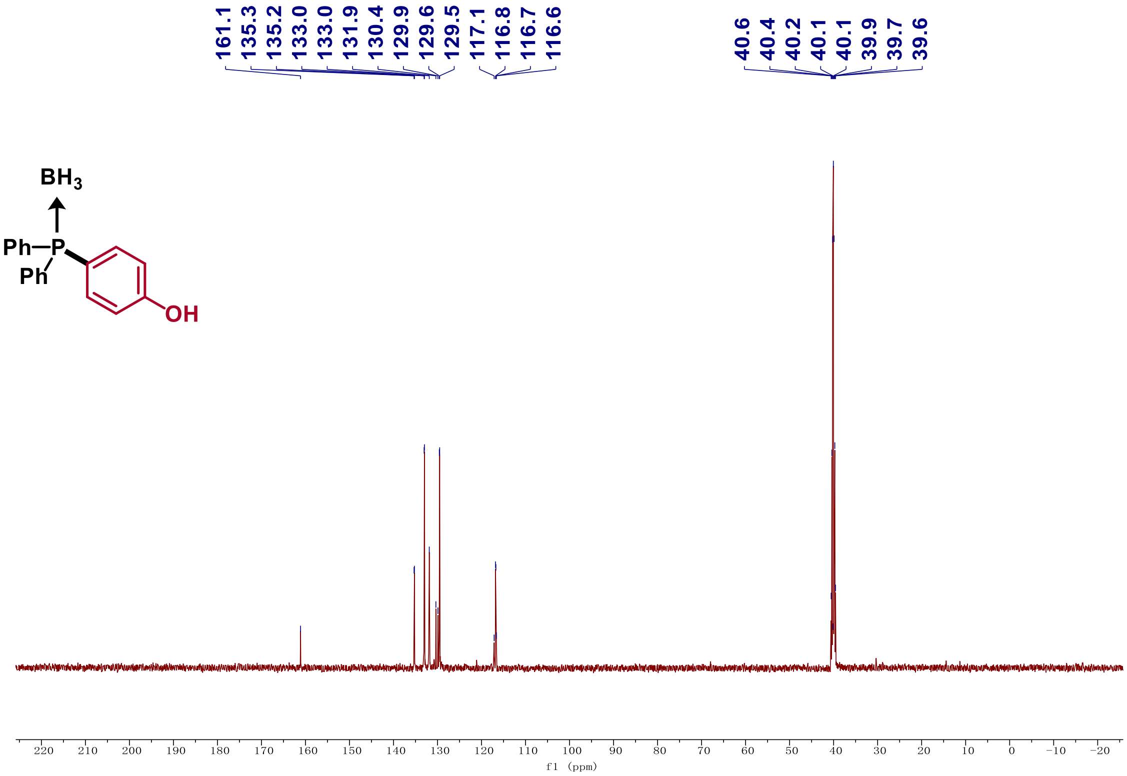

^{11}B NMR of compound **7** (160 MHz in $CDCl_3$)

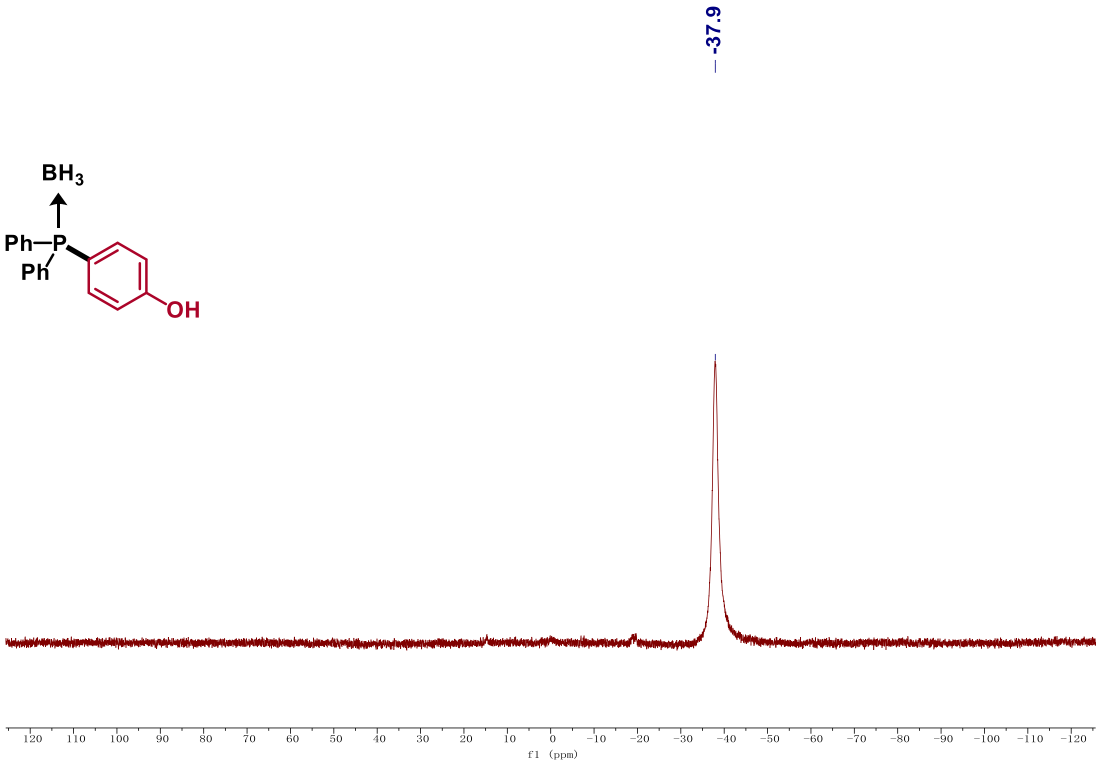

^{31}P NMR of compound **7** (202 MHz in $CDCl_3$)

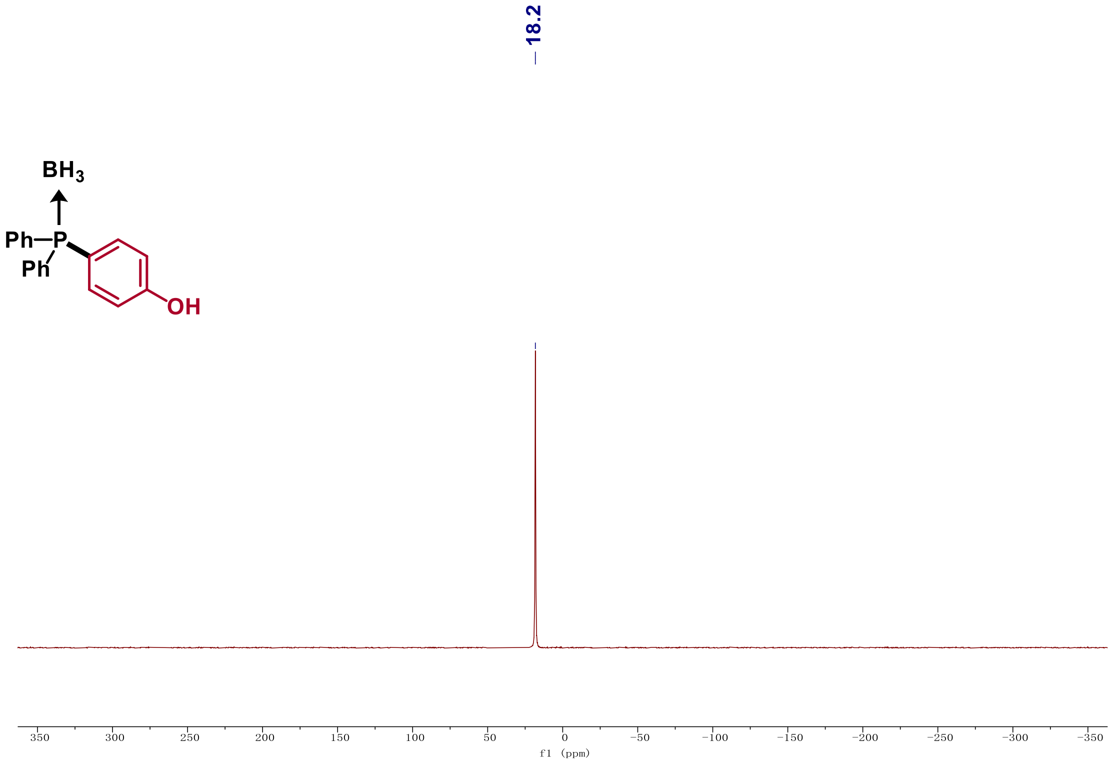

^{1}H NMR of compound **8** (500 MHz in $CDCl_3$)

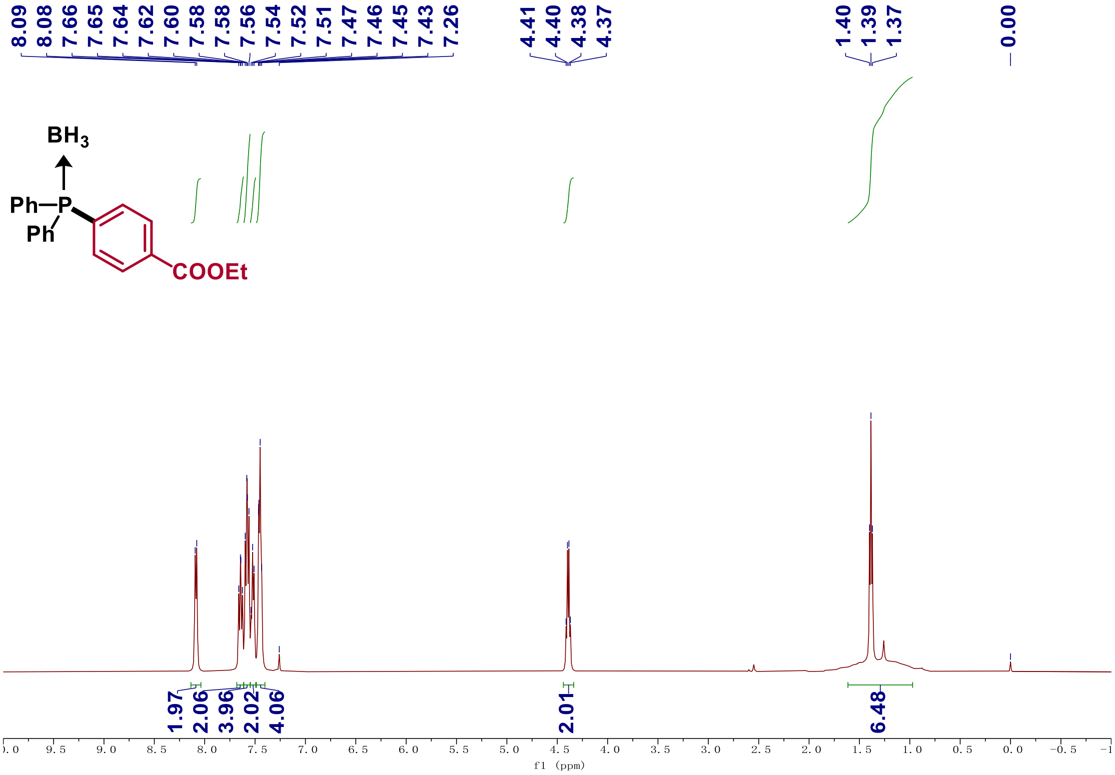

^{13}C NMR of compound **8** (126 MHz in $CDCl_3$)

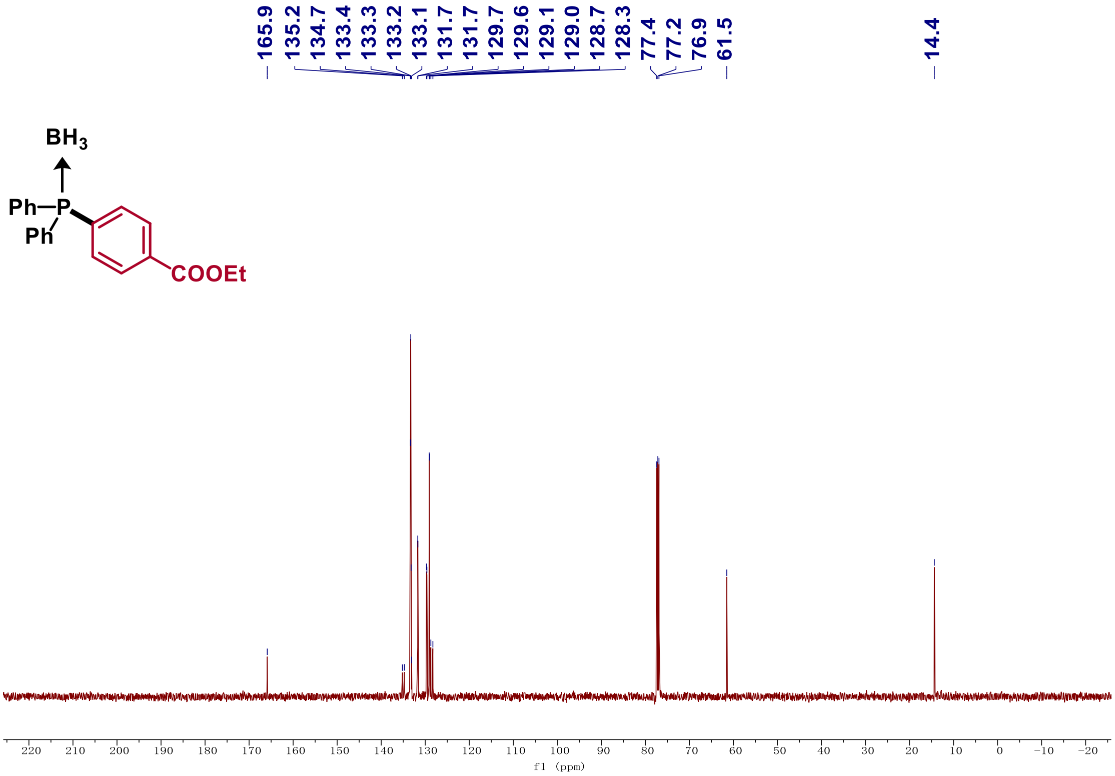

^{11}B NMR of compound **8** (160 MHz in $CDCl_3$)

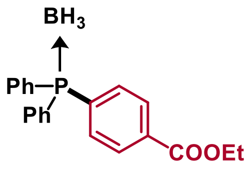

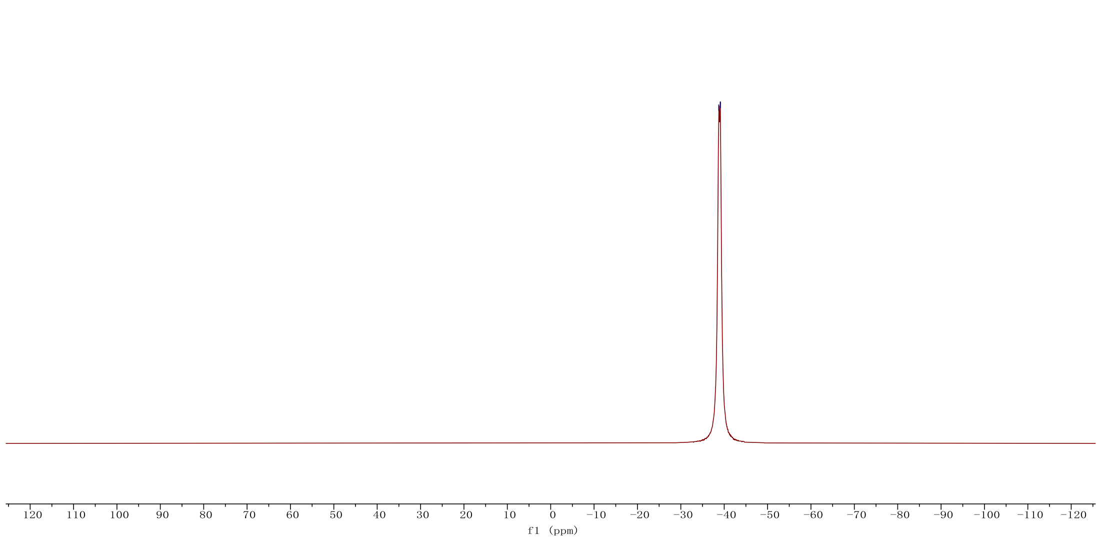

^{31}P NMR of compound **8** (202 MHz in $CDCl_3$)

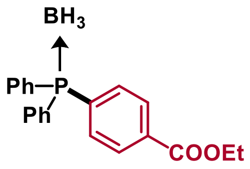

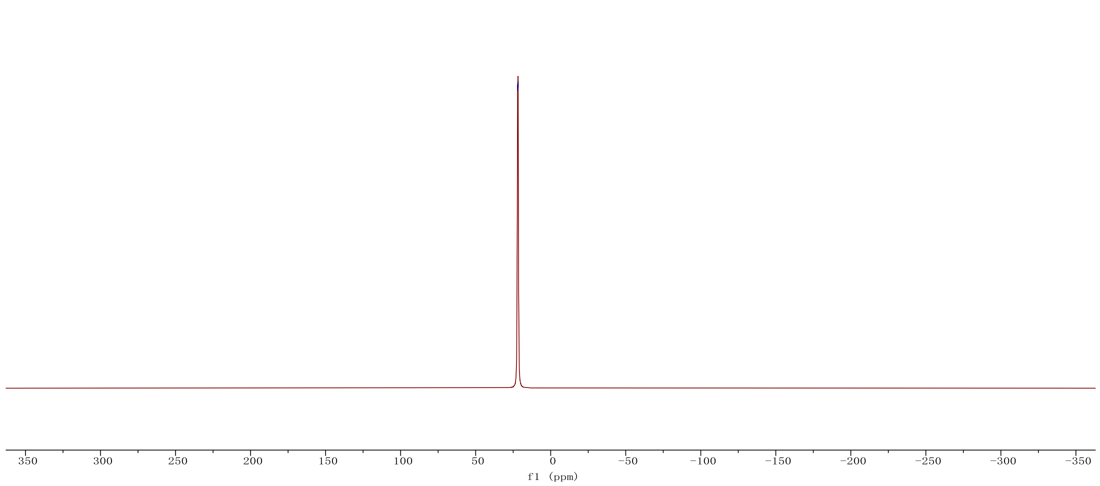

^{1}H NMR of compound **9** (500 MHz in $CDCl_3$)

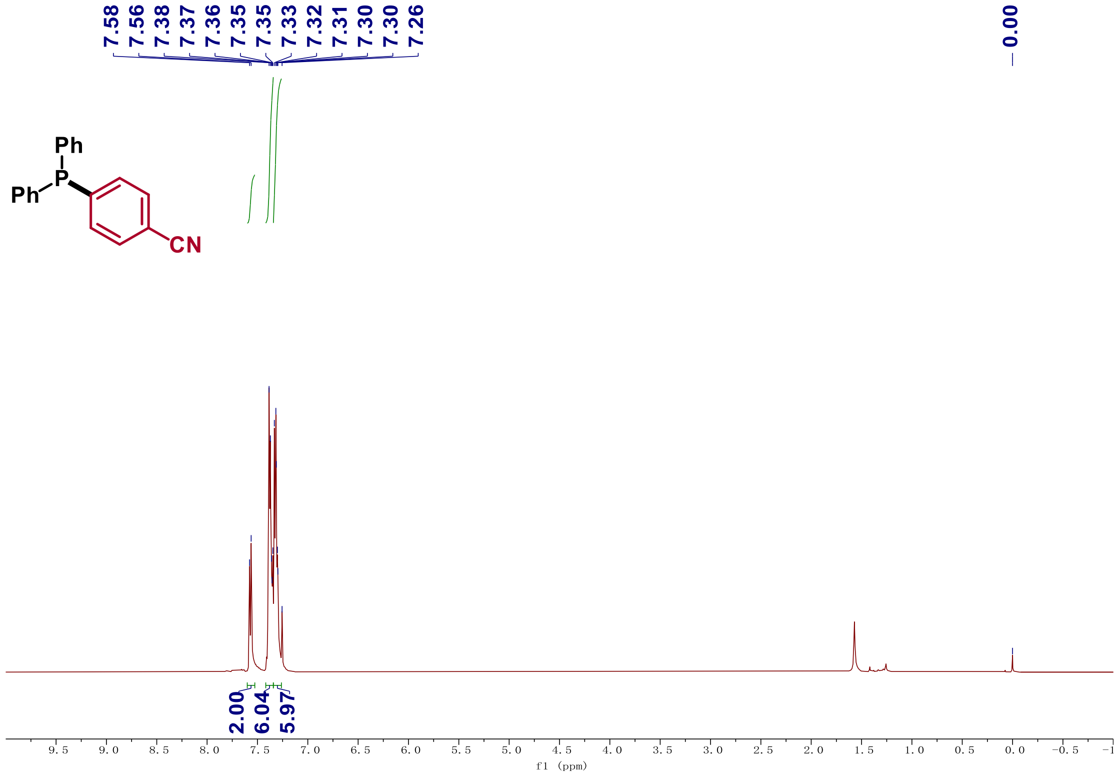

^{13}C NMR of compound **9** (126 MHz in $CDCl_3$)

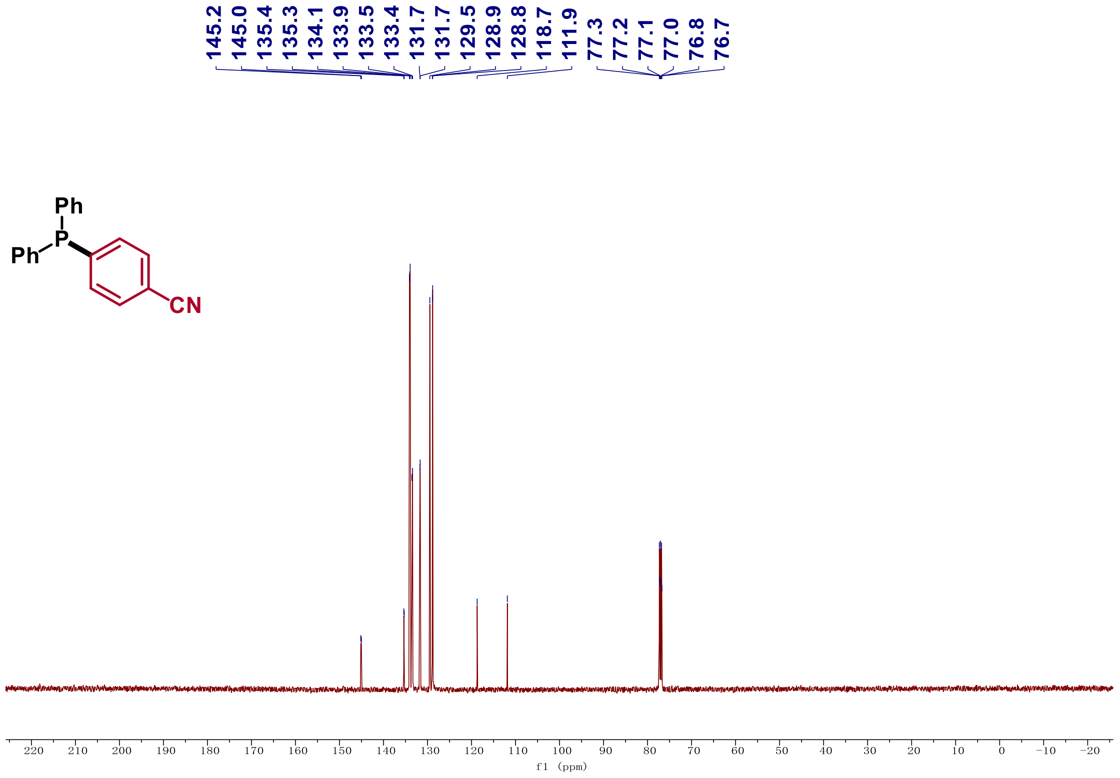

^{31}P NMR of compound **9** (202 MHz in $CDCl_3$)

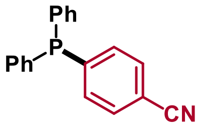

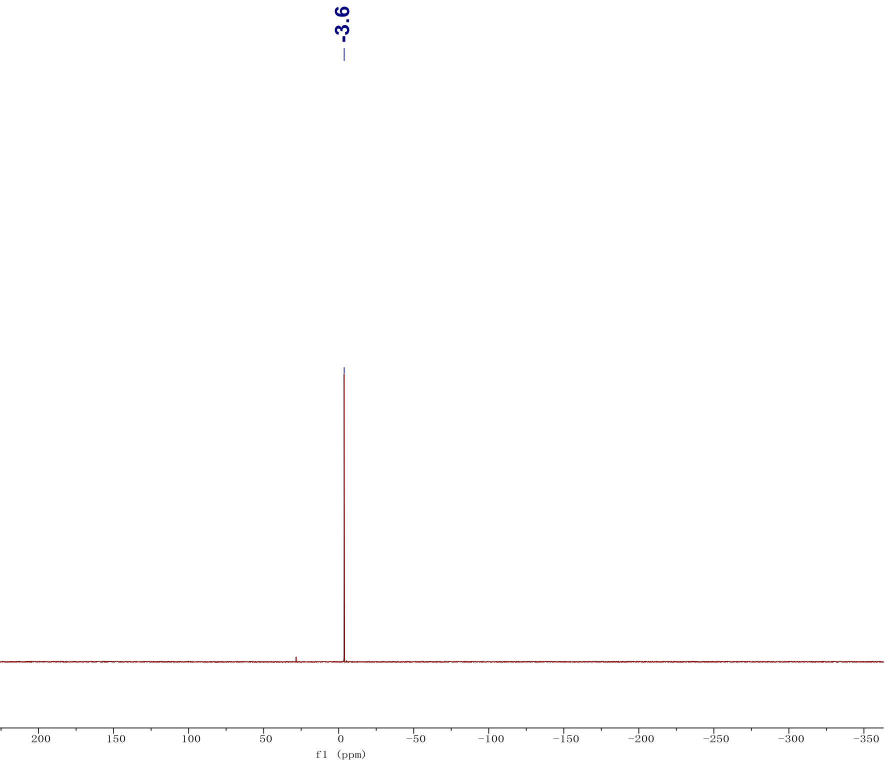

^{1}H NMR of compound **10** (500 MHz in $CDCl_3$)

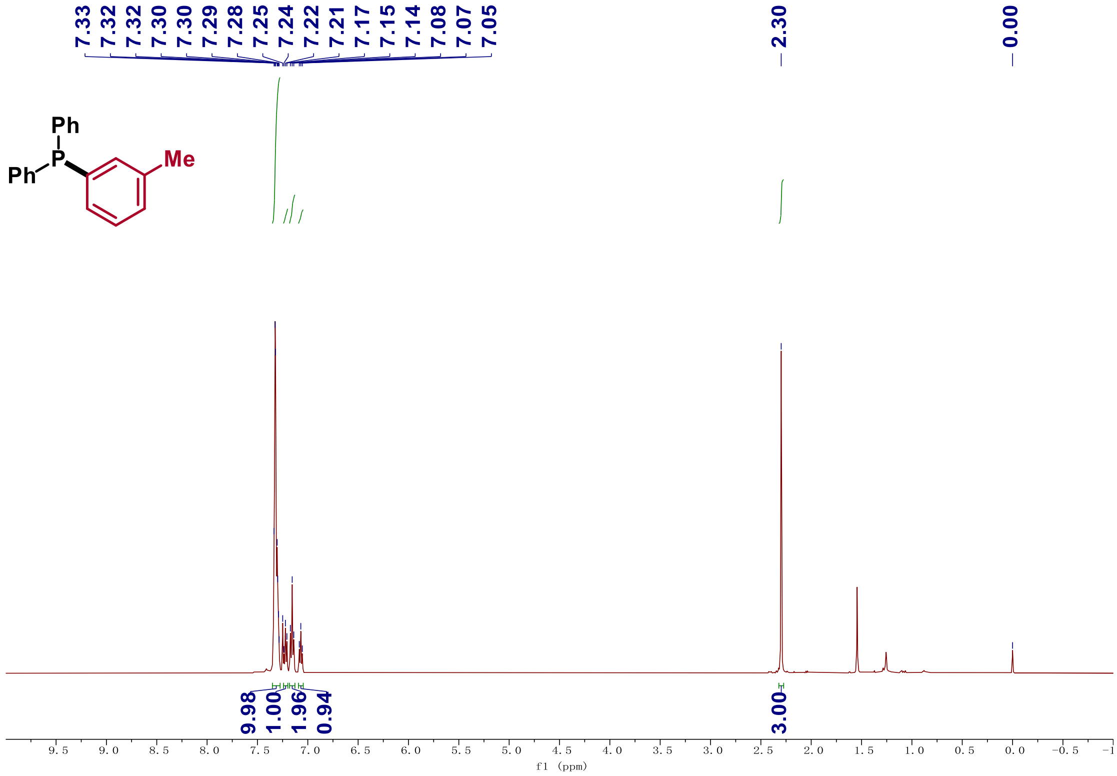

^{13}C NMR of compound **10** (126 MHz in $CDCl_3$)

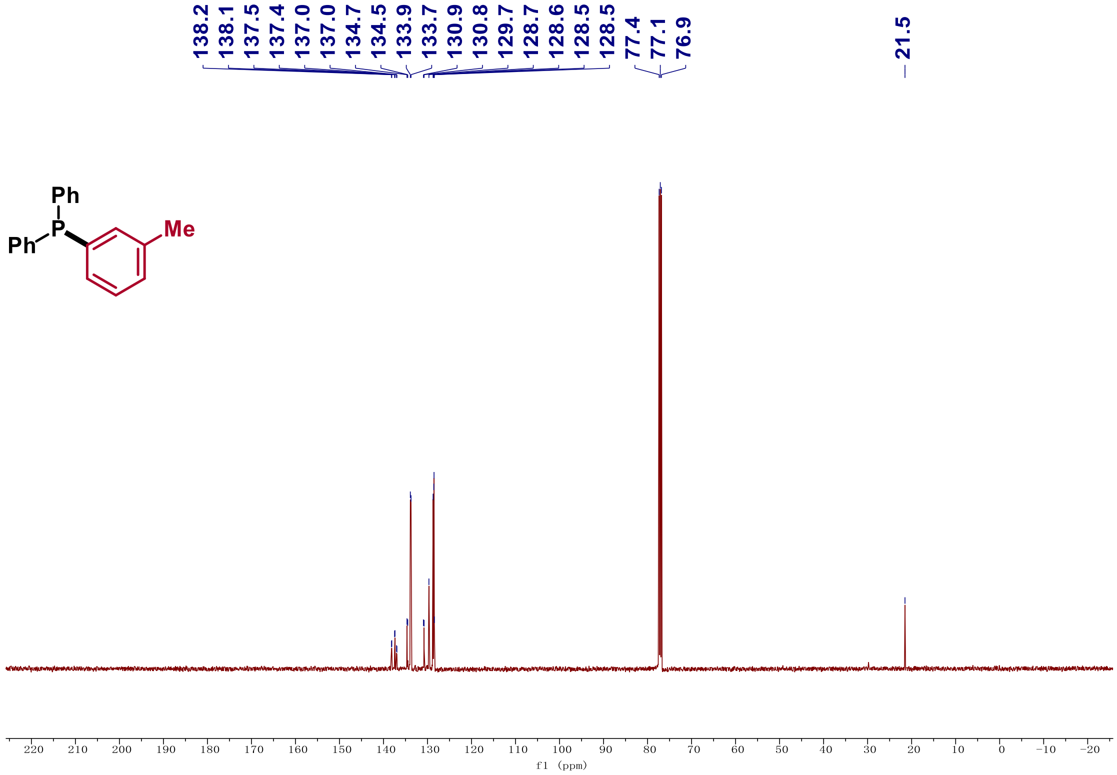

^{31}P NMR of compound **10** (202 MHz in $CDCl_3$)

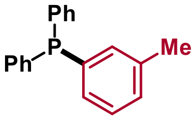

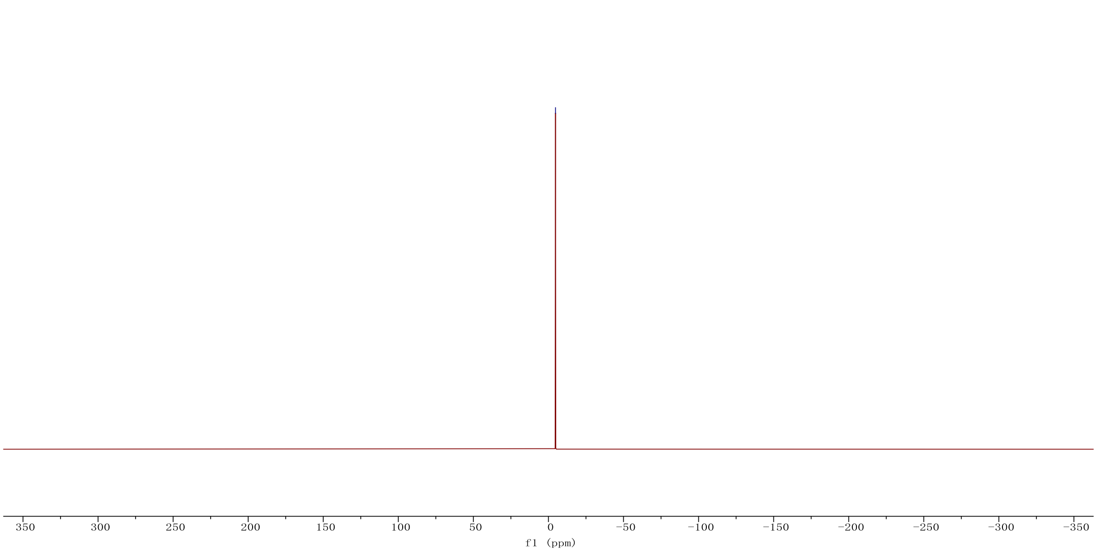

^{1}H NMR of compound **11** (500 MHz in $CDCl_3$)

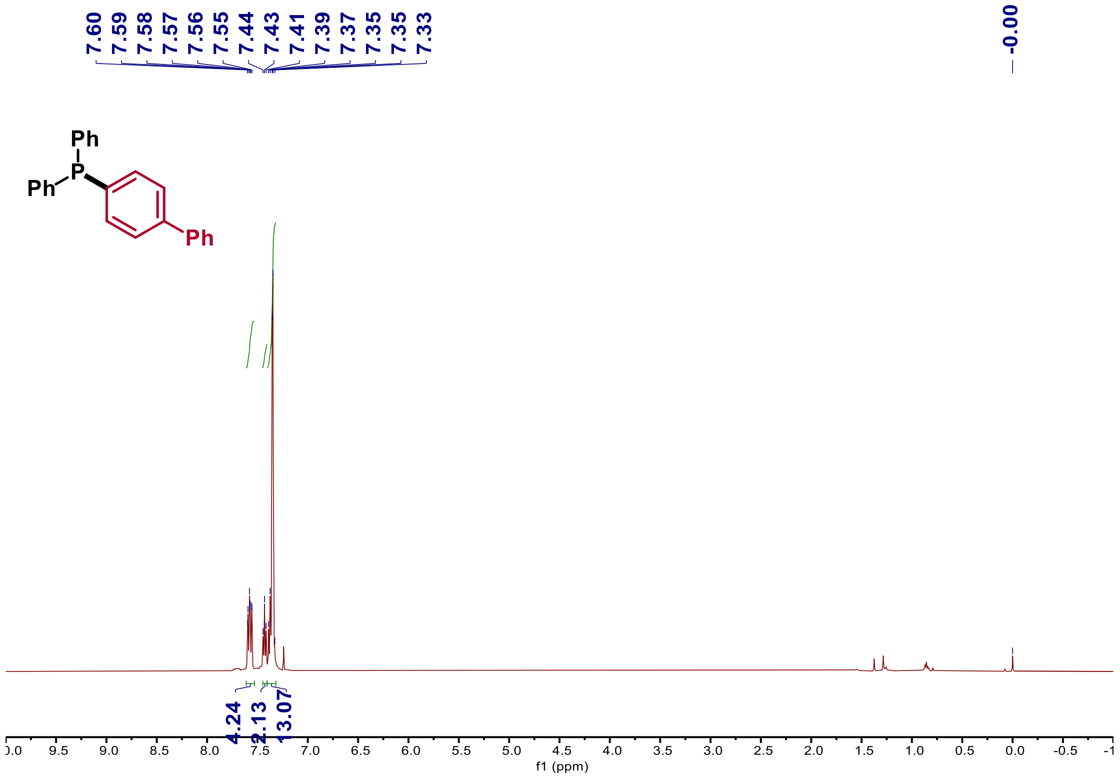

^{13}C NMR of compound **11** (126 MHz in $CDCl_3$)

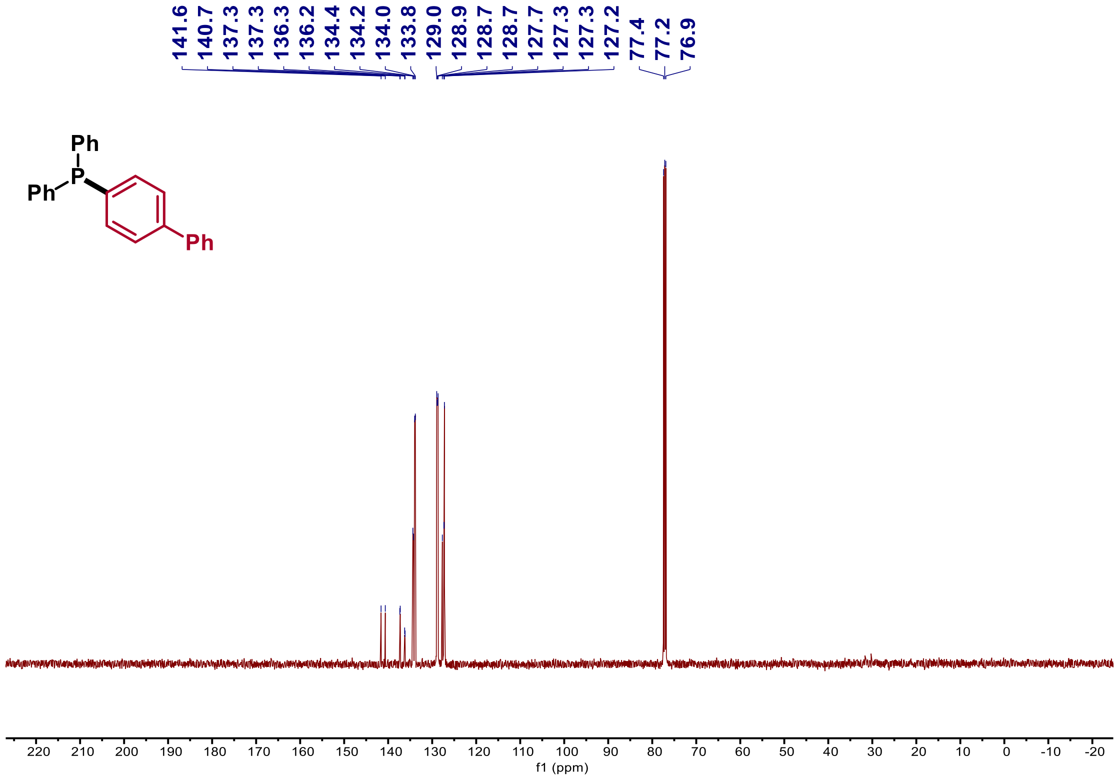

^{31}P NMR of compound **11** (202 MHz in $CDCl_3$)

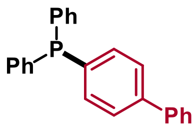

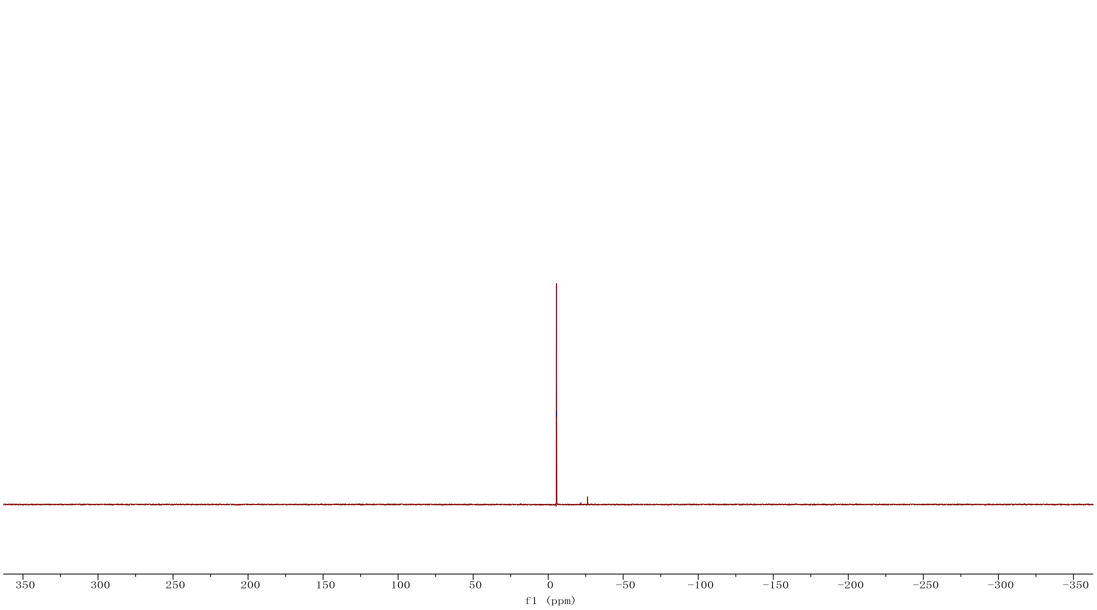

^{1}H NMR of compound **12** (500 MHz in $CDCl_3$)

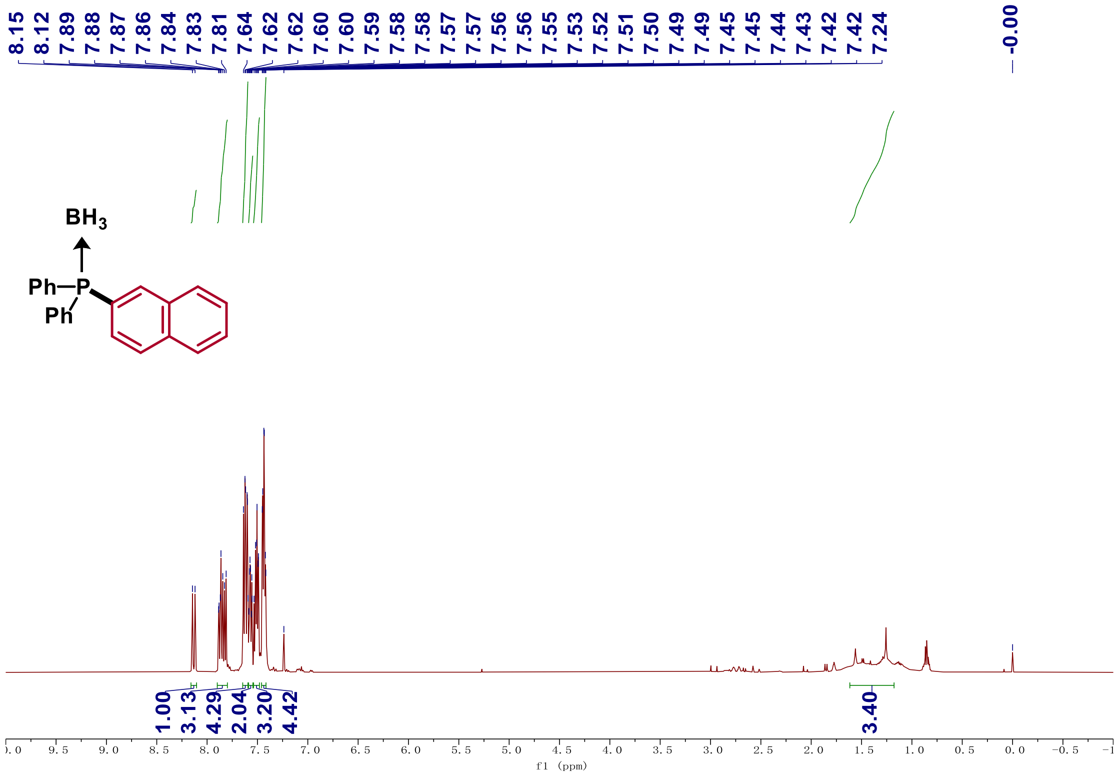

^{13}C NMR of compound **12** (126 MHz in $CDCl_3$)

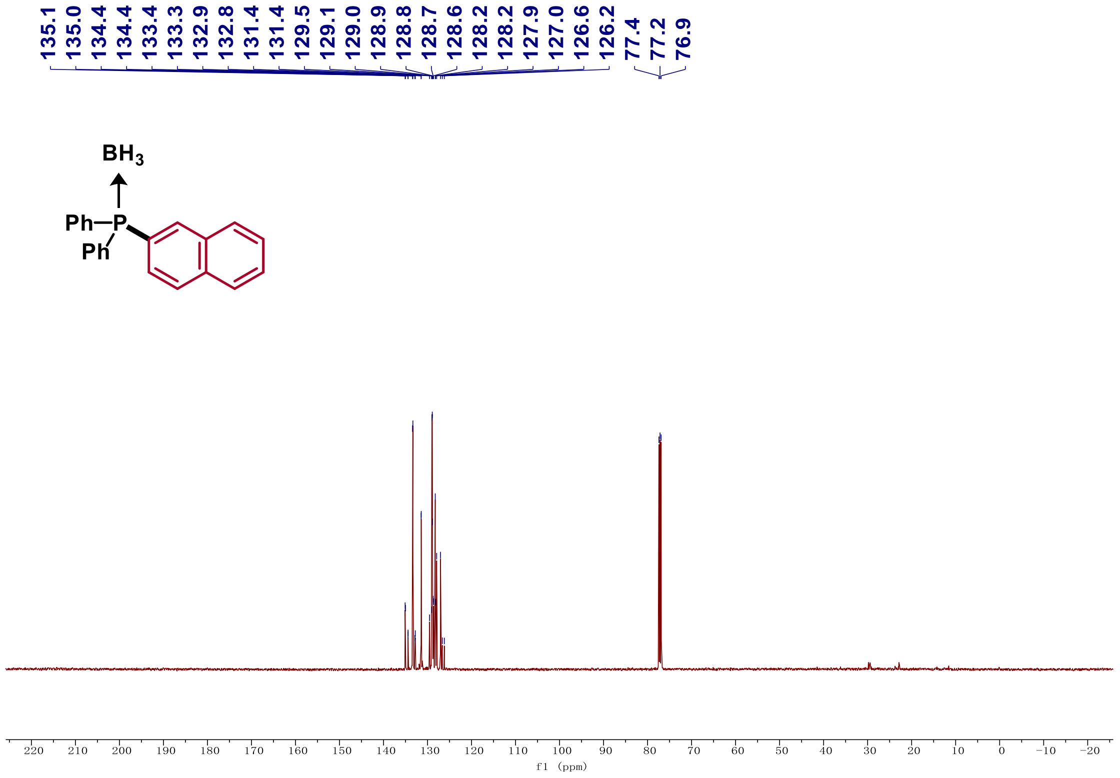

^{11}B NMR of compound **12** (160 MHz in $CDCl_3$)

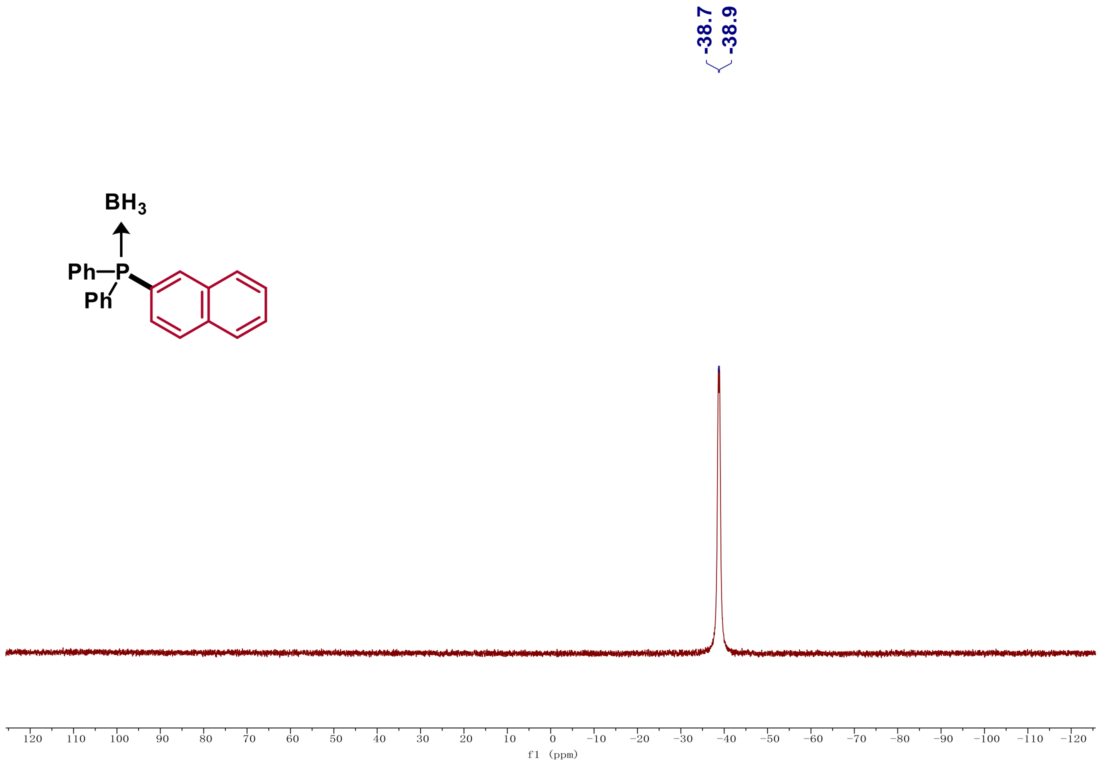

^{31}P NMR of compound **12** (202 MHz in $CDCl_3$)

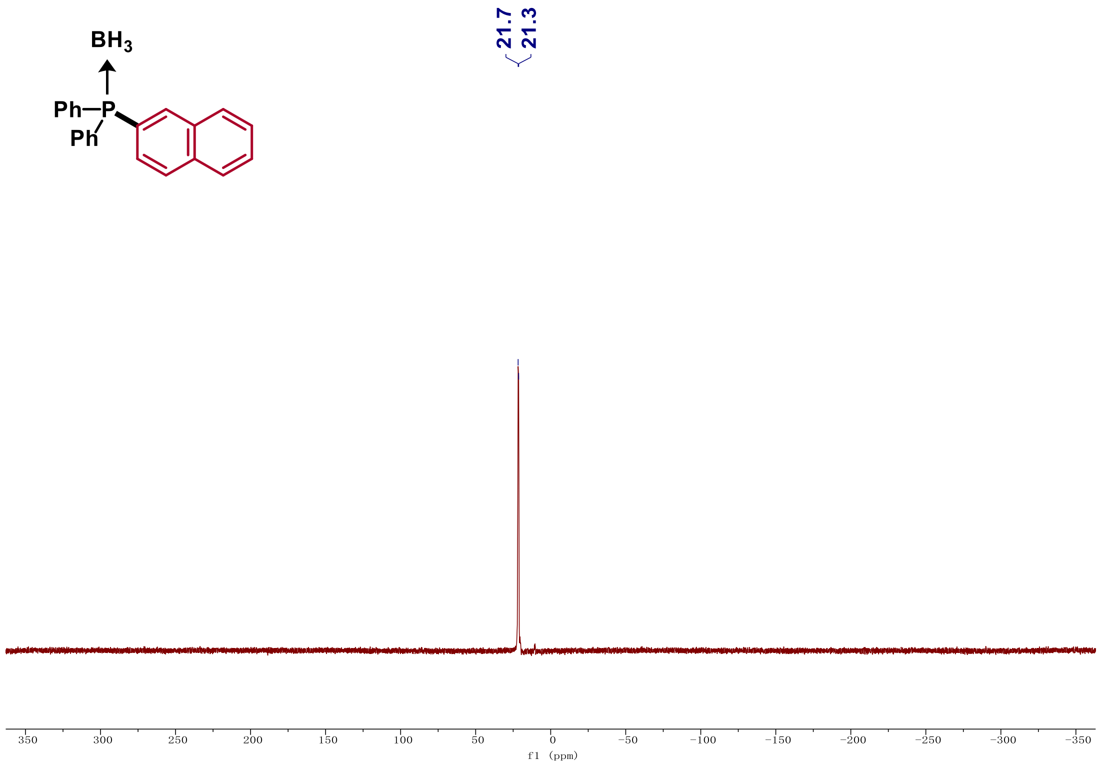

^{1}H NMR of compound **13** (500 MHz in $CDCl_3$)

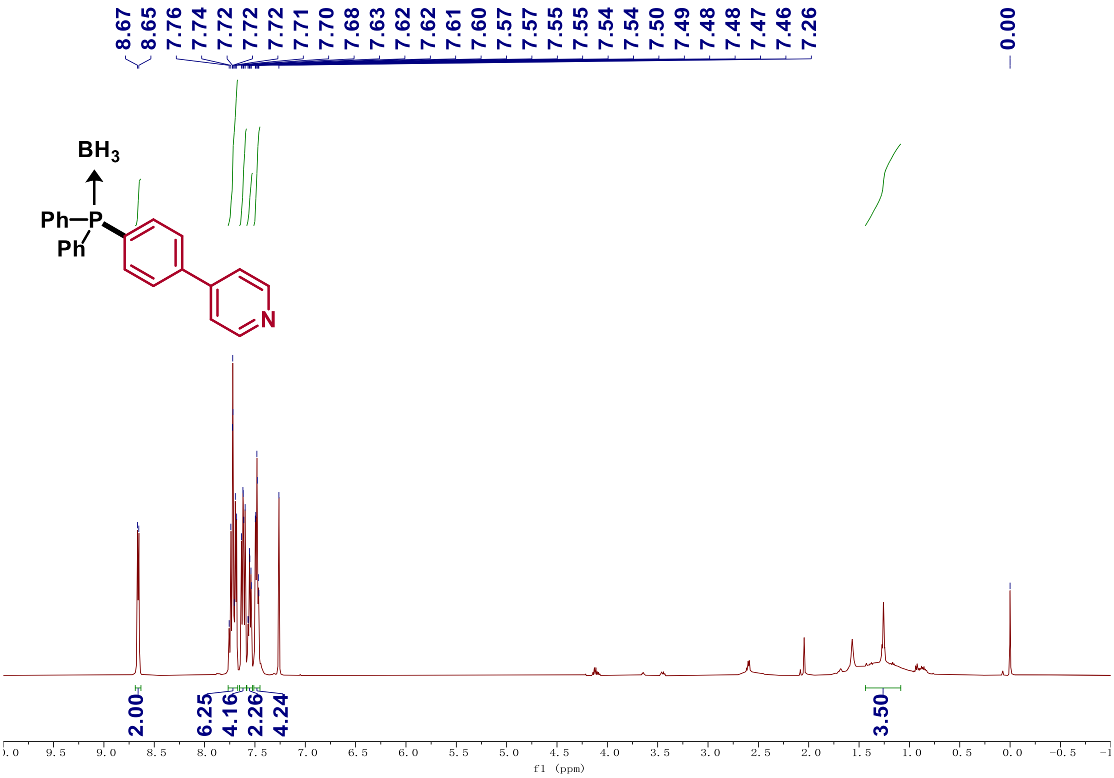

^{13}C NMR of compound **13** (126 MHz in $CDCl_3$)

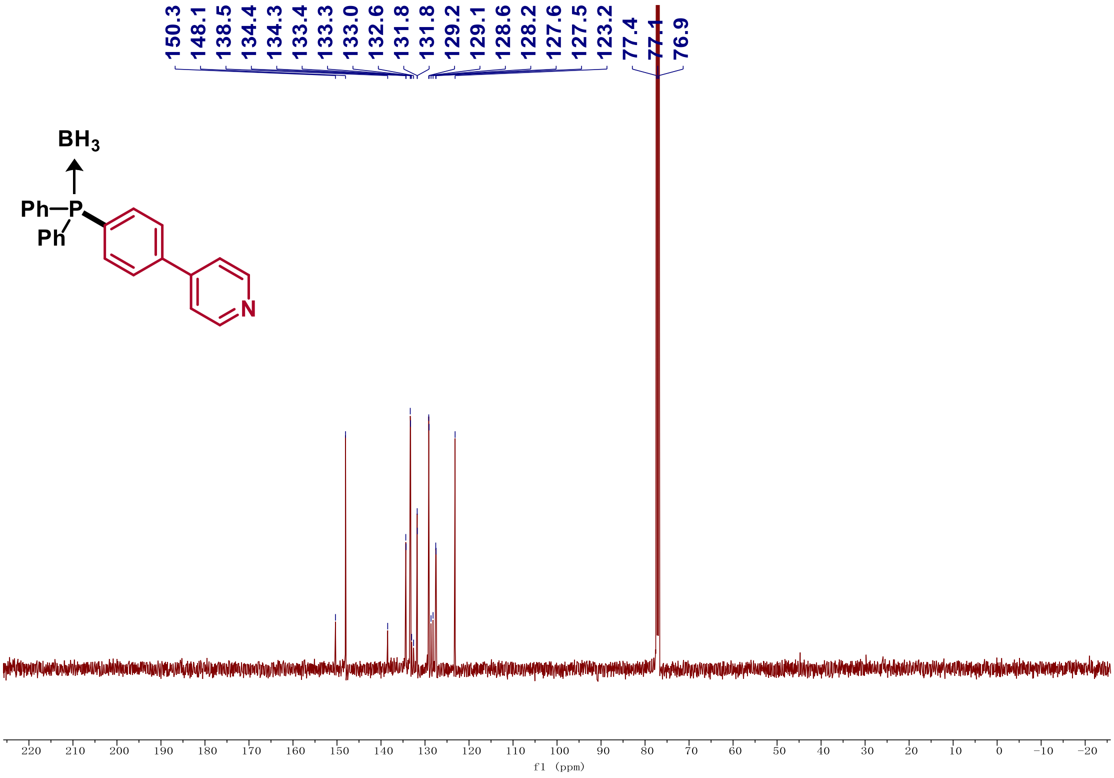

^{11}B **NMR** of compound **13** (160 MHz in $CDCl_3$)

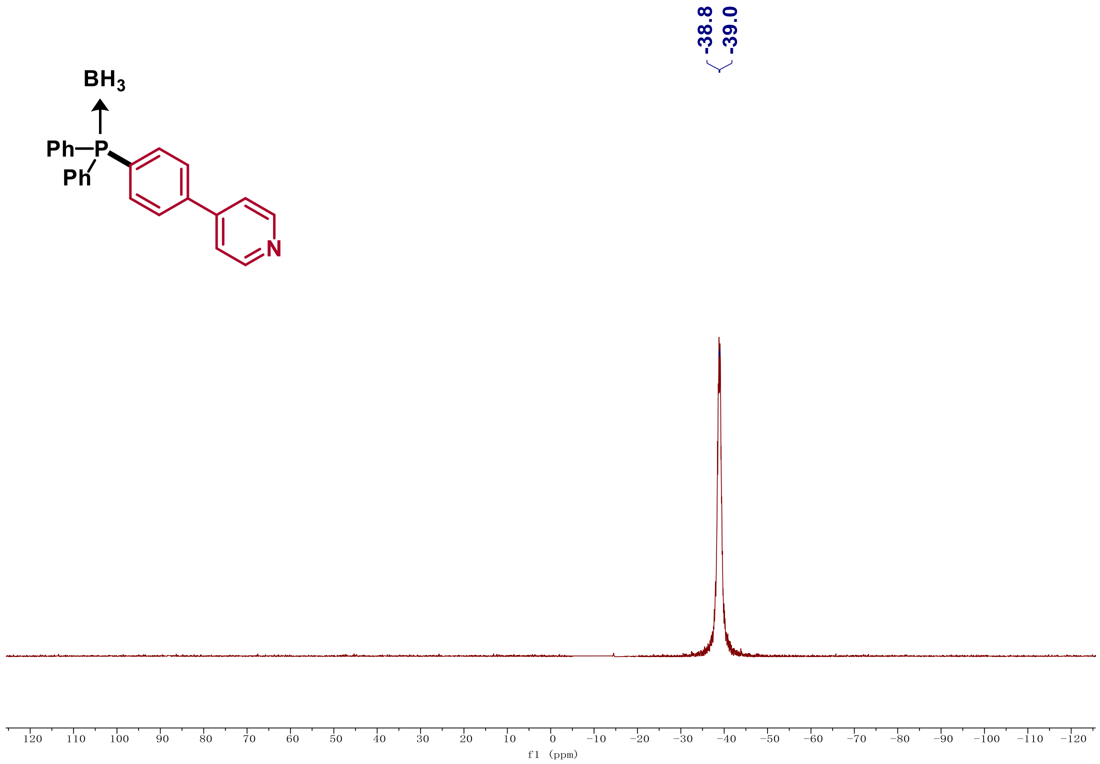

^{31}P **NMR** of compound **13** (202 MHz in $CDCl_3$)

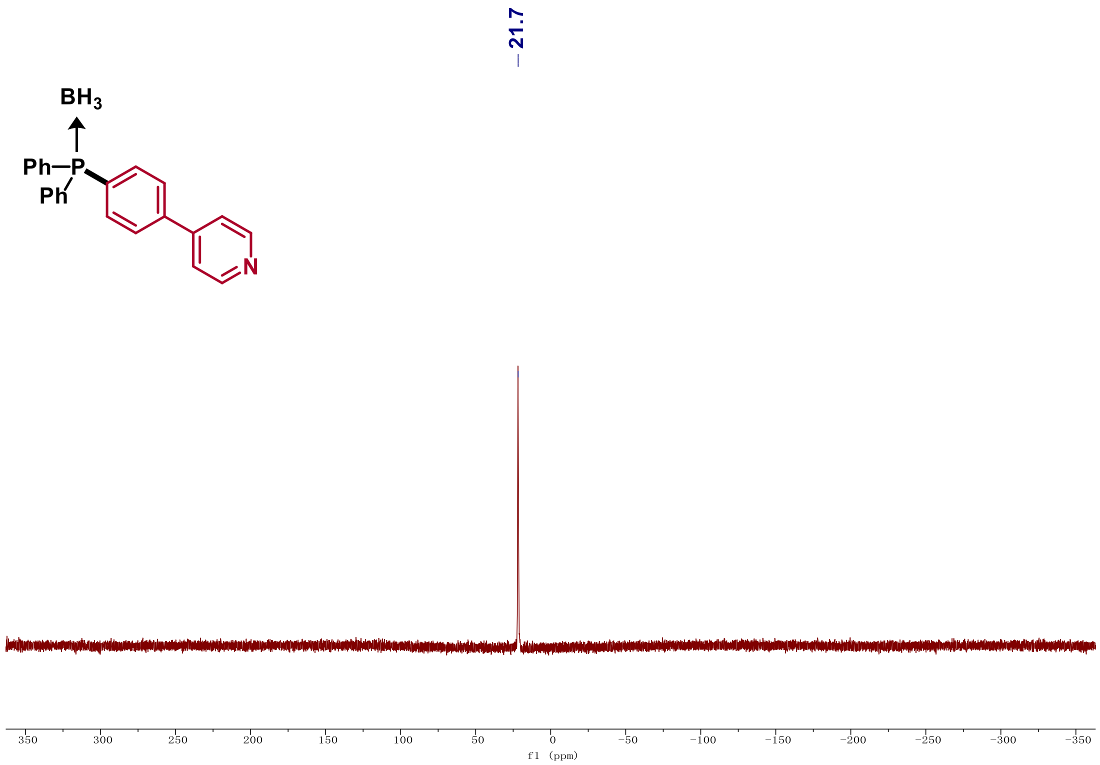

^{1}H NMR of compound **14** (500 MHz in $CDCl_3$)

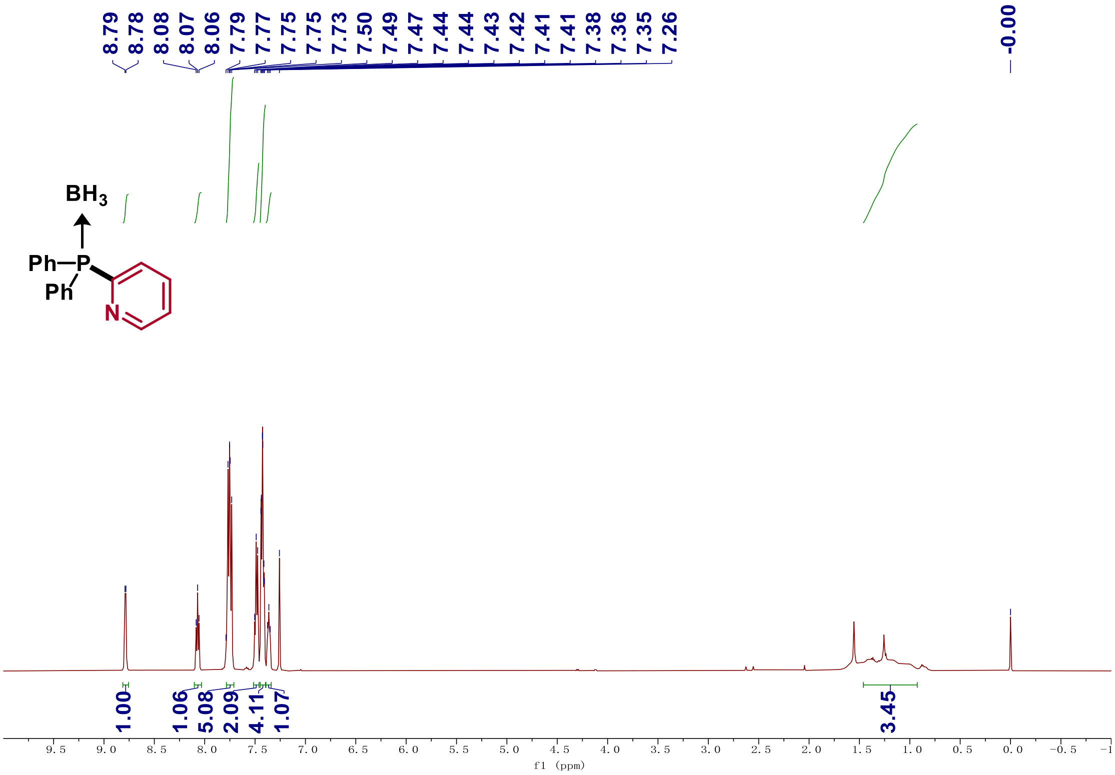

^{13}C NMR of compound **14** (126 MHz in $CDCl_3$)

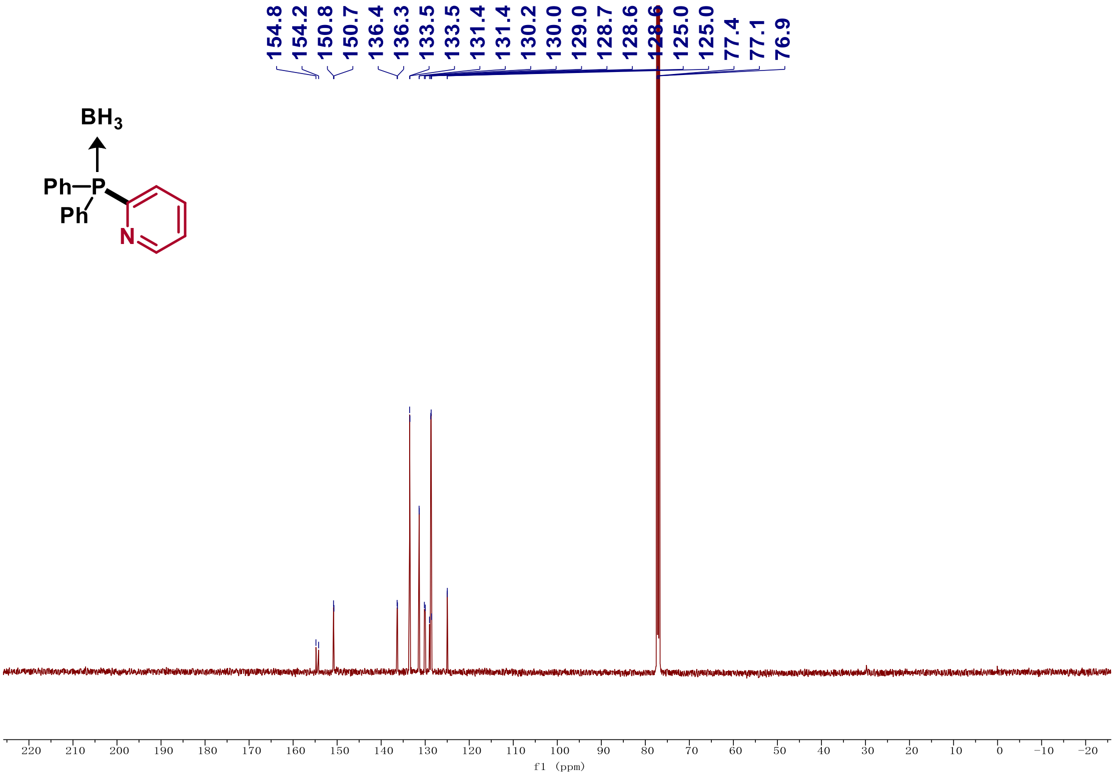

^{11}**B NMR** of compound **14** (160 MHz in $CDCl_3$)

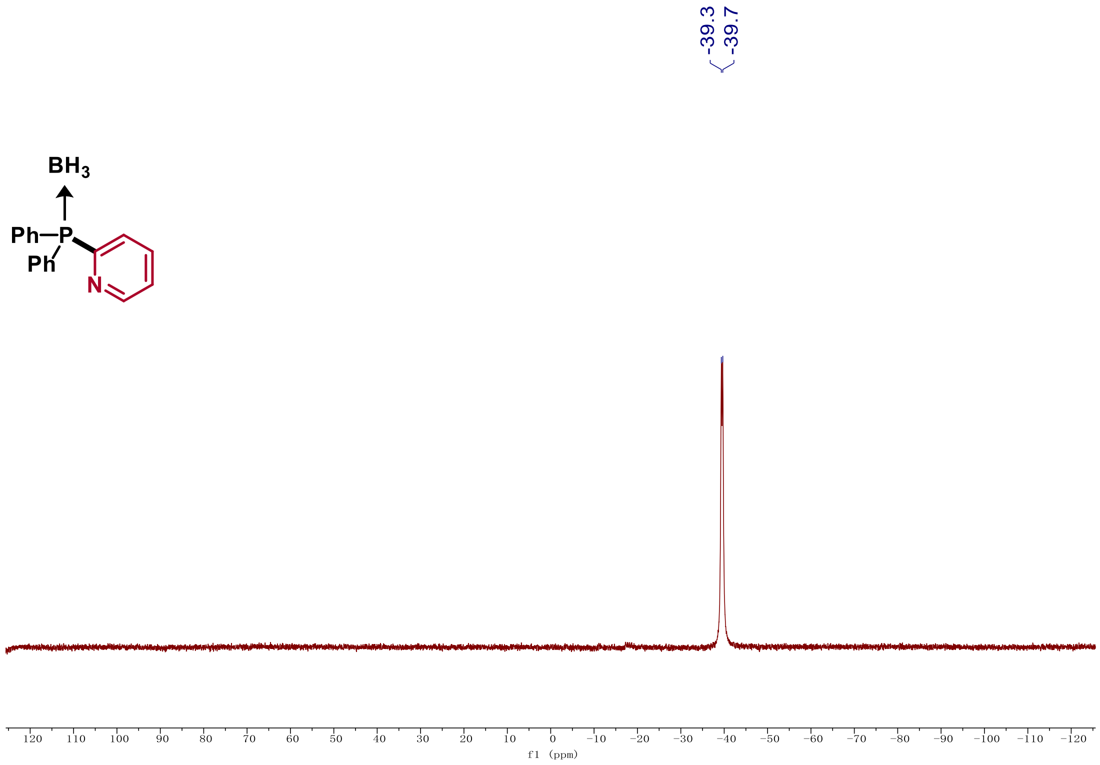

^{31}**P NMR** of compound **14** (202 MHz in $CDCl_3$)

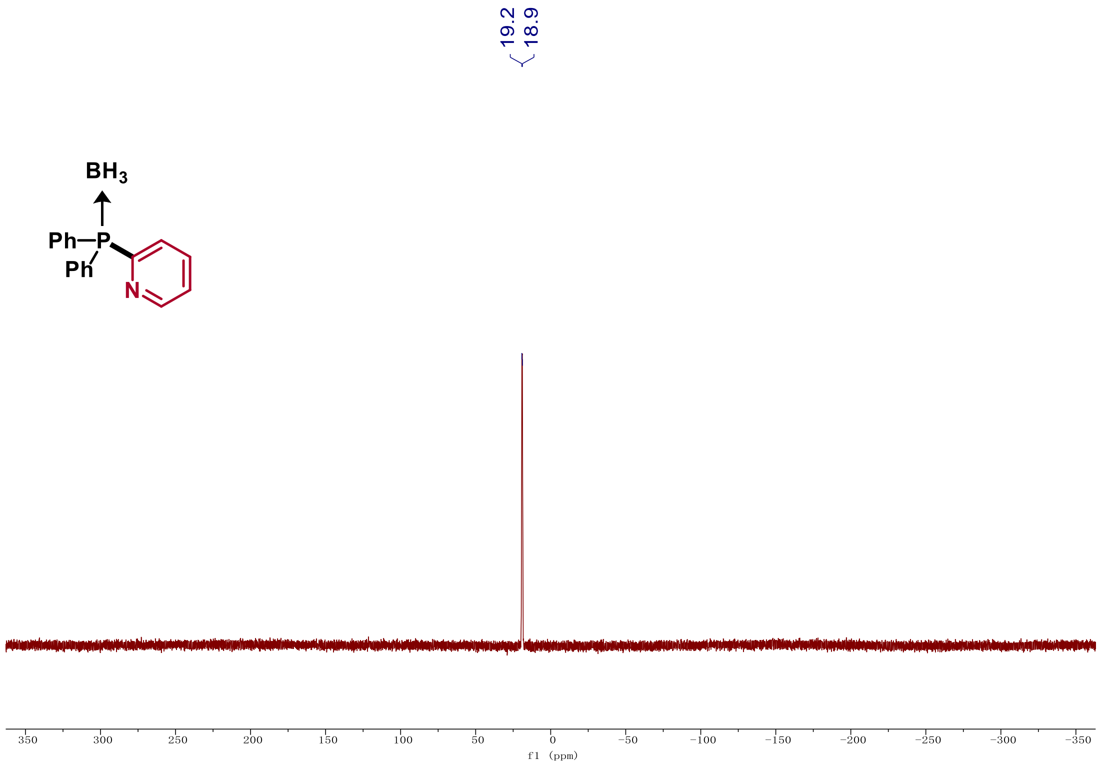

¹H NMR of compound **15** (500 MHz in CDCl₃)

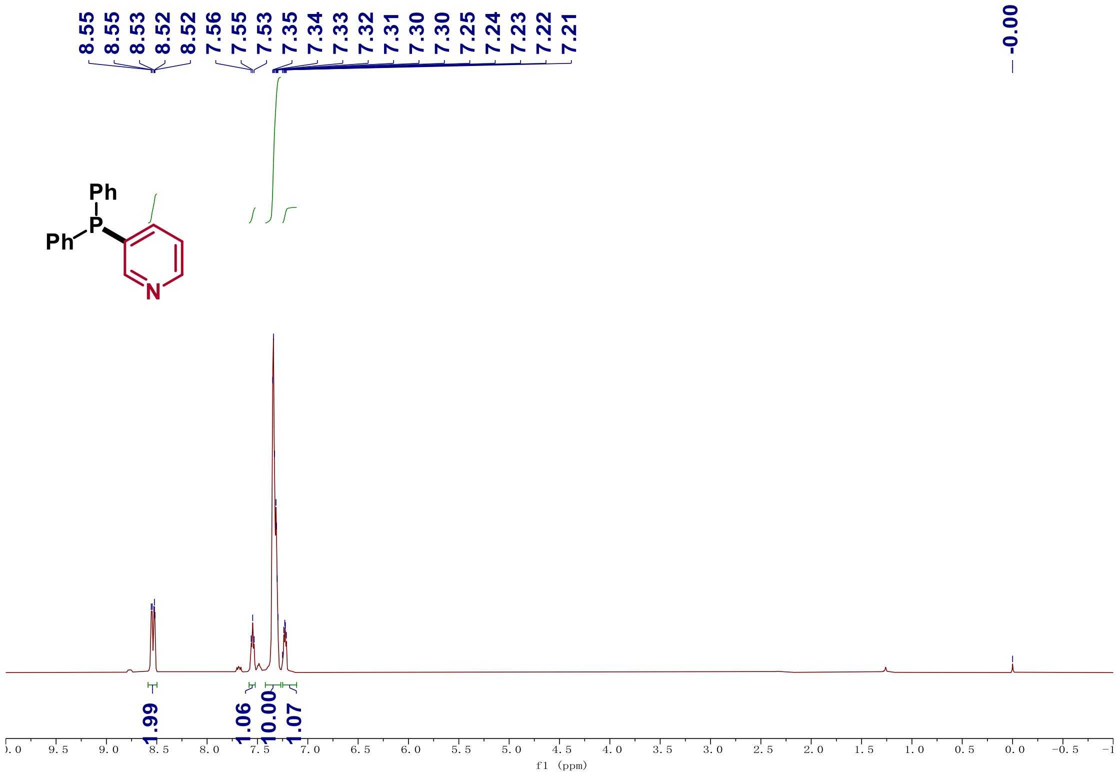

¹³C NMR of compound **15** (126 MHz in CDCl₃)

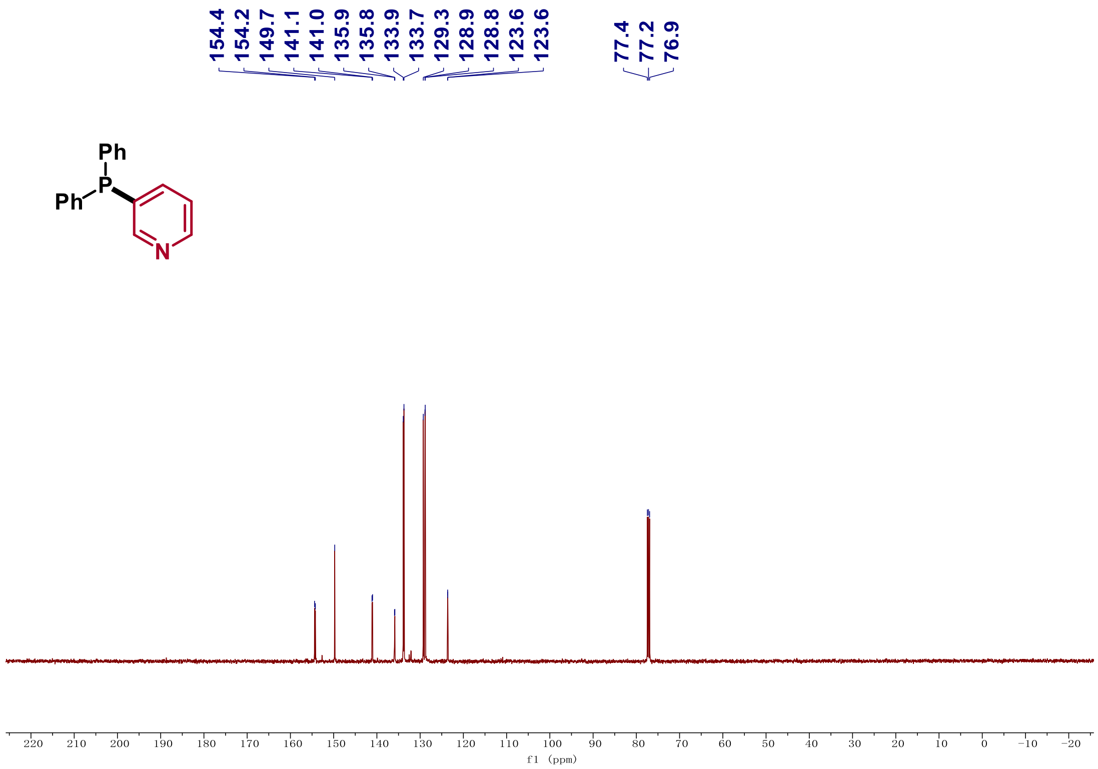

^{31}P NMR of compound **15** (202 MHz in $CDCl_3$)

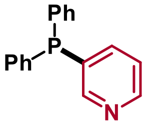

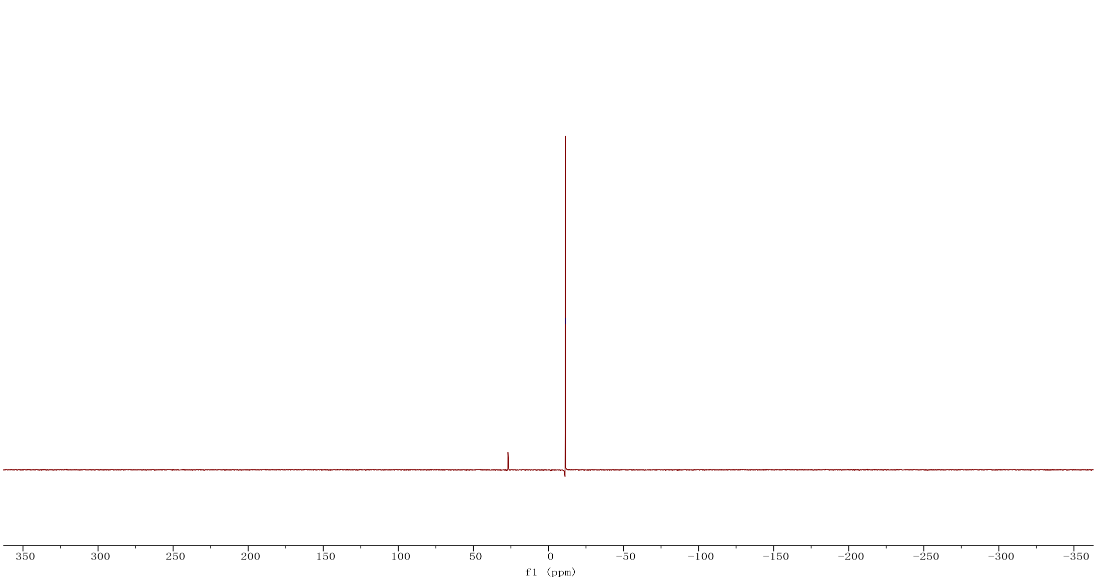

¹H NMR of compound **16** (500 MHz in $CDCl_3$)

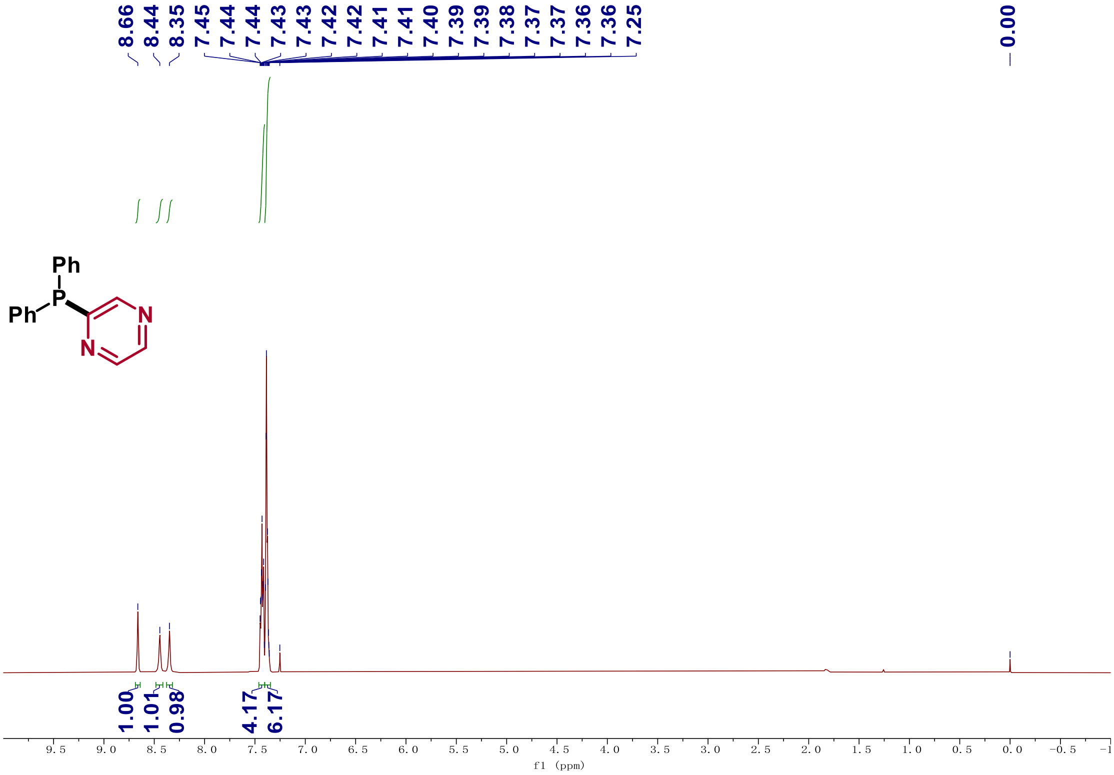

¹³C NMR of compound **16** (126 MHz in $CDCl_3$)

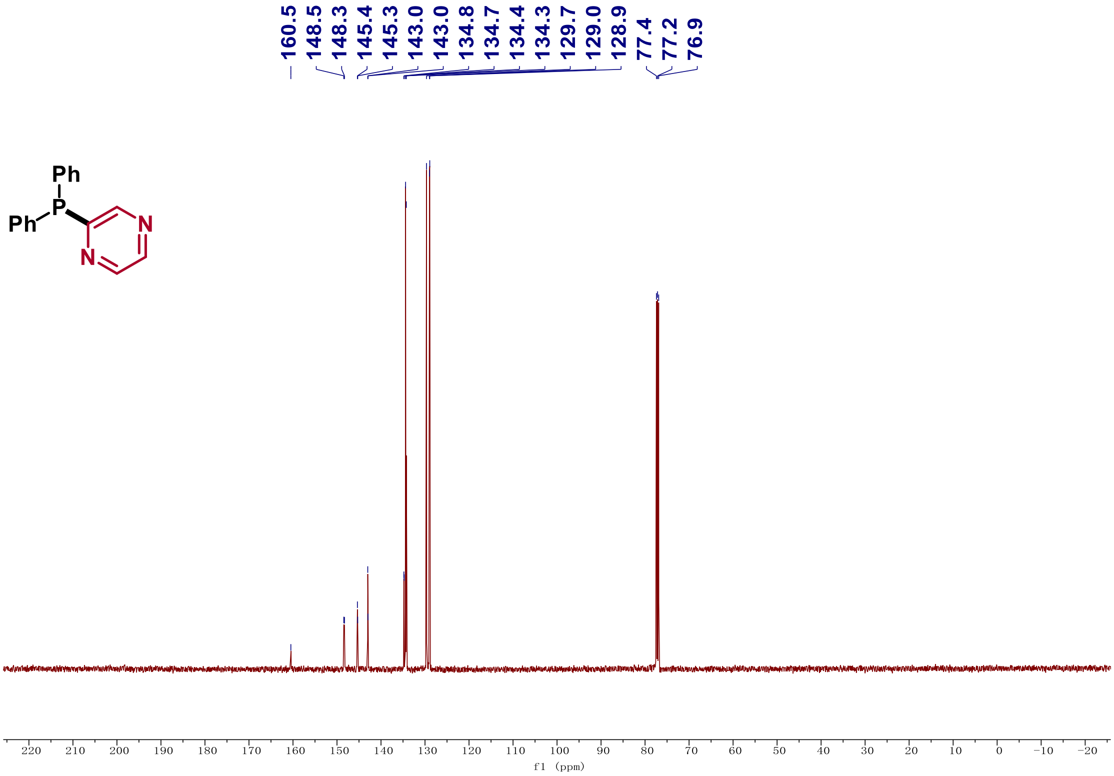

^{31}P NMR of compound **16** (202 MHz in $CDCl_3$)

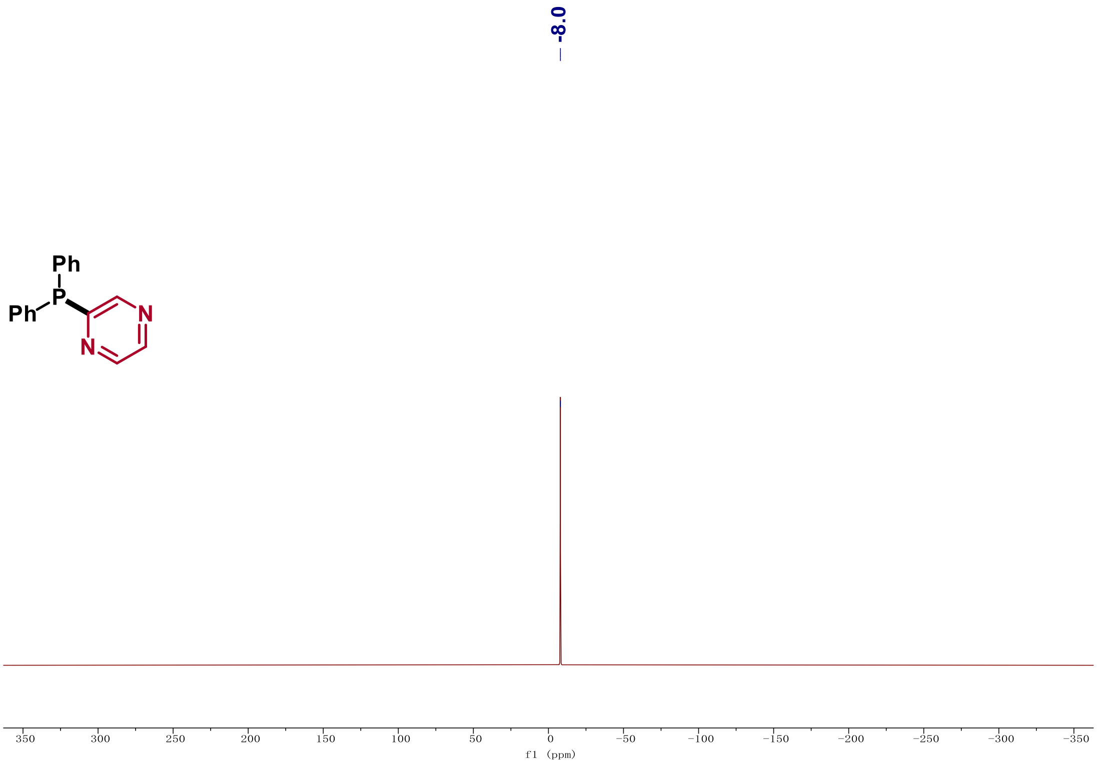

^{1}H NMR of compound 17 (500 MHz in $CDCl_3$)

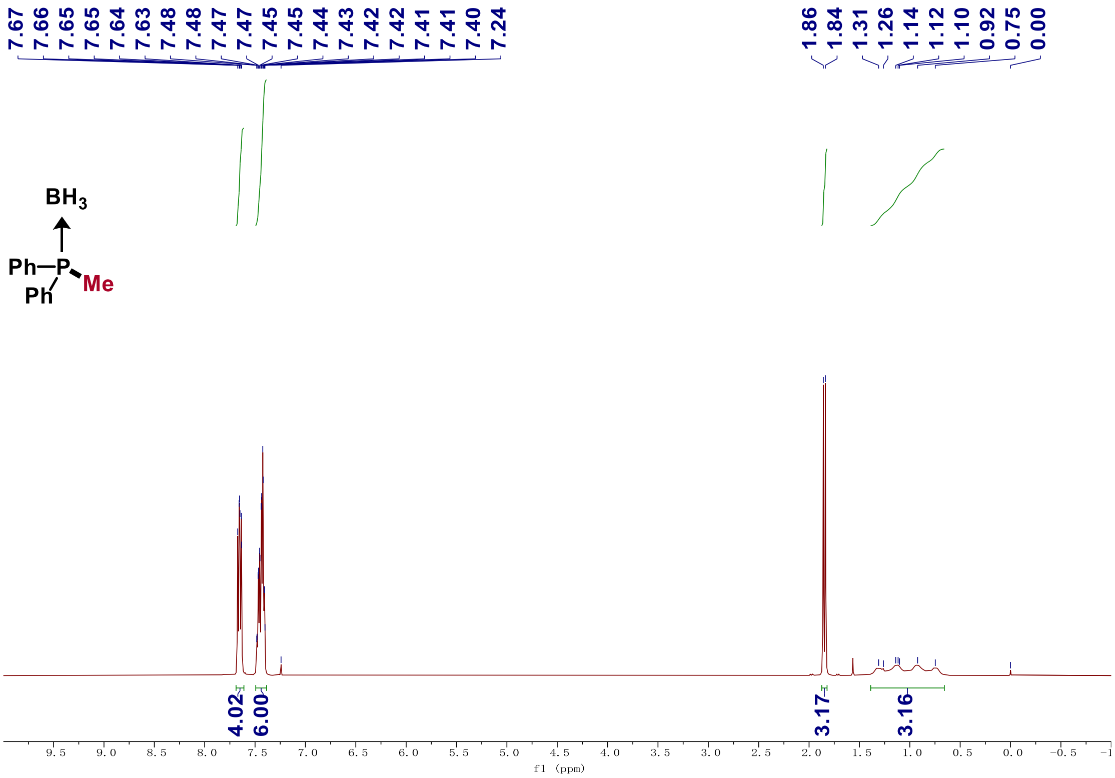

^{13}C NMR of compound 17 (126 MHz in $CDCl_3$)

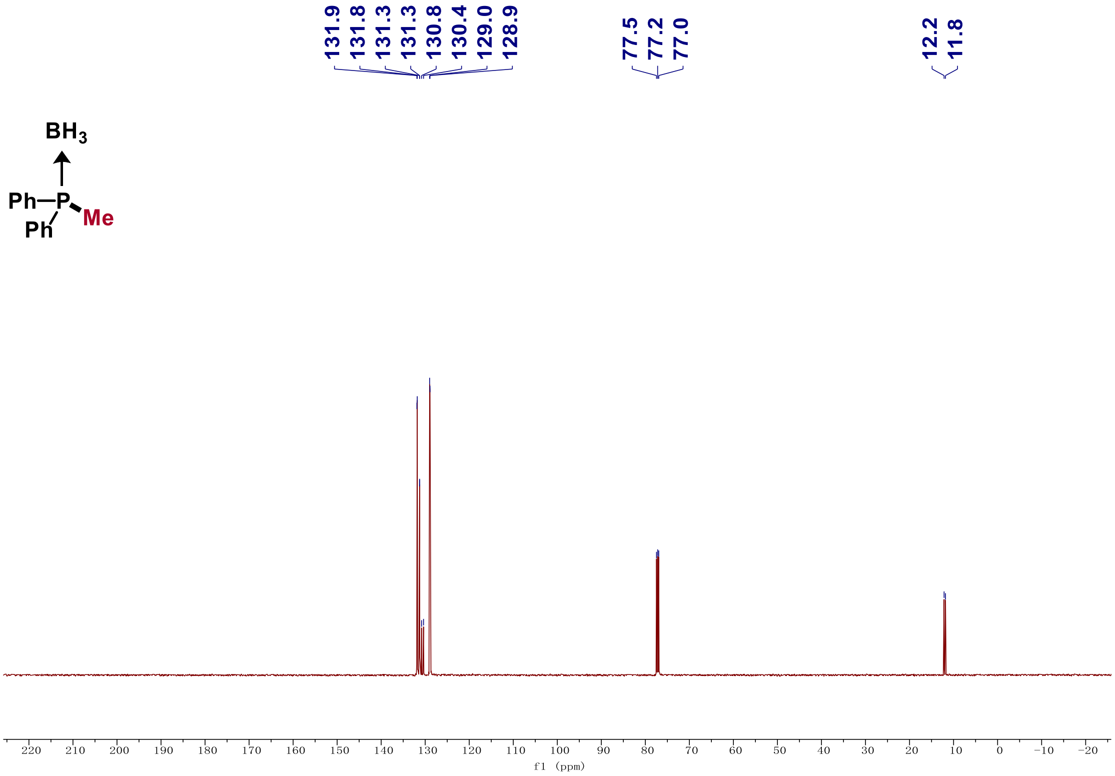

[11]**B NMR** of compound **17** (160 MHz in $CDCl_3$)

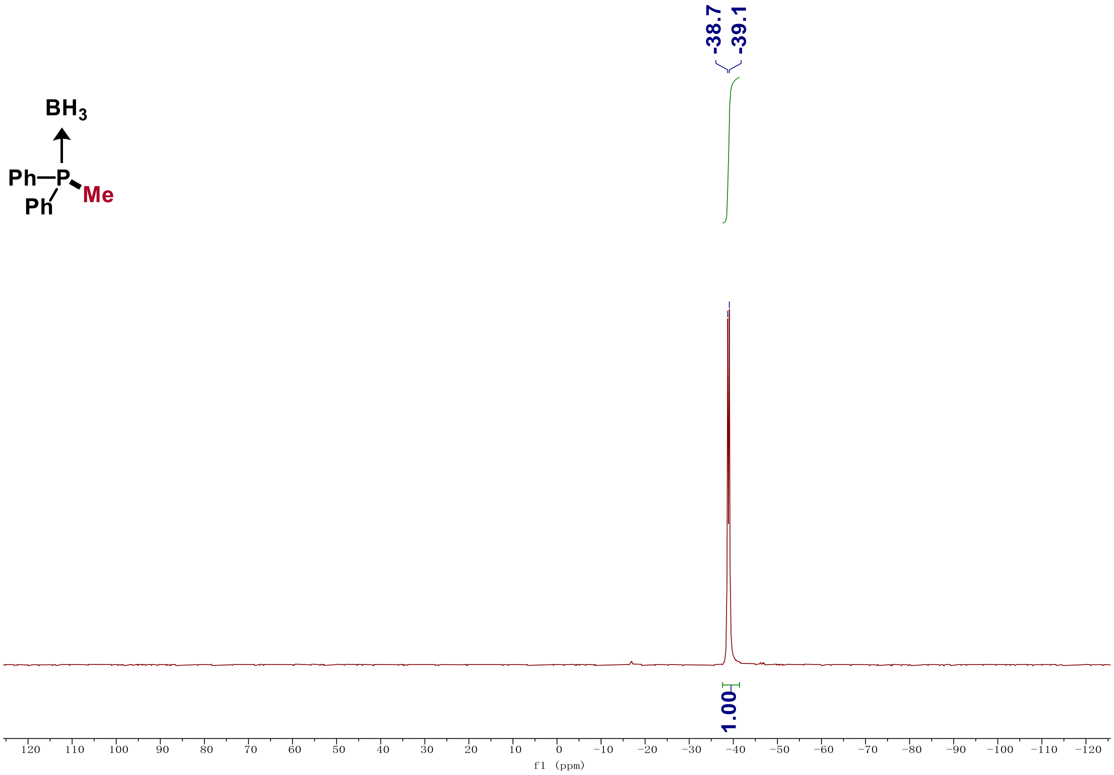

[31]**P NMR** of compound **17** (202 MHz in $CDCl_3$)

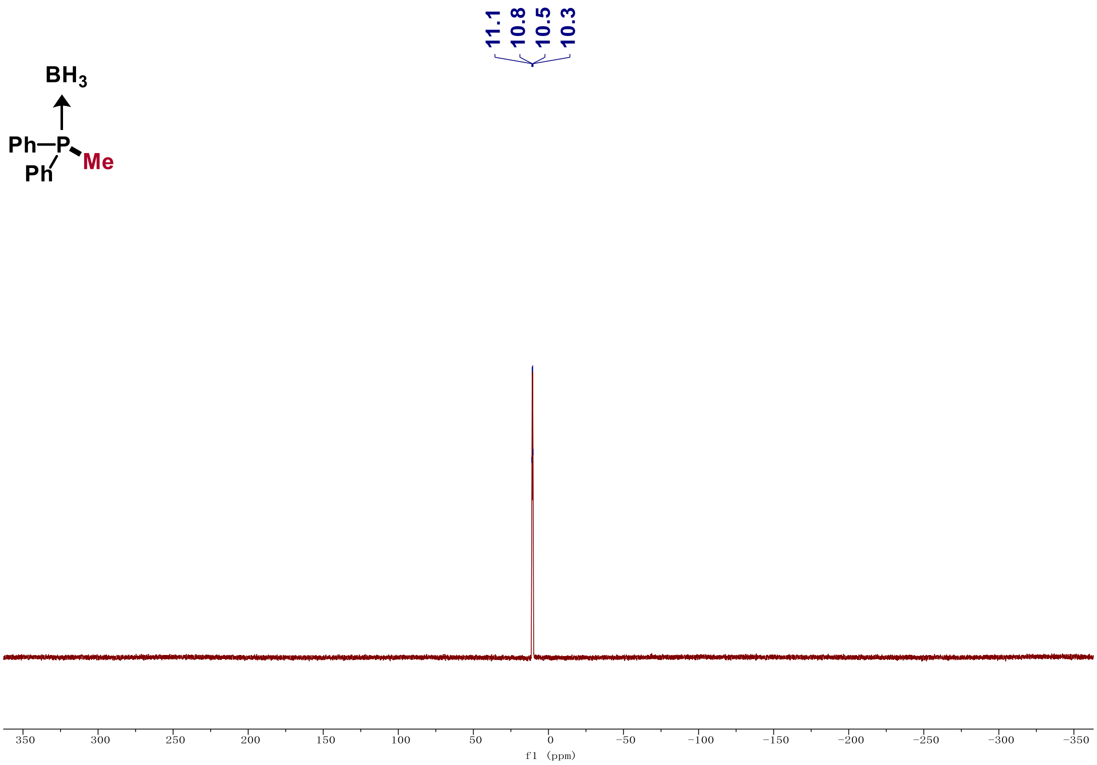

^{1}H NMR of compound **18** (400 MHz in $CDCl_3$)

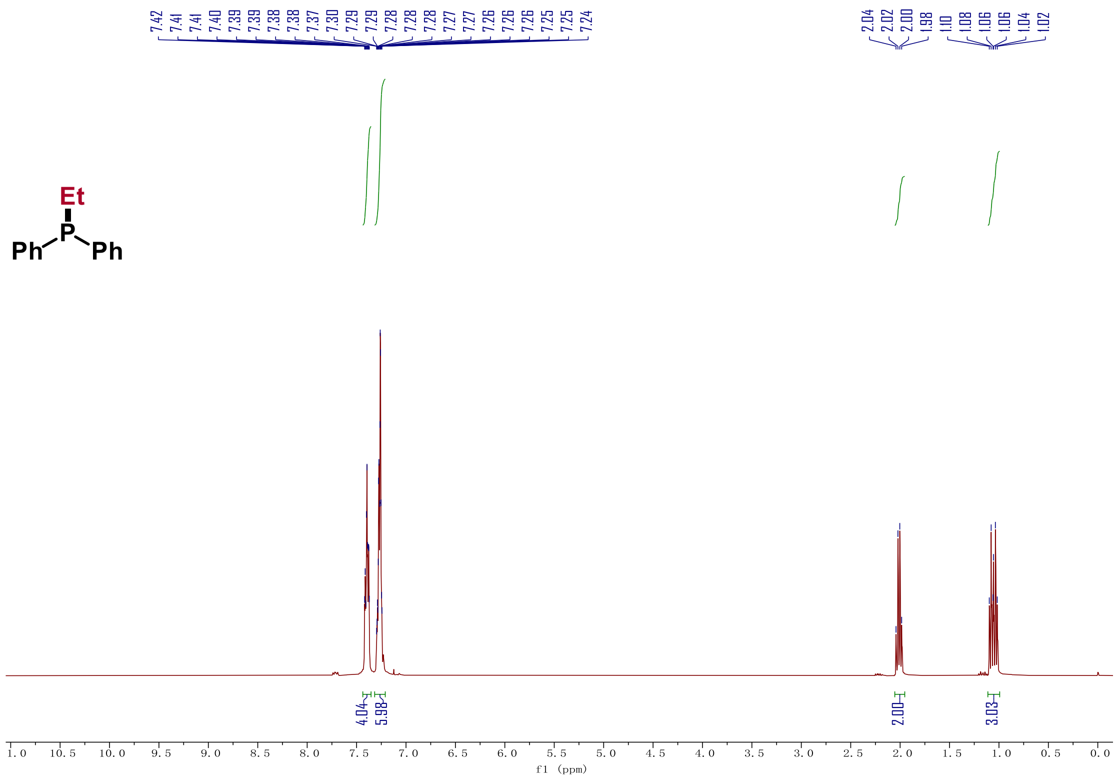

^{13}C NMR of compound **18** (101 MHz in $CDCl_3$)

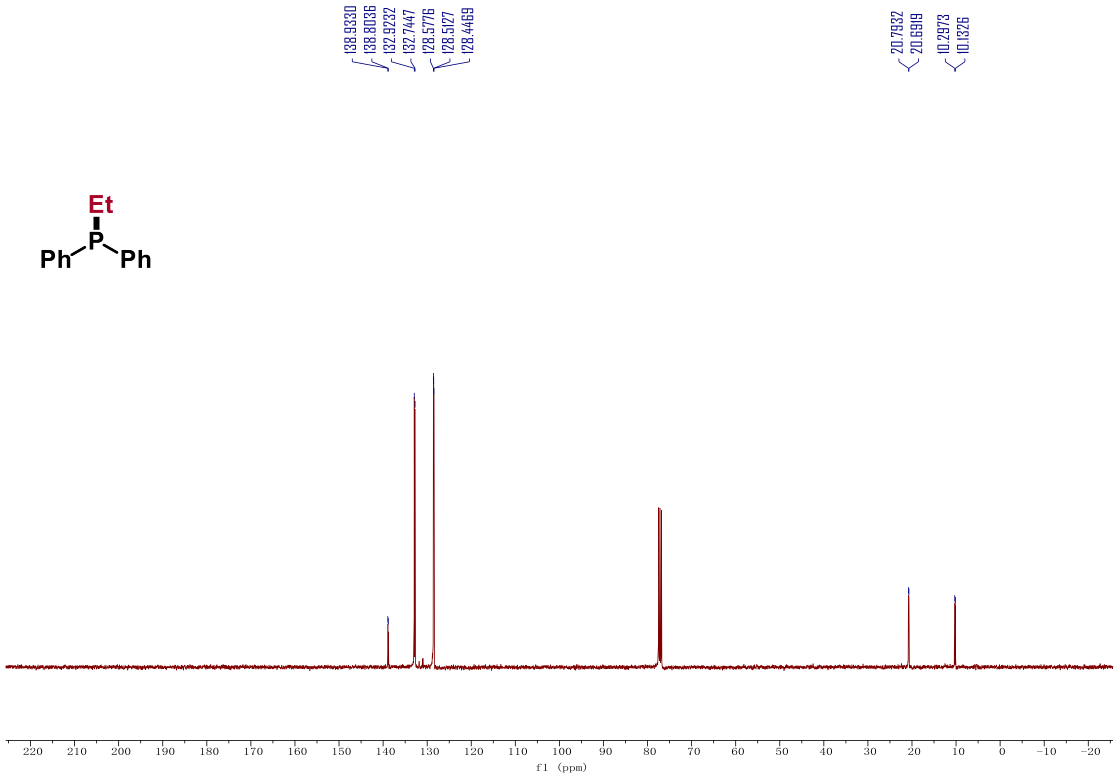

^{31}P NMR of compound **18** (202 MHz in $CDCl_3$)

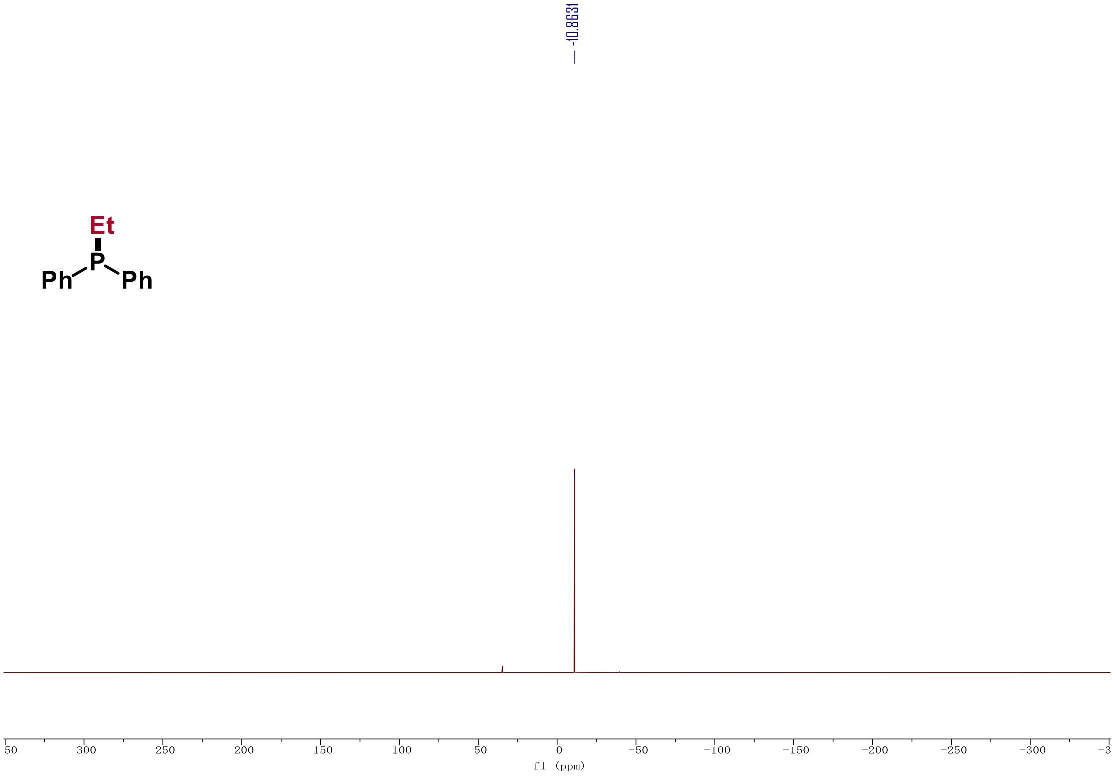

^{1}H NMR of compound **19** (500 MHz in $CDCl_3$)

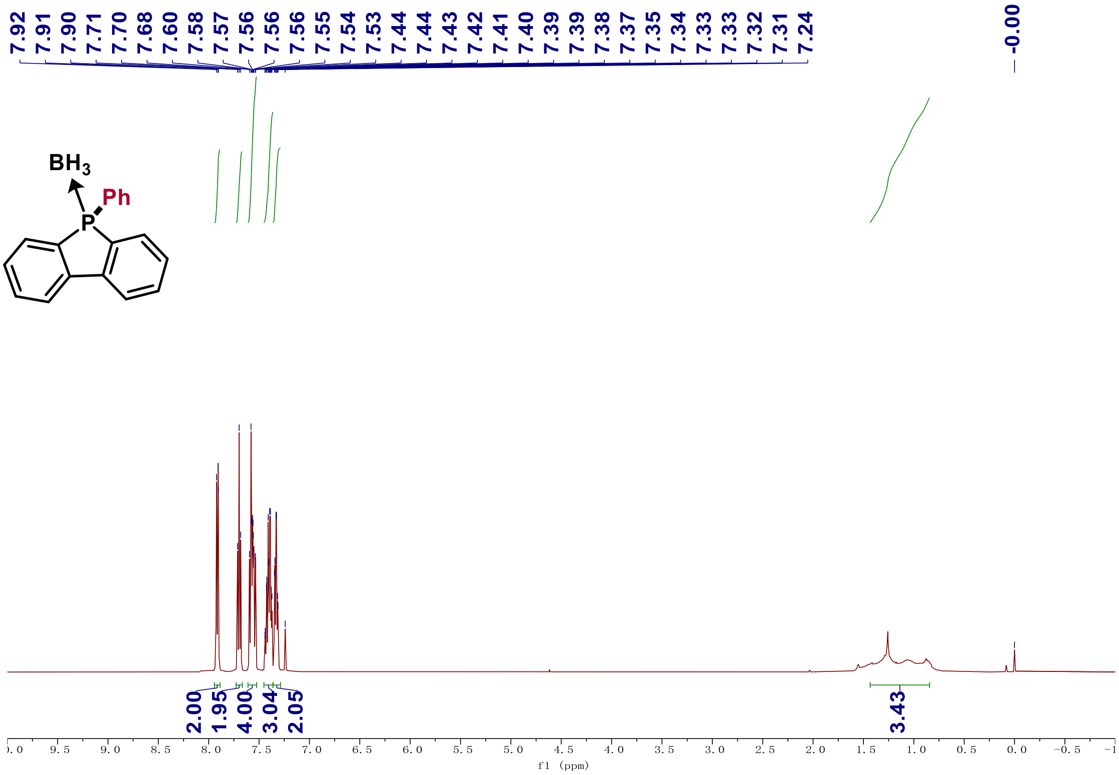

^{13}C NMR of compound **19** (126 MHz in $CDCl_3$)

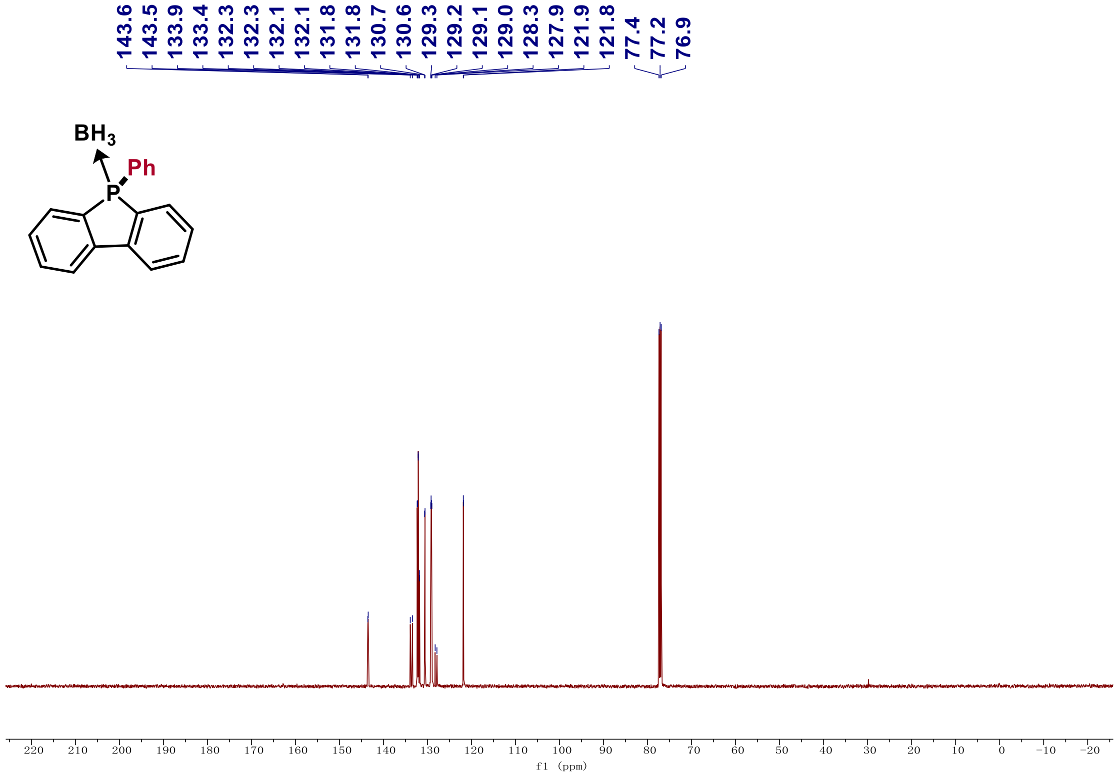

^{11}**B NMR** of compound **19** (160 MHz in $CDCl_3$)

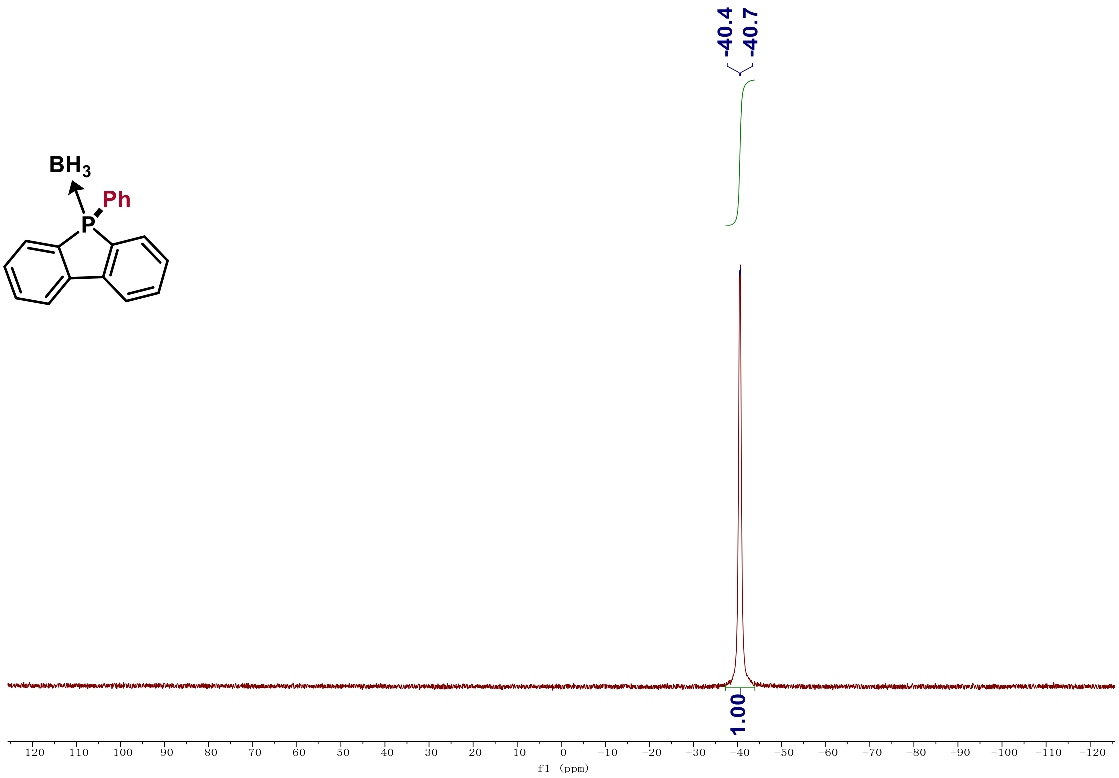

^{31}**P NMR** of compound **19** (202 MHz in $CDCl_3$)

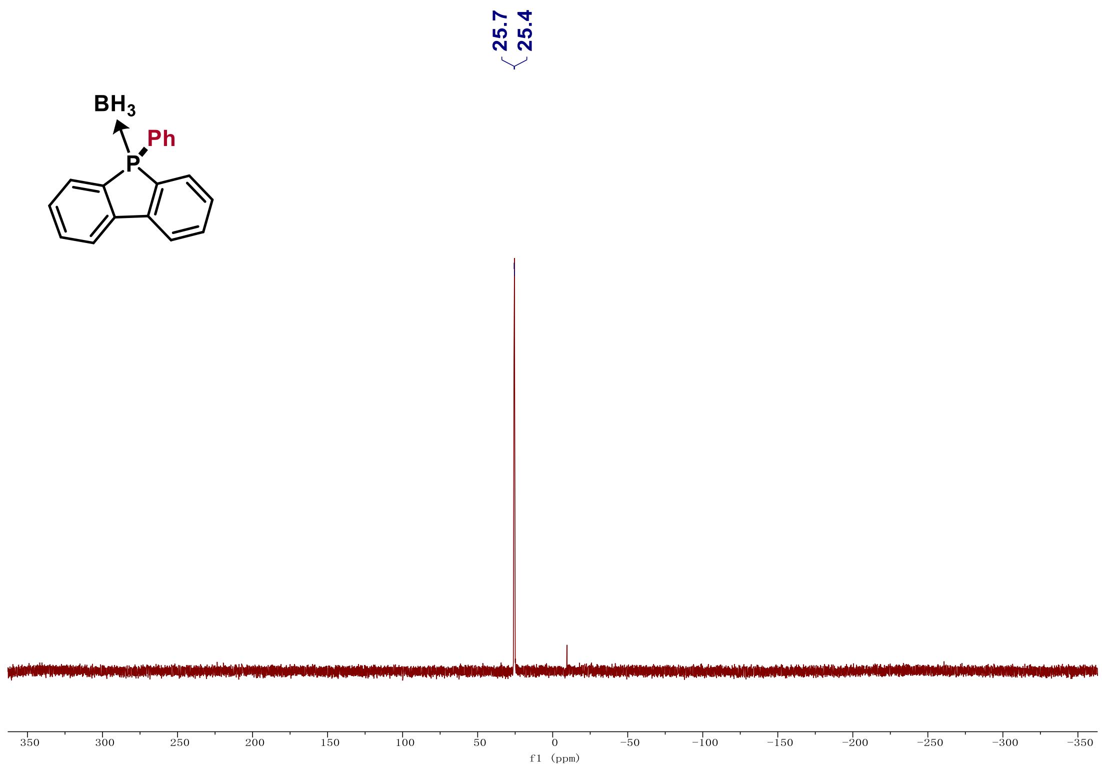

^{1}H NMR of compound **20** (500 MHz in $CDCl_3$)

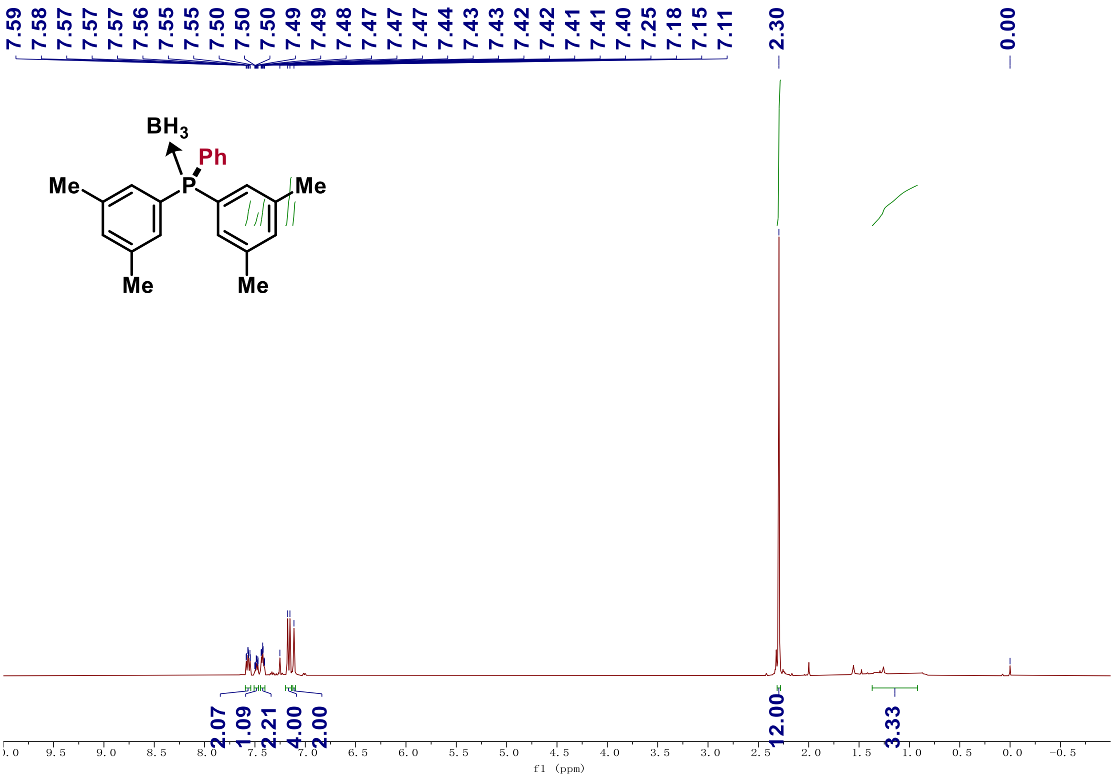

^{13}C NMR of compound **20** (126 MHz in $CDCl_3$)

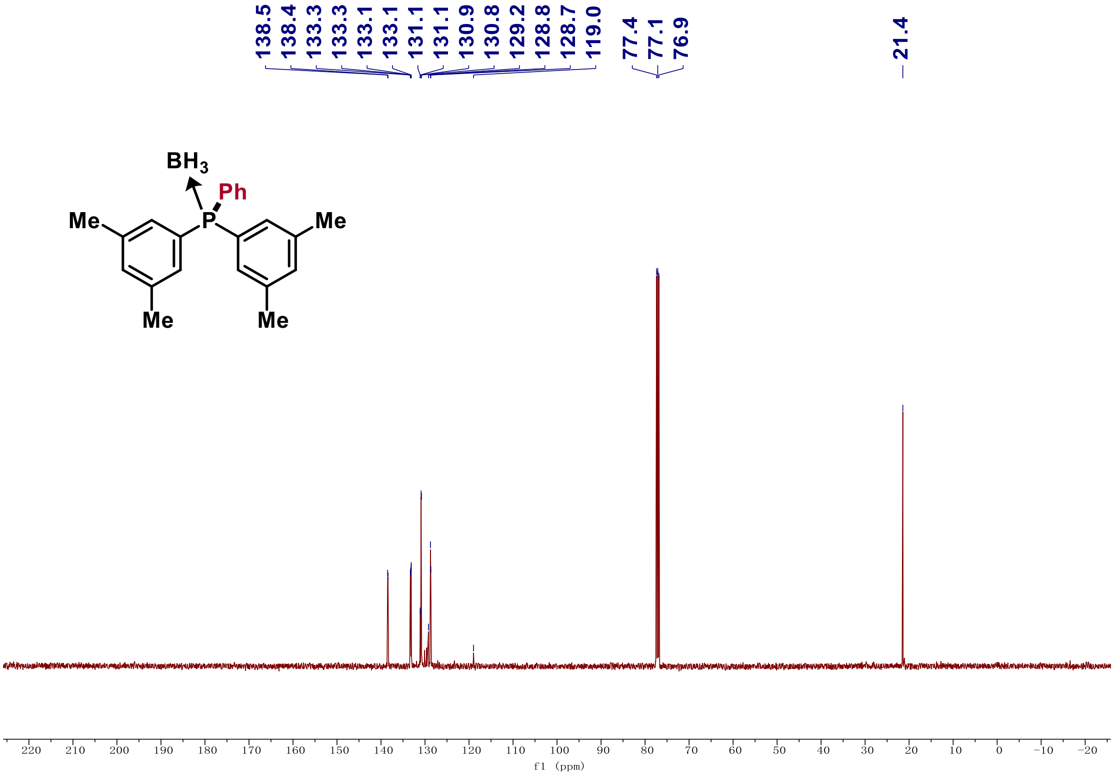

^{11}B NMR of compound **20** (160 MHz in $CDCl_3$)

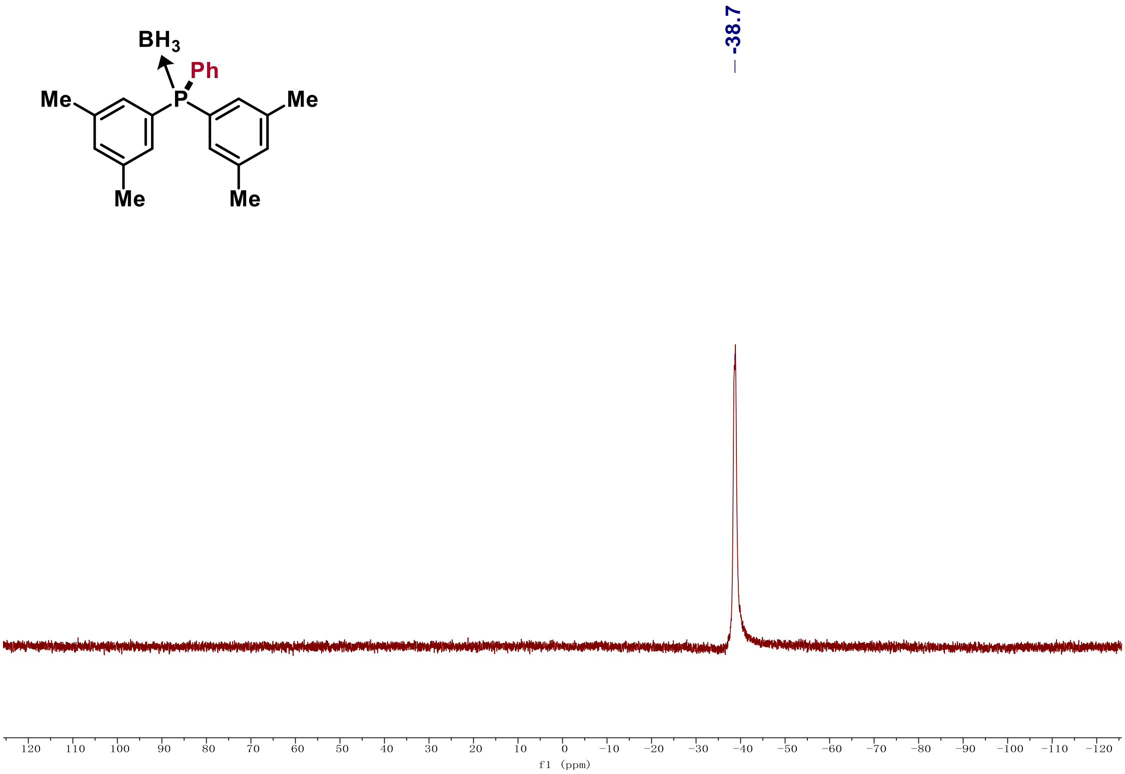

^{31}P NMR of compound **20** (202 MHz in $CDCl_3$)

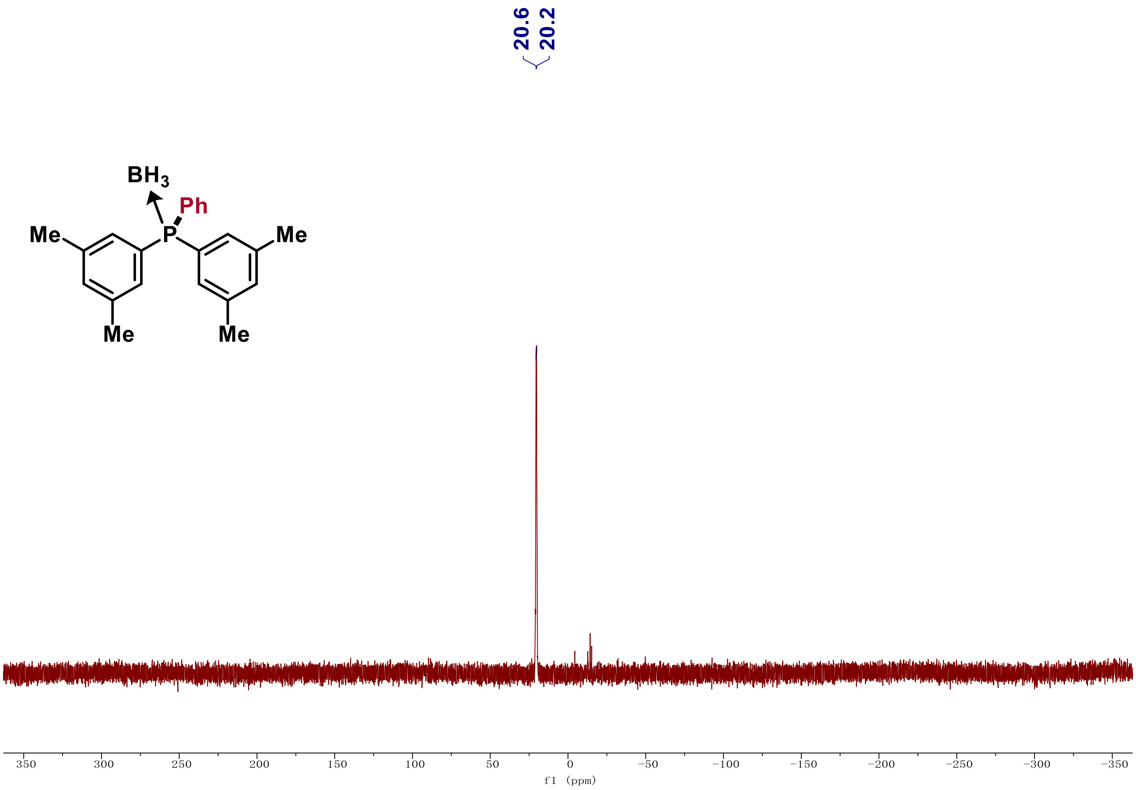

^{1}H NMR of compound **21** (500 MHz in $CDCl_3$)

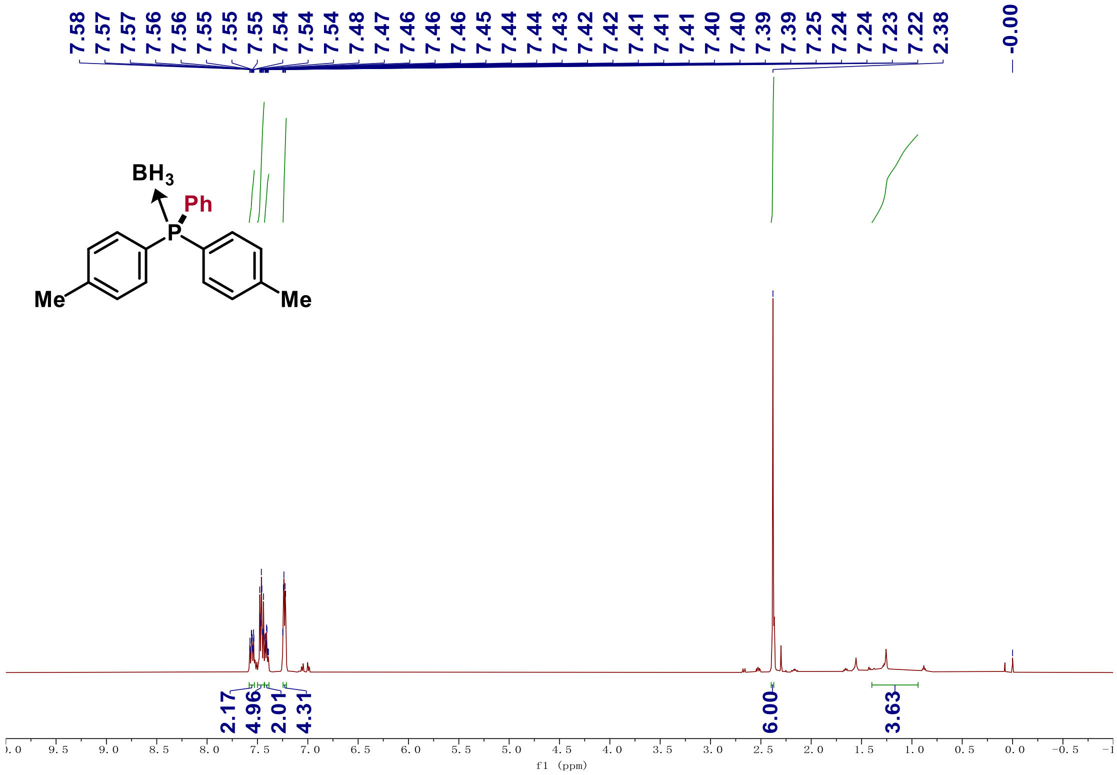

^{13}C NMR of compound **21** (126 MHz in $CDCl_3$)

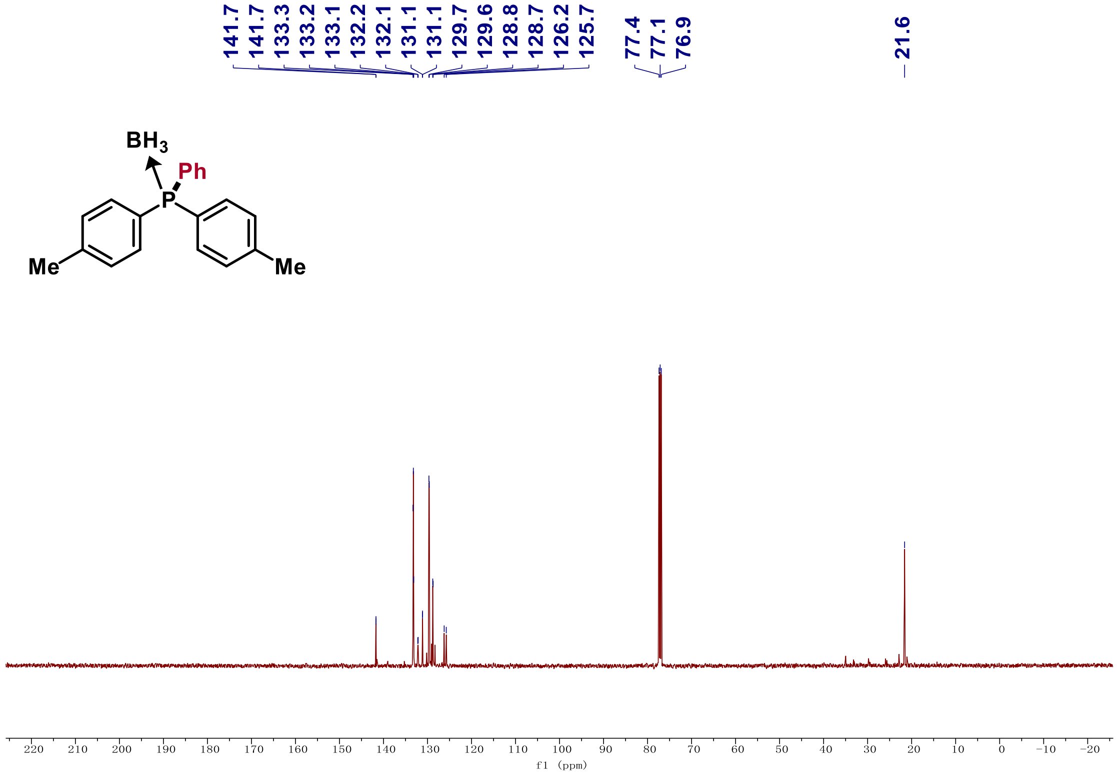

^{11}B NMR of compound **21** (160 MHz in $CDCl_3$)

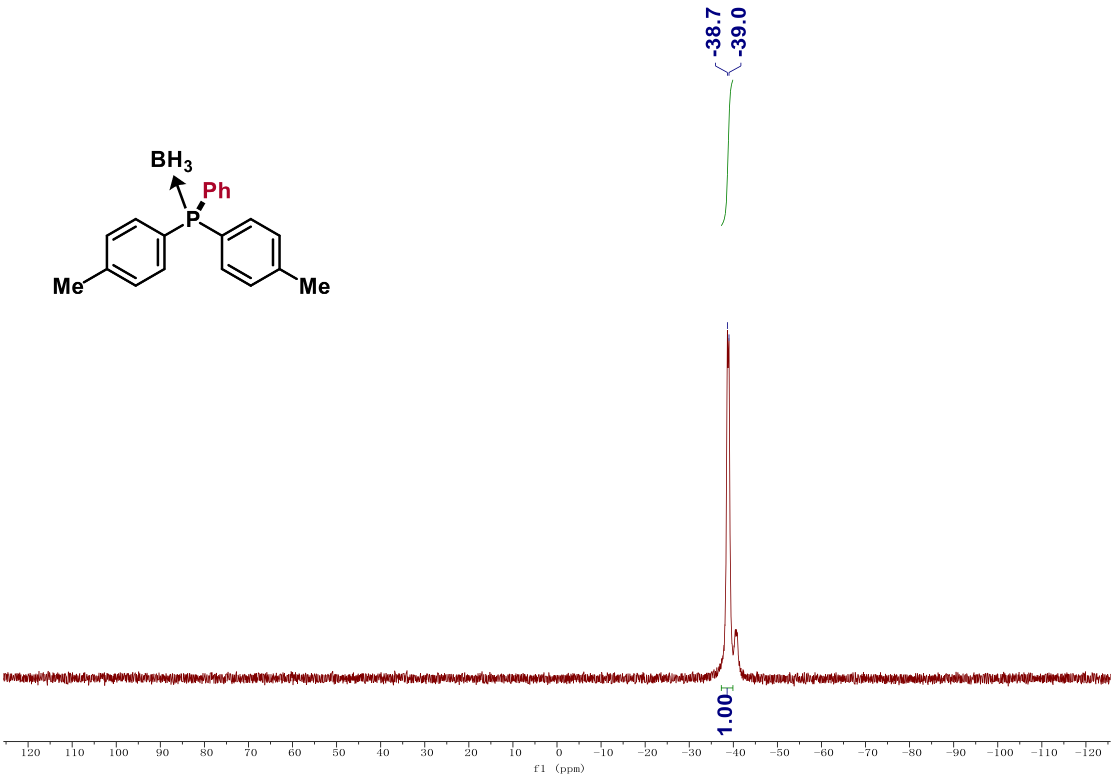

^{31}P NMR of compound **21** (202 MHz in $CDCl_3$)

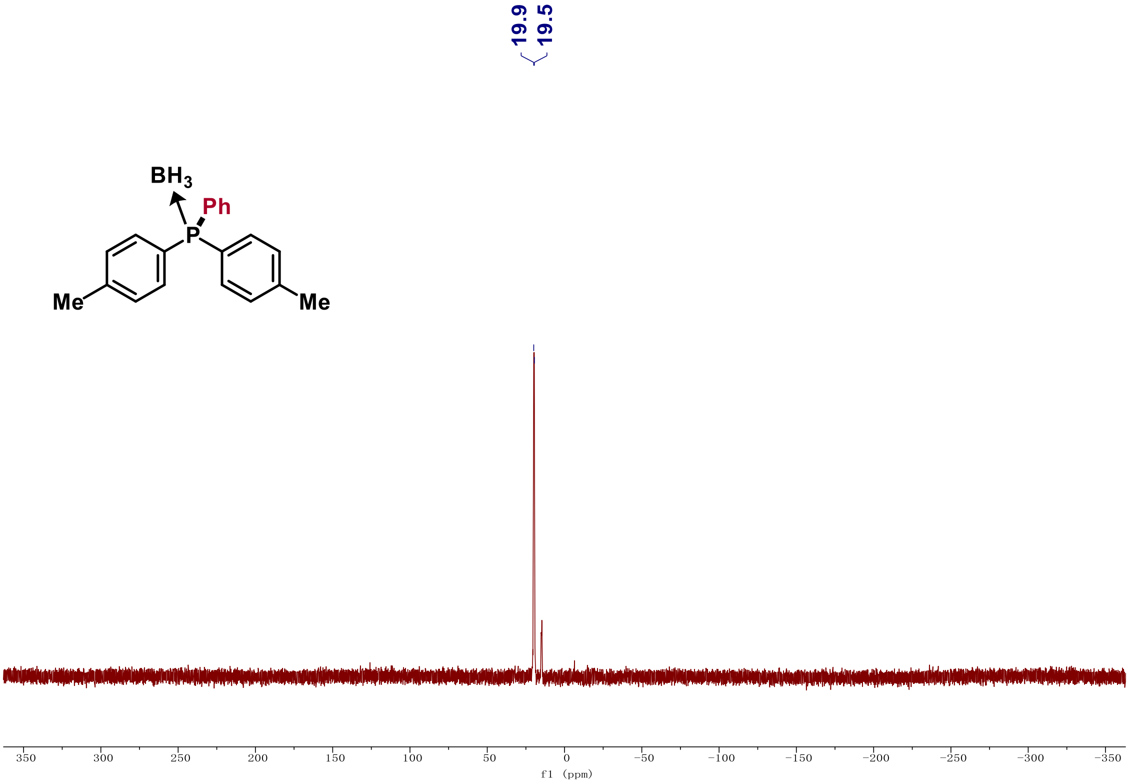

^{1}H NMR of compound **22** (500 MHz in $CDCl_3$)

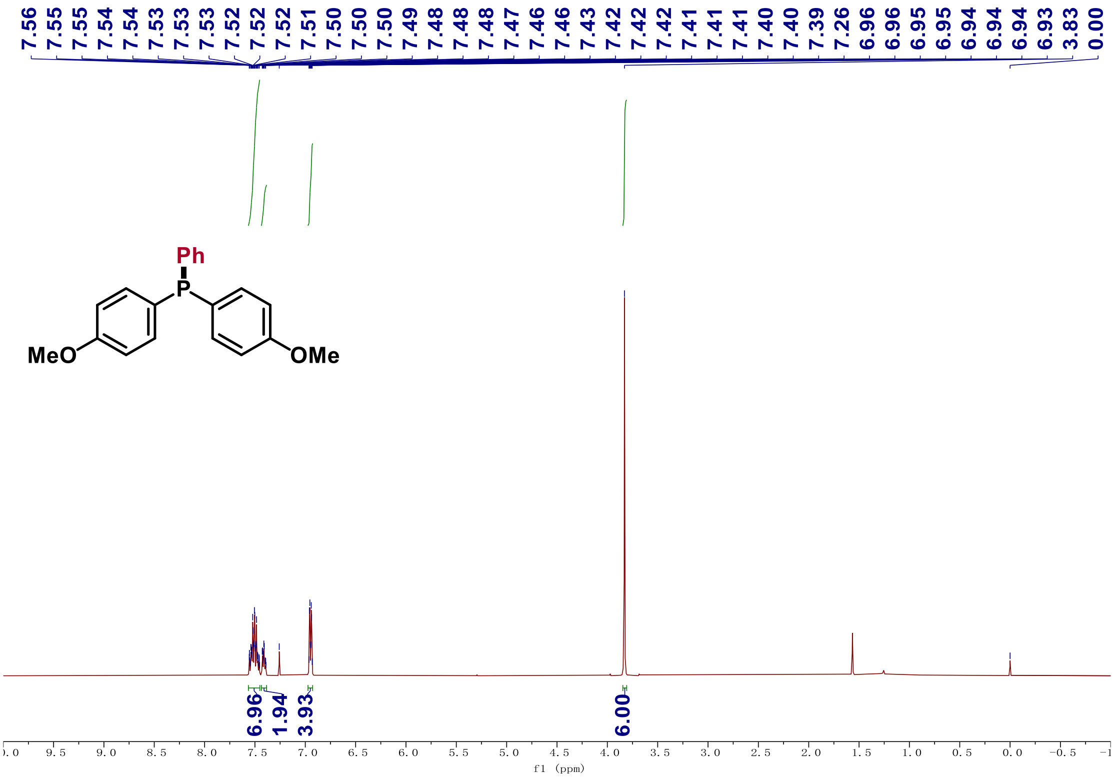

^{13}C NMR of compound **22** (126 MHz in $CDCl_3$)

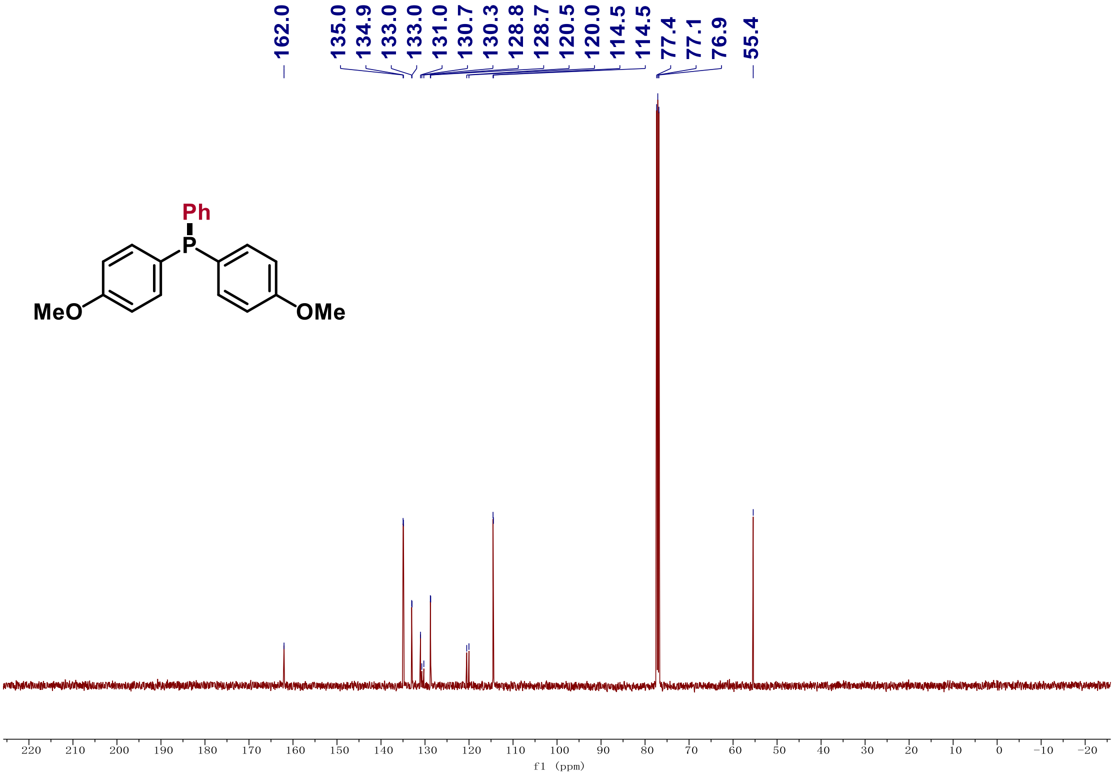

^{31}P NMR of compound **22** (202 MHz in $CDCl_3$)

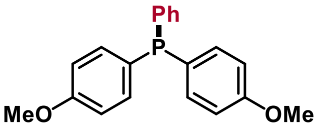

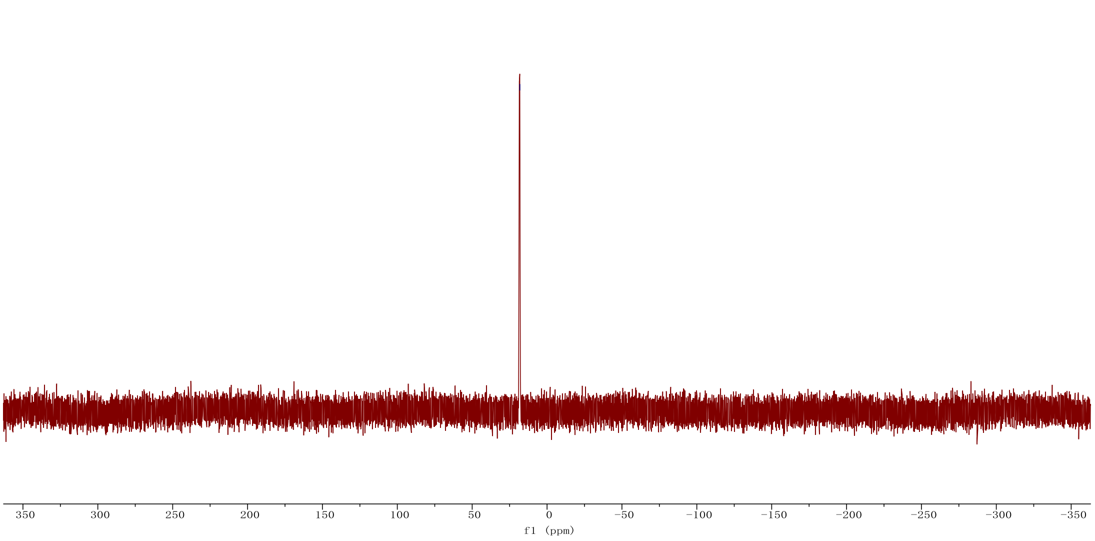

^{1}H NMR of compound **23** (400 MHz in $CDCl_3$)

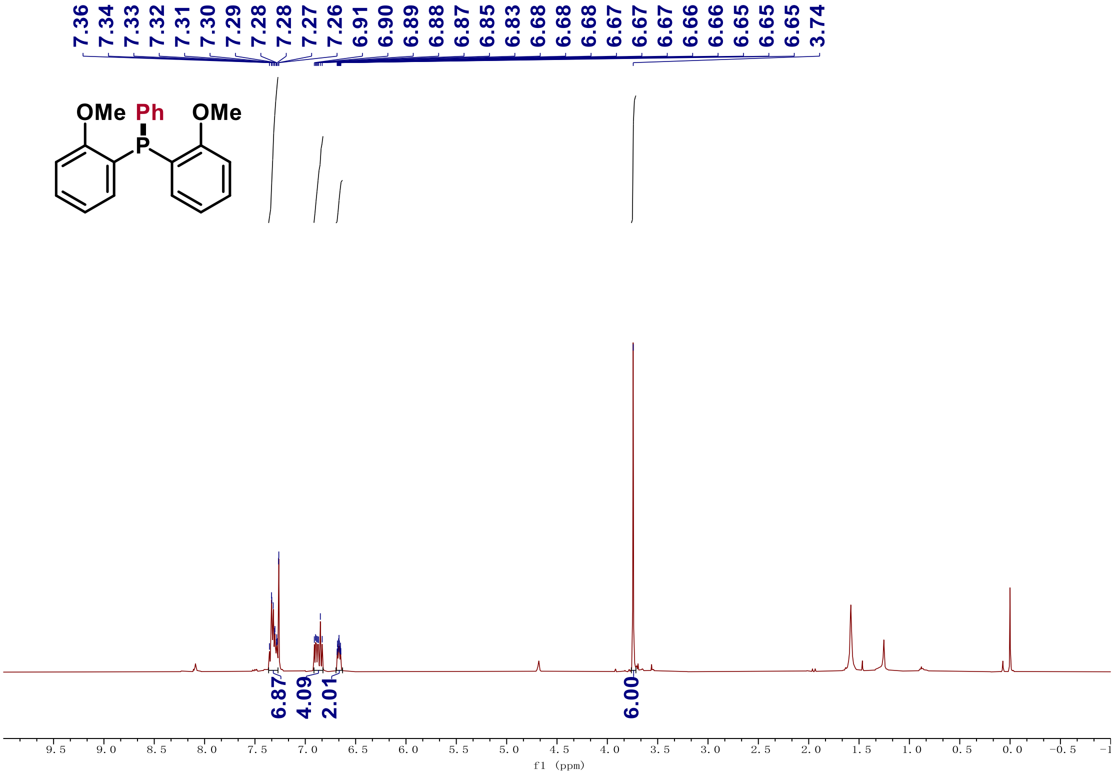

^{13}C NMR of compound **23** (151 MHz in $CDCl_3$)

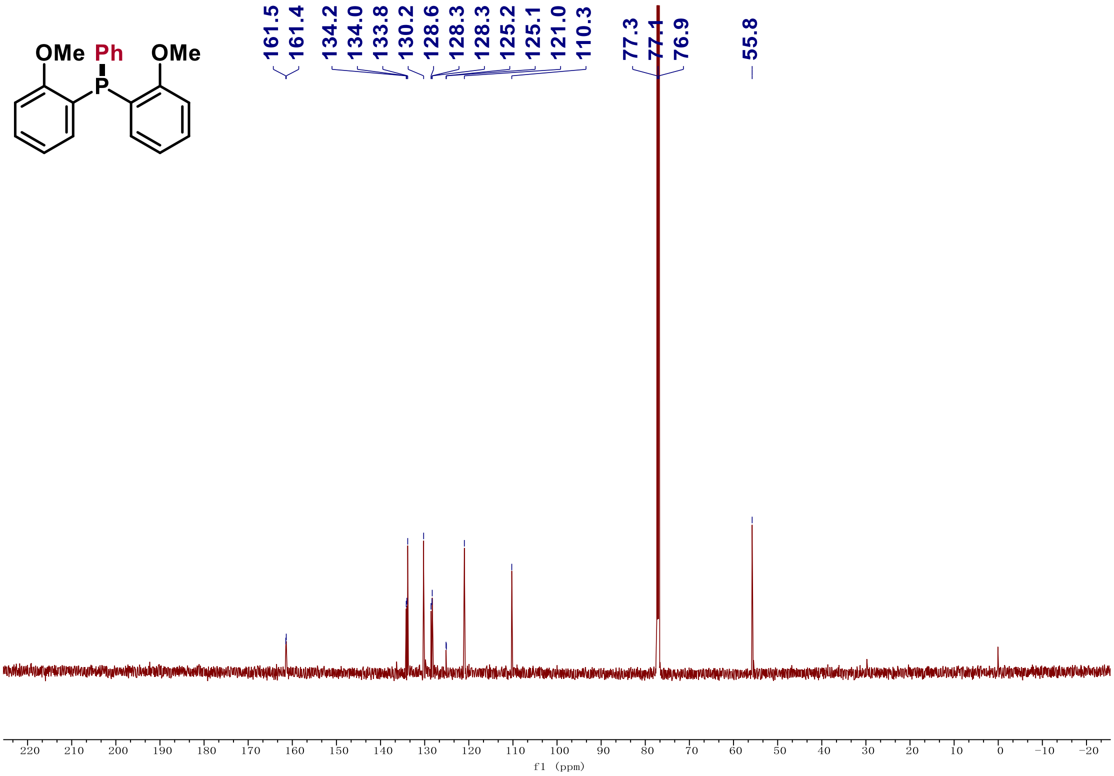

^{31}P NMR of compound **23** (162 MHz in $CDCl_3$)

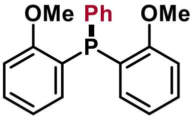

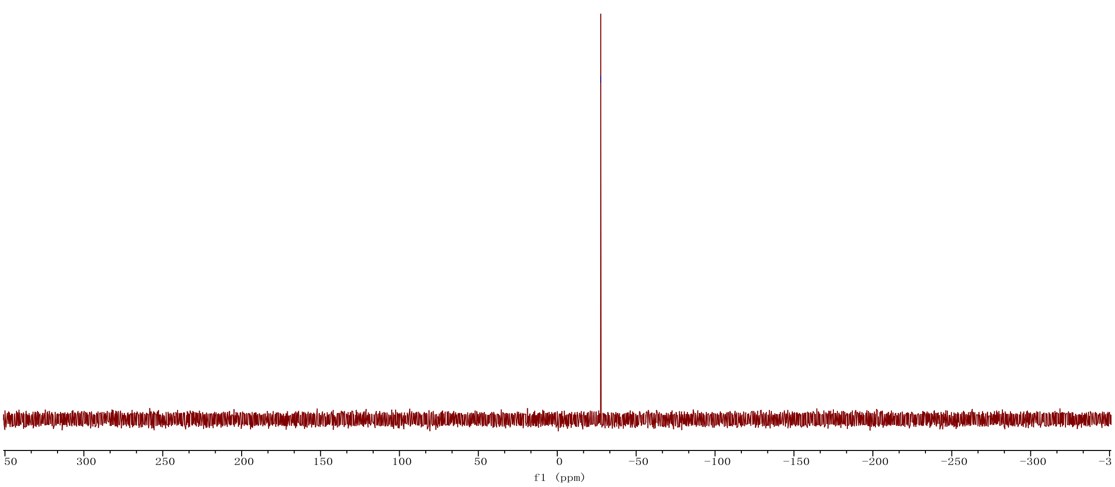

^{1}**H NMR** of compound **24** (500 MHz in $CDCl_3$)

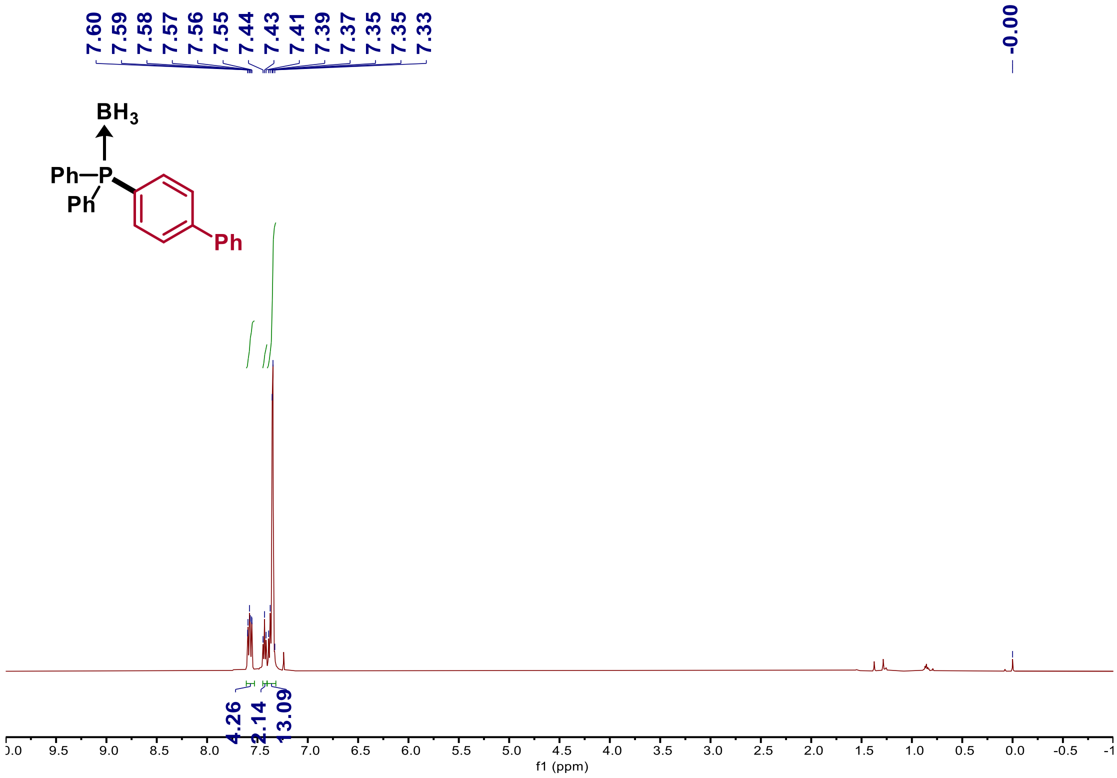

^{13}**C NMR** of compound **24** (126 MHz in $CDCl_3$)

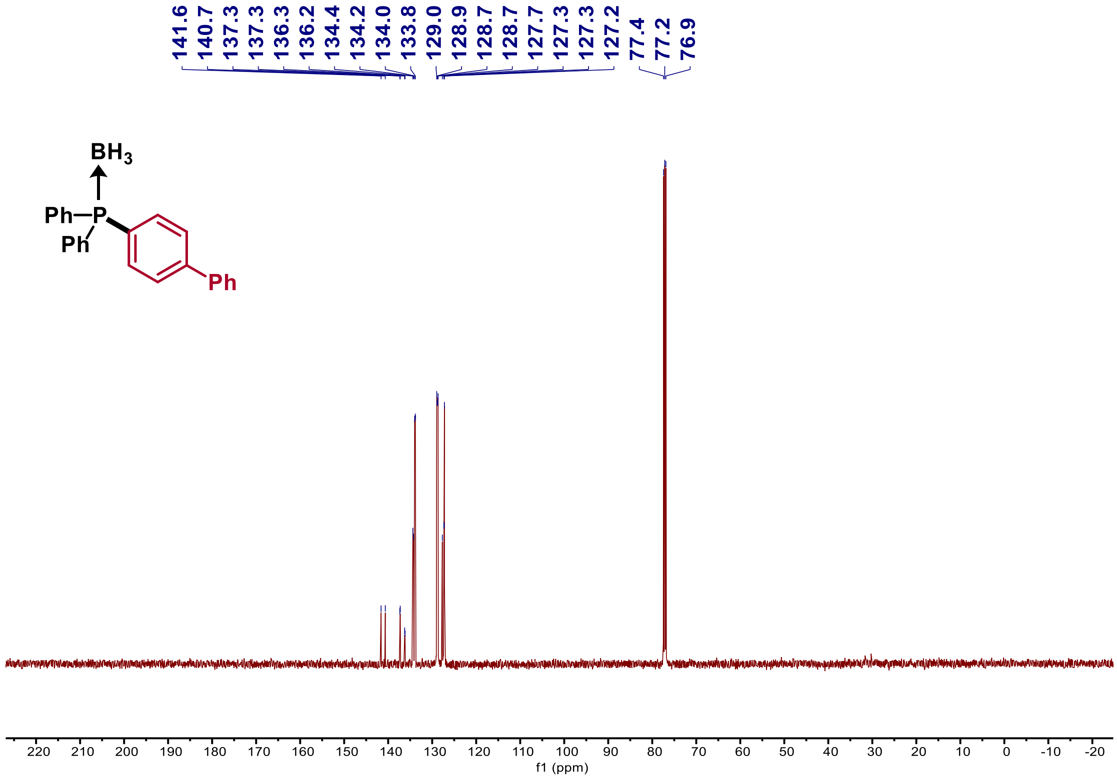

^{11}**B NMR** of compound **24** (160 MHz in $CDCl_3$)

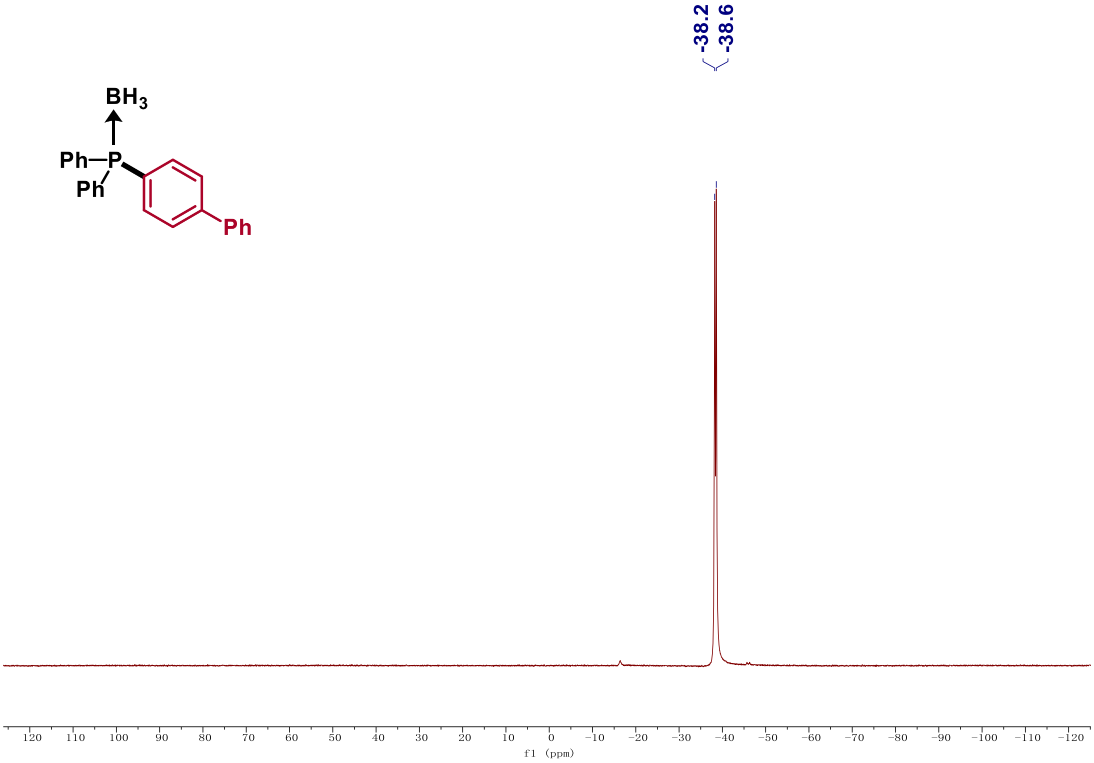

^{31}**P NMR** of compound **24** (202 MHz in $CDCl_3$)

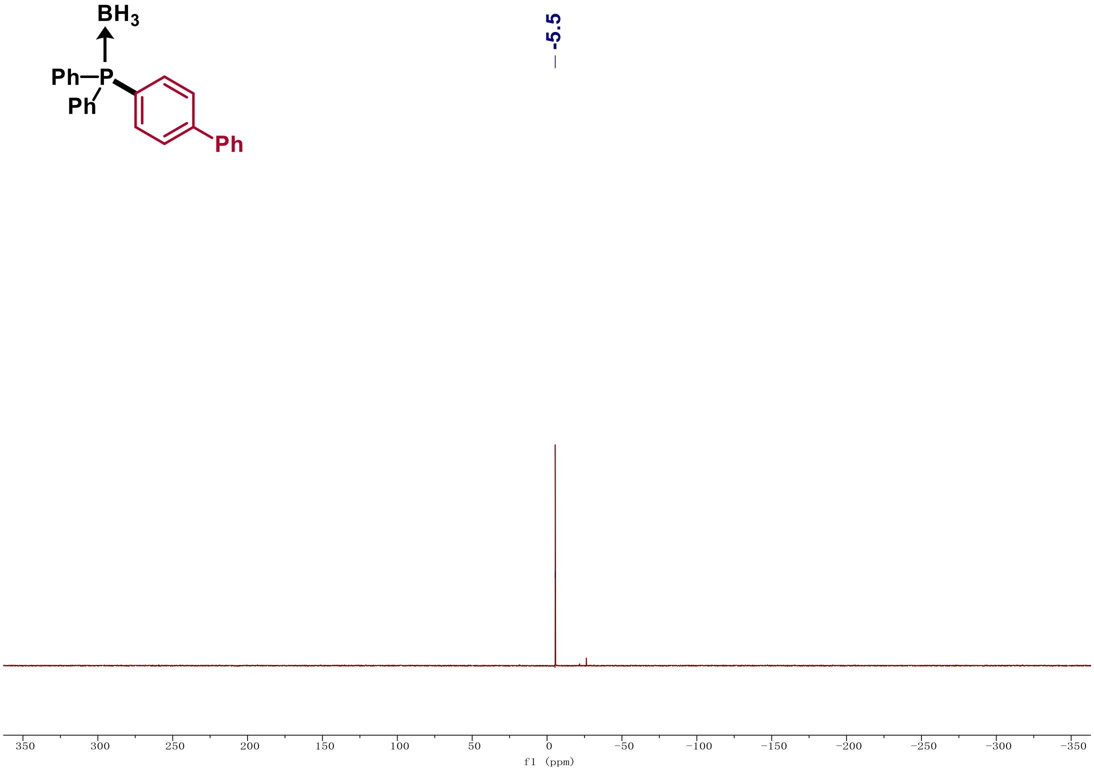

^{1}H NMR of compound **25** (500 MHz in $CDCl_3$)

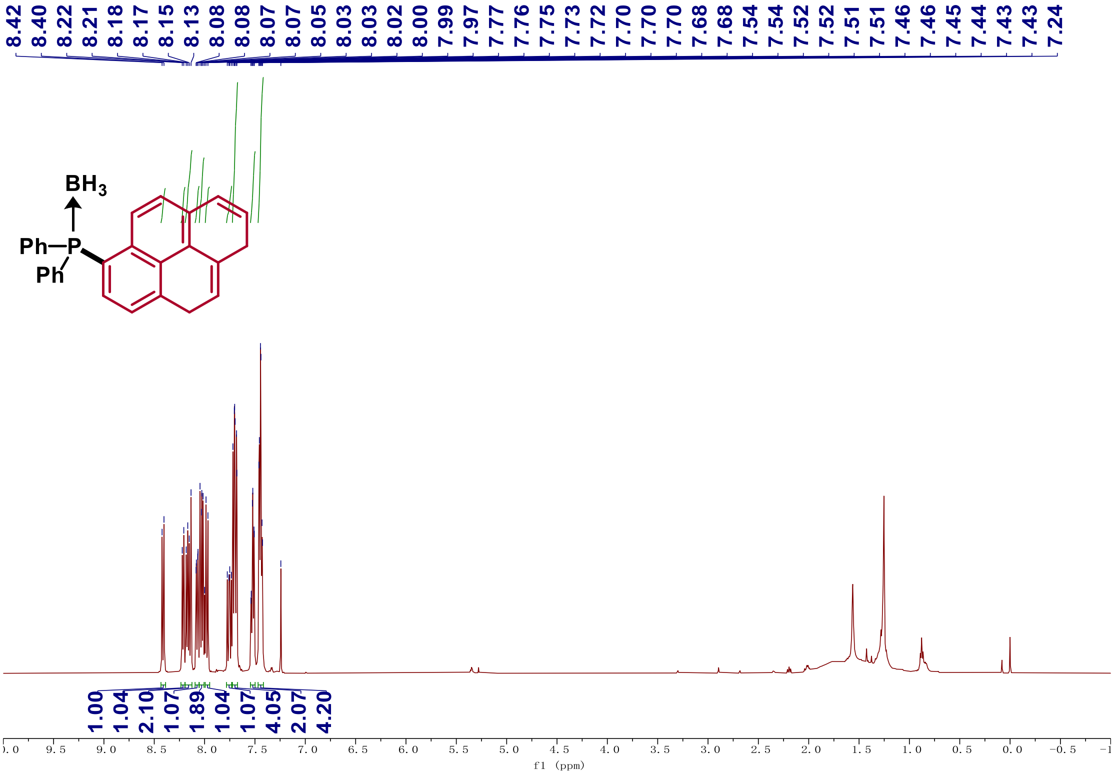

^{13}C NMR of compound **25** (126 MHz in $CDCl_3$)

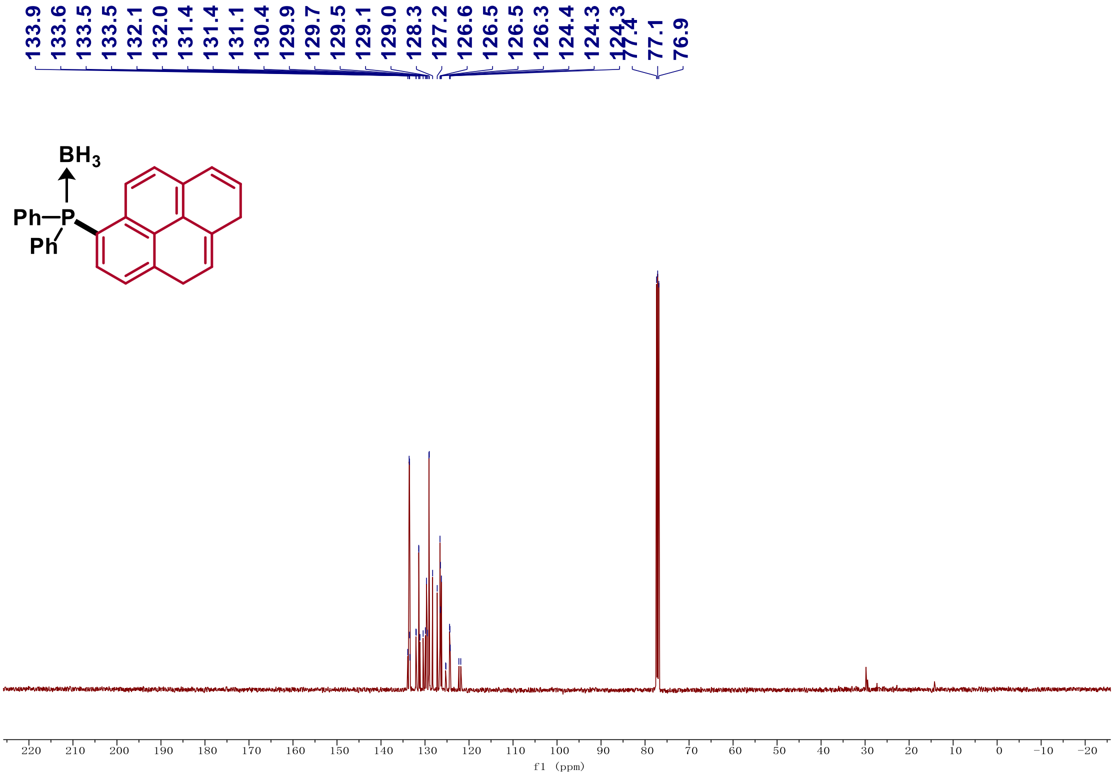

^{11}B NMR of compound **25** (160 MHz in $CDCl_3$)

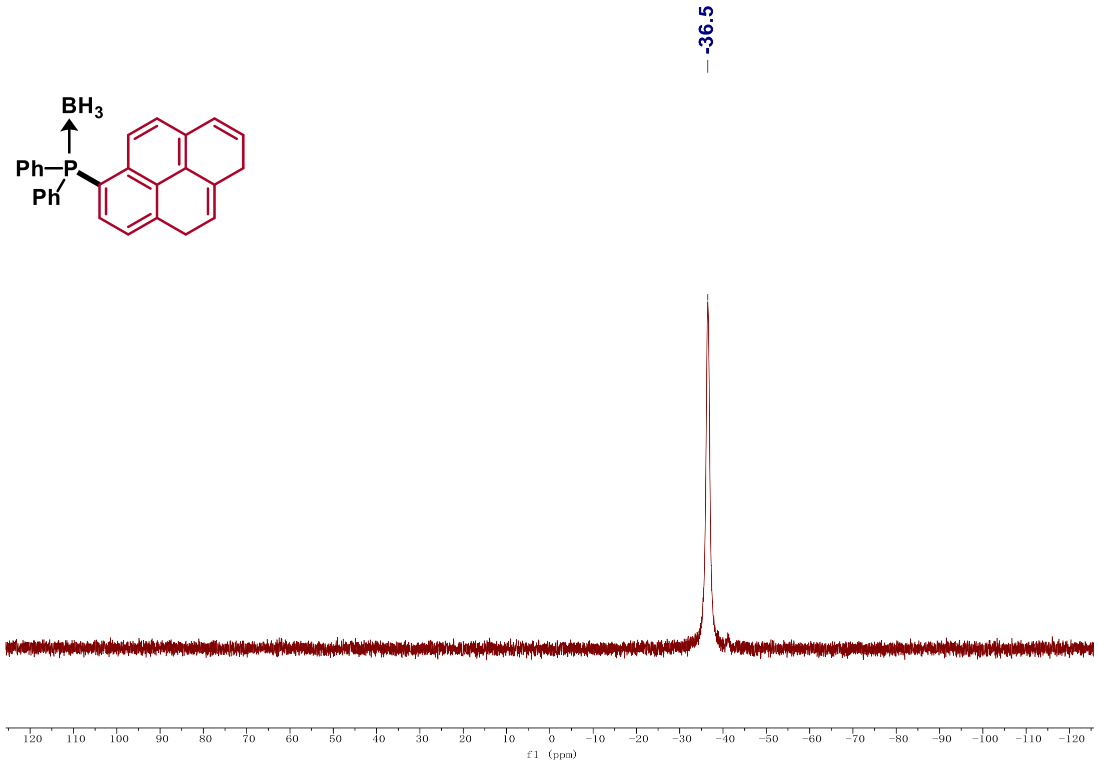

^{31}P NMR of compound **25** (202 MHz in $CDCl_3$)

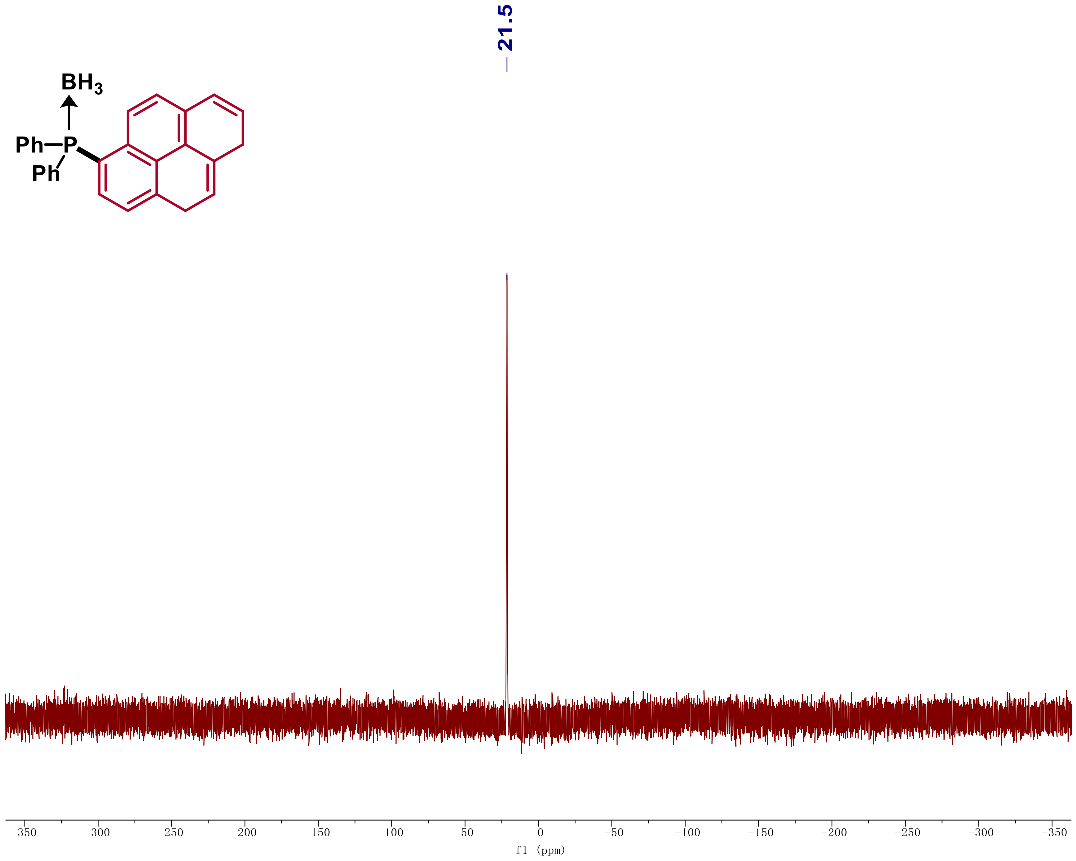

^{1}**H NMR** of compound NHC **B** (500 MHz in DMSO-d_6)

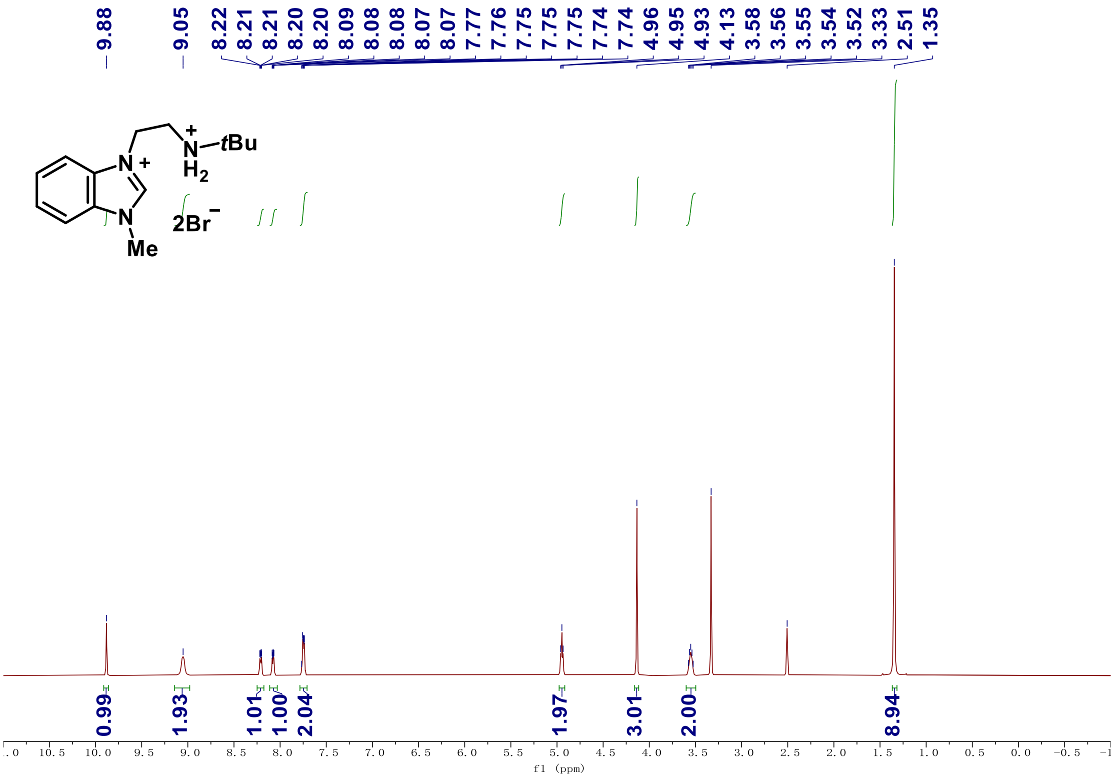

^{13}**C NMR** of compound NHC **B** (126 MHz in DMSO-d_6)

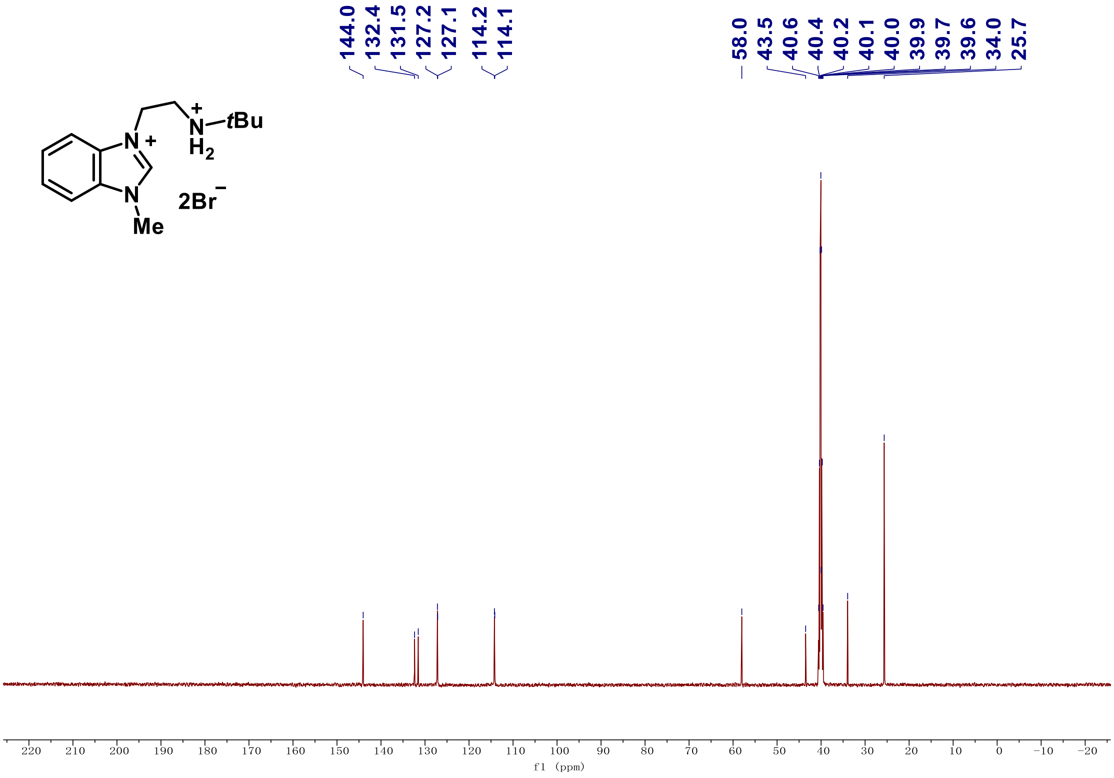

^{1}H NMR of compound NHC **C** (400 MHz in DMSO-d_6)

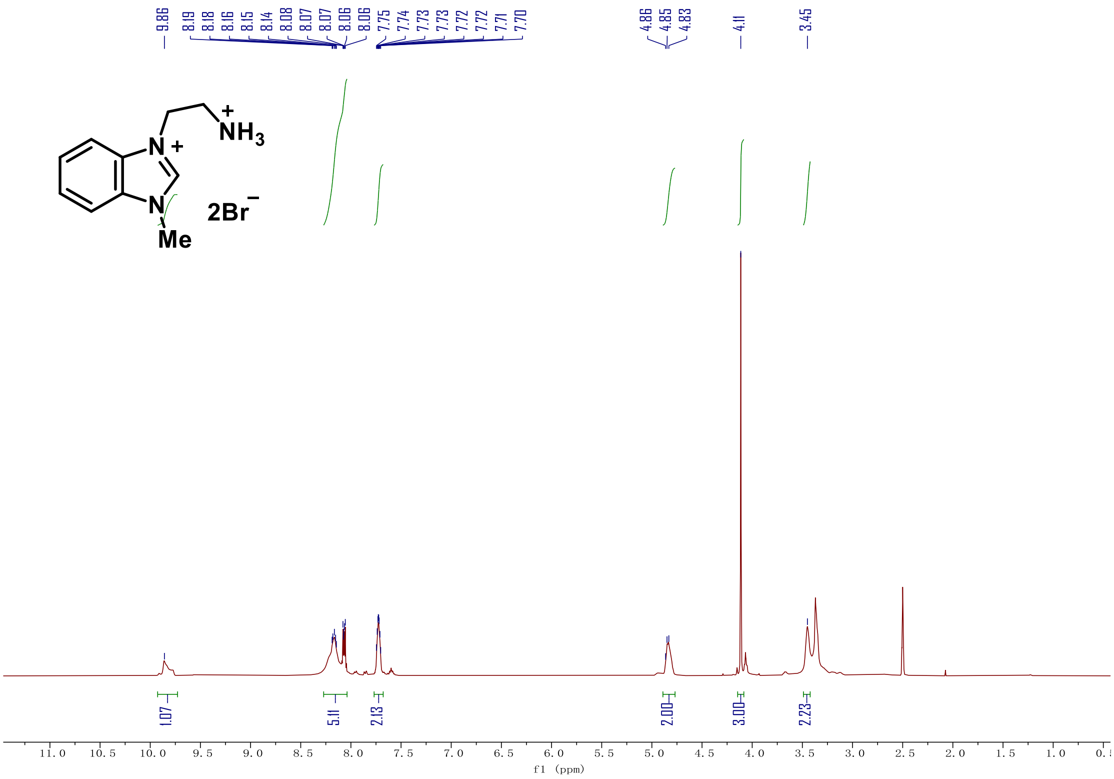

^{13}C NMR of compound NHC **C** (101 MHz in DMSO-d_6)

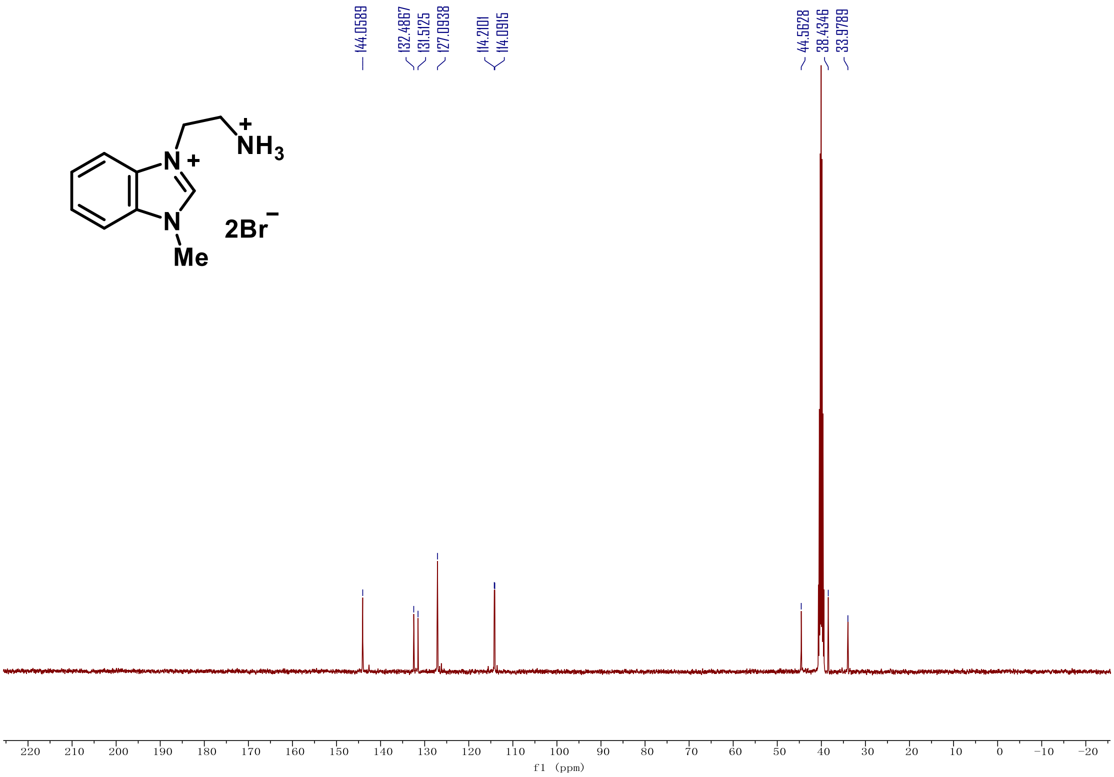

^{1}H NMR of compound NHC **D** (500 MHz in $CDCl_3$)

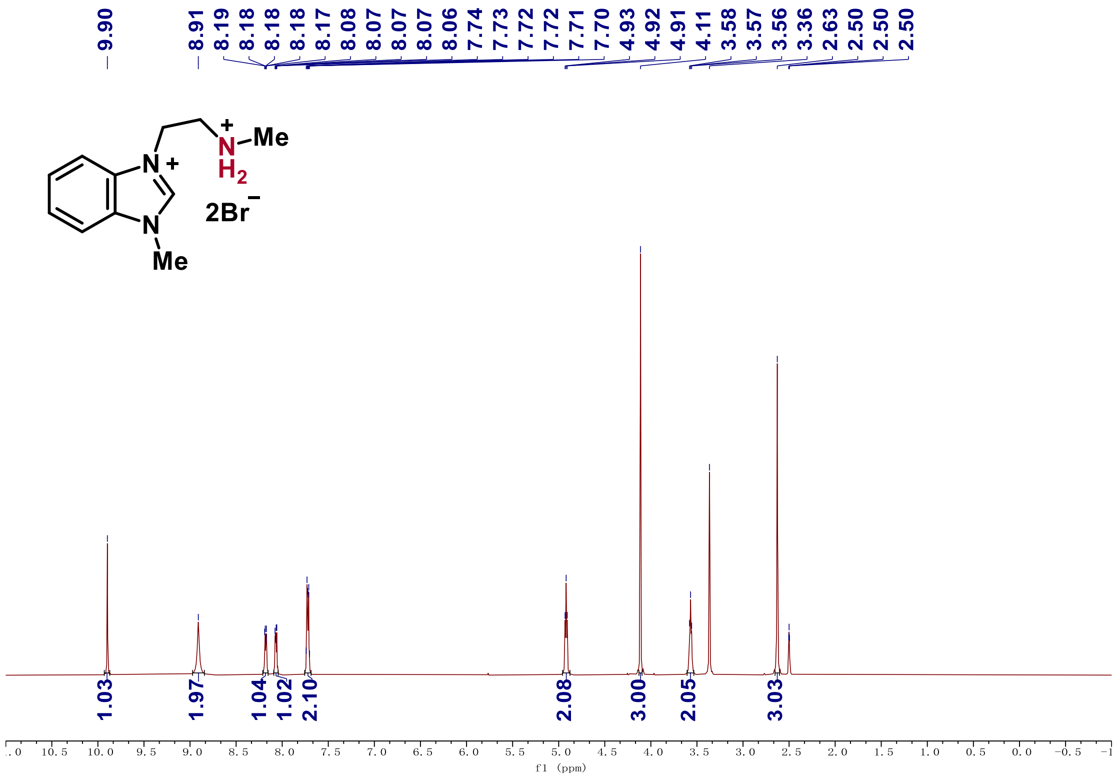

^{13}C NMR of compound NHC **D** (101 MHz in $CDCl_3$)

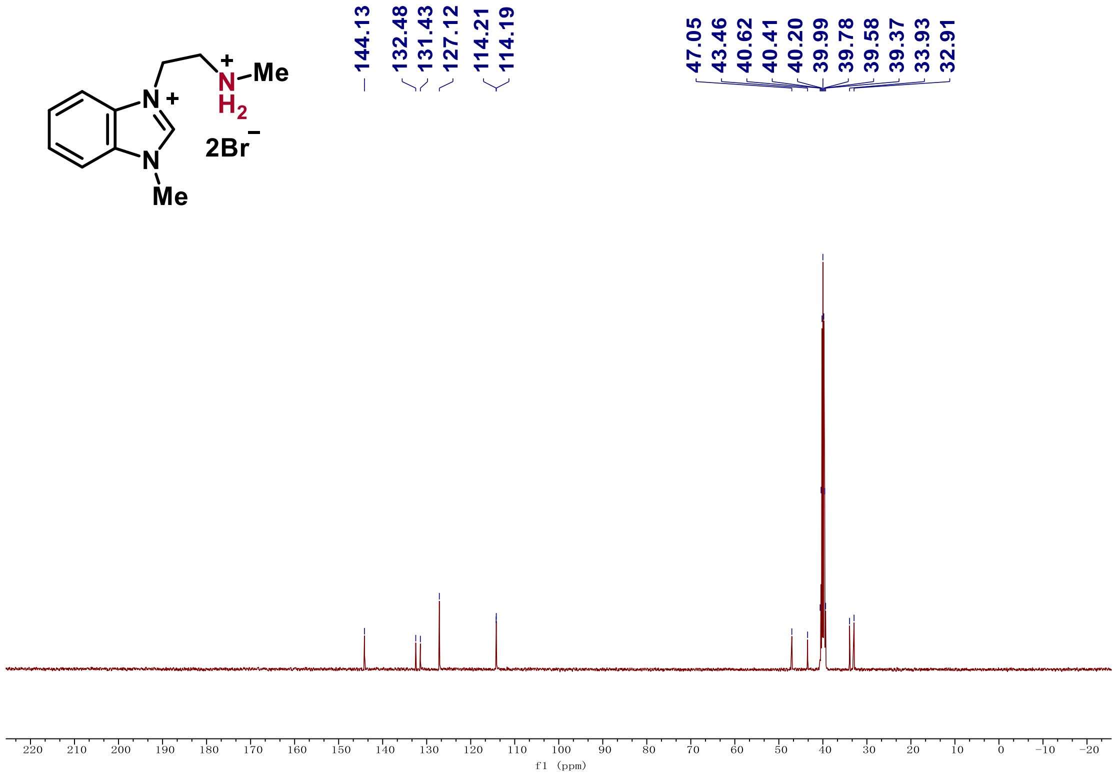

^{1}H NMR of compound NHC **E** (500 MHz in DMSO-d_6)

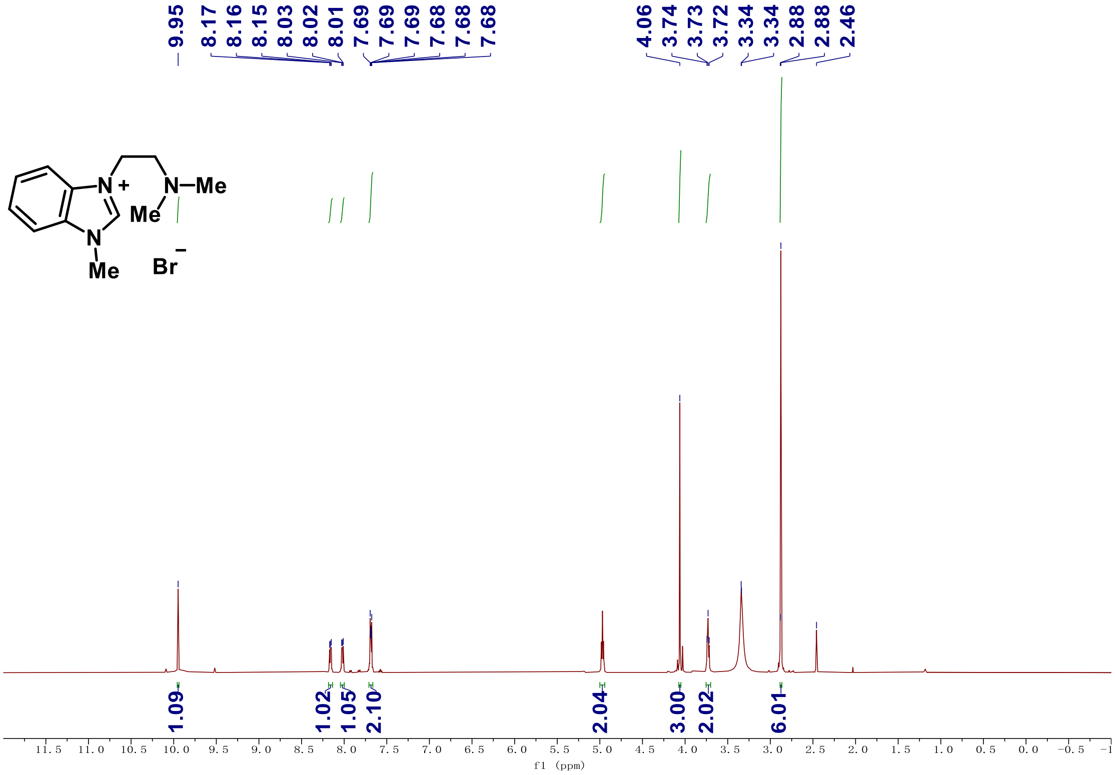

^{13}C NMR of compound NHC **E** (126 MHz in DMSO-d_6)

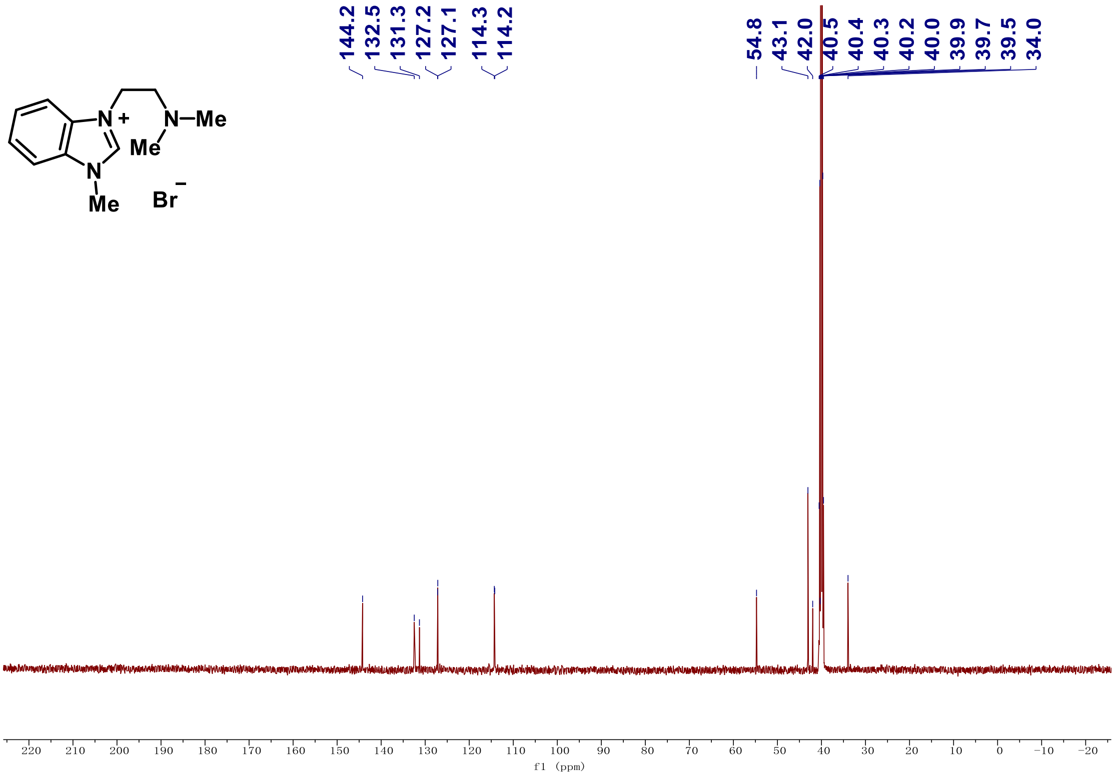

^{1}H NMR of compound NHC **F** (500 MHz in DMSO-d_6)

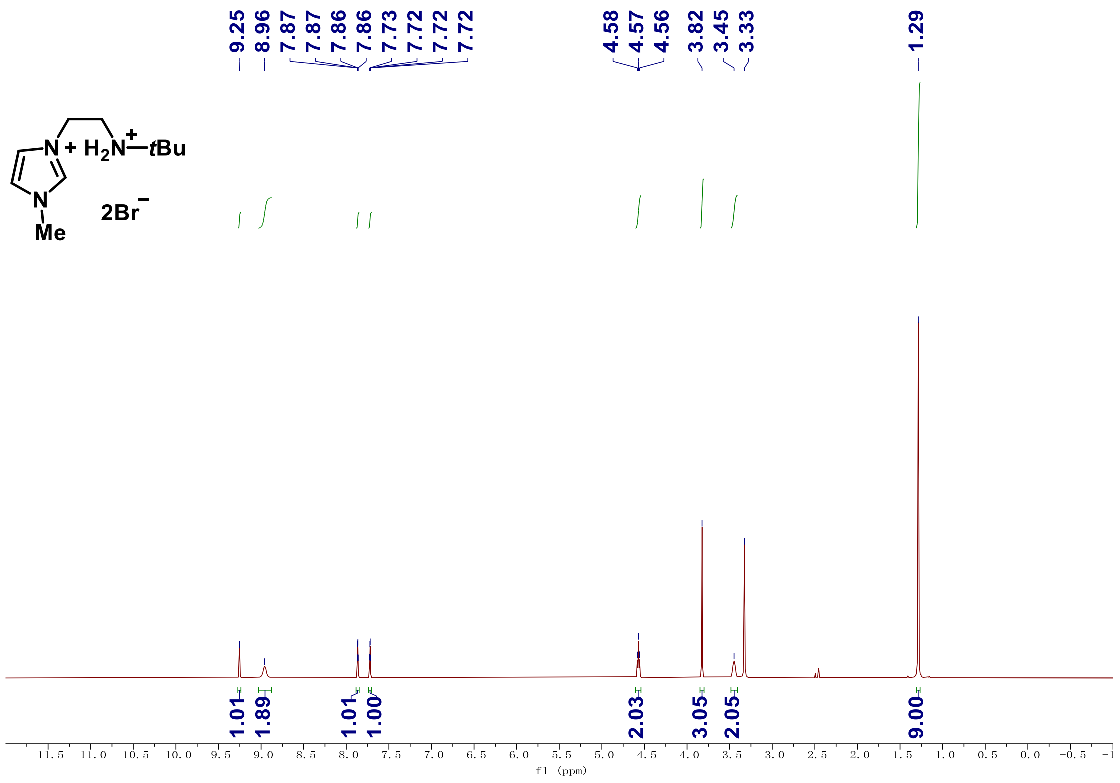

^{13}C NMR of compound NHC **F** (126 MHz in DMSO-d_6)

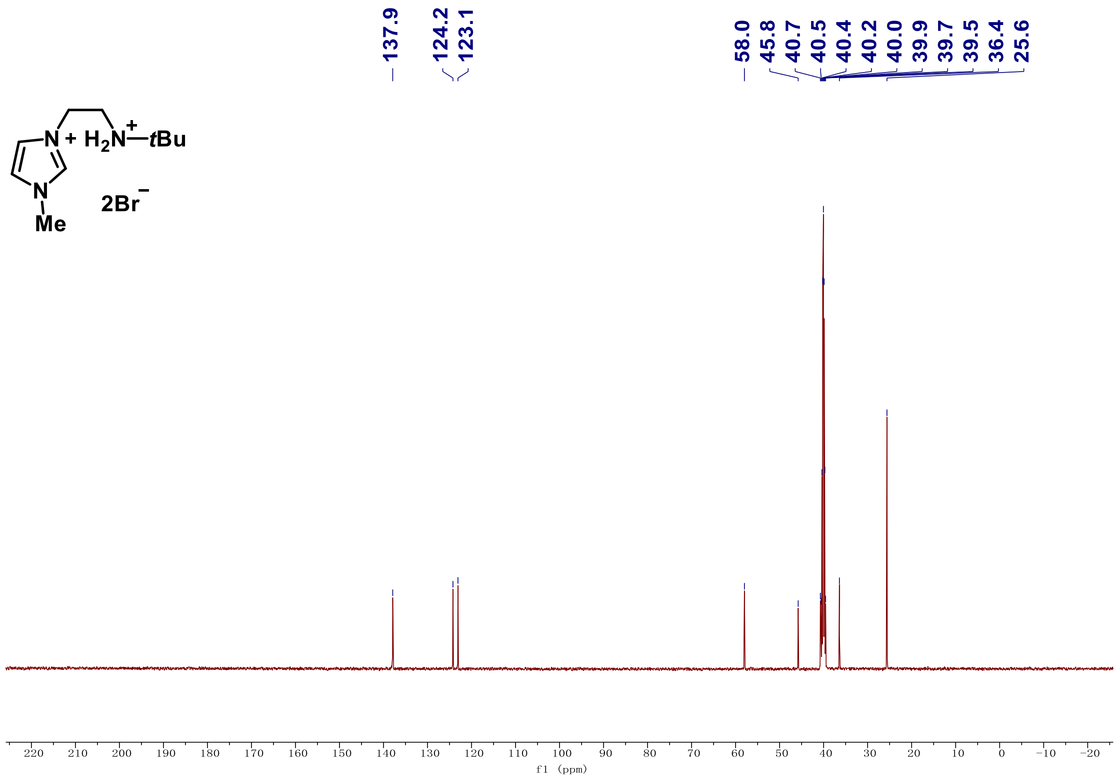

^{1}H NMR of compound NHC **G**(500 MHz in DMSO-*d*$_6$)

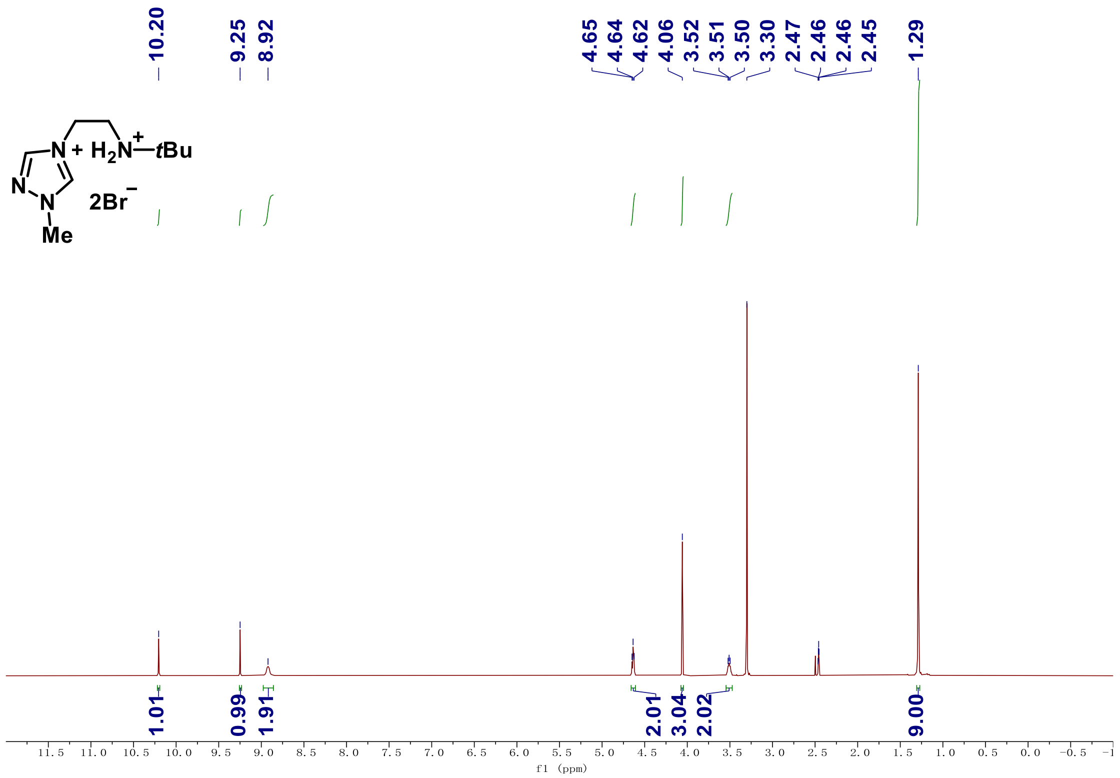

^{13}C NMR of compound NHC **G** (126 MHz in DMSO-*d*$_6$)

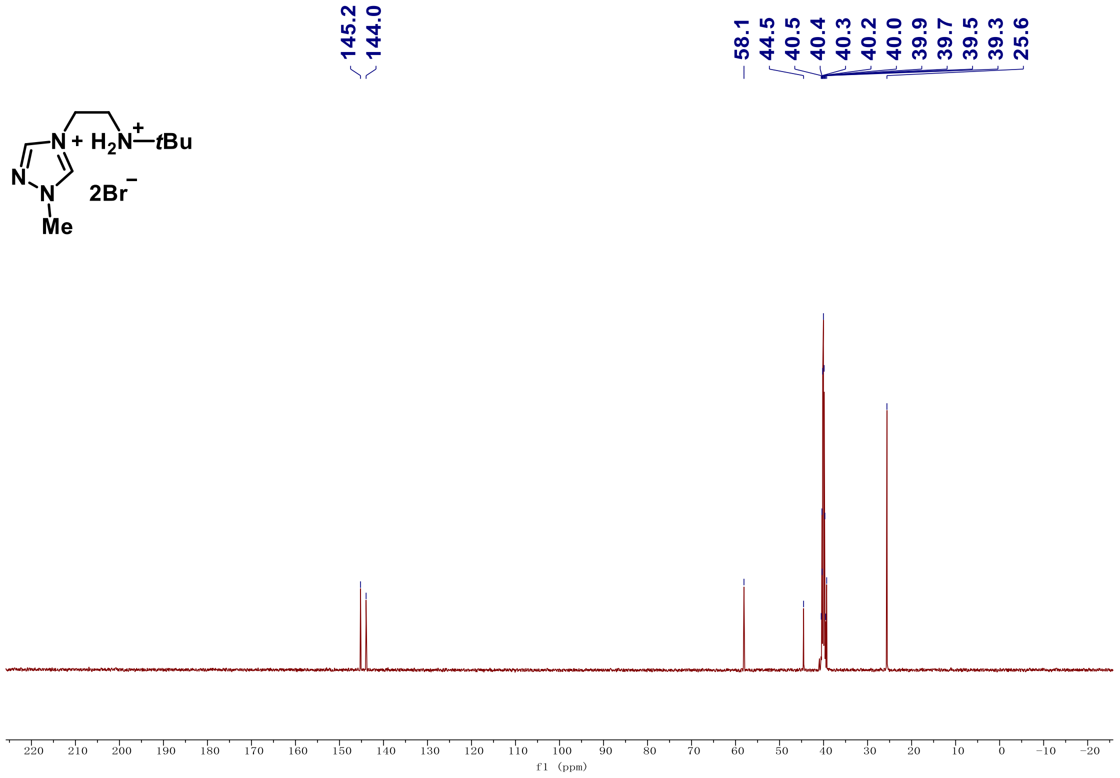

^{1}**H NMR** of compound **28** (500 MHz in $CDCl_3$)

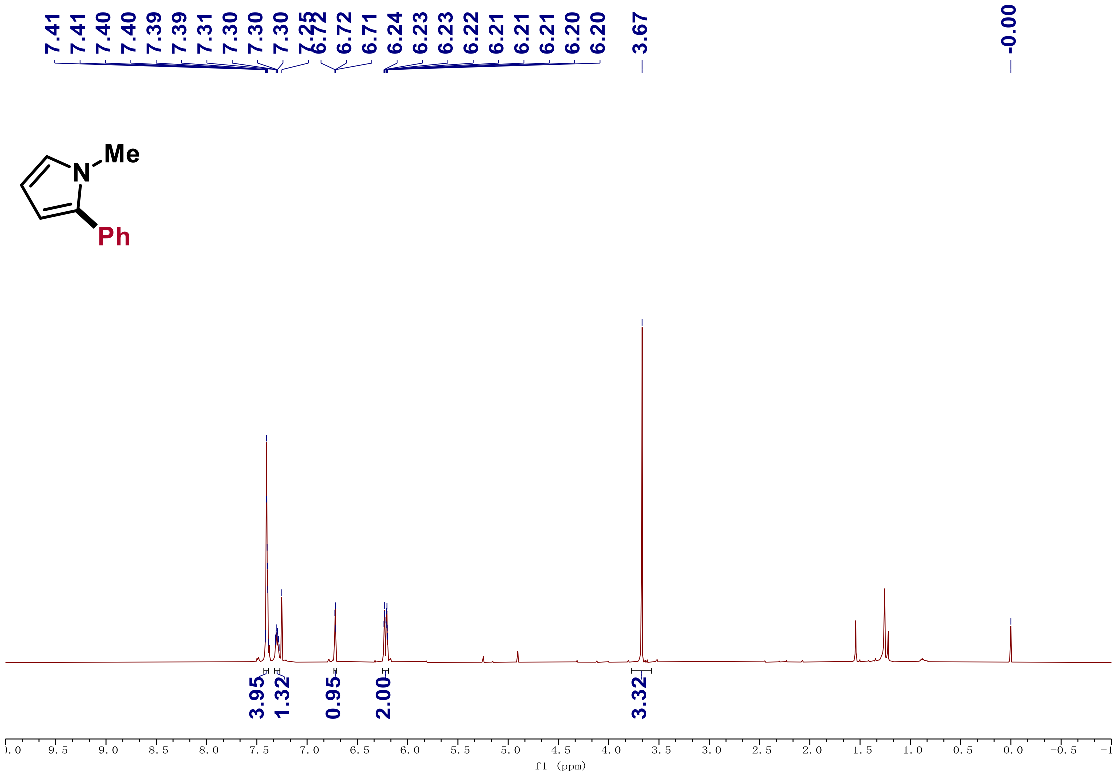

^{13}**C NMR** of compound **28** (126 MHz in $CDCl_3$)

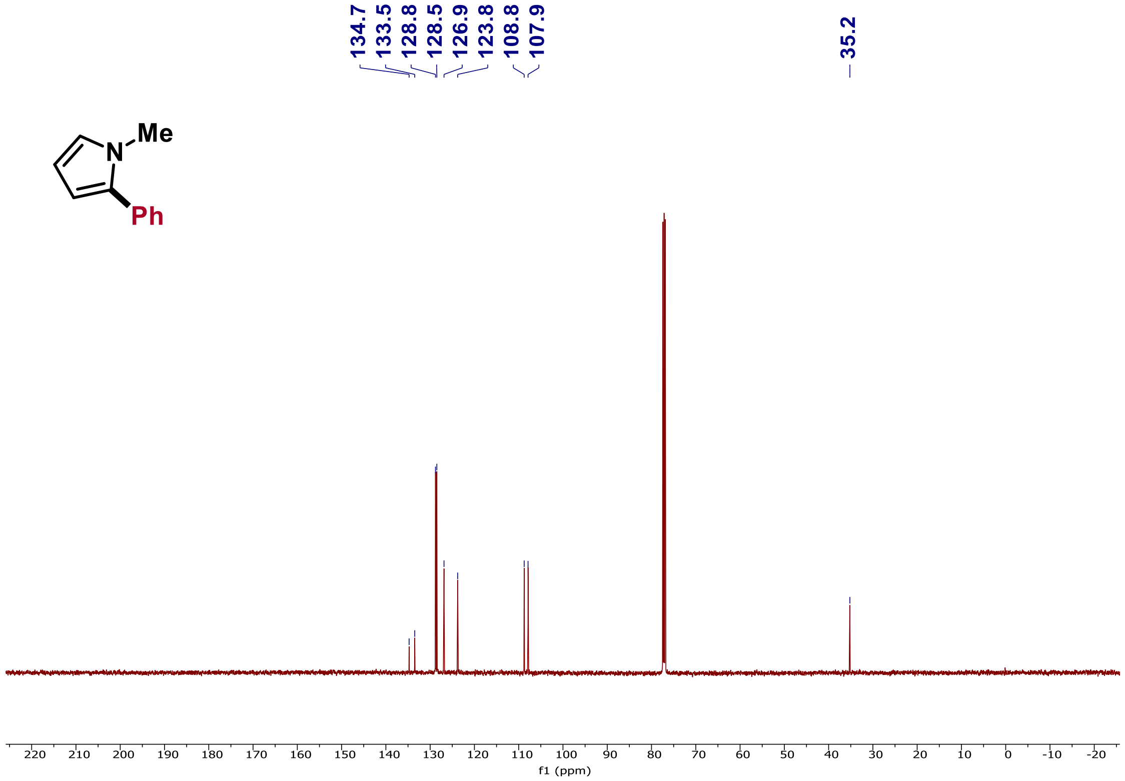

^{1}H NMR of compound **31** (400 MHz in $CDCl_3$)

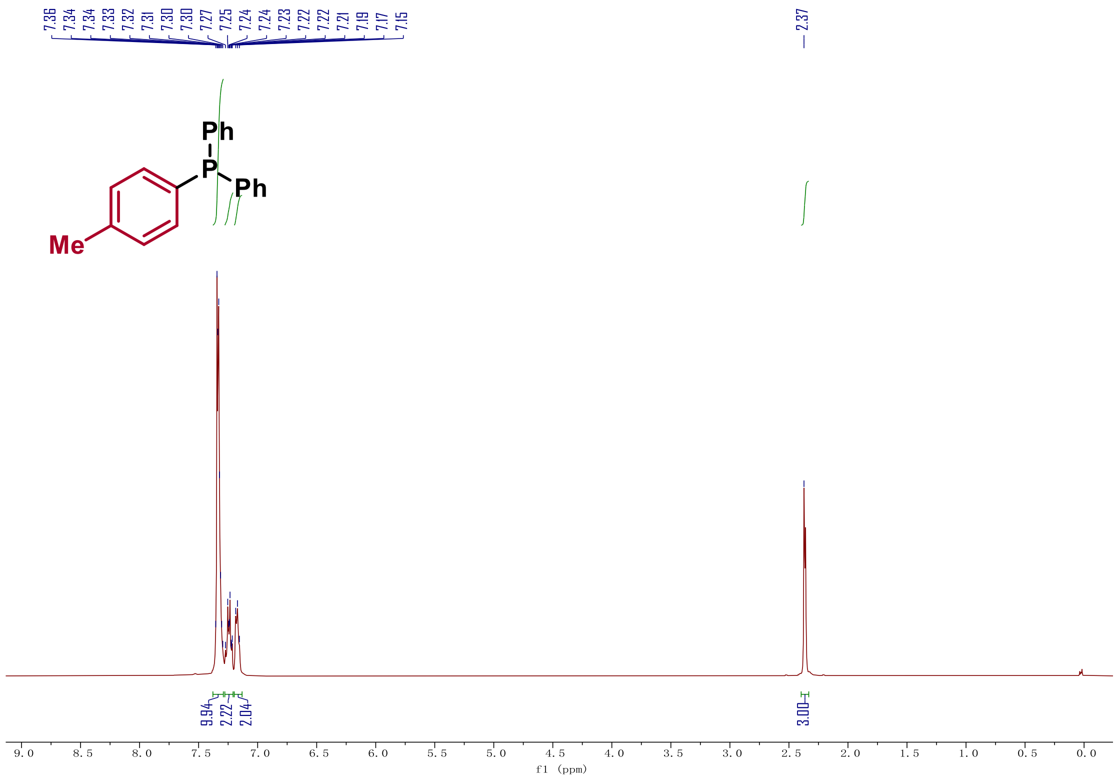

^{13}C NMR of compound **31** (151 MHz in $CDCl_3$)

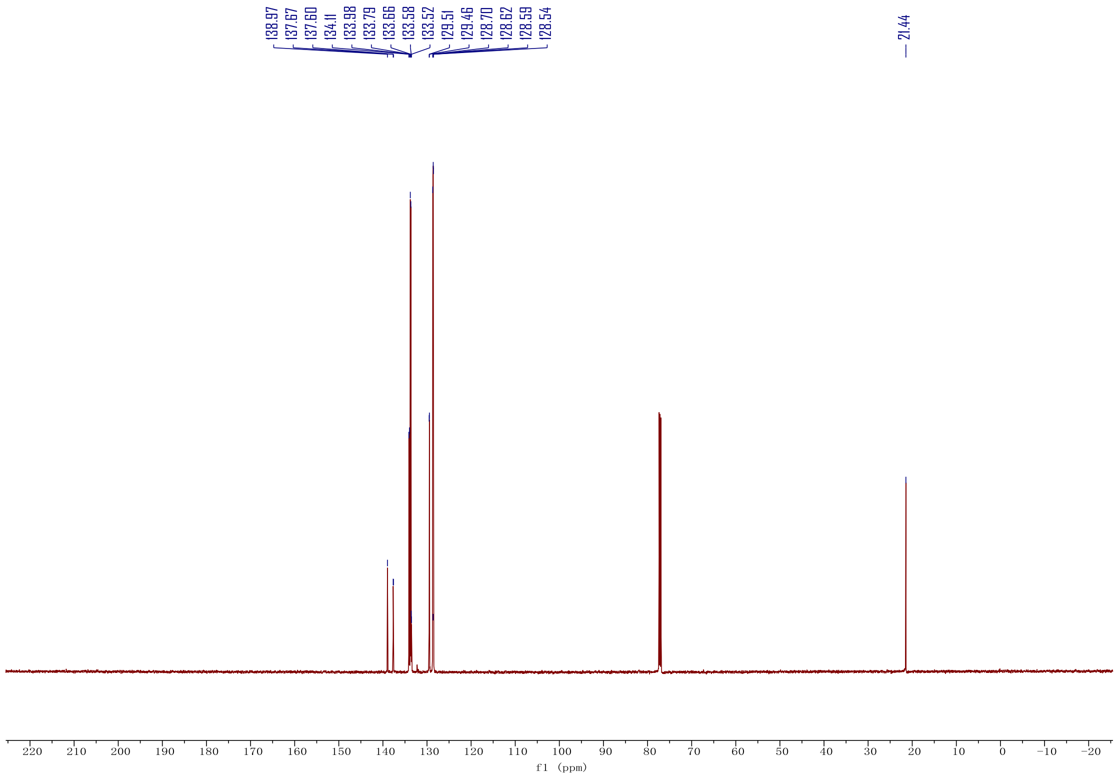

^{31}**P NMR** of compound **31** (162 MHz in $CDCl_3$)

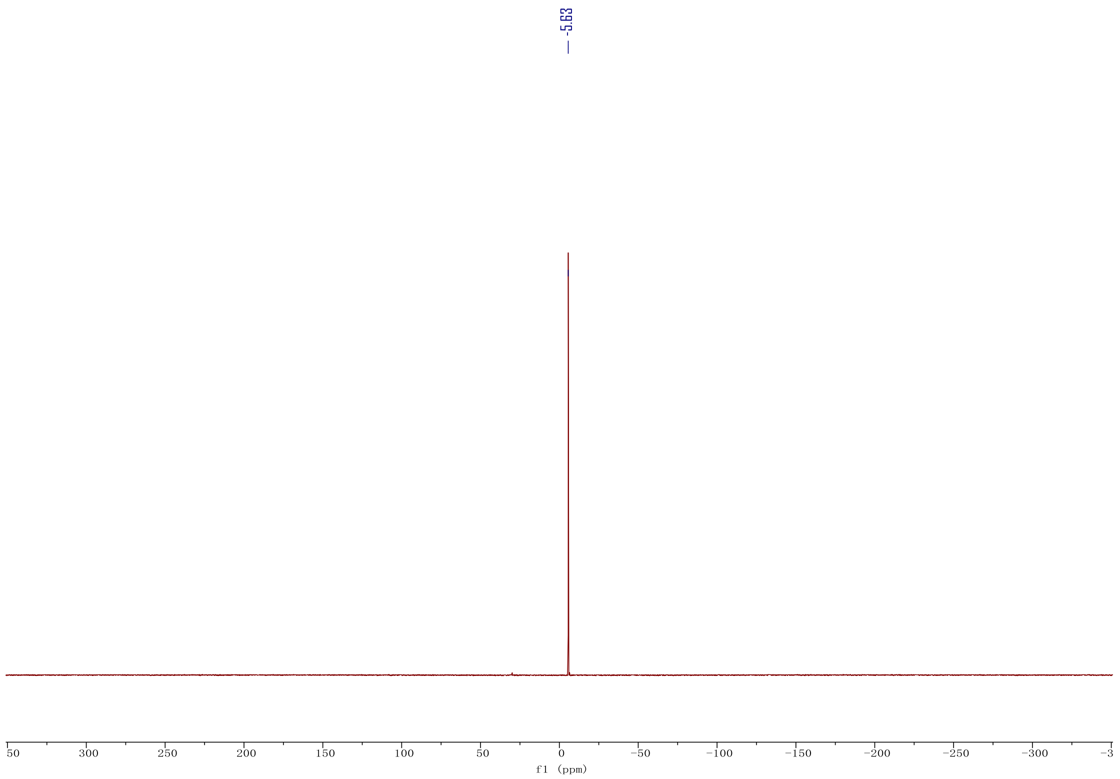

^{1}H NMR of compound **32** (400 MHz in $CDCl_3$)

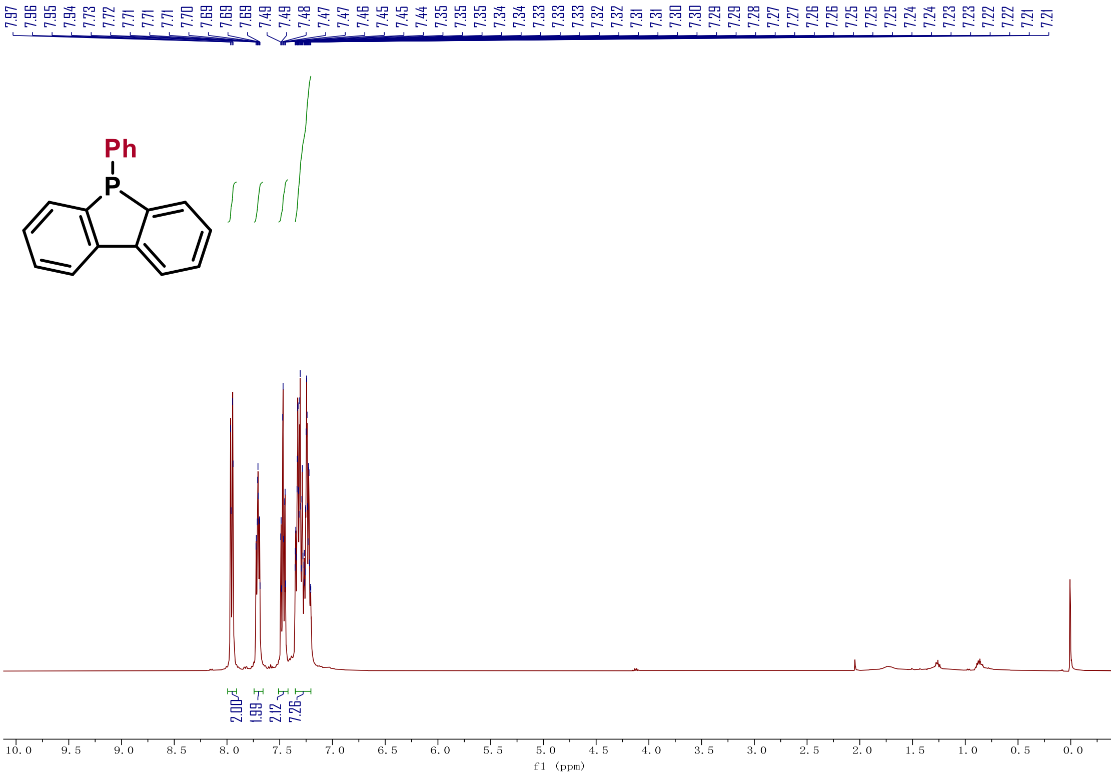

^{13}C NMR of compound **32** (151 MHz in $CDCl_3$)

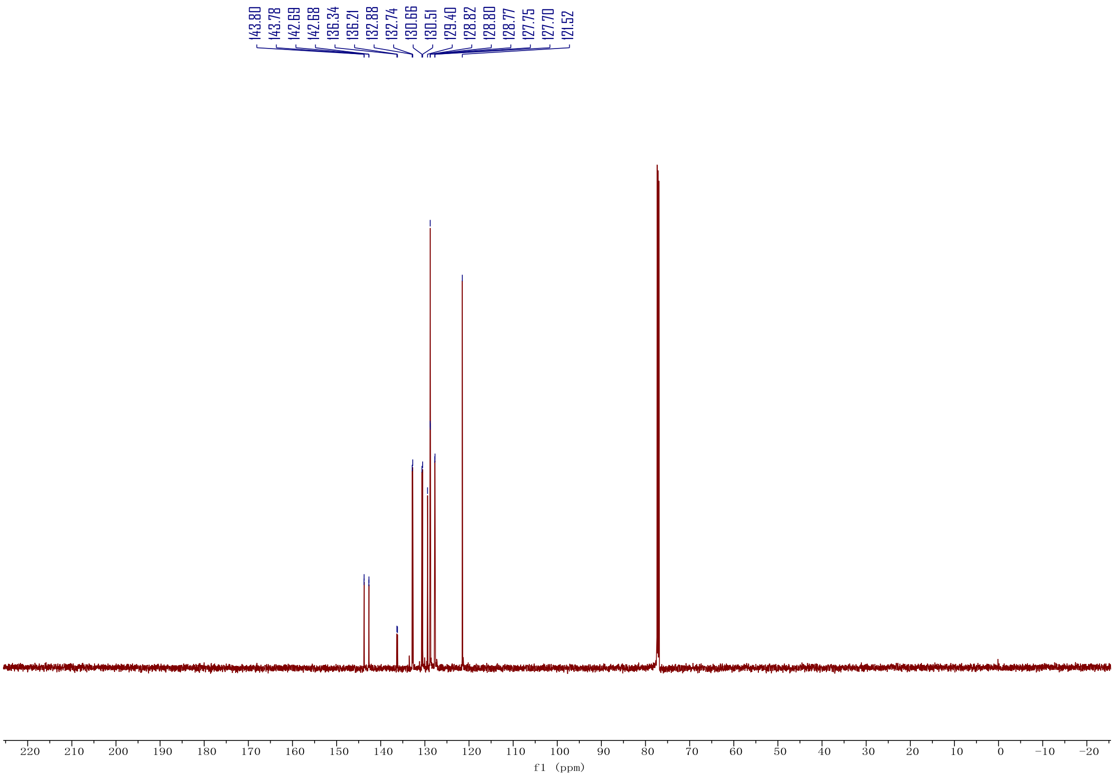

^{31}P NMR of compound **32** (162 MHz in $CDCl_3$)

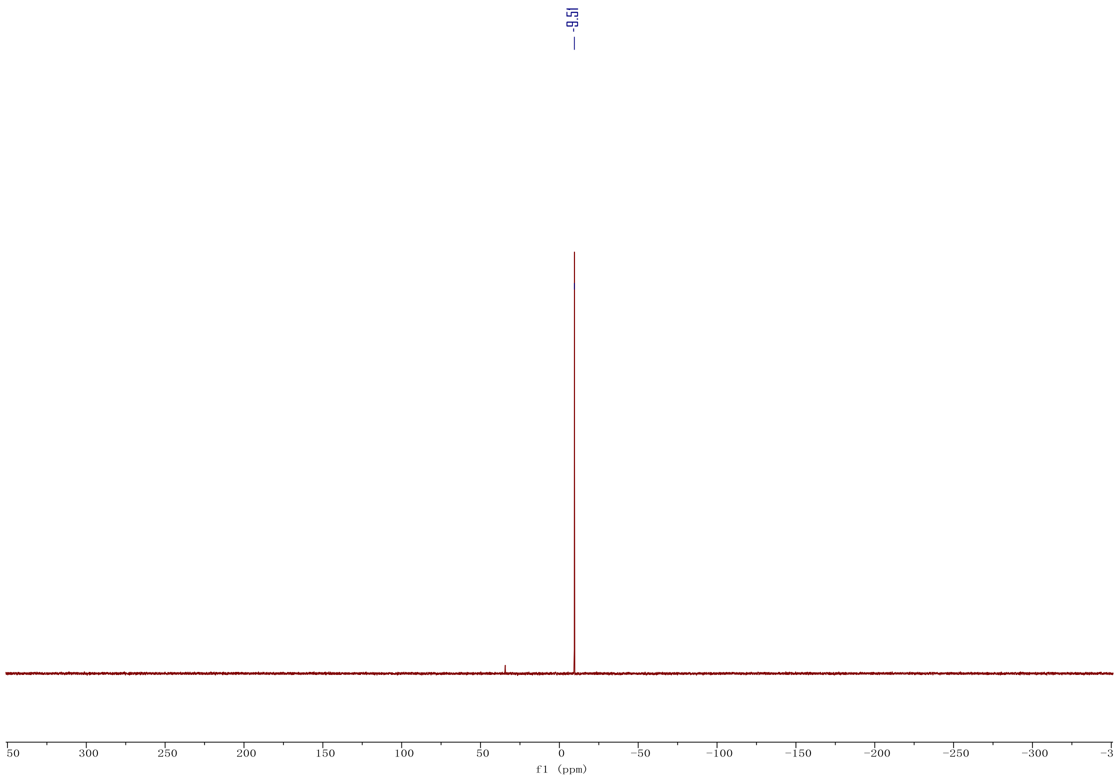

^{1}H NMR of compound **33** (400 MHz in $CDCl_3$)

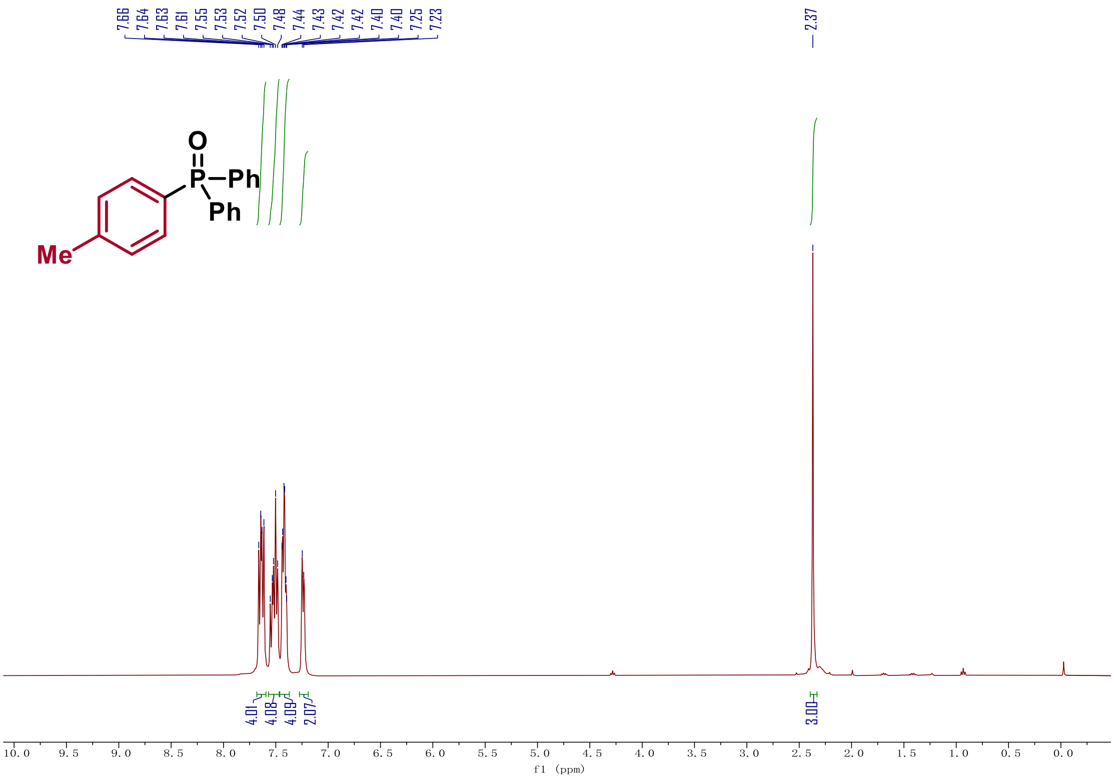

^{13}C NMR of compound **33** (101 MHz in $CDCl_3$)

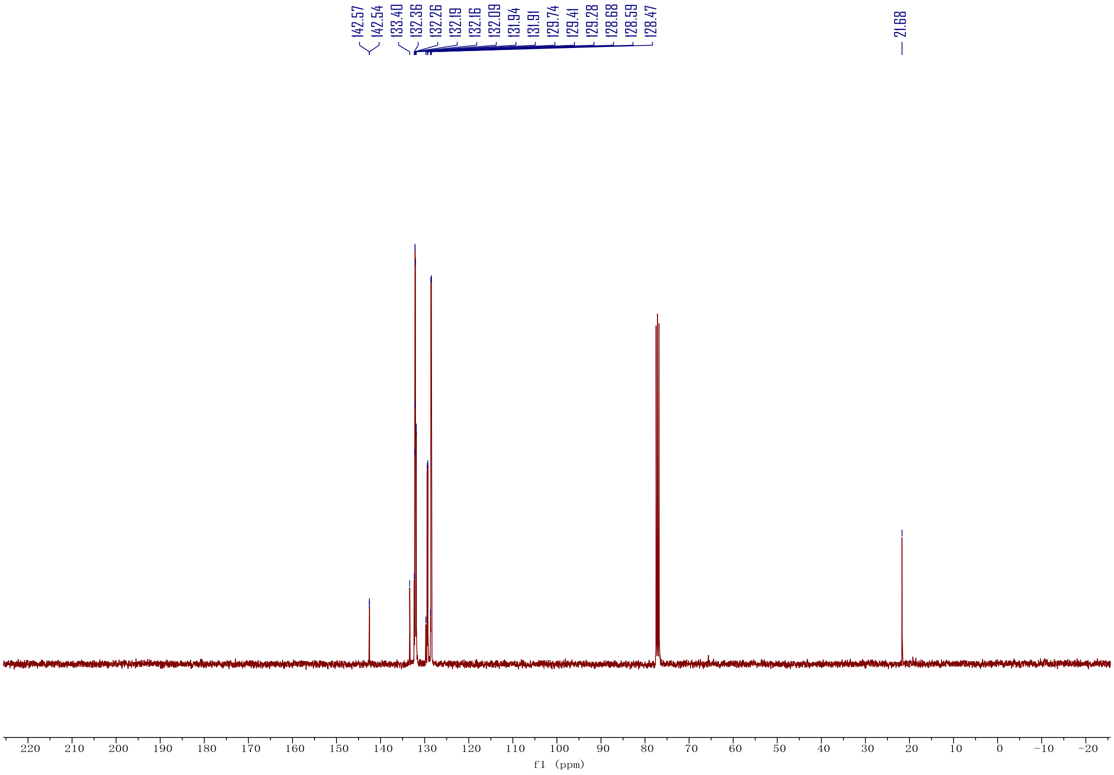

^{31}**P NMR** of compound **33** (162 MHz in $CDCl_3$)

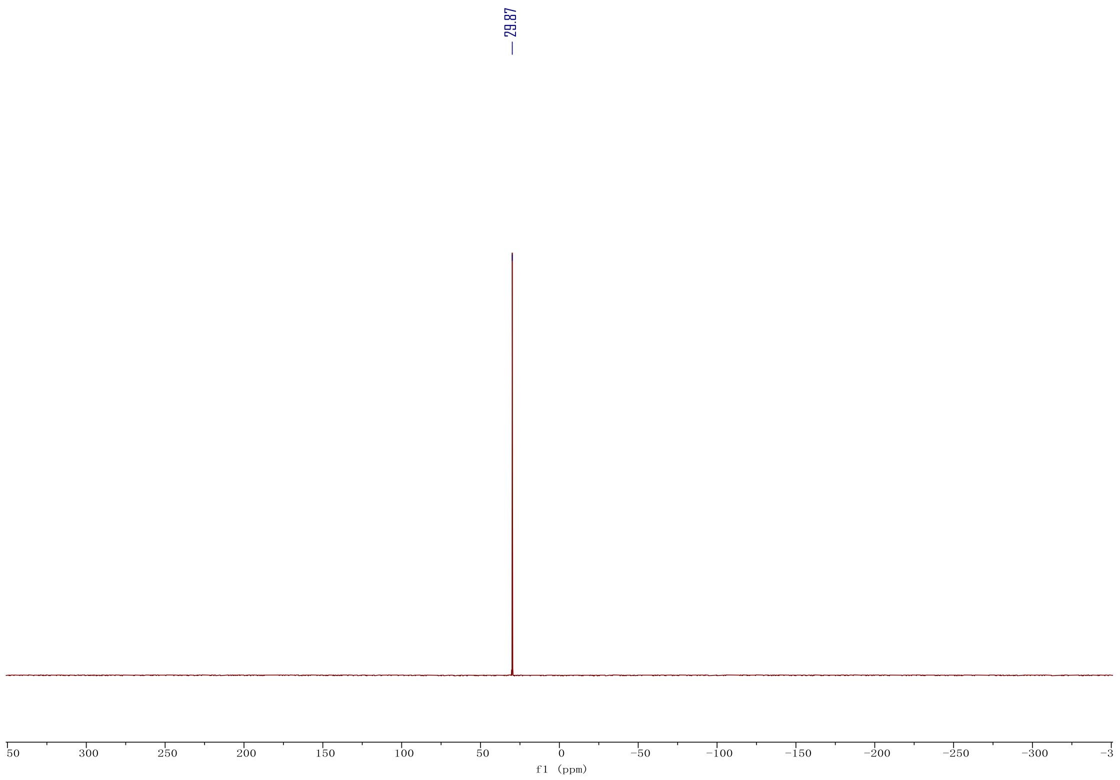

^{1}H NMR of compound **34** (600 MHz in $CDCl_3$)

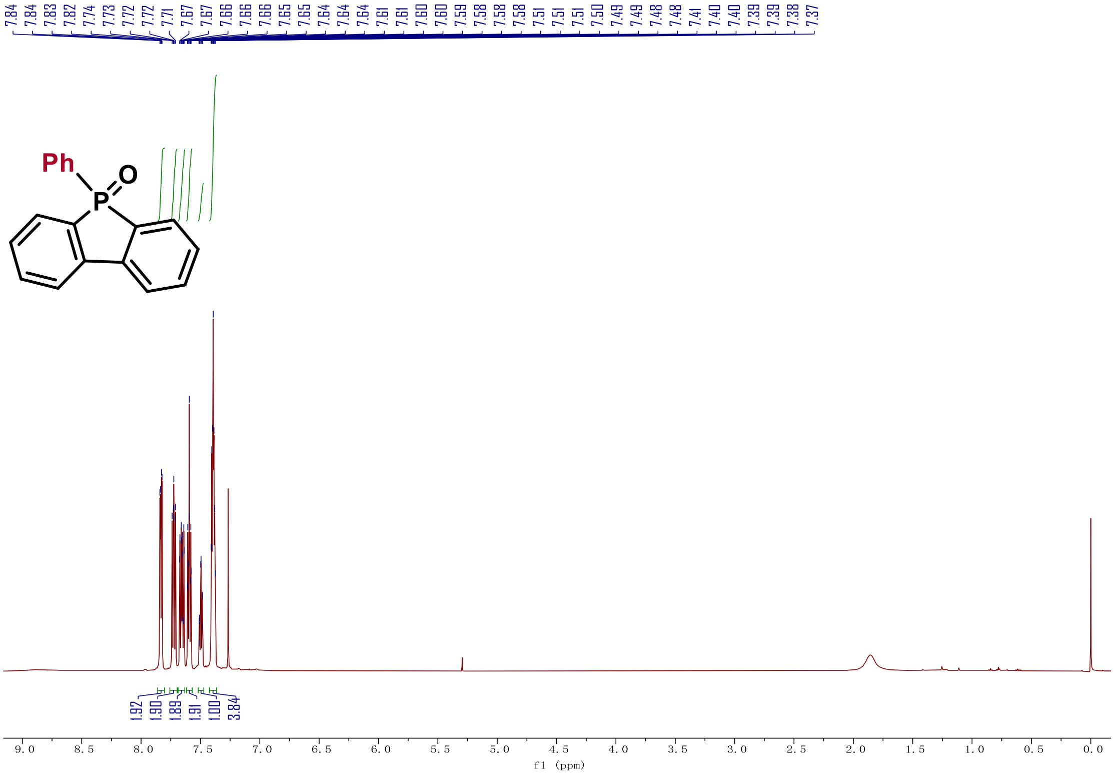

^{13}C NMR of compound **34** (151 MHz in $CDCl_3$)

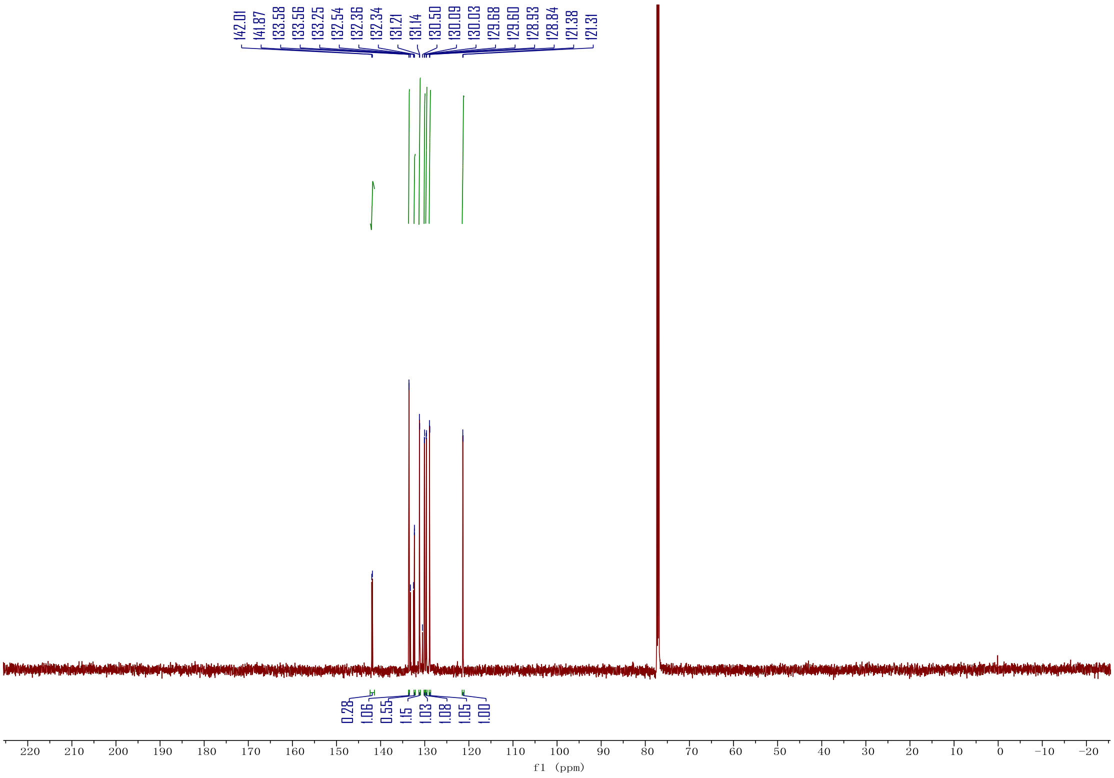

^{31}P NMR of compound **34** (243 MHz in $CDCl_3$)

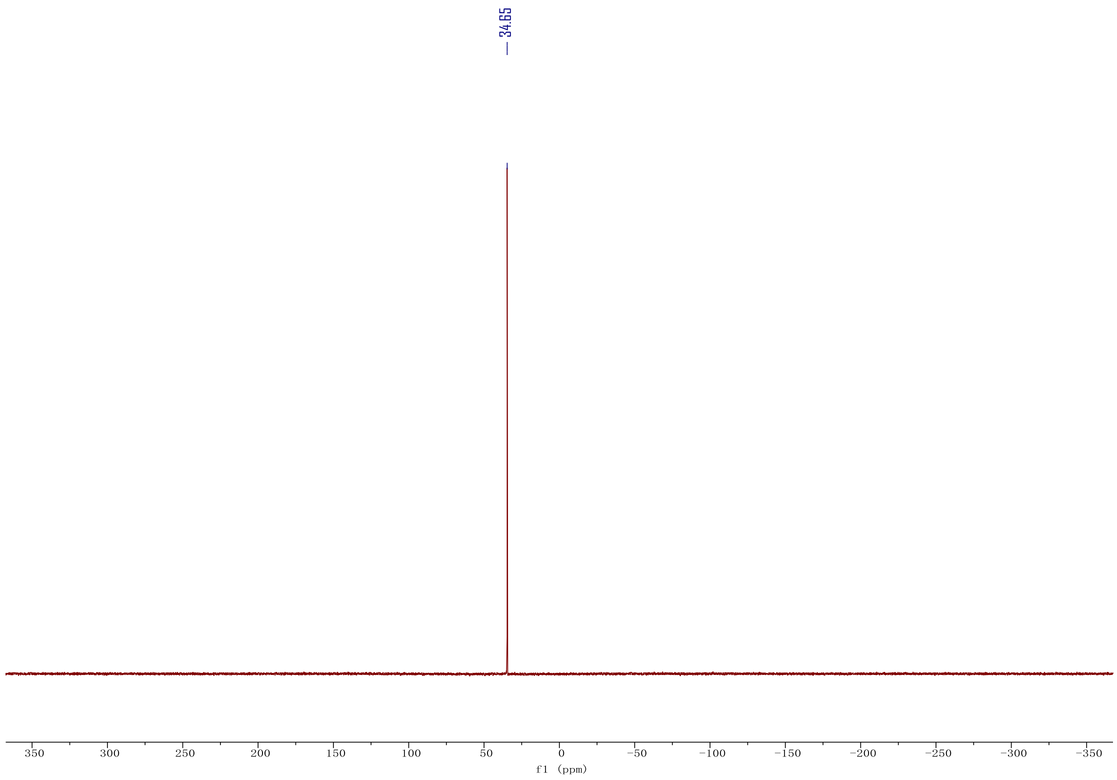

^{1}H NMR of compound **35** (400 MHz in $CDCl_3$)

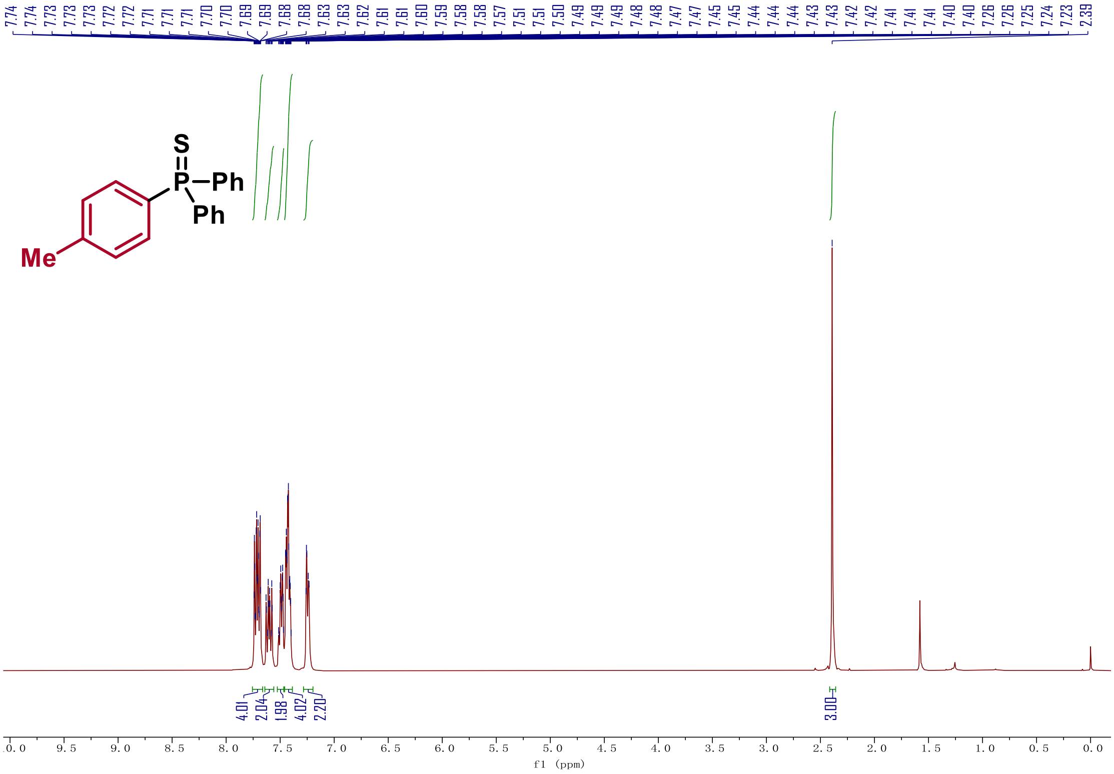

^{13}C NMR of compound **35** (101 MHz in $CDCl_3$)

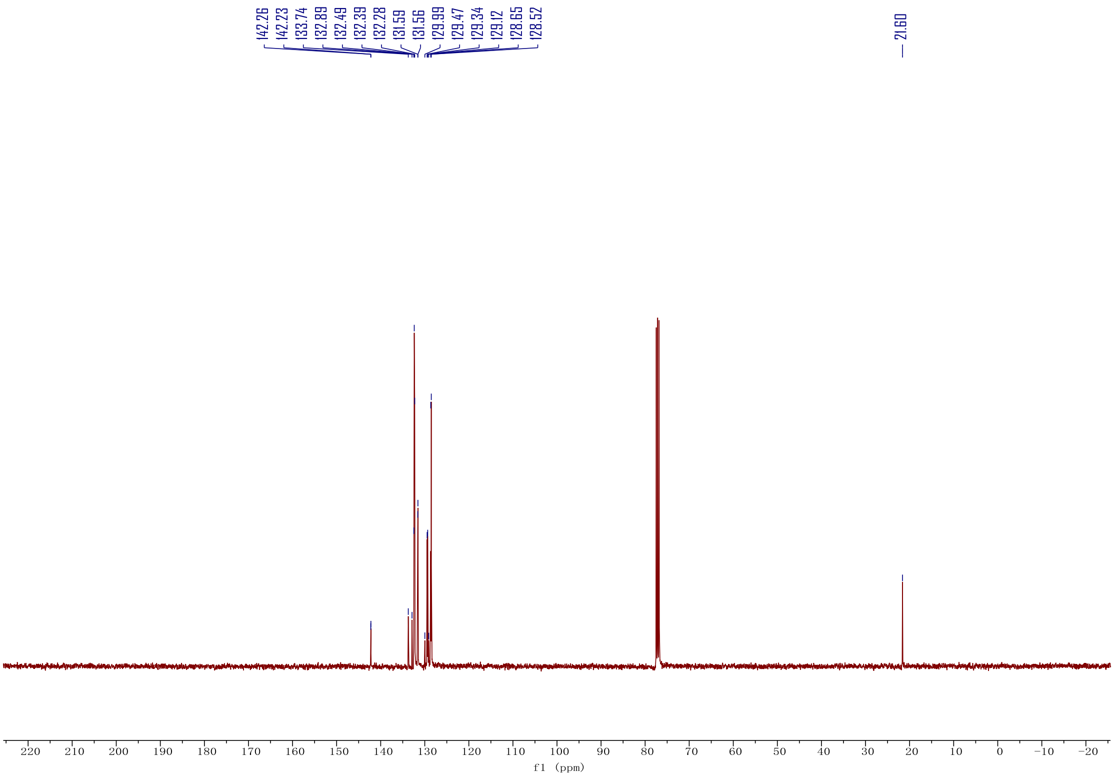

^{31}P NMR of compound **35** (162 MHz in $CDCl_3$)

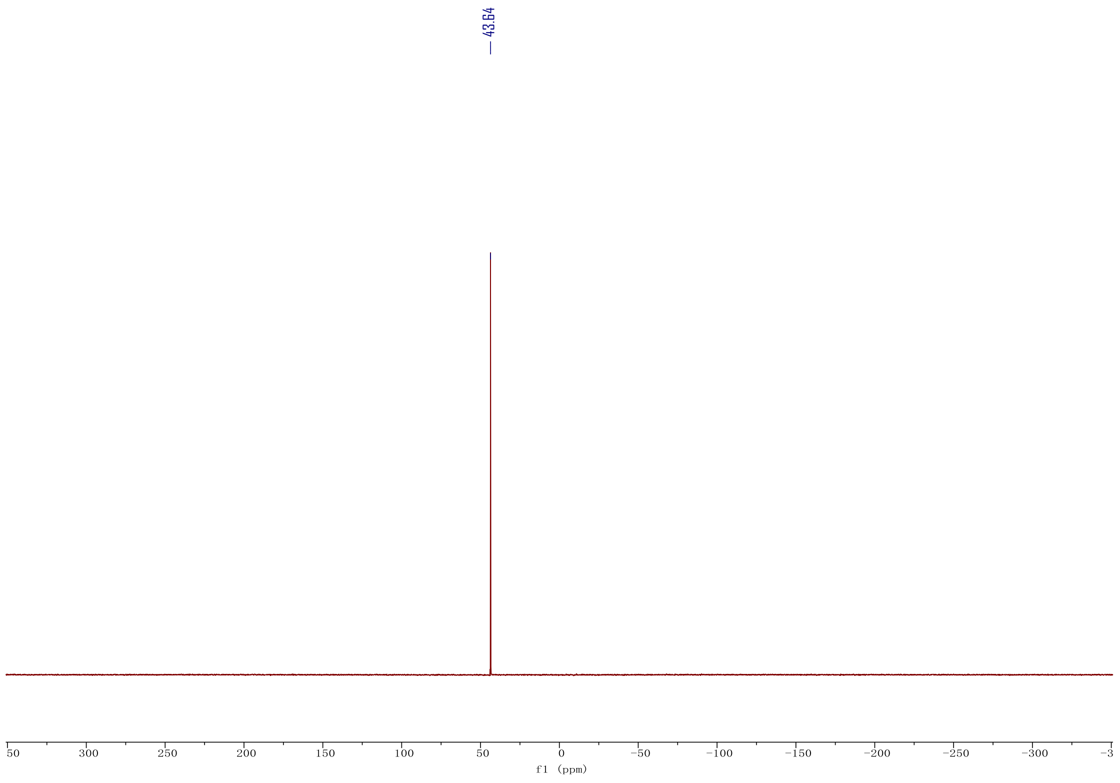

^{1}H NMR of compound **36** (400 MHz in $CDCl_3$)

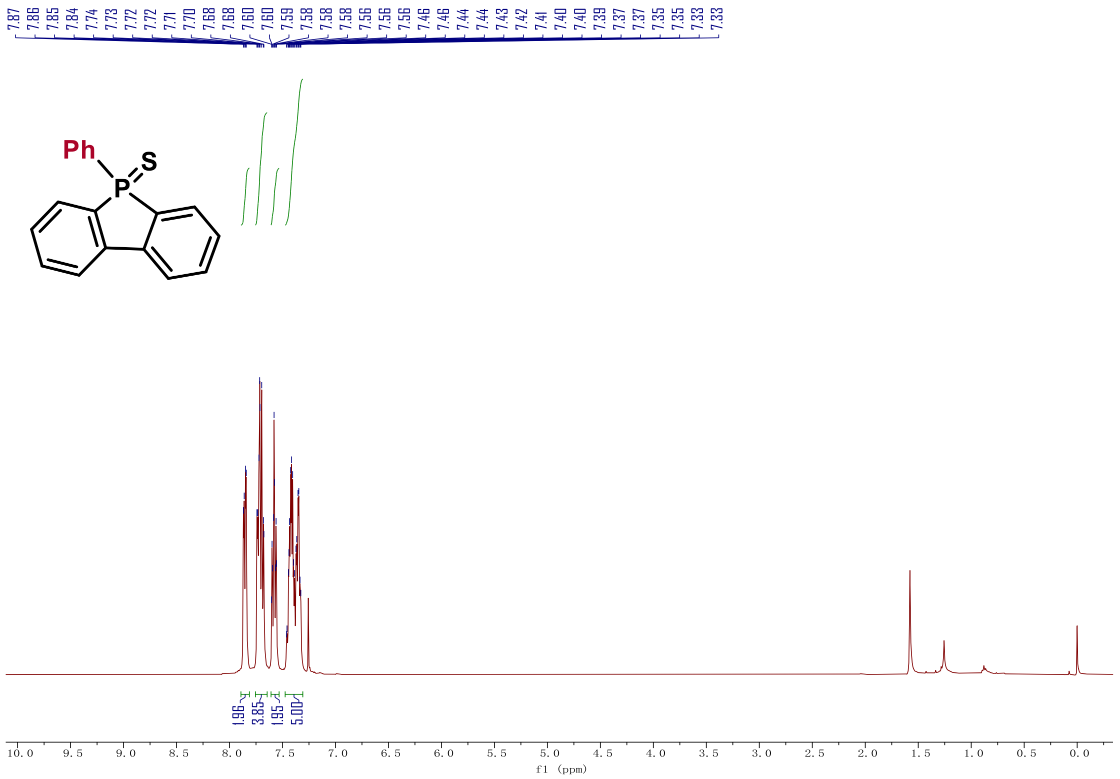

^{31}C NMR of compound **36** (101 MHz in $CDCl_3$)

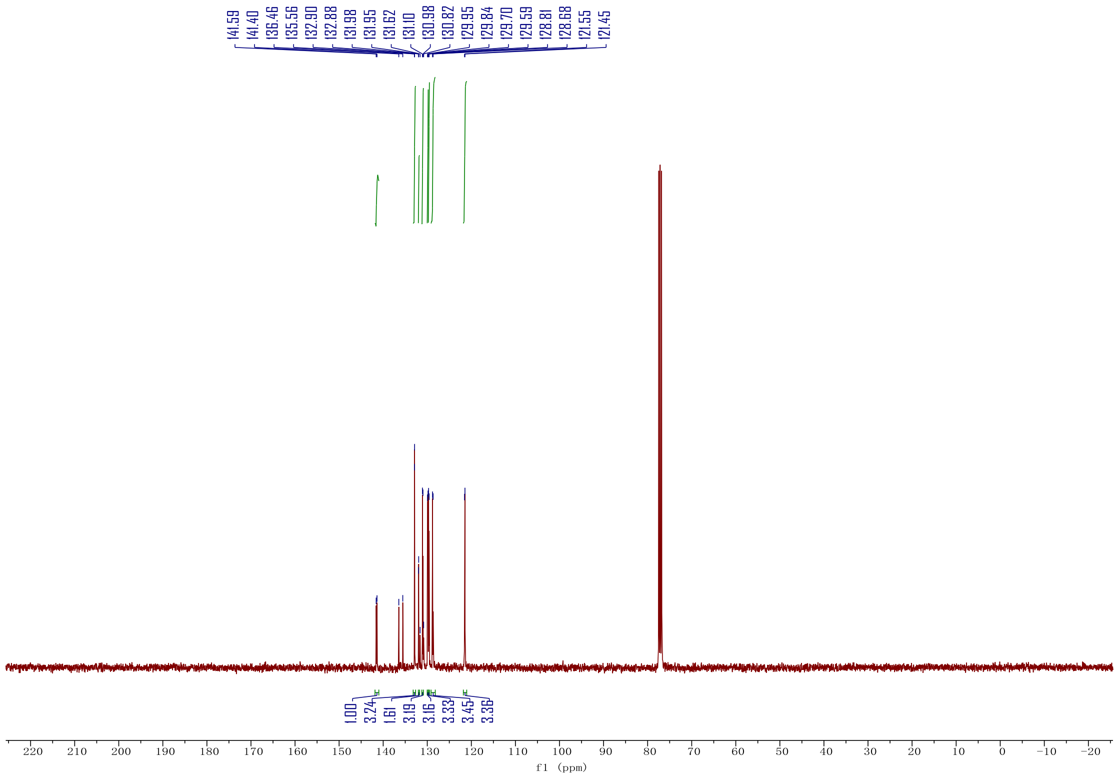

^{31}P NMR of compound **36** (162 MHz in $CDCl_3$)

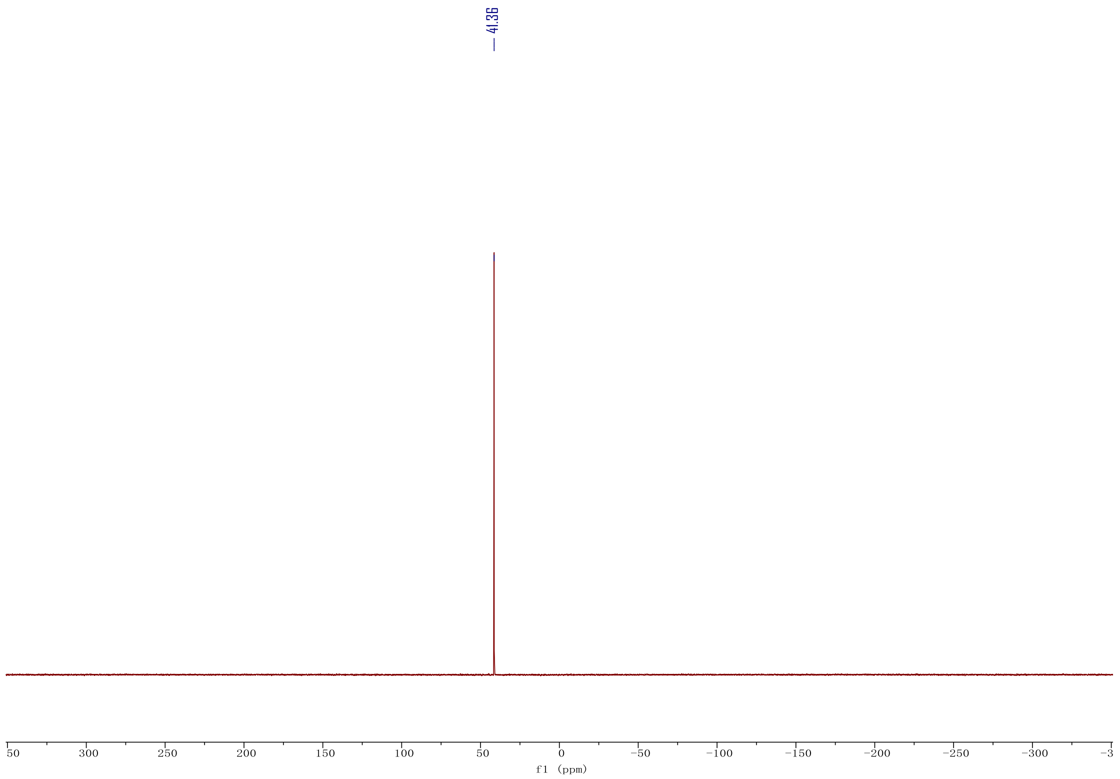

^{1}H NMR of compound **37** (600 MHz in DMSO-d_6)

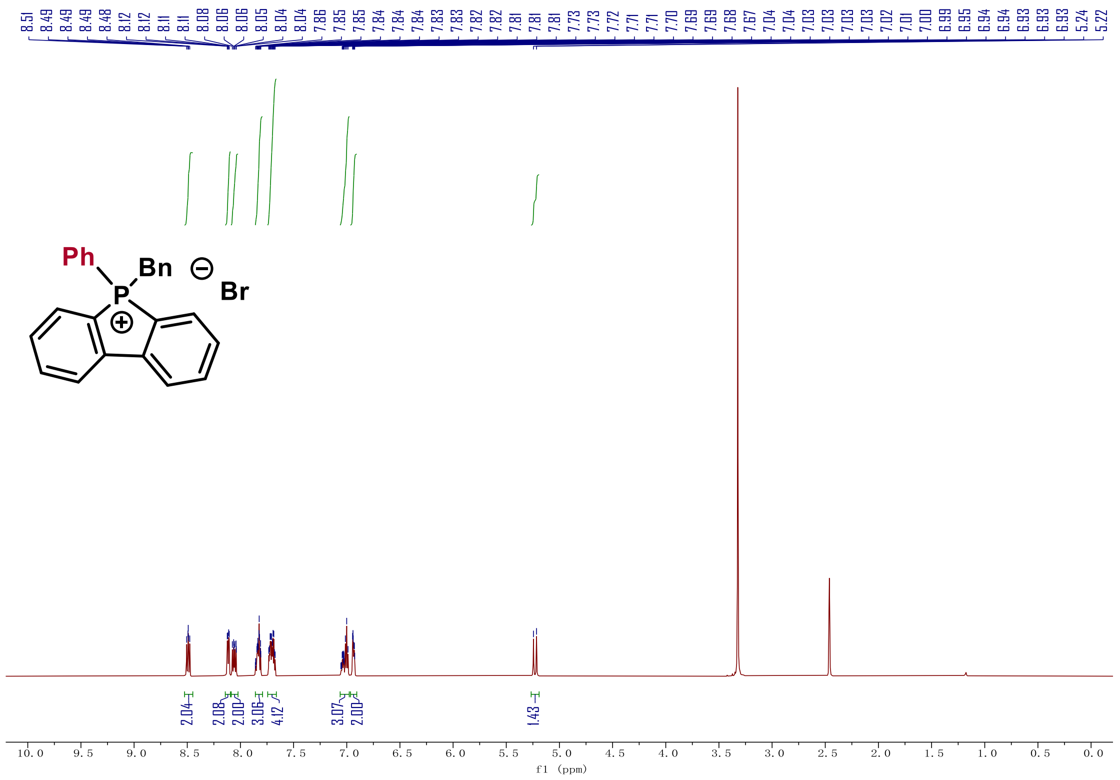

^{13}C NMR of compound **37** (101 MHz in DMSO-d_6)

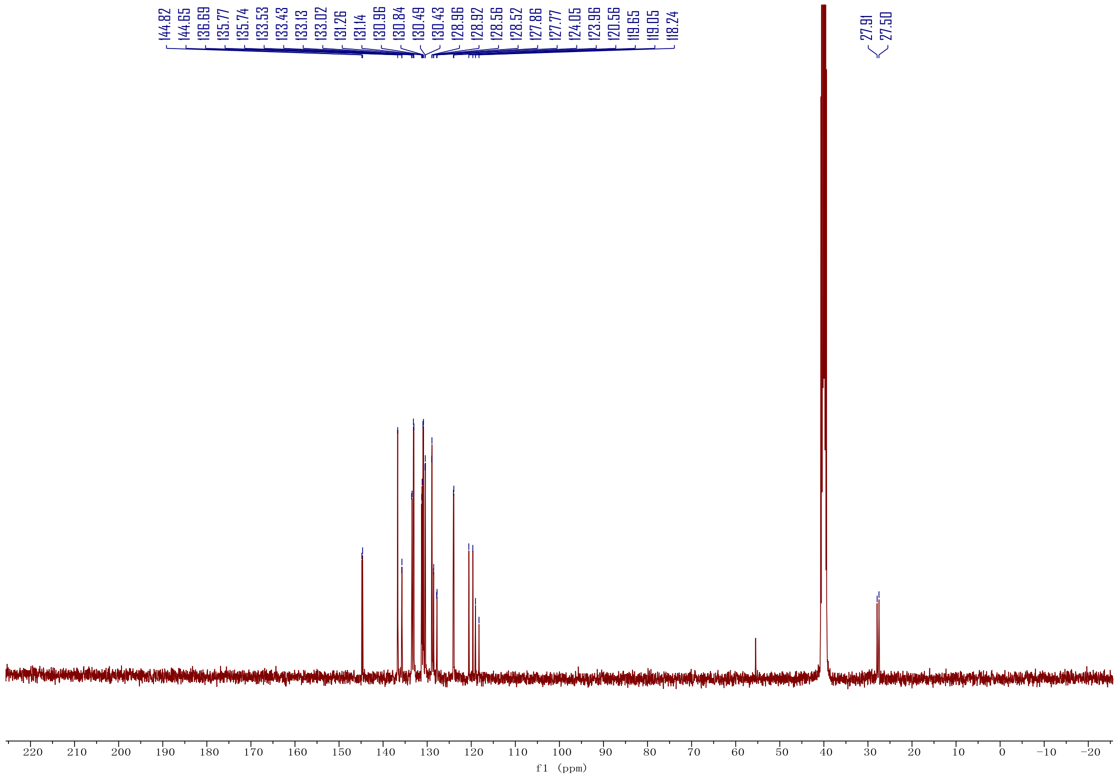

^{31}P NMR of compound **37** (243 MHz in DMSO-d_6)

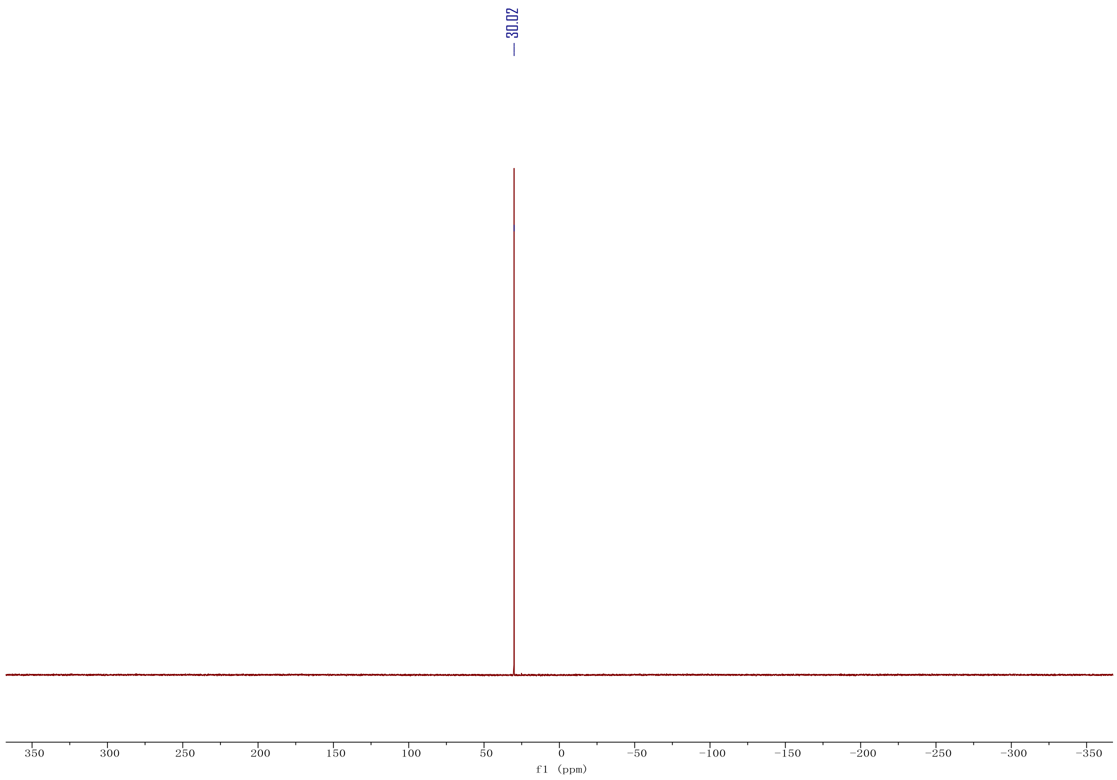

8. Single crystal X-ray diffraction of NHC-B[28]

Single crystals of NHC-**B** were grown by slow evaporation of its EtOH solution. Single-crystal X-ray diffraction data were collected with a Bruker D8 Venture diffractometer. The crystal was kept at 150 K during data collection. The structure was solved with the ShelXT program using Intrinsic Phasing method. And refined with the ShelXL-2018 package using Least Squares minimization. All nonhydrogen atoms were refined with anisotropic thermal parameters. Hydrogen atoms were placed in idealized positions and refined using a riding model. Supplementary crystallographic data have been deposited at the Cambridge Crystallographic Data Center (CCDC 2305944). Displacement ellipsoids are drawn at the 30% probability level.

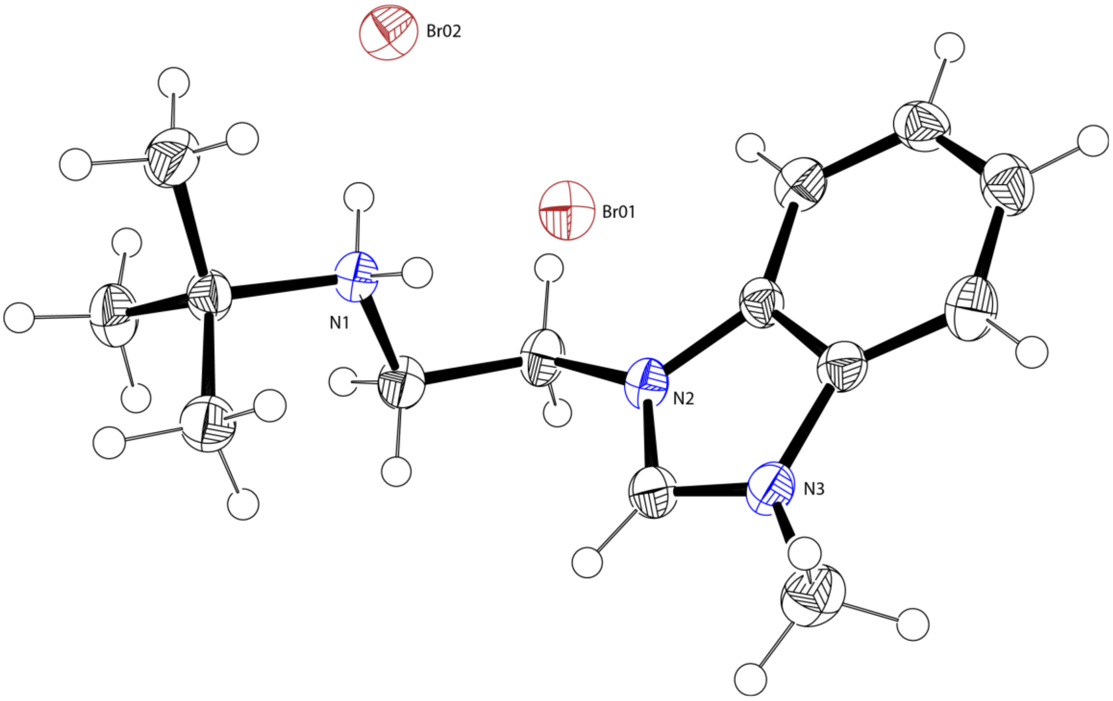

Table S1 Crystal data and structure refinement for B.

Identification code	A	
Empirical formula	C14 H23 Br2 N3	
Formula weight	393.17	
Temperature	150 K	
Wavelength	0.71073 Å	
Crystal system	Monoclinic	
Space group	P 1 21/n 1	
Unit cell dimensions	a = 5.8181(6) Å	α= 90°.
	b = 27.297(3) Å	β= 102.059(4)°.
	c = 10.6172(13) Å	γ = 90°.
Volume	1649.0(3) Å^3	
Z	4	
Density (calculated)	1.584 Mg/m^3	
Absorption coefficient	4.908 mm^{-1}	
F(000)	792	
Crystal size	0.11 x 0.1 x 0.08 mm^3	

Theta range for data collection	2.099 to 28.383°.
Index ranges	-7<=h<=6, -36<=k<=36, -14<=l<=14
Reflections collected	23110
Independent reflections	4113 [R(int) = 0.0495]
Completeness to theta = 25.242	99.9 %
Absorption correction	Semi-empirical from equivalents
Max. and min. transmission	0.7457 and 0.5883
Refinement method	Full-matrix least-squares on F^2
Data / restraints / parameters	4113 / 0 / 176
Goodness-of-fit on F^2	1.062
Final R indices [I>2sigma(I)]	R1 = 0.0380, wR2 = 0.1079
R indices (all data)	R1 = 0.0529, wR2 = 0.1160
Extinction coefficient	n/a
Largest diff. peak and hole	0.859 and -0.940 e.$Å^{-3}$

9. References

[1] J. K. Čermák, V. Církva, *Tetrahedron Lett.* **2014,** *55*, 4185-4188.

[2] W.-C. Shih, C.-H. Wang, Y.-T. Chang, G. P. A. Yap, T.-G. Ong, *Organometallics* **2009**, *28*, 1060–1067.

[3] C. Wang, R. Qi, H. Xue, Y. Shen, M. Chang, Y. Chen, R. Wang, Z. Xu, *Angew. Chem. Int. Ed.* **2020,** *59*, 7461-7466.

[4] L. Du, P. Cao, J. Xing, Y. Lou, L. Jiang, L. Li, J. Liao, *Angew. Chem. Int. Ed.* **2013,** *52*, 4207-4211.

[5] J. Li, M. Lutz, A. L. Spek, G. P. M. van Klink, G. van Koten, R. J. M. Klein Gebbink, *J. Organomet. Chem.* **2010,** *695*, 2618-2628.

[6] R. Yu, X. Chen, Z. Wang, *Tetrahedron Lett.* **2016,** *57*, 3404-3406.

[7] M. Van Overschelde, E. Vervecken, S. G. Modha, S. Cogen, E. Van der Eycken, J. Van der Eycken, *Tetrahedron* **2009,** *65*, 6410-6415.

[8] M. I. Rogovoy, M. P. Davydova, I. Y. Bagryanskaya, A. V. Artem'ev, *Mendeleev Commun.* **2020,** *30*, 305-307.

[9] C. Clarke, D. J. Fox, D. S. Pedersen, S. Warren, *Org. Biomol. Chem.* **2009,** *7*, 1329-1336.

[10] V. Diemer, A. Berthelot, J. Bayardon, S. Jugé, F. R. Leroux, F. Colobert, *J Org. Chem.* **2012,** *77*, 6117-6127.

[11] D. B. G. Williams, P. D. R. Kotze, A. C. Ferreira, C. W. Holzapfel, *J. Iran. Chem. Soc.* **2011,** *8*, 240-246.

[12] A. Haque, K. M. Alenezi, H. E. Moll, M. S. Khan, W.-Y. Wong, *Molecules* **2022,** *27*, 4253.

[13] H. Zhou, J. Zhang, H. Yang, C. Xia, G. Jiang, *Organomet.* **2016,** *35*, 3406-3412.

[14] M. Tobisu, T. Furukawa, N. Chatani, *Chem. Lett.* **2013**, *42*, 1203–1205.

[15] Y.-L. Tu, B.-B. Zhang, B.-S. Qiu, Z.-X. Wang, X.-Y. Chen, *Angew. Chem. Int. Ed.* **2023**, *62*, e202310764.

[16] K. Yin, M. Wei, Z. Wang, W. Luo, L. Li, *Org. Lett.* **2023**, *25*, 28, 5236–5241.

[17] P. Lian, K. Wang, H. Liu, R. Li, M. Li, X. Bao, X. Wan, *Org. Lett.* **2023**, *25*, 7984–7989.

[18] J. Xue, Y.-S. Zhang, Z. Huan, J.-D. Yang, J.-P. Cheng, *J. Am. Chem. Soc.* **2023**, *145*, 15589–15599.

[19] H. Ohta, Q. Xue, M. Hayashi, *Eur. J. Org. Chem.* **2018**, 735–738.

[20] S. Affandi, R. L. Green, B. T. Hsieh, M. S. Holt, J. H. Nelson, E. C. Alyea, *Syn. React. Inorg. Met.-Org. Chem.* **1987**, *17*, 307.

[21] P. A. Byrne D. G. Gilheany, *J. Am. Chem. Soc.* **2012**, *134*, 9225–9239.

[22] M. J. Frisch, G. W. Trucks, H. B. Schlegel, G. E. Scuseria, M. A. Robb, J. R. Cheeseman, G. Scalmani, V. Barone, G. A. Petersson, H. Nakatsuji, X. Li, M. Caricato, A. V. Marenich, J. Bloino, B. G. Janesko, R. Gomperts, B. Mennucci, H. P. Hratchian, J. V. Ortiz, A. F. Izmaylov, J. L. Sonnenberg, D. Williams-Young, F. Ding, F. Lipparini, F. Egidi, J. Goings, B. Peng, A. Petrone, T. Henderson, D. Ranasinghe, V. G. Zakrzewski, J. Gao, N. Rega, G. Zheng, W. Liang, M. Hada, M. Ehara, K. Toyota, R. Fukuda, J. Hasegawa, M. Ishida, T. Nakajima, Y. Honda, O. Kitao, H. Nakai, T. Vreven, K. Throssell, J. A. Montgomery, Jr., J. E. Peralta, F. Ogliaro, M. J. Bearpark, J. J. Heyd, E. N. Brothers, K. N. Kudin, V. N. Staroverov, T. A. Keith, R. Kobayashi, J. Normand, K. Raghavachari, A. P. Rendell, J. C. Burant, S. S. Iyengar, J. Tomasi, M. Cossi, J. M. Millam, M. Klene, C. Adamo, R. Cammi, J. W. Ochterski, R. L. Martin, K. Morokuma, O. Farkas, J. B. Foresman, D. J. Fox, Gaussian 16, Revision A.03, Gaussian, Inc., Wallingford CT, 2016.

[23] P. J. Stephens, F. J. Devlin, C. F. Chabalowski, M. J. Frisch, *J. Phys. Chem.* **1994**, *98*, 11623-11627.

[24] a) S. Grimme, J. Antony, S. Ehrlich, H. Krieg, *J. Chem. Phys.* **2010**, *132*, 154104; b) S. Grimme, S. Ehrlich, L. Goerigk, *J. Comput. Chem.* **2011**, *32*, 1456-1465.
[25] A. V. Marenich, C. Cramer, D. G. Truhlar, *J. Phys. Chem. B* **2009**, 113, 6378–6396.
[26] a) E. Runge, E. K. U. Gross, *Phys. Rev. Lett.* **1984**, *52*, 997-1000; b) R. Bauernschmitt, R. Ahlrichs, *Chem. Phys. Lett.* **1996**, *256*, 454-464; c) R. E. Stratmann, G. E. Scuseria, M. J. Frisch, *J. Chem. Phys.* **1998**, *109*, 8218-8224.
[27] C. Y. Legault, CYLview, 1.0b; Université de Sherbrooke, **2009**.
[28] L. Farrugia, *J. Appl. Crystallogr.* **2012,** *45*, 849-854.
